# Supporting Better Evidence Generation and Use within Social Innovation in Health in Low- and Middle-Income Countries: A Qualitative Study

**DOI:** 10.1371/journal.pone.0170367

**Published:** 2017-01-26

**Authors:** Madeleine Ballard, Jenny Tran, Fred Hersch, Amy Lockwood, Pamela Hartigan, Paul Montgomery

**Affiliations:** 1 Centre for Evidence-Based Intervention, University of Oxford, Oxford, United Kingdom; 2 The George Institute for Global Health in the Oxford Martin School, University of Oxford, Oxford, United Kingdom; 3 Saïd Business School, University of Oxford, Oxford, United Kingdom; 4 Global Health Sciences, University of California San Francisco, San Francisco, California, United States of America; University of Bedfordshire, UNITED KINGDOM

## Abstract

**Background:**

While several papers have highlighted a lack of evidence to scale social innovations in health, fewer have explored decision-maker understandings of the relative merit of different types of evidence, how such data are interpreted and applied, and what practical support is required to improve evidence generation. The objectives of this paper are to understand (1) beliefs and attitudes towards the value of and types of evidence in scaling social innovations for health, (2) approaches to evidence generation and evaluation used in systems and policy change, and (3) how better evidence-generation can be undertaken and supported within social innovation in health.

**Methods:**

Thirty-two one-on-one interviews were conducted between July and November 2015 with purposively selected practitioners, policymakers, and funders from low- and middle- income countries (LMICs). Data were analysed using a Framework Analysis Approach.

**Results:**

While practitioners, funders, and policymakers said they held outcome evidence in high regard, their practices only bear out this assertion to varying degrees. Few have given systematic consideration to potential unintended consequences, in particular harm, of the programs they implement, fund, or adopt. Stakeholders suggest that better evidence-generation can be undertaken and supported within social innovation in health by supporting the research efforts of emerging community organizations; creating links between practitioners and academia; altering the funding landscape for evidence-generation; providing responsive technical education; and creating accountability for funders, practitioners, and policymakers.

**Conclusion:**

How better evidence-generation can be undertaken and supported within social innovation in health is a previously under-operationalised aspect of the policy-making process that remains essential in order to refrain from causing harm, enable the optimization of existing interventions, and ultimately, to scale and fund what works.

## Introduction

In recent years, there has been a proliferation of projects, organizations, businesses, and research studies aimed at solving problems stemming from the inaccessibility or lack of health care facing millions of people around the world. These efforts have developed ‘social innovations,’ defined by the editors of the Stanford Social Innovation Review as “the process of inventing, securing support for, and implementing novel solutions to social needs and problems.”[1] Most studies examining how evidence is used to advance social innovations in health have found discrepancies between ideal and actual practice. Milat et al. found that while research evidence is used in decision-making, its contribution was limited by the lack of research on effectiveness and cost-effectiveness [2]. These findings were echoed by Yamey et al. who interviewed implementation experts purposively selected for their expertise in scaling-up health interventions in low- and middle- income countries (LMICs), and found that a major barrier to effective implementation was inadequate research [3]. A systematic review of barriers and facilitators of evidence usage by policymakers in any field found that the primary barrier was poor access to quality, relevant and timely research [4].

Though research on the scaling, i.e. widespread replication and/or adoption [5], of social innovations in health exists [6–10], such literature notes only that evidence plays a role in scale-up—it does not explore what *types* of data are most useful and persuasive to policymakers and funders nor how these data are interpreted and applied. Consequently, practitioners working in the field (i.e. health service providers, NGO workers) are left with little guidance on how to rigorously and efficiently assess effectiveness and feasibility of their intervention [8, 9, 11]. Moreover, these papers typically focus on a subset of actors (e.g. only implementation experts) and have not fostered a conversation across all actors required to scale programs. Though the value of evidence generation is well-documented—good evaluation enables the identification of harms [12] as well as the optimisation of existing interventions [13]—how policymakers, funders and practitioners might work together to better create and use it remains under-explored.

Given this, the objectives of this paper are to:

1. Understand the beliefs and attitudes of policymakers, practitioners, and funders toward the value and types of evidence for scaling social innovations for health
2. Understand practitioner, policymaker and funder approaches to generating evidence for systems and policy change
3. Understand how better evidence-generation can be undertaken and supported within social innovation in health.

## Methods

### Study design

As the focus of this inquiry was to provide rich descriptions of complex phenomena, illuminate the experience of actors with widely differing stakes and roles, and generate hypotheses about potential solutions, qualitative methods were used [14]. One-on-one interviews were conducted via Skype or telephone to increase participation and enable sampling from a wide range of geographies. The Department of Social Policy and Intervention Research Ethics Committee (DREC) provided ethics approval in accordance with the procedures established by the University of Oxford. Participants were approached by email and provided written consent to participate and for the researchers to publish findings. Three of those contacted declined to participate; all three said they did not feel qualified to comment on the subject. The study was reported in accordance with the Consolidated criteria for reporting qualitative research (COREQ) checklist [15].

### Participants and setting

Thirty-two interviews were conducted between July and November 2015. In order to understand a range of viewpoints, we targeted three categories of stakeholders: practitioners, policymakers, and funders. Health practitioners were sampled via the network of a prominent social entrepreneurship foundation that identifies and supports high-performing innovators (i.e. strong strategic partnerships, scalable model, diversified funding base, >1 million USD operating budget etc.). Policymakers and funders were purposively selected for their experience working with participating practitioners and, in the case of funders, their eminence in healthcare financing (>2 billion USD direct grantee support per year). Participants were classified by their primary present occupation; several participants had expertise in one of the other areas (i.e. practitioners who later became policymakers). Recruitment was on a first come, first served basis.

### Procedure

Interviews lasted 30–45 minutes and were predominantly conducted by MB and JT. Neither MB nor JT had a prior relationship with those they interviewed and participants likewise had little knowledge of the interviewers. Both MB and JT were doctoral students at the time of the study and both had previous experience working in healthcare in LMICs. A topic guide tailored to each type of stakeholder was used, which included perceived value of research, understanding of study design, experiences working with the various groups, and suggestions for improvement. The guide was piloted on the first two interviews of each stakeholder type and subsequently refined. Interviews were audio recorded and transcribed verbatim (see S1 Dataset for anonymised transcripts). No repeat interviews were conducted; no gratuity was offered to participants.

### Analysis

Data were analysed using a Framework Analysis Approach. This is a matrix-based approach to qualitative data analysis. It involves analysing verbatim transcripts for themes based on a combination of a priori issues and emergent themes [16]. This was chosen over more inductive methods because the aim of the study was to both achieve pre-set objectives and inductively explore respondent accounts.

MB and JT became familiar with the data by reading through transcripts while listening to audio recordings. The two researchers independently coded the same five transcripts before meeting to discuss key themes and draft an initial coding framework. Both researchers independently coded five further transcripts using this framework before conferring to refine it. This process was repeated until no new themes emerged and the final coding framework was finalised (see S1 Appendix for final coding tree and definitions).

A qualitative data-management package (QSR NVivo version 10.2.1) [17] was used for coding and analysis. At the conclusion of coding, data were summarised into a series of matrices, one for each theme, with one row per participant and one column per code. The matrices were analysed by making connections within and between codes and participants; this process was influenced by a priori research objectives and by concepts generated inductively from the data [18].

### Validity

Each transcript was double coded by MB and JT who met at regular intervals to compare classification of supporting quotes into codes, discuss deviant cases, and resolve discrepancies in interpretation. A draft of the paper was returned to participants for comment and/or correction on quotes and feedback on the findings.

## Results and Discussion

14 practitioners, 12 funders, and 5 policymakers working in eight LMICs were interviewed (Table 1). There were no notable discrepancies in data interpretation between co-researchers.

**Table 1:** Participant Characteristics.

		Practitioners	Funders	Policymakers	Total
		N (%)	N (%)	N (%)	N (%)
**Gender**	Male	10 (71)	9 (75)	5 (83)	24 (75)
	Female	4 (28)	3 (25)	1 (17)	8 (25)
**Role**	Founder /Leader	6 (93)	3 (25)	N/A	16 (50)
	M&E* expertise	3 (21)	0 (0)	0 (0)	3 (9)
**Focus^**	Specific Country	7 (50)	0 (0)	6 (100)	13 (40)
	International	5 (36)	12 (100)	N/A	17 (53)
	Developing Country Focus	12 (86)	12 (100)	5 (83)	29 (91)
**Occupation**	Manager/ Executive	12 (86)	11 (92)	N/A	23 (72)
	Analyst	2 (14)	2 (17)	N/A	4 (13)
	Researcher/ Academic	3 (21)	0 (0)	N/A	3 (9)
	Clinician	4 (29)	0 (0)	N/A	4 (13)
	Minister	0 (0)	0 (0)	6 (100)	6 (19)

*M&E: Monitoring and Evaluation

^ Individuals worked in a large range of geographic regions: Caribbean; Central and South America; Central, Southern, and East Asia; East, West, Central and Southern Africa; and the Middle East and North Africa.

### 1. Beliefs and attitudes about evidence

#### Practitioners

Practitioners conceived of the utility of evidence in three primary ways:

I. Research as an accountability mechanism

Several practitioners highlighted outcome evaluations, which measure the change that has occurred as result of a programme, as a way to ensure recipients of their program benefitted as intended. They pointed out the necessity of measuring programme effectiveness as a natural extension of their obligation to provide essential services.

Research is a tool of justice…how are we holding ourselves accountable … to whether or not ideas that we have are helping anyone or hurting people. If we don't measure, we don't know. (P2)

One practitioner, however, was sceptical of the alignment between what practitioners claimed they believed about research and the rigor with which they follow through on these convictions:

When you really get down to it…no one really wants their work to be looked at with a fine-toothed comb. (P12)

II. Research as an opportunity to refine service delivery.

Process evaluations, which examine the implementation of an intervention, were cited as a means to identify “where [services] can be improved” (P5, P6). For example, one practitioner was able to use the variability between programme sites to refine operations in places that were underperforming:

… sites cross-share data [enabling them] to both critique and ask questions as to why their retention numbers are so high or so low, what kinds of things are they doing that might be different… (P10)

III. Research as a means of fulfilling expectations of funders and policy-makers.

Another group cited external pressure as the primary reason to conduct outcome and operational research. While they saw research as a burden (“we tend to do what we sort of have to do,” P1) or were passive decision-makers in conducting research (“we define our success by meeting our targets as determined by whatever funder’s proposal or whatever funder’s requirements are,” P12), others viewed building a joint research agenda as a positive, synergistic process. This is especially true of those practitioners working closely with the Ministry of Health (MOH) in their respective countries. By aligning or jointly developing research questions with the MOH, practitioners were better placed to scale nationally (P11) and create the trust necessary for novel approaches:

If we had gone into these areas with the Ministry and said: “Oh we actually want to do something that is completely different than your current national policy,” the Ministry would have blocked it…the research we did together…opened the door to…joint innovation. (P2)

While practitioners are in favour of research, they are not convinced that funders and policy-makers use it to make decisions. One practitioner declared, “a huge amount of interventions that happen in the global health space…are scaled in an evidence free zone” (P2). Several practitioners believe funders have a groupthink dynamic and suggested personal relationships often trump evidence:

We just got a call from [a funder] on Friday, potentially giving us a million dollars to launch this [program]… as soon as one drops, others will drop… (P4)

As in Yamey [3], practitioners asserted that funders are more moved by stories or personal connections than rigorous counterfactuals, preferring emotional appeals (e.g. individual testimonies) to population-level data:

I mean we do report to donors…[but] that's much more… stories than it is an analysis. (P1)

Because if someone said, “give us evidence” we bring out someone who can tell a powerful personal story…there's a donor base that responds to [anecdotes] and doesn’t respond to “… RCTs and … confidence intervals”. (P4)

While some practitioners conceded that this trend was changing—“in the coming years organisations that are not [becoming] evidence-based, they [will] face challenges in raising resources to support their work” (P14)—most felt that funders in particular did not understand evaluations well enough to critically appraise study designs and evidence quality:

Few people understand…the difference between the clustered-randomized trial and one that’s just randomized. (P6)

#### Funders

While funders say they hold outcome evidence in high regard, this is often not translated into practice. Funders spoke of the importance of external evaluations (F4), strong data systems (F1) and ultimately “creating an impact” (F4). Yet for several of the funders, especially those from family and corporate foundations, identification of potential grantees is not systematic and often based on referrals through "networks" of trusted partners (F1, F2, F10, F11). While most stressed that referrals guarantee closer scrutiny and not funding; two admitted that “it’s a bit of an echo chamber” (F2) and that “the deck is [potentially] stacked against” organisations without connections (F10). Moreover when asked how they evaluate potential organisations, funders cited organisational health metrics (F2), “visionary” leadership (F11), or novelty, rather than outcome evaluations:

Sometimes it’s just a gut feeling. It might be something very new. It might be untested, but you kind of have a sense that there’s real excitement. (F9)

Even when funders cited clear outcome metrics (e.g. “DALYs [disability adjusted life years] averted, return on investment,” F7) they admitted they did not critically engage with the underlying studies. Several had misunderstandings of technical details in evaluation research or admitted research expertise was lacking on their teams:

Frankly, we don’t have program officers who are extensively trained in M&E. (F7)

Others questioned whether, as funders, they could ever adequately assess a given programme:

I think one of the things you have to accept when you’re funding projects…is that reporting on an application to its owner is an honour-based system. (F5)

There was recognition that funders could be driving the “evidence-free zone”:

Most organisations don’t effectively measure their own impact because the funders don’t ask them to and the funders don’t ask them to because they don’t really understand it well enough or it’s … not in their best interests to ask for it…this sector has simply been … “put hopeful money in and be unclear on what you get out”. (F1)

That said, funders are not a homogenous group, and several echoed practitioners on the idea that times were changing and had clear ideas about the types of evaluations they would like to see:

We feel like [strong M&E] is now required by the field to scale and it’s just healthy…to know what impact you’re having. (F11)

We’d love to see more organisations pay attention to meaningful, concrete quantification of their work (e.g. pilot, larger trials)… (F3)

While some funders were very understanding of the challenges faced by organisations to generate quality evidence—noting the difficulty of gathering real time data (F6) or the challenge of doing qualitative research without “sufficient money” (F9)—other funders expressed their own frustrations with NGO and health service practitioners:

Many organisations either don’t think very systemically about what they should measure to either show an impact or…how to iterate…their approach. Often, [it’s] implementation of an idea without clear testing beforehand. (F3)

We would like obviously the grantees to have a strong theory of change…for that to be laid out in terms of a results framework…I think that’s where a lot of the grants frankly are lacking. (F7)

#### Policymakers

Policymakers also mostly held evidence in high regard—e.g. “We’ll never take things on that haven’t worked yet,” (M6)—but were much more likely to be explicit about the types of evidence they valued to identify interventions to scale. As one Director General of a MOH in West Africa explained:

New interventions are basically selected based on… if an intervention is proven to be effective…especially in similar settings, and if there are very strong evidences. (M3)

One policy-maker however, admitted to not rigorously reviewing the evidence for solutions implemented: “I think the [research] evidence of those things… I’m assuming that it’s already known… but honestly I didn’t read anything recently.” (M5)

Consistent with earlier findings [7, 18], they revealed a preference for local evidence—“the fact that it works in Bangladesh doesn’t mean that it works in [country name]” (M2)—however, there was also a willingness to pilot ideas from different places:

There are some interventions that you need to do a pilot … and if those interventions are proven to be effective after the pilot, then you go for them. (M3)

Policy-makers also recognised the challenges of generating evidence and did not always demand that evidence for policy and program interventions be generated with the same level of rigor as that required in clinical trials for drugs and diagnostics:

When we find out that the benefits outweigh the risks, we sometimes say to them, “Go” without necessarily having the highest of all evidence, [a randomised trial], and then we monitor and rule on that as we go. (M6)

Though, in keeping with previous literature [2, 4], policymakers noted that evidence is only one factor when making a scale-up decision, conceding that interventions aligned with national priorities (M2) or introduced by trusted colleagues (M3) are more likely to considered. They roundly rejected, however, the idea that personal ties were sufficient to bring something to scale:

What you bring—the quality of product that you bring—is very important, the amount of evidence, and the evidence of the impact this intervention had in other countries is very important. So, it’s not only the question of who brings it, but what contribution could it make [based on] the evidence. (M2)

### 2. Approaches to evidence generation

Practitioners face several barriers in undertaking research. A central tension is striking the right balance between rigor and operational relevance. While practitioners conceded that exacting methods would make their results more convincing—“in an ideal world I would want to see … more academic rigor… to really figure out how … you prove this” (P3)—many expressed concerns that such “over the top” designs could “slow [them] down” (P3, P2, P14).

Compounding this issue is the difficulty of finding accessible technical expertise. Almost every practitioner had a technical query they could not answer (see S1 Dataset). Practitioners also highlighted human resource shortages (P14), difficulty identifying useful resources (P10), and above all, the expense they perceive to be associated with rigorous research: “my first evaluation cost three times my [programme] budget. Now that is wrong” (P4).

Perhaps as a result, most of the organisations interviewed, despite being sampled from a group recognised as high performing (i.e. strong strategic partnerships, scalable model, diversified funding base), have never conducted longitudinal research, used counterfactuals, or identified which parts of their program mediate its effects

More troubling is the finding that practitioners, funders, and policymakers have given little systematic consideration to potential unintended consequences of the programmes they implement, fund, or adopt. While a small number were open about harms, the vast majority did not have rigorous systems to measure harm, and several denied that harm was possible.

Two practitioners talked very explicitly about harm, one due to unintentional exposure to environmental risks for staff and the other as a result of aggressive implementation leading to burn out, reductions in quality of delivery and thus potentially endangering patients (P2, P10). Both noted the role of constant data collection in being able to identify these damaging patterns and stressed that recognising such pitfalls was “as important, if not more important” as measuring programme success (P2).

Many more practitioners, however, were not able to articulate how their programmes measure potential harms:

That’s just a fascinating question. We have not dug in there. (P13)

There are no ‘checks and balances’ that we put in place other than following the already predefined country rules and regulations. (P14)

A small number were vigorously defensive about their organisations, dismissing the possibility of any ill effects or their severity—even after repeated probing:

I’m a little stuck to answer that. I think there’s times when we don’t always do our work as well as we’d like but I think in the world of health, what we’re doing is…I’d hate to sound self-righteous, but it’s fairly noble. (P9)

Funders fell into a similar taxonomy, with some denying it (“I actually think in this space that’s not a serious issue,” F6) and most having failed to consider it systematically (F7):

Well, within the health space, we are really focused on the distribution of health services and goods so in terms of the harm aspect we haven’t spent a lot of time thinking about that. (F11)

One difference is that, among those who had considered it, funders felt less likely to hear of harm or empowered to stop harm than practitioners:

We also fund only people with really good monitoring systems in place so that untoward consequences, unintended consequences, should be picked up fairly early… I don’t know anybody who has a fool proof way of avoiding unintended consequence. (F1)

My parents have asked me, “Well, what does the foundation do in situations [where harm has been caused]?” …and I don’t know. (F7)

### 3. Doing better: supporting evidence generation and use

While most enquiries into scale and evidence generation have only spoken to one set of stakeholders and have thus collected a series of claims by one group about the failings of the other [4, 19], by speaking with all groups concurrently we were able to convene a constructive discussion and guide the identification of areas in which actionable strategies for improvement could be generated.

#### 1. Supporting the research efforts of emerging organisations

Research is often side-lined during the early stages of organisational development, despite its importance. Practitioners cited the importance of establishing data collection mechanisms in the early stage of a new organisation as it is “costly” to compensate for poor or non-existent data down the line (P2, P8). Yet, as one practitioner points out, young organisations face a catch-22 when attempting to grow their programs and data systems:

It's rough out there…community service organisations …don’t get funded because they don’t have evidence, and they don’t have evidence because they don’t have the money or the smarts. (P4)

There is also the tension between exacting evidence standards without ‴extinguishing’ innovation and small community generated projects” (P4). Practitioners suggested prioritising identification of promising early-stage health organisations and supporting them to create "meaningful evidence…[when they] don't have the human resources and the financial resource" (P4). This includes “building the capacity of human resources” from LMICs, including local researchers and practitioners, as a central part of measurement and implementation (P9).

#### 2. Creating links between practitioners and academia

Practitioners and funders noted that “academic research isn’t set up to match the most qualified investigators with implementing organisations that are trying to take on [big] problems” (P2). While, for example, the Economic and Social Research Council (UK) and other programs attempt this, all parties suggested an additional formal mechanism for partnerships be created and supported.

Though organisations acknowledge that there are challenges in finding the right academic fit—“culture shock” (P6), “misaligned priorities” (P2)—many touted the benefits that academic expertise could bring: “[the academic team] made a control group…added measurements…[assessed] the long-term impact…we use [those] results to do advocacy”(P5). While others noted that an academic affiliation is typically required to receive ethics approval (P4).

Echoing the findings of Yamey [3], respondents noted that the comparably lower standing of implementation science among researchers was a barrier to well-designed, applicable research; raising the status of implementation science is imperative, especially to attract young academics to the field (F8).

… one of the things they’re working on at Harvard [and Johns Hopkins] is…recognising good, operational research as part of someone’s academic portfolio so that that gets rewarded in the tenure system. (P9)

Even so, it was acknowledged that despite a few appointments based on these new criteria, there was a long way to go to change the perception that success only comes via “hardcore first and last author papers” (F8). A potential stopgap measure while this larger structural change takes place would be for funding agencies to provide small stipends for academics to be “fellows” (e.g. similar to IDEO) at global health NGOs (P6).

#### 3. Change the funding landscape for evidence-generation

While universities are partially responsible for structuring what type of research gets undertaken, the research grant-making process, especially in the United States, is another driving force:

There are constraints on trying to get implementation science funded right now in the U.S. … there’s no National Institute of Implementation Science. So, implementation science or operations research is actually quite difficult …because it’s not obviously [funded] in one place or another. (F8)

Some practitioners have found ways to "game the system" by finding academics who are willing to give a portion of their time ("either as a donation or as a portion of their FTE [full time equivalency]") However, because they are "not on traditional NIH [National Institutes of Health] pathway" doing research requires mobilising large amounts of unrestricted funding that can be hard to come by:

All your donors and your partners, all want lot of reporting and a lot of M&E stuff, but nobody really wants to pay for it. (P1)

Often the last year of the program, people are scrambling so much for new money…I’m being paid to analyse data…but [right now] I’m writing [funding] proposals. (P12)

One funder identified a funding gap as organisations grow:

I’d like to see kind of a funder continuum…it’s a valley of death once you get to be like a 5 or 8 million dollar a year organisation…everyone wants to give $100,000 or $250,000 but no one wants to write a seven-figure check. (F10)

The lack of unrestricted funding for research makes it harder to train local staff (P9), attract and retain qualified researchers (P14), build robust M&E platforms (P14), clean existing data (P8, P14), and conduct rigorous trials and in-depth qualitative work (P6):

The ideal scenario is…if you look at any major tech company; they have an internal R&D department. Justifying funding for that is not complicated because they make a lot of money. Figuring out how to justify internal R&D departments at global health NGOs is super important. (P6)

Unrestricted or research-oriented funding must be made available to encourage robust research.

#### 4. Lean research and technical education

While several practitioners and funders accused researchers of “misplaced precision” (F8) they also valued “rigorous” research. Practitioners identified the need for “approaches to evaluation…readily off the shelf [that organisations could] use as tools to actually implement evaluation” (P10), smaller, “real-time” studies that enable organisations to “course-correct” as they go (P12, F6), and cheaper evaluation methods (P4).

When people talk about evidence-based decision making… A lot of the programme teams don’t have that kind of analytic training. (P12)

Such components could form part of a toolkit that would enable practitioners to easily perform unobtrusive, light touch—“lean”—research in everyday practice. Though others cautioned against re-inventing the wheel, noting that “a lot [of material] is already out there” (F2), suggests that an essential complement to any toolkit must be education regarding how to search for and understand existing strategies.

#### 5. Creating accountability

A more profound and less easily operationalized suggestion was given voice by one practitioner:

[We often say] we are doing things to “prove a model” etc. That presupposes that what we're doing already works and that we figured everything out and sometimes precludes opportunities to really take measurement as our opportunity for learning and for accountability, because a lot of global health interventions fail and a lot of great ideas don't end up working out. (P2)

One funder noted, “organisations are not as…eager and willing to do a good job of implementing a high-impact model that they did not themselves develop” (F1). Rather than believe that individual organisations will solve any given problem, funders suggested that “most” practitioners “don’t have all of the pieces” and so should think of themselves as part of a larger team that should work together to solve a project (F6). While one practitioner mentioned her organization actively encouraged the adoption of any program with similar goals and outcomes (P13), the vast majority were focused on scaling their particular model. The ways in which this individualistic, competitive climate is encouraged by the celebration of social entrepreneurs and the conceptualization of social innovation in health as “a business” must be considered.

Several policymakers were adamant that practitioners also needed to be held accountable for making early and transparent contact with the MOH in their respective countries. As one MOH representative from East Africa explained:

NGOs [should] come direct into the Ministry…presenting what they have and how this could be integrated with what the government is doing… Continuously engaging with the Ministry…is very important. Sometimes, [practitioners] go somewhere without informing the Ministry and [do] something which is contrary to the … position of the Ministry…which is a disaster…because that resource is simply spent in vain in something that the Ministry doesn’t appreciate. (M2)

Ministries of Health must likewise be held accountable for creating an environment that allows social innovation to flourish in their countries. This means proactively developing relationships with NGOs working in their countries to ensure alignment and create opportunities for collaboration and piloting new ideas. Moreover, as one funder asserts, “once something’s been proven with world-class research to work, the governments [should] free up resources to actually implement [it]” (F10).

Funders recognize, however, that they too need to improve:

I think there’s a lot of bad behaviour on the funders’ side: when they do single year and when they do restricted, when they take a lot of people’s time and make them jump through all kinds of hoops and then don’t use the information at all for anything. (F10)

As one funder put it, “I don’t know if you can think of any foundation head who’s ever been fired for lack of impact, so why would you even measure it if it isn’t really that important?” (F1). One way of creating accountability for funders may be through the use of a priori evidence standards. The Social Research Unit at Dartington (UK) offers a framework for evidence standards based on questions around practicality, rigour, outcomes, side-effects and replicability [20]. Their application has not been without difficulty–less than 4% of the initial 240 applications to one government program met the standards, indicating a lack of evidence-based programmes from which funders and policymakers could choose. While it is possible to disagree with the content of the standards, the fact that they exist proves that it is possible to pursue impact in a transparent, rigorous fashion and have this vision adopted by governments, funders, and implementers alike.

## Conclusion

Thirty-two one-on-one interviews were conducted with purposively selected practitioners, policymakers, and funders from LMICs to explore (a) beliefs and attitudes towards the value of and types of evidence in scaling social innovations for health, (b) approaches to evidence generation and evaluation used in systems and policy change, and (c) how better evidence-generation can be undertaken and supported within social innovation in health.

While practitioners are largely convinced of the merits of rigorous research, they face several barriers in undertaking it. A central tension is striking the right balance between rigor and operational relevance; an issue compounded by the difficulty of finding accessible technical expertise. Practitioners assert that funders and policymakers are more moved by stories or personal connections than rigorous counterfactuals, but these assumptions were not necessarily borne out in decision-maker narratives. Though funders didn’t typically question or critically appraise the evidence of effect with which they were presented, policymakers tended to be very explicit about the types of evidence they valued when considering interventions as candidates for scale. In several areas, funders and policy-makers align with practitioners.

One area where practitioners, funders, and policymakers converge is that few have given systematic consideration to potential unintended consequences of the programmes they implement, fund, or adopt. While a small number were open about harms, the vast majority do not have rigorous systems in place to track harm, and a number are in denial that causing harm is even possible.

By speaking with all groups concurrently we were able to convene a constructive discussion and guide the identification of areas in which actionable strategies for improvement could be generated. Those that emerged were: supporting the research efforts of emerging organisations; creating links between practitioners and academia; altering the funding landscape for evidence-generation; providing responsive technical education; and creating accountability for funders, practitioners, and policymakers.

### Strengths and weaknesses of the study

Only by fostering a conversation across the entire ecosystem of actors required to scale programs can entrenched issues be confronted in creative, mutually agreeable ways. This is the first qualitative study to simultaneously explore practitioner, funder, and policymaker views on evidence generation and scale. Three limitations of the study were (i) policymakers proved more difficult to secure for interviews than expected and so theoretical saturation was not reached for this group, (ii) researchers were not sampled as a separate group, despite emerging as a major actor in the discussion, and (iii) the relatively short interviews time (~35 min average), while enabling a large and relatively diverse sample, meant less depth was achieved with each participant. The credibility of this study was nonetheless enhanced by the use of multiple analysts to independently code data and review interpretations.

### Implications

The findings of this study highlight issues that are relevant to enabling better evidence generation and use in health by practitioners, funders and policymakers alike. Because this inquiry pushed participants for specific, actionable suggestions, the “doing better” proposals provide deep insights into evidence generation, understanding ‘pain points,’ and ideas for potential solutions.

Better evidence is not a panacea—taking interventions to scale is a complex process that requires the recognition of the importance of evidence in decision-making, understanding between practitioners, funders and policy-makers and the use of a variety of evidence typologies. Nonetheless, how better evidence-generation can be undertaken and supported within social innovation in health is a previously under-operationalised aspect of this process that remains essential in order to refrain from causing harm, enables us to optimise existing interventions, and ultimately, to scale and fund only what works.

## Supporting Information

S1 Appendix Final Coding Tree and Definitions. (DOCX)

S1 Dataset Anonymised Rough Interview Transcripts. (ZIP)
