# Supplementary material for: Supporting Better Evidence Generation and Use within Social Innovation in Health in Low- and Middle-Income Countries: A Qualitative Study
Source: PLoS One. 2017 Jan 26;12(1):e0170367. doi: 10.1371/journal.pone.0170367 (PMC5268497; doi:10.1371/journal.pone.0170367)
Supplement: S1 Appendix — (DOCX) [file pone.0170367.s001.docx]

Final coding tree and definitions:

| **CODES** | **DEFINITION** | |
| --- | --- | --- |
| **Doing research** |  | |
| - Rationale/Motivation to do research | What motivates the organization to do research | |
| - Outcome indicators | Put all outcomes (process, organizational, health, economic etc.) under this node. | |
| - Study design | Descriptions of study designs used or not used/why (specific study conducted, thoughts about RCTs) | |
| - Data Collection | How data is collected | |
| - Costs descriptive | How much does it cost to run your M&E (money that exists) | |
| - Harm | How harm is conceptualized, measured, not measured… | |
| - Manpower descriptive | What people (#, job description) run M&E | |
| - Internal evidence usage | How orgs who collect data use the data | |
| - Examples |  | |
| **Doing better** |  | |
| - Barriers no solution | Insurmountable challenges | |
| - Costs conceptual | Money that doesn't exist (yet!) | |
| - Manpower conceptual | Manpower that doesn't exist (yet!) | |
| - Advocacy | Groups pressuring funders/decision makers on behalf of issue/concept | |
| - Evaluation technical input | Help with technical aspects of evaluation research | |
| - Communication (external) | Individual orgs learning to pitch their model to funders/decision-makers | |
| - Best practice sharing | Making information available to other practitioners on how to do things | |
| - Education | Education of decision makers (or staff internally) by org or group on how to understand and value rigorous research and its importance | |
| - Community ownership | Input from target population | |
| - Early stage support for orgs | Challenges in setting up research/M&E capability in early stages | |
| - Academic pathways | Discussion of changing tenure system, rewarding/valuing operational research | |
| - Funder accountability | Funders held accountable for impact | |
| **Exerting influence/Knowledge Translation** | |  |
| - Environmental | Context (e.g. crisis, popular opinion). Opportunities that exist a priori | |
| - Personal relationships | Interpersonal familiarity that leads to policy change, possibly in absence of other forms of persuasion (e.g. evidence...) | |
| - Emotive (stories, photos) | Stories, photos etc. that elicit primarily an emotional response | |
| - Formal evaluation | Policymakers/funders being convinced by outcome evidence, counterfactuals, external evals | |
| - Education | How educating decisionmakers helped them use research to make decisions | |
| **Descriptions of working w/external actors** | | |
| - Interactions w/funders | As per node name | |
| - Interactions w/policymakers | As per node name | |
| - Interactions w/academia | As per node name | |
| - Interactions w/industry | As per node name | |
| - Interactions w/other | As per node name | |
| **Background** |  | |
| - Org mission | 1-3 sentence statement of org's mission | |
| - Big question | The question(s) about programs/efficacy etc. that the org would like to answer but has not | |
| - Org Issue background | Background to the issue the organization is working on | |
| **Funder** |  | |
| - Criteria | The criteria funders want fulfilled before funding an org | |
| - Identification | How funders find orgs to evaluate | |
| - Support provided | What funders provide (e.g. guidance, money etc.) | |
| - Rationale | Why they collect the info they collect from orgs they already fund | |
| **MOH** |  | |
| - Criteria | The criteria MOH want fulfilled before supporting an org | |
| - Identification | How MOH find orgs to evaluate | |
| - Support provided | What MOH provide (e.g. guidance, money etc.) | |
| - Rationale | Why they collect the info they collect from orgs they already fund | |
