# Supplementary material for: Supporting Better Evidence Generation and Use within Social Innovation in Health in Low- and Middle-Income Countries: A Qualitative Study
Source: PLoS One. 2017 Jan 26;12(1):e0170367. doi: 10.1371/journal.pone.0170367 (PMC5268497; doi:10.1371/journal.pone.0170367)
Supplement: S1 Dataset — (ZIP) [file pone.0170367.s002.zip › Data/Data - Interview transcripts/M3.doc]

**Interviewer:** I want to ask you first; maybe you could just explain your role in the ministry of health in your country, just briefly for the record? [00:00:09]

**M3:** OK. I'm the Deputy Minister of Health responsible for relief settlements and public health. Also, I’m the chair head, the national head in Government Donor Respond. You know that all these countries in the past few months and year have been battling the Ebola virus disease.

I'm in charge of the respond in our country. Prior to that, I was the Assistant Minister of Health for Disease Prevention that is looking at the special diseases, non-communicable diseases, mental health supply chain, and all of that. Yes, that's my role in the ministry of health. [00:01:10]

**Interviewer:** Lots of work. My first question is how did you hear about this health organisation? [00:01:28]

**M3:** This health organisation, I had colleagues working in the ministry of health. I think it was two years ago. A colleague working in the ministry of health was working in a South African country -- I think maybe it's either Botswana. I think it’s Botswana or one of them.

This colleague was working with the WHO, I think as the WHO country representative in a certain African country. She proposed that an organization that can move medical supplies, and vaccines to the last [inaudible 00:02:23] to ease transportation exist there – this health organisation.

She did propose that through the Chief Medical Officer of our country who's also a Deputy Minister. The Chief Medical Officer did ask me to look at this and did some [thesis] as to how to move about. I quickly got on the website of a health organisation, and I saw the presentation of how they work, and was able to invite them to our country. That's how the ball started to roll. [00:03:11]

**Interviewer:** What is it that made you really trust this organisation, think, "I believe in their ability to do this work in a way that will benefit your country? [00:03:26]

**M3:** Now, from the first glimpse, let’s put it this way, before this organisation our transport system in the ministry, the servicing of vehicles, vehicle repairs, replacement of parts, the supply chain system taking drugs and medical supplies from the central medicine store to the periphery and at the health facility/clinic level, was a huge and new challenge.

After listening to colleagues in other countries, or other areas this organisation has worked in. We've seen how improved these areas are, especially with [inaudible 00:04:16] rate and service delivery in terms of moving drugs and medical supplies to the last man.

Since the fact is this is a challenge in our Ministry of Health in our country, we tend to collaborate with the government agency responsible for logistics -- that's the General Services Agency. Also, felt that it is an organization to try, and test, and see how that works. I think, since this health organisation have got on the ground, we saw improvement in terms of management of vehicles, in terms of sample transport during the Ebola Crisis. I think it should be of great innovation and I really [inaudible 00:05:08] innovations, and see that they improve data collection, they improve information sharing, they improve health management and information systems, transport of some commodities to the [inaudible00:05:23] [crosstalk]. The information they arrive at the village. [inaudible 00:05:26] the medical supplies. [00:05:33]

**Interviewer:** You mentioned, all ready, you’re seeing improvements in vehicle management and other things that this health organisation have been working on. In what way is the ministry of health tracking those improvements? What monitoring/evaluation are you doing of these, sort off, vehicle maintenance types of interventions? [00:06:09]

**M3:** We have our own unit for transport and logistics management, and the frequent break down of ambulances, the frequent break down of operational vehicles have been minimized since regular servicing of these vehicles and replaced of parts, the tires, and other important parts on the vehicles have started by this health organisation.

I have supervised, myself, as head of the Incident Management System [Changing] on transport of specimen -- people who are suspected of Ebola -- to get their blood samples to the lab for testing, has improved significantly. Where we're having back load now is to test...in normal laboratories, we have to test for utensil samples.

Transport is coordinated with each of the counties, though we still require improvement. By no way that, I have any peculiar interest in this health organization as a person, but I believe in a separate delivery, service delivered [beforehand] of this. And so not that I am championing this organisation, but I'm championing service delivery. [00:07:38]

**Interviewer:** Yeah, of course. I want to ask you about another organization. I know you work with a friend of mine, from another health organisation. Also, was a big part of the Ebola response, working with the ministry in your country. How did you come to learn about other organisations, because they're actually from your country yeah? [00:08:14]

**M3:** Yes. This other organisation has been working in our country, and has been working with the ministry health. What touches me most is when we designed a program, together with [REDACTED], working in [REDACTED]in very hard to reach remote villages to deliver high- impact, life-saving interventions

First of all, we wanted to know how much would it cost a person to deliver anti-malaria drugs, at the same time take care of diarrhea and other respiratory infections. We wanted to know, how do you reach the last man to transport commodities. We also wanted to know how much we had to pay a community health volunteer.

These are core indicators that separated another health organisation and his team. Seeing progress that we made in [REDACTED]District, really remote [REDACTED] and this now has been expanded to rural settings, other remote areas. I think even have this health organization, collaborating with another health organisation, will have synergistic approach because this health organization want to reach as remote villages as possible Where testing facilities, health centers and hospitals are not and people there need access to services. Yes, we work with this health organization very closely. [00:10:06]

**Interviewer:** I Know this health organisation, they used to be working in this location, sort of small. Then they worked with the ministry of health and ministry of health said, "Hey, Maybe your help could be used in [REDACTED]District." Again, what made the ministry say, "Yeah, we think this health organisation Team can really help the county health team in that area to see success?" Why did you pick, let’s say, their team? [00:10:55]

**M3:** Our goal in the ministry of health [and] development of the area in the health care delivery system is service delivery. We want to see our children live longer. We want to see immunization services -- children are fully immunized with the required doses of vaccines.

We want to see reduction in malaria deaths. We want to see reduction in diarrhea, pneumonia. These are the major causes of deaths in our country, and so organization though small, they are striving to reach a described [inaudible 00:11:39] is something that we subscribe too. Even at the level of the ministry of health, at the level of the president of our country we have exclusive confidence in what this one person is doing in a health organisation. The fact that the team is not in [REDACTED], sitting there, and [buildings] are accessible and they are willing to go to inaccessible areas, lack of assets, very remote. I think we all need to rally around them and give them the necessary support that is needed to move to other counties. We've seen [REDACTED]the area, we've gone on a few ourselves. Some part of our team in the ministry have travelled, flew into [REDACTED]District, hard to reach area. I have seen community health volunteers in action and providing high-impact, life-saving services. [00:12:49]

**Interviewer:** That's great. Are there other organizations? Obviously, you really have a good relationship with various health organisations, you've known them for some time, you respect the work they're doing with the ministry of health. Are there other organizations?

You don't have to name the exact organization but just to give an example of other partnerships where you think the ministry says, “No, we don't like the work you're doing. We don't really believe you're being helpful." And the ministry decides, with the pool fund or some other way, not to direct support towards that organization and whatever innovation that organization is suggesting. [00:13:40]

**M3:** Now, there reach a time I have not done other cost benefit analysis, or I have not done scrutiny of organization-by-organization to know their strength and weaknesses. I wouldn't be particular or specific to an organization, but I do know very well that there are challenges with services that we want to deliver that are not moving forward.

Example is our supply chain system has been very weak, managing drugs, medical supplies, and all of that. For example, there’re organizations that are working inside facilities that are [inaudible 00:14:27] here. So [inaudible 00:14:29] quality indicators that we’ve [set] for those facilities and deliver service in their organization on the [inaudible 00:14:38]. What we intend to [inaudible 00:14:40] some of it is to do a report/monitory of each organization.

We want to provide real service there, because some organizations get resources from our [pool fund]. Some organizations get resources through USAID; they have implemented projects on the field. So it would give us an opportunity that we can monitor some of these services that they're providing to the people of our country. And come out and say, “Yeah, we think your organization promised to do this but you are not achieving this.” But we have not done that yet, so I wouldn't be specific on an organization.

[background noise] [00:15:22]

**M3:** Are you there? [00:15:27]

**Interviewer:** Yeah, I'm there. Listen, I have a follow up on what you just said, actually. When you're considering evaluating the strengths and weaknesses of these different organizations, what exactly are you looking at? What things about an organization tell you its strong, or what things tell you its weak? [00:15:55]

**M3:** Come again. [00:15:57]

**Interviewer:** You're saying the Ministry of Health hasn't carried out say an evaluation of all the organizations, their strengths, their weakness. If the Ministry of Health was looking at a new organization or an organization that all ready exists in your country, and was going to evaluate that organization, was going to evaluate their work. What types of things would it look for to say...would it look at, I don't know, studies the organization has done? Would it go to visit the organization? Would it talk to other colleagues in the area? What types of things would it do to really get to know how this organization is performing? [00:16:54]

**M3:** We have service delivery indicators, we have administrative indicators, and we have operational indicators. Once there are few service delivery indicators, what we've done is that organizations are supporting health care facilities. Some organizations are supporting entire counties in primary health care. So we deliver or develop certain indicators in terms of service delivery. How many pregnant woman, based on the [inaudible 00:17:37] population of that clinic, that have received, for example, the IPP [00:17:43] preventive treatment for Malaria? How many people have delivered in the facility [inaudible 00:17:54]? How many pregnant women die? Why they don’t service this?

We also monitor stockpile information to know the number of days or weeks that a facility has been stocked out of drugs. These are the indicators that we have been looking at. Some of that work has been done. I’m sure that our team providing operation services for our [inaudible 00:18:22] program has been… [inaudible 00:18:23] program so some NGOs in the country have indicators. They're only few that have not made the indicators. They're some that are making [progress]. [day-to-day] I don't have the information now to say this one or that one is [inaudible 00:18:40].

What we will do in the future, if it is a new organization, is to hold you accountable to those indicators that we set for ourselves. For instance, in our agreement with this health organization our contractual agreement, they are saying that was in our first agreement...that building their office, service stations, service of vehicles, delivering drugs and supply, making sure that services [inaudible 00:19:13], sample transport, all of those are met [densely]. In one or two years we can evaluate product, they can cooperate. Those are the things that we're looking for. [00:19:31]

**Interviewer:** In what way do you collect the data for those indicators? [00:19:39]

**M3:** Some are health care facilities. Most will come from the health care facilities because we all ready have our staff management information system set up. So health care facilities [inaudible 00:19:53] sending their reports. We monitor the stockpiles; we monitor the operational goals. We monitor people who are working, all of those things. From the care, you have is 14 days. Yeah, that's how we monitor them. [00:20:09]

**Interviewer:** Great. You mentioned that this health organisation is helping you solve the challenge the Ministry has with managing its fleet of vehicles. That other health organisation is really helping the ministry with the challenge of reaching people in those far off areas that your country has. What other challenges remain for the Ministry? What are the big challenges, if you had to say right now, that the ministry is facing? [00:20:53]

**M3:** We have staff [acquisition]. A lot of that was because of people motivation, honor the salaries, people are working half of each period there is no open facilities. And so [inaudible 00:21:16] more reason for self-motivation has been a new challenge. We also realized the fact that the Ebola Crisis has shown how vulnerable and weak the system is, and how we need to build the systems at all levels in terms of, such [inaudible 00:21:38] all areas.

We also note that the number of resources at the ministry of health cannot provide adequate help. The Kenya Project has services for the people of our country and, so we [inaudible 00:21:50] Government of Our country Project for the support. We're also looking at [inaudible 00:21:58] with our health care facilities that don't have a department that handles special diseases. Yeah, these are critical areas. Supply chain again has turned up. It has stood up as one of the major challenging areas, that is storage, distribution, minimize of deaths. Yeah, those are the areas that I think for now we’re [lacking]. [00:22:30]

**Interviewer:** Yeah. Last question. I want to respect your time. If there was one thing that would help support the Ministry in being able to identify new groups like these health organisations, that would help the Ministry meet some of the challenges that you've just listed. What would help the ministry identify and scale those challenges better, faster? [00:23:08]

**M3:** I've been monitoring and tracking foreign [inaudible 00:23:14], so our [anarchy] unit needs a lot of help -- we get the information but not following up. Following up to know what is working and what is not working is very key, and so improving our monitoring capacity is also something that the supply side and not only the demand side for the [inaudible 00:23:47] is critical. [00:23:54]

**Interviewer:** What do you mean by demand side and supply side in this case? [00:23:58]

**M3:** The thing is, we brought in the periphery testing facility, and they need services. We ourselves, we get those services and give to them for their monitoring, the services that we deliver, and making sure they’re [ideas], they’re teaming their intended purpose or they’re [inaudible 00:24:22]. If the hospital or clinic is demanding drugs and medical supplies then we supply it. Or if the people of our country or services are broken to [inaudible 00:24:37], how do we pack in the staff and it's important. [00:24:45]

**Interviewer:** I thank you for your time. I know it's a busy week for you, but we appreciate your insights on this, and hope to be able to work to help that process. Is there any last thing you want to add just about finding these innovations? [00:25:15]

**M3:** Information is still there, and these independent interviewees or finding information is critical. So whatsoever information that you can share with me [inaudible 00:25:32], we can share can share [inaudible 00:25:34]. That will be great, thank you. [00:25:37]

**Interviewer:** Yeah, absolutely, we will definitely be sharing with you, with others who have kindly participated. So thank you very much. [00:25:45]

**M3:** You're welcome, bye-bye. [00:25:47]

**Interviewer:** Bye-bye. [00:25:47]
