# Supplementary material for: Supporting Better Evidence Generation and Use within Social Innovation in Health in Low- and Middle-Income Countries: A Qualitative Study
Source: PLoS One. 2017 Jan 26;12(1):e0170367. doi: 10.1371/journal.pone.0170367 (PMC5268497; doi:10.1371/journal.pone.0170367)
Supplement: S1 Dataset — (ZIP) [file pone.0170367.s002.zip › Data/Data - Interview transcripts/P1.doc]

| Interviewer |  | We have... so... just fitting... just switching out of the... um... brought your recording equipment, make sure we are all set up, which we are... and so... |
| --- | --- | --- |
| Interviewer | 0:00:19.02 | Our first question is what... can you basically tell us about what you do, just briefly, which I think we know, but just for the purposes of (0:00:33.8) |
| P1 | 0:00:34:4 | [REDACTED] |
| Interviewer | 0:03:08.4 | Thank you, that's great [REDACTED] |
| P1 | 0:03:25.8 | Thank you, thank you for saying that. You know, I heard somebody quoting a famous (0:03:33.0) where they come from, but you only care about what you experience, and actually few people have experienced it, and therefor care about it. Only few people know what to do about it, and because of our background we thought that's something... not only that we were able to do, but we must do because nobody else is thinking about it. One other thing i should say about our work is that we see ourselves as a partnership organization and we like to work [0:04:12.2] because they have the greatest need to reach all the population for healthcare. Some public health to emergency, so (0:04:26.2) |
| Interviewer | 0:04:33.4 | If you, so... I know you had a lot of success obviously, partnering with a number of people, deploying these transportation logistical systems, but at this point, what do you think, what's the biggest question that you wanna answer right now about the impact of the implementation of your program? |
| P1 | 0:04:58.6 | The impact of the implementation... well... We think that both the implementation and the (0:05:07.7) will be transformed if people realize the importance of this and this is dedicated to something that is really (0:05:24.0) and certainly, definitely prioritization. I think that if it's going to help recognize this... it’s a key piece, key enabler to everybody else doing their job more effectively and more cost effectively. I think we're being in a very different place and its interesting, its proof of that... proof of that because the ministry of health has prioritized this, they have told the big founders that (0:06:00.4) that it's a priority for them and therefore you are able to put it in place and then everything else moves much more smoothly; most people get immunization and a so lot of patients are (0:06:14.9). But it needs to stop being renegaded to something of little importance to global health. |
| Interviewer | 0:06:23.8 | And so, what are the ways you mentioned that you've, sort of, being successful in that country and getting this message across and being able to get them to take up that charge and give it the due priority that it deserves and what did you do there that enabled you to effectively communicate that message and what do you think in places where that hasn’t been distributed strongly and what did you tried there and what could you compare, contrast a little bit. |
| P1 | 0:06:57.4 | Yeah, I can. There are three key factors in that. First of all, the WR for World Health Organization based in ([REDACTED]) is from a particular country. She saw the work of (0:07:16.5) and what it was doing in terms of proper (0:07:20.2) diagnosis and also (0:07:23.1) and other issues in a very difficult way. And she supported it right from the beginning from WHO and when she retired and went back to that country, she said to the minister of Health: "You need (0:07:37.5) for help here, because we got a broken health system and the fundamental (0:07:42.4). Second of all, the Ministry were* having trouble with [REDACTED] because [REDACTED]are sending in drugs that they are not reaching the people who need them and felt that they needed their help to (0:08:04.3). And then, thirdly, they got Ebola and they realized that if you don’t (0:08:13.6) rural areas that an outbreak can quickly turn into an epidemic and epidemic can turn into a pandemic and so the world took that very seriously and said: "Ok, we have to make sure that this gets done". But what's great about that country is that they have a very... We have an experience in another country where the whole health system and the people affecting it maybe come out as very tormented. While that country has very... I’m not saying it's perfect, but certainly everybody is going in the same direction. Than they have huge number of vehicles donated to them because they said: "We've got to use these to build our health system out of export ideas and we can’t have these vehicles just breaking down after eight months”; and they are waiting, they don’t take this route to the next level. So, it was really several, you know (0:09:19.0) one of the things we've learned from that... Oh, and all of a sudden they went to see the national model, releasing model (0:09:34.2) model that we do with the [REDACTED] government and they went to see how that operated (0:09:41.1)a matter of really prototyping your rural communities and somebody (0:09:49.3). Some people think get confused about the [REDACTED] whether you spend most of your money on the easiest to be (0:09:59.6) population and those who does are the ones who are most likely to want to engage with us. And also some people really just, I mean... |
| P1 |  | (0:00:05.7) So you need to shift it in the prioritization. But as (0:00:12.4) and contrast, where we struggle is where either the government’s ministry of health is (0:00:23.0) independent or if they are unwilling to go to the big funders like [REDACTED] if they are unwilling to go to them or [REDACTED] even, for (0:00:39.8) so it's a complicated little cocktail of things that can make the magic work, or simply bring it to a grinding halt, which we finally found there, because (0:00:57.2) but this really worked; made a difference to elementary health in terms of (0:01:08.6) makes a difference to health workers because they reach the people whom they serve and the huge coverage means that people get healthcare that was never even known they even have health worker. So it is consummation, but takes people (0:01:29.1) macro level to have a change in their thinking |
| Interviewer | 0:01:40.6 | Yes, definitely good, definitely complex. Let's unpack a little bit about your staying there. So, you mentioned this woman from the the ministry of health had seen your programs in action and actually seen them, so that was what is was, like a quite transformative experience, in terms of them becoming an ally and then becoming an advocate for transportation investments. Obviously... |
| P1 | 0:02:09.3 | I suppose I visualize at something that would change things and being able to get people (0:02:19.6) in the middle of New York. Really, it’s impossible, in the middle of various countries, let's say: this is how you do it, and it's not... we're not being (0:02:40.3) in saying it and (0:02:46.7) we're not doing brain surgery, it really is... it doesn’t need systems when (0:02:57.3) you've got to make sure taht you've got (0:03:01.5) and all the vehicles, so if you manage it, it's complicated and actually costs in intellectual... confused, in a way to put this together actually what you put together is inconsistent... it can be managed in monitors and done well and the thing your people could see is has (0:03:29.0) it will make a difference. But I'm going to ask people like doctor ([REDACTED]) if I can get them to be ambassadors, and talk to the ministry of health to give us the budget which we want (0:03:44.0) But, nevertheless, the thing is... is a goal for me. |
| Interviewer | 0:03:49.5 | One of the things you said was great, like, making these people who have seen it to be ambassadors for us; what are the other ways you are thinking about, jeez, how do we make this more provocative for people to you; bring more people out to your sites, do you take videos, do you... what are some of this strategies you use to communicate the success a little more to people who can actually make decisions? |
| P1 | 0:04:18.5 | I think we have a (0:04:19.5)... you know, what we tend to do, we... I don't mean we get confused cause without (0:04:29.4) our traditions. But when it comes to photos of things, do you think that's (0:04:36.7) individual donors. And when it comes to the big macro... binational and multinational (0:04:45.9) you tend to think in terms of rapport; i think like we're doing the wrong thing, and it helps (0:04:52.3) about it because I think there is a way of engaging people that are at a very high level, but because they are human beings after all, of course they are, with the numbers, with the facts, with external verification, but also with pictures and... |
| P1 |  | We start also with pictures and (0:00:03.7) and so on. Because I think we can keep things to the dry, for the very big thunderess* 0:00:10.1 And then we also (0:00:11.7) the people with responsibility to do this things (0:00:18.5) and so on. I think we're probably presenting to them, in their own way. I think we search 0:00:25.4 8 for many of us. A little bit to--I also had conversations with people at the UN. How does big… of that size work with small, in terms with the size that we are. But never the less, what we're coming up with, is the systems changes, it’s not the kindda Sunday approach. So I do think that there are things that they could--people at the macro level, could really be engaged and listened to, in sense of innovation what is about system change and about something that could be scaled and replicated. |
| Interviewer | 0:01:07.9 | Right the other thing that I was gonna’ ask you about was, you mentioned, so there's making it (0:01:16.9) for people being the mere images, videos, and there was the sorta’ that you’ve said, the numbers and the facts. Are they evaluations of Stanford? What did the evaluation looked like? What type of study designed to use? What was the goal? How did that take place? |
| P1 | 0:01:37.5 | The Stanford study goal was to look at the various layers of the health system. From the (0:01:50.9) health to the people at hospitals and laboratories. to look at the health work file by file; to look at the community and what it means to them. And in a way--it comes out in a way that we've always said that you can reach five times more people. Cause the health workers condone more often. As you can be predictable, cause you know you're being profiled (0:02:20.9) by a (0:02:22.7) you could be reliable. And it actually turns out that within real community, they value predictability in all the things, cause they know this is the health work that they're coming on Thursday. We will be there on Thursday, so they won't go out in the field; they won’t do--they won’t have to walk all the way to the clinic 0:02:44.2 And so knowing that that's gonna happen too many times they walk to the clinic (0:02:53.4) because the vehicle’s broken down or because (0:02:57.5) vehicle would get there [noise] |
| Interviewer | 0:03:16.8 | Sorry can you give me 5 minutes. [noise] |
| P1 | 0:04:59.0 | Strategy about how to do that. You record one end where you always do something that works, rather than the divination that draws people's attention to the issue or the solution. |
| Interviewer | 0:05:22.5 | Okay so then I guess there’s two questions to--so then do you face any challenges? And were there challenges in deducting the evaluation? And then what sort of challenges--you sort of hinted at challenges at the disseminations, what did those looked like and how do you think those can be overcome? So I'm sorry double barrel question? |
| P1 | 0:05:46.6 | Huh the evaluation with the responses certainly found some challenges because--if you go from the (0:05:58.9) and so I think that the (0:06:07.8) of the initial stature took longer to set up than they thought it would. But once it was got going, I think that they were fine. I think that it was difficult for everybody because the [REDACTED]foundation and all of us were aware that if you claim health care outcome you're going to be challenged. The moment you say that you've improved life expendency or stop, many people from dying or whatever it is, the academia is going to challenge you, and then you (0:06:52.3) We believe that it was important to show that this was about health system. What does this do to the health system, in terms of enabling and giving access to community to health care. And so what we were doing was measuring the health system and the impact of our work on it, rather than health outcome. But as they've pointed out, there are a lot of studies, there are 100 years to show that people had access to health care. Their health care (0:07:31.0) so they probably didn’t need to do that, but there was quite a lot of struggling in the initial stages without the--what study should... |
| P1 |  | Examples and taking the to* (0:00:01.6) that quickly, so we're doing that nationally in three counts in various countries. Ad, oh, sorry, and (0:00:18.0) we do it nationally. We collect all the data about how much each intervention cost and what is the cost of collecting one file, for example, that we know that we can look in today, how we get cost down, how we're doing journeys as well(0:00:43.0) are they efficient in terms of cost, so we are looking at all sorts of things of that kind. |
| Interviewer | 0:00:55.8 | So do you mostly use then all that data just in terms of improving your own operations or is that also part of stuff that you would (0:01:04.3) |
| P1 | 0:01:05.8 | We do that mainly, i mean we do of course to donors and to the individual donors about those thing, that's much more.... i don't mean, wait, what sounds rude to the donors, but you know, it's much more story, that it is an analysis. But to be honest with you, we have so much data, we collect so much data and we have a real struggle with... because it's costly to get them what they want, to analyze it and that we produce it. (0:01:40.9). You know, we just don’t have enough (0:01:44.6) to do that, i mean we just don’t. So we tend to do what we thought (0:01:51.5) but it's going to be within my limit, certainly during the next year, now that we got the proof from this country about how you can implement this (0:02:07.0) and what difference you could make so quickly and what the actual cost and what the outcome is. I've got to (0:02:18.6) on that over the next year. To get this be viewed as a higher priority than it has been, as it is… |
| Interviewer | 0:02:33.3 | So, in a perfect world, what do you think will help you kick start that project? |
| P1 | 0:02:45.1 | Well I think (our organisation) actually need more expertise an manpower on the (0:02:58.9) So, we... It's a very funny thing, and I'm sure all the organizations you are talking to, all your donors and your partners, all want lot of reporting and a lot of (0:03:25.0) stuff, but nobody really wants to pay for it. So you know... cause they want to pay for something else. But it think in an ideal world I would want to see a much more, sort of, academic (0:03:48.9) in a way not to just academic (0:03:53.5) sake, but to really figure out how do you prove this, how do you show the global health community that this is very significant, it's you know... of course there are lot of other significant things, you know, lots of others, but this is one thing, you know. You look at all these things like the new development goals and there might be one sentence, in the appendix, on transport and this (0:04:27.5) is very hard to get the point over. So in the ideal world, I'll have much more expertise and focus on finding a way to, in the languages of the big guys, like [REDACTED]and so on, really would find appealing, just can prove the point. That would be my ideal world. |
| Interviewer | 0:04:59.9 | Great, wonderful. Last question for you, I said I wanna respect your time we said we'll chat for thirty minutes and it's been just about thirty minutes so we just have one last little question for your leave and it's kind of a downer but also it's important. Do writers think about ways in which the program can actually be harmful and do they do any measurements for that* effects. |
| P1 | 0:05:29.8 | We do. We think about environmental issues actually, but in fact because until our organisation is there, people do things like tipping oil into water (0:05:44.9) so we have practices in terms of vehicles, the proper ones. We're training people to make sure that dispose of lubricants in a proper and safe way, so actually there is a potential with vehicles. But because our organisation keep people not to do that, so I think that any program I suppose has the potential to do harm if you're not being guided by the ministry of health. That's why we like to work with the ministry of health, because we don't do what we want to do with health, we want to just enable them to do what they want with health. So I really think that if you work, you know, outside the government, same work, you certainly could do harm. I guess that if you were competing for budget, people might argue that, say example, i don't know, more health workers are more valuable than budgeting for vehicles, but actually you are wasting your time and highly trained health workers if they have to walk everywhere and they get so disfundant*, they can’t do their jobs anyway, so I don't think, I mean, certainly we're not doing any harming, in terms of the environment, we make sure our vehicles are very well calibrated, so they don’t... they cause the lowest possible emission. Then, you know, if you think of the developed world, Los Angeles, what's that pumping out into the world and what one small motorbike with a health worker, you know, in the middle of nowhere is going to do, its ridiculous to even consider. And so, I might be flattering ourselves*, but i don't think so. |
| Interviewer | 0:08:03.9 | Okay |
| P1 | 0:08:05.9 | We're a lot of people. We employ almost [REDACTED]people in [REDACTED]countries in [REDACTED], and well, by the time we finish taking people out in this country,, we'll be way over [REDACTED]. And by the way, we also do gender balance, so we never train more men than women if we can help it. So (0:08:28.9) into business with men and women and being a mechanic is just as well done by a woman that it is my a man, [REDACTED] we need to support the gender issues that are present in the developed world, in terms of Africa and healthcare. |
| Interviewer | 0:08:56.9 | Great. Oh, brilliant. Cool. Excellent. Well ok, I'll let you go cause i wanna respect your time, but thank you so much for talking to us. |
| P1 | 0:09:06.4 | It's OK. I hope I didn't go on too long, but as you can tel, i pretty excited about it all, and I'm just grateful to you for asking us, because, you know, moaning about people not prioritizing it, but you are, so I'm very grateful to that. And I'm... It's great to be part of this work that you are doing. |
| Interviewer | 0:09:32.3 | Cheers, thank you form... there's no record from people who (0:09:36.0) so, thank you and I look forward to talking again soon. |
| P1 | 0:09:42.6 | Very nice to hear you; speak to you soon |
| Interviewer | 0:09:44.5 | Speak to you soon |
| P1 | 0:09:44.5 | Speak to you soon. Bye Bye |
| Interviewer | 0:09:45.8 | Bye Bye |
