# Supplementary material for: Supporting Better Evidence Generation and Use within Social Innovation in Health in Low- and Middle-Income Countries: A Qualitative Study
Source: PLoS One. 2017 Jan 26;12(1):e0170367. doi: 10.1371/journal.pone.0170367 (PMC5268497; doi:10.1371/journal.pone.0170367)
Supplement: S1 Dataset — (ZIP) [file pone.0170367.s002.zip › Data/Data - Interview transcripts/P2.doc]

| P2 | 0:00:00.0 | So I work for an organization as a [REDACTED], and (0:00:13.5) has been working after the past decade with the our ministry of Health to design and build (0:00:23.3) universal healthcare systems. So, what we focus on [REDACTED] |
| --- | --- | --- |
| Interviewer 1 | 0:01:35.5 | Can you tell us a little bit more about how you particularly got involved with (0:01:40.3) |
| P2 | 0:01:43.9 | [REDACTED] I got frustrated with the experience of doing research and doing studies that then got into publications and set on (0:02:06.4) and saw very little application, if anything. So around that time, I moved to this country do some research together with (0:02:20.6) and during that time, while we were working and doing research in this country, our friends and neighbors, because we were living in the communities where serving. Our friends and neighbors were getting sick and they’re unable to access the care they needed and we had a number of very formative experiences watching babies die in front of us. Attending the funerals* young children, young mothers every week and accompanied patients as they struggle and fail to access care and (0:03:03.0) And those experiences brought us on to this facts. |
|  |  | **0:03:08.1 [noise] 0:03:24.4** |
| Interviewer 1 | 0:03:26.0 | Hey [REDACTED], you seem to be cutting out a little bit, are you still there? |
| Interviewer 2 | 0:03:30.3 | Yeah, I'm still here. |
| Interviewer 1 | 0:03:31.5 | Ah, here we go. |
| Interviewer 2 | 0:03:34.8 | That could be quite tossy* P2, and (0:03:38.0) a lot of people working in the research are not seeing much of it 0:03:46.7 [noise] I guess falling onto the (0:03:58.2). To you, how does... we show--define success? How do you find success and how do you measure that success? |
| P2 | 0:04:12.8 | Yeah, absolutely. So, our work focuses are on providing universal access to care and stopping preventable deaths and (0:04:25.2) communities. Toward that end, we build a model of proactive healthcare that combines active (0:04:33.2) action, doorstep care and (0:04:37.6) to reinforce an extend* to government primary care centers. So that combination of proactive community is managed by communal workers and (0:04:52.2) the referral to capacitated primary care sending that we work with according to the government (0:04:59.8) The ways that we measure success or failure, you know... I guess you asked about success, but for us is... If we're actually taking time to measure, then.. .we measure things where we have a question, right? So we need to know the answer to something. I think there is... Sometimes that kind of conversation is that we are doing things to prove a model etc. That presupposes that what we're doing already works and that we figured everything out and sometimes for (0:05:46.8) opportunities to really take measurement is our opportunity for learning and for countability*, because a lot of global health interventions fail and a lot of great ideas don't end up working out. So, for us, we care a lot about measurement, really, it was builded* in the DNA of our organization that everything that we do, that we can try and measure. We build into every implementation a measurement of its success or failure, where success and failure (0:06:29.3) in some situations. Primary indicators that we look at are, on the trusted side, a number of visits at the (0:06:41.4) clinics, so are we providing care to more people, reaching toward universal health coverage and universal access. How fast we are reaching patients, because we are very centered on this idea that earlier access to care can be a catalytic outlet for reducing (0:07:04.8) child mortality and so we look at time from symptom onset to treatment onset and percentage of kids that are at age five that are reached with effective antimalarial treatment, for example, within 24-48 and sometimes two hours of symptom onset. And most importantly, we look at mortality. And we look at mortality not just at the level of the health system, but at a population level. Our measurement system is, i would say, are two tiered. The first tier is internal to the health system. In that we are measuring how much care we provide and the quality of that care. And so, we have built system that enable us to analyze how much care (0:08:08.4) provide, how fast they reach their patients and what percentage of these patients get care without any clinical errors. So that's an example of the data we collect from patients that we do reach and say looking at the data from last month we can say: "OK, in the month of June our (CH) - 75 (CHW) are carried out in site and cared 382 children that are sick. And those 382 children, of them 75% were reached within 24 hours of symptom onset; 88% within 48 hours of symptom onset and 90% (0:09:02.8) hours of symptom onset. And those are some examples of measures that we have for the population that we reach. We have another layer though, because, for us that's really not enough because we care even more about the patients we fail to reach that about the patients we do reach because those patients are often the most vulnerable and that's the area we need to improve on. I'm sorry, just one second. |
|  |  | **0:09:39.8 [noise] 0:11:44.6** |
| P2 | 0:11:45.8 | 8 They were like: "We need to talk to you right not" Sorry, I'm back. Yeah, we do population level measurement as well. WE look at access to care, percentage of kids getting effective malaria treatment within 24 and 48 hours of symptom onset at a population level and count mortality rates. And we use... Currently, today what we've been doing is we've been using a population (0:12:22.8) cluster (0:12:23.5) methodology to get a representative sample of the populations that we serve and that we're doing a (0:12:30.8), rather an interrupted time series study, surveying representative sample of the populations we serve every year. That is most of what we have done today, today we published two studies, one on qualitative and one on quantitive* and coming forward, we have three more studies that are in the work, one of which is (0:12:57.5) original interpreted time series (0:13:00.2) people get six years of data (0:13:04.4) and one given population under five mortality rates and then we have two randomized control trials being planned. One very small and one very large. |
| Interviewer 1 | 0:13:22.5 | So what are (0:13:23.5) |
| Interviewer 2 | 0:13:26.4 | So are you ever found... You guys, I think, are unique among a lot of the social (0:13:35.6) health that we are speaking to and in that you employ quite rigorous trial designs, randomized control trials, randomized designs like interpreted time series. What has moved you to make the investment to undertake those types of trials and how is the reception of those... that of evidence been among those you seek to influence? |
| P2 | 0:14:05.3 | For us, the most important reason to do the research is the patients. We see... I've seen research be a, you know, relatively useless in a number of context, my own research. That just talking about research that I have done and so, research doesn't necessarily have to be helpful, a lot of (0:14:32.9) to this work is seeing that, but used correctly, we believe that research can be an implemented justice. And research is a tool of justice. Because we're here being partners in a company (0:15:00.3) to the communities that we serve. But how are we holding ourselves accountable to our patients, to whether or not, ideas that we have are helping anyone or hurting people. If we don't measure, we don't know. And we want to purchase (0:15:19.5) knowing how much damage is... can be caused by good intentions and knowing the best way for us to figure out how to serve, how to handle the greatest impact for a patient and move forward because of justice is to manage rigorously. I think about global health work in terms of (0:15:48.2) big frameworks, there's a benevolent framework and there is a justice framework. And the (0:15:55.8) framework thinks about OK, it’s kind of OK that the world is the way it is and it's a good thing for people who have more to give and do good deeds for those who live in poverty, sort of a charity framework. There is a different kind of framework that says that the world is profoundly broken and that we have a collective responsibility to address injustices that surround us. If we subscribe to the second framework than we have amended that it actually makes change. And if we're not measuring whether we are making change or not, who is holding us accountable and how? So that's, sort of, how we see it. First and foremost, there's an addition to that, some strategic and tactical importance to doing research for our organization, sort of, scale up and translating what works into a bigger change, change that... at larger scale. Cause we care very deeply about (0:17:12.2) were working on, on the global level as well, in addition to the patient's research priority. And so... |
| Interviewer 1 | 0:17:21.0 | Sorry. |
| P2 | 0:17:25.1 | So we... For example, we're just sitting down with the minister of health on Monday and his... Really, the research that we did together with the minister of health that kindda opened the doors to that partnership and that created opportunities for joint innovation. So, if we had gone into these areas with the Ministry and said: "Oh we actually want to do something that is completely different than your current national policy"; The Ministry would have been... They would have blocked it. They would have felt threatened by it, they would considered* it dangerous. And perhaps with good reason. It's important for them that they are protecting their population and making sure people adhere national policies. By innovating in the context of joint operational research with them we get to frame things in terms of: "Ok, the Ministry has goals, we would like to be helpful by doing operational research with you, trying some new things and testing them up. With no obligation that these things, no presupposition that these things are going to work. But if they do work, than that will provide useful information for your policy planning, going forward and thinking about what the next Ministry national policy is, the next phase of the Ministry's health system should look like." So, for example, when we started our idea for CHW is dangerous in this country*, there is quite a bit of resistance. And a year ago, within a few months after we published the study with them, the Ministry approaches and says: "We want to set up CHW's on every remote village in the country. Will you help us rate the plan?" So, that relationship has grown out of research and research has also enabled us to remain a humble and sort of engaged partner with them (0:19:46.5) providing a service to them, being a sort of in evident broker. That... Serving them by feeding evidence into their policy (0:19:59.9) and conducting research together with them. Sorry, giving you rather long-witted answers. |
| Interviewer 1 | 0:20:08.4 | That sounds like you worked a lot with the Ministry of Health, it’s really.... Particularly (0:20:14.6) because of research. Are there any other stakeholders that you aim to influence? I guess particularly looking at your research that you are doing. |
| P2 | 0:20:29.7 | Yeah, I'm... And that does influence the study designs that we do. So, the minister, we showed him on Monday our [REDACTED].. The minister cares that is is published, he cares about the change that we documented and under five mortality, he cares that we are measuring together with his team indicators that are his priority goals. And she doesn’t really care whether it was an interrupt, the time series study or whether it was an random ask control trial even though methodologically our study--we can make, cause a lot of confusion. There are a number of limitations to it. From the minister’s perspective, he sees a [REDACTED] drop in child death rates and he says, "Okay I want this everywhere in my country. How do we think about doing that?" But we also know that ministries at home don’t make their decisions in a vacuum. In order to get there, he is going to--the minister and the law in general is going to need other partners from the international community. And those partners, what we've found care about, what the public in the land said, or in another general, was it in our seats here or not. And so if we wanted to bring in an build momentum which we do was to buy laterals and the multi-laterals; the major institutions that can provide catalytic founding and bring new policies to the minister of health to scale. Then at the same time, we need to be doing research that they're going to consider relevant and rigorous as well. And that can help inform a global best practice as possible. So that’s part of the reason of why we're planning of doing a number of carefully--hopefully very carefully designed RCTs that will generate a lot of evidence that could help inform global based practice, and globalize some of these international partners. |
| Interviewer 1 | 0:22:57.0 | Has your research ever been designed to target people outside of, I guess the government space? So, private enterprise, academia or other audiences? |
| P2 | 0:23:10.7 | Private enterprises probably not. I don’t think we're targeting academia. I think that--in the sense that the editors of the peer-reviewed journals and their colleagues. We're embedded in academia, we are--our work is now in collaboration with three different universities. So in a sense we're embedded in the academia scene. But--the editors of the journals that we submit to are academics. So I guess in those senses, yes. In order--engaging that, those communities, we are aware of their expectations, etc. and they're relevant to us. They are also relevant because its science and it’s true that, without a comparison group or a control group, there are limitations and they cause a lot attributions that they can make. And well-designed research helps you answer the question better. So I guess in that sense, we try to learn as much as possible from our academic collaborators and we try to put things together that are going to bridge between what is going to be relevant and important for our patients and if they’re not going to get in the way the care they need and what is going to be rigorous in terms of us to learn of how to better serve them. Does that make sense? Did I answer your question? |
| Interviewer 1 | 0:25:12.0 | Yeah. It's great. |
| Interviewer 2 | 0:25:13.6 | I ask… [REDACTED] alluded to before. You guys seem to be in prime position to have the ability to undertake such rigorous research. Just wondering, what kind of resources does [REDACTED] use to conduct an evaluation of research that you do? So we're talking about like what staff members do you employ? Number? What kind of other resources do you use, such as with data collection? |
| P2 | 0:25:47.6 | You know, we've been bout-strapping most of our research to the--within number of members of our team. Wear multiple hats. And with a number of our research collaborators that they are doing the work program. And spending somewhere between 10.000-20.000$ a year on the research itself. That's a lot of volunteer hours. That's changing, we started serving about a [REDACTED] people --so the stakes are higher, our budget is larger, and the skill of the research that were doing is larger. So we are just finalizing the retreatment right now, that the dedicated director for the division of research for us, [REDACTED] He's [REDACTED] professor and the cost of hiring him was actually quite modest. And we also are--came % FTIs for a couple of our collaborators at [REDACTED]. But even all of that, still represents a small percent of our budget. |
| Interviewer 1 | 0:27:30.7 | So following that. What are some of the particular challenges that you face in conducting the evaluation research? |
| P2 | 0:27:42.5 | I think for a while our team has been working on a body of research--how do I express this? So there's a traditional pathway in academia; someone gets their PHD and does a thesis, or there's a fellowship after their residency and a place for a K Award. And there’s a research under a principal investigator. And that research is usually the principal investigator's research and then eventually they develop their own body of research, and become head of their own lab or their own shop; basically applying for grand some. Moving forward the body of research is their focus. We have all of these research and we've wanted to do and needed to do, and we, in order to do it well, we need to engage some of this amazing--need this folks with these amazing skills and the structure of academia is such as that they have their own research agenda already. And we don’t have the time for one of to sorta stop--one of the members of our team to stop what we’re doing to start a fellowship and get a K Award, and wait 6 years, or 10 years, or 15 years to get--to start doing the scale of research that we're planning on doing. So we've kindda had to game the system in order to do this stuff. We've had to be fortunate enough to find academics who* are willing to give a portion of their time, either as a donation or as a portion of their FTE. In addition to their existing body of research we've had to mobilize unrestricted founding and move it toward research since were not on traditional NIH pathway. I think those are some of the challenges are related to the fact that the system of academia and academic research isnt set up to match the most qualified investigators with the biggest and most important trials out there who are implementing organizations that are trying to take on this problem and could benefit from working with them. |
| Interviewer 1 | 0:30:33.7 | Okay. So in a world where you did have access to all the research personnel that you require and also the funding that you require, what are the challenges with the (0:30:47.0) |
| P2 | 0:30:49.8 | 8 There are a lot of operational challenges in terms of designing research that's appropriately rigorous but operationally relevant and figuring out how to make sure that were integrating the research that it doesn’t slow us down. So those are pieces that we think about a lot. And it did into the varying degree of our design. So it’s challenging to work in this places of extreme poverty and there are just challenges to working in these areas , and there are challenges where if you do a clinical trial in an area like in the US where people are getting standard of care already, then it creates a very different situation then when you’re working in areas where there are a tons of evidence based interventions that aren’t implemented. So that’s another challenge, but I think the ones I’ve said in the beginning are the biggest challenges. And once that we’ve been fortunate with and its been possible for us to overcome but non-trivial issues. |
| Interviewer 1 | 0:33:01.6 | Huh. The very big issues that I think it was the--so I guess you kindda touch on these already, but in perfect world, what do you think would help you do your work better? |
| P2 | 0:33:22.9 | I think things that will help us do our work better, would be more mechanisms to connect both by graduate students and principal investigators with NGOs that want to do rigorous relevant research. And sort of more mechanisms for those kinds of partnerships to be found and developed. I’d say other founding mechanisms that focus on health systems related research and that are more flexible than the NH pathway. I think those are the two biggest things and I would say also more pathways for evidence to get plight. The last thing I'll mention is that a huge amount of intervention that happen in the global space that we see are skills and evidence presumed. And from our perspective that represents a great injustice and a great beast for our patients. And we would really like to see the whole global sector rally around rigorous measurement and holding ourselves accountable together, and holding each other accountable. Those are the things I can think of |
| Interviewer 1 | 0:35:30.5 | Yeah I can’t agree with you more, particularly on the last point that there’s a lot of things that happen in the global health space and giggle where the evidence isn’t quite there yet. Just drawing back into what you do with milestone, is the final question, have you thought about ways in which your program could gently be harmful? And you've touched on these quite nicely towards the beginning about, we can’t measure our successes but we have to measure the failures as well. Is any of your research targeted towards looking at how thing could be potentially harmful? |
| P2 | 0:36:17.4 | Any of our research are* targeted on whether something can be potentially harmful. Much of our research looks out whether what we’re doing is working, and if it doesn’t work then in a sense, that’s harmful, because--yeah I guess I’m not sure I understand the question. If were measuring things carefully then were as interested when an intervention trial fails, or causes harm. That is as important as--if not more important does as when something succeeds. And so we learn a bunch of things about how to make the work we do better. For example, when we first ruled out our CHW intervention, we got the ratio CHW population way off. And our CHWs were working very long overtime hours, and eventually they burned out on that. In this study that we did we saw that their performance drop off. And so that was a big thing for us to find, and based on that we tripled--we dramatically changed our CHW numbers and our ratio on CHW population. |
| Interviewer 2 | 0:38:18.9 | Exactly. Well that actually brings us to the end of the interview. [REDACTED]did you had any more (0:38:28.0) |
| Interviewer 1 | 0:38:28.7 | No I just want to thank you P2 for articulating these ideas and you said you rambled but you actually--I think you were quite coding, especially making that explicit link between research and justice. And it was really just a pleasure to listen and learn from you. And were so happy that you decided to join with us on an interview, and we definitely wanna--we know you’re a man in high demand so we wanna respect your time and let you get back to your work or your evening, but thanks again. |
| P2 | 0:39:07.2 | Respondent: First my pleasure. Do you guys have access to… would it be* helpful for you to have access or you already have access to the couple of all these studies that we’ve done so far? Would that be helpful? |
| Interviewer 1 | 0:39:18.8 | That would be very, very helpful. Yeah we’ve got to have a copy of the research that you’ve done so far. That would be very great. |
| P2 | 0:39:28.8 | Okay. It’s very modest stuff. If its helpful happy to share it. I can even just send you over this Skype call right now so I don’t forget. I’m sending you two studies. One is [REDACTED] and the other is [REDACTED] [REDACTED]study really help us understand what are the road blocks on the path to care that our patients face, and help us inform our health systems designs. I don’t know if that’s working. |
| Interviewer 1 | 0:40:45.5 | Yeah it seems to be kindda coming through but definitely slowly. If you want shoot it on over here. If that’s easier, I think it would load better. |
| P2 | 0:41:00.1 | Okay. |
| P2 | 0:41:31.5 | Right. I just emailed them to you, so you should gotten* those. Thank you both for taking the time to talk and I really want to hear your feedback on how did things go and what you guys learn and yeah want to learn from both of you. |
| Interviewer 1 | 0:41:56.4 | Yeah definitely I think were--that's one thing that [REDACTED] does well, make sure things get (seminated) among participants etc. and that they are intervertive* process that we can keep feeding back what we learn back to you guys, such that its actually useful and that we can better support the health of this generation and that for practitioners but also policy makers and funders can take it a little more seriously. So cheers, thanks. we'll all look for the (0:42:29.8) that you’re still sending. Bummer go internet but if we don’t get it we'll come find you for sure. Thanks P2. |
| P2 | 0:42:44.1 | Alright thank you both. Great talking you.* Talk to you soon. |
