# Supplementary material for: Supporting Better Evidence Generation and Use within Social Innovation in Health in Low- and Middle-Income Countries: A Qualitative Study
Source: PLoS One. 2017 Jan 26;12(1):e0170367. doi: 10.1371/journal.pone.0170367 (PMC5268497; doi:10.1371/journal.pone.0170367)
Supplement: S1 Dataset — (ZIP) [file pone.0170367.s002.zip › Data/Data - Interview transcripts/P3.doc]

| Interviewer | 0:00:03.3 | P3, can you tell me a little about what you do, just in your own words? |
| --- | --- | --- |
| P3. | 0:00:11.1 | Yeah, [REDACTED] works to imbed an environmental health sustainability framework into the healthcare sector [REDACTED] |
| Interviewer | 0:00:36.9 | And how did you particularly get involved with your organisation? |
| P3. | 0:00:40.5 | [REDACTED] |
| Interviewer | 0:00:43.3 | Yeah. And how did you get interested in the idea and how did all come out? |
| P3. | 0:00:49.0 | I mean, the core... the core thought was... I've been engaged in issues around environmental contamination and its impact on communities for probably [REDACTED], since the [REDACTED] disaster and focused in particular on the chemical industry and realized that the only way that we're gonna change the economy away from the reliance on toxic chemicals, on fossil fuels, on industrial agriculture is to get some downstream industries to essentially reject their addiction to these practices and these technologies. In another words, a sort of detox program for the economy. And so, in that context, healthcare made enormous sense because of framework; ethical mandate to do no harm - Hippocratic oaths. And they couldn't be saying: "Yes, yes, we are polluting our neighbors because it's in service of health" It's a sector that can't address that contradiction. Second, until it way of actually organizing the health sector in opposition to the fossil fuel and chemical industry, if you wanna know the truth of it. And then, but at the same time we were concerned that the only way we are going to really address this rising epidemic of cancer and obesity and diabetes and asthma and other chronic diseases of XXI century was actually to address the environmental health conditions in which people live, and who better to lead that effort in the health sector. And then the third thing is just (0:02:46.7) which was at the time we were thinking of doing it in the [REDACTED] was reporting that medical waste incinerators were the largest source of dioxide emissions in the country and responsible for at least 10% of all mercury emissions*. So these were (0:03:08.4) chemicals for a lot of our work and so the fact that the healthcare sector was such an egregious polluter, it just created this really great opportunity to address it and engage with them. |
| Interviewer | 0:03:23.4 | Yeah, wow! I was... It's a... That's quite surprising that the medical community itself applies such a big part in contributing to the environmental harms. |
| P3. | 0:03:34.8 | You know, right? I thought so too. And i the [REDACTED] it gets worse because in the [REDACTED] healthcare itself is 18% of the economy. [REDACTED]. So it's huge sector, huge. |
| Interviewer | 0:03:56.4 | Wow, OK. So, wow. It sounds incredible the work that your organisation does, and it sounds like it actuates a lot of the environmental issues that (0:04:09.8) with the healthcare system. But is there a big question that you want to answer about the impact of implementation of the program that you are on? |
| P3. | 0:04:21.4 | Big question... Yeah, I think that the biggest thing is how can we incorporate environmental health issues into you know... as a core component of addressing global health, addressing (non-clinical) diseases, addressing the major health priorities of the XXI century, because what we now know is that the diseases of poverty, the diseases of the environment are actually killing more people, impacting more people that infectious diseases which we've spent (0:05:10.4) century, and in real, serious way in the last 30-40 years addressing. So, just as one fact: indoor and outdoor air pollution kills twice as many people as AIDS, TB and malaria combined. And so when you think about the enormous infrastructure that's been created through the [REDACTED] and how much money the [REDACTED] foundation puts into malaria and these things. There is almost nothing, nothing put into addressing air pollution from the health sector. So, it is a totally new reality related to our addiction to fossil fuels that is creating a mega driver for global health. And it's barely on the radar screen of the healthcare sector, I mean, WHO is talking about it, now the Pope is talking about it, we've got (0:06:19.6) to talk about in in the [REDACTED] and obviously the [REDACTED] healthcare sector is waking up, but we got a long way to go to really get environmental issues essential to address in population health. That's the core fact. |
| Interviewer | 0:06:39.4 | Actually, I mean... The (0:06:42.8) released that report = |
| P3. | 0:06:45.9 | Yes. |
| Interviewer | 0:06:46.4 | = this month. Seems like it's been a long time coming, it should have come out a lot earlier and... |
| P3. | 0:06:53.1 | Well it's the second round. They did it in [REDACTED] and at that time they did it with no press and no strategy, they just did it. And now, there's a much bigger engaged infrastructure to take advantage of that report until leveraged for more political purposes, so I think we're, you know, in a better position and the analysis is more sophisticated, so we're not just talking about, you know: "Here's all the horrible things that are gonna happen to people (0:07:23.8) because of climate change"; but if we address climate change, we will have enormous health (0:07:29.1) benefits in addition to economic benefits for our society. So, it's really trying to bring the health arguments to the (fore) in a positive way. |
| Interviewer | 0:07:42.6 | Yeah, it's extremely important just to be more engaged and have more of a framework for thinking about these things. |
| P3. | 0:07:50.6 | Yeah. |
| Interviewer | 0:07:51.0 | Which brings me to the next question. How does your organization define success? |
| P3. | 0:07:59.9 | We do a lot of stuff by measuring the impact of our work in the sector. So we know, for example, we try to get the healthcare hospitals to identify a problem with us and then to change their purchasing practices or policies, in many cases its purchase and practices. Initially to change the marketplace for those products, so trough their purchasing essentially they're making policy by buying things that are more environment responsible, that are safer. So, you know, an example of that, in our world was getting the healthcare sector to stop buying mercury thermometers. [REDACTED] So for us is kindda interplay between market and policy to transform practices and products. [REDACTED] |
| P3. | 0:00:04.0 | It's my point of view... it's just the beginning; it's just a very early indication that we can have a big impact. |
| Interviewer | 0:00:11.3 | Yeah |
| P3. | 0:00:11.9 | It's good, and it's very... it's the beginning |
| Interviewer | 0:00:19.9 | Okay, it sounds like... the way you think about impact it's a bit about kindda translating things into practices and policy. (0:00:28.7) as well, particularly [noise] going on a global level. |
| P3. | 0:00:33.6 | Yeah |
| Interviewer | 0:00:34.0 | Are there any other ways you think about impact? |
| P3. | 0:00:37.8 | Yeah, i mean, so... We also do some kind of straight-up advocacy, and so we were trying to build a global consensus at a* advocacy level that we need to rapidly phase out coal, both from a public health point of view, but also from a climate change forcing point of view, if we're gonna have any hope of addressing climate change. We have to eliminate coal over the next 20 years. So, we're working in a number of key countries which are still committed to coal, to bring the health arguments into those energy debates, those policy debates, to say: "Look, you maybe think this is a cheap energy source, but the public health consequences are massive"; and i think obviously that's starting to change the way the [REDACTED] government thinks because of the massive deaths and air pollution of [REDACTED] and other [REDACTED] cities, and we're trying to create similar momentum in [REDACTED] |
| Interviewer | 0:01:53.6 | Wow, very nice. |
| P3. | 0:01:58.5 | Another one, another example of that would be: We've created a [REDACTED] healthcare climate challenge to get hospitals and healthcare systems around the world making significant commitments around their own climate footprint and measuring together greenhouse gas reductions. And so, we are trying to roll up thousands of hospitals, not the numbers of thousands of hospitals to show how they could, as a sector lead by example in the transition tour of renewable energy economy - low carbon healthcare. |
| Interviewer | 0:02:35.7 | Yeah, wow, that's great. |
| P3. | 0:02:38.2 | Another example would be... We're in a project with the United Nations to develop environment ( 0:02:46.1) standards for their whole system, like UNDP, UNICEF, whatever... the other, WHO, UN Population Fund and so, for us success would be implementing those environmental standards and transforming their $5 billion dollars a year of purchasing toward safer and more environmentally responsible products. |
| Interviewer | 0:03:22.7 | Okay, so it sounds like the work that you do, you talked a lot about the policy makers. What are the government (0:03:29.9) [noise]. Do you use the data and the work that you do to influence any other actors? |
| P3. | 0:03:40.9 | Let's see.. Yeah, like documenting... by documenting the health costs of reliance on dangerous technologies would be environmental exposures from certain medical devices. we're trying to influence obviously all the (0:04:08.5) companies around changing their practices, so that they themselves are also innovating to address demand being created by the buyers, the hospitals and health systems, so we're trying to influence the whole eco-system in that regard. |
| Interviewer | 0:04:27.4 | Yeah, that's it |
| P3. | 0:04:32.9 | And also, i mean... I would say there is three roles in this era of climate change that i think healthcare has to play. One role is about being resilient. And that means that they are designing healthcare that it can withstand the extreme weather events the climate change is bringing and be the anchoring response related to those climate related emergencies, so we can't have a situation anymore like happened in New York on in Hurricane Katrina were basically the system's fails. They evacuated the hospitals and then people are screwed, it's crazy. So they have to be the last buildings standing and know how to respond. That's one. Two, they should lead by example, I’m describing in the way around changing their food practices to support sustainable agriculture, changing their energy practices to (0:05:40.9) energy, detoxing their supply chains to bring safer and more environmentally superior products to the market. All of that. Water, pharmaceuticals, transportation, buildings, all of it. And then... Third is being messengers*, advocates, because they're enormously trusted professionals in our society, more than anybody else. And so they can raise their voices in support of policies to put a price on carbon, to, you know, phase out toxic chemicals, to substitute sustainable agriculture instead of all this industrial agriculture that's destroying the planet and people's health. They need to be advocates for those policies that are essentially health policies, all these are health policies. |
| Interviewer | 0:06:34.1 | Yeah, sure... I don't think the (0:06:39.2) mentioned before, the healthcare sector hasn't quite caught on to what impact they can have and (0:06:45.4) if there was some way of kindda of (0:06:50.3) and get them to lead by example, be advocates for climate change would be fantastic. |
| P3. | 0:06:57.0 | Exactly. |
| Interviewer | 0:06:59.3 | So, moving on to the next question. Sounds like you had a lot of success in a lot of the different projects that your organisation has been running. I was just wondering how do you measure the progress of your work, so what outcome variables do you use, why do you use these? What methods do you use, that kind of thing. |
| P3. | 0:07:20.6 | We collect a lot of data from hospitals, so one thing that I could send you is we ran a three year initiative in the [REDACTED] called [REDACTED] initiative and we created in [REDACTED] different sustainability challenge areas, we created metrics and then got hospitals to participate in this initiative. I think overall there were about twelve hundred hospitals in the [REDACTED] and then they could pick one of [REDACTED] challenges and they have to submit data. And so we, over the course of three years we got more and more hospitals to submit data and to show progress. So i can send that to you. Cause it will capture a lot of what I'm talking about but so, for example. In the energy thing it will be showing there're... energy efficiency improvements. In food they've been showing, they've been documenting how much they were eliminating sugar-sweetened beverages from their hospitals and how much were they redirecting their purchasing toward local unsustainable farmers. Thus reviews they role as economic anchor, not just anchor of health. In chemicals, they would be switching from IV bags and other equipment that had these... that were made from PVC plastic to alternative plastics that didn't have the same environmental footprint. Yes, each area had very specific metrics, so we do a lot to measure that and then we have a membership organization that collects a bigger set of data from a smaller set of hospitals that apply for awards. And so it's all sorts of stuff around recycling rates and their use of single-use medical... reusable medical devices and all sorts of goals, there's like so many different metrics. So there's a... probably collect* that from about [REDACTED] hospitals in the [REDACTED] and then recollect the... |
| P3. | 0:00:12.2 | …which was just an extension of what we did in the [REDACTED]. So we're trying to be as matrix driven as we can, in terms of outcome. |
| Interviewer | 0:00:22.6 | Yeah that’s great. So, with something like the [REDACTED] where you’re collecting a huge amount of data, what kind of resources does it take from your organization? |
| P3. | 0:00:35.3 | We had staff people who kindda work with the hospitals and then there’s just people that just focus on the data. It’s bigger production. ‘Cuz the hospitals are reluctant to do that. |
| Interviewer | 0:00:56.8 | Could you give me a rough percent of the personnel and finances that go into the data collection side of things for your organization? |
| P3. | 0:01:09.7 | We have probably about—well, let’s see, in the [REDACTED], let’s say, we have about [REDACTED] people who just (0:01:25.6) in the hospitals, and their overall sustainability strategies. Then we had content experts’ probably [REDACTED] or so on energy clients. [REDACTED] on chemicals, then like [REDACTED] on food, because we have so much money for food. [REDACTED] buildings searching through content experts and then sort of account manager types. And then there’re a few people; probably two people that just focus on data collection and matrix. And then in [REDACTED], and in [REDACTED] we got a pretty small staff-in each place about 5 people. |
| Interviewer | 0:02:26.4 | Yeah. Okay. It feel… still quite a big process and a lot of resources need to be used to collect the quality data that you need, to know what you’re doing and kindda to tell the future directions of what you’re doing as well. |
| P3. | 0:02:42.9 | Yeah, also there’s a lot of hand holding. |
| Interviewer | 0:02:49.0 | Quite a specific question that we're asking everyone is, how do you feel about randomized trials particularly in your field? Would it be appropriate to conduct one? And why, or why not? |
| P3. | 0:03:03.0 | It's not a core concern for us, really. And it’s an expensive thing, and takes a lot of time, and we're much more driven by a case-study and (0:03:28.6). And to the extent that we're trying to show that the particular intervention has some positive simple outcome. It hasn’t been a big piece of our work. But if we were doing that, then we would probably work with the hospitals at best, as opposed to doing it ourselves. |
| Interviewer | 0:04:15.5 | It is a very expensive, time consuming... |
| P3. | 0:04:17.6 | I just think its little over the top sometimes. |
| Interviewer | 0:04:24.2 | Yeah okay. So I guess… tell me, referring more to the research that you’re undertaking within your networks. What are some of the challenges that you’re facing in conducting you’re evaluations? |
| P3. | 0:04:39.9 | Well, beside* just sort of getting people just to submit the kind of data can be translated into across systems. That’s the big issue. We struggle with that a lot on an international level because everyone has their own system. So that’s a big issue. Even as healthy hospitals initiative in the US. The thing that taked* us the longest to do was to figure out the matrix that everybody agreed to, and to measure it collectively. So that's a big issue. Also a big issue, extracting information from hospitals is challenging. |
| Interviewer | 0:05:47.3 | Okay, in what way? |
| P3. | 0:05:52.3 | All of those are big things. |
| Interviewer | 0:05:56.3 | Okay, in what ways is it difficult to extract information from hospitals? |
| P3. | 0:06:03.1 | They’re busy people, and you’re dealing with multiple departments and they may not have been collecting this data so much in the health care at least. There’s* a lot of black boxes about health picture bought. What things actually cost. It’s probably why it’s so expensive, because there’s so many inefficiency and corruption along the way. Not corruption like petty corruption, just undemand corruption-things being costly, certain amount of money, and you can’t really see what they do cost. So that’s (0:06:55.5). Then, I think it’s the true general health care. Unless you force people to get data about anything, they (0:07:11.8) in this country we had to force hospitals to tell us what you’re hospital exception rate, they don’t want to tell you. What are your injury rates? You have to force those things on hospitals. What is your definition rates? All these things, they really don’t want to tell you. We're mostly trying to do when we're asking those kind of questions is make them feel good and take the money. In these kinds of things is good to raise them up, because it makes them feel good in their (0:07:53.4) By reducing our energy and intensity we're saving money potentially by buying healthier (0:08:07.6). |
| Interviewer | 0:08:16.3 | So, if we want to take away the two big challenges that you have which is having a common metric across different context and also accessing the data that you need, are there any challenges that remain? |
| P3. | 0:08:32.3 | The big challenge is that if you look at--I mean it goes back to the beginning (0:08:41.4) The big challenges is that mainstream health care, even as it starts to address population health issues, is it really yet evaluating the environmental health conditions in the communities that they serve. That is a fundamental problem. If you look at these NCD coalitions they're just not yet focused on these core issues. That's the big issue. If you’re in terms of what--this broader coalition that know we've been talking the school center about, and school foundation and these other health groups. The reason that I’ve participated and actually help to drive that initiative was in a way to infiltrate that thinking, to say "Hey if you guys are working on all these different issues around global access to health care, and higher equality implementation around the world." These things can be incorporated. We're never gonna have--our organization will never have the capacity to deal with all of that implementation, but if other people start saying, "Oh yeah we're gonna deal with these issues" then it becomes more mainstream. That’s the really big challenge I think for us, and that's why we want to keep… |
|  |  |  |
| P3. | 0:00:00.0 | This is why we want to keep our foot in the door with these (0:00:03.6) so that we can... we've got very actionable things that hospitals and health systems can do to improve their environmental performance. And so, the more that we can bring that to this other coalitonal efforts, the better it will be. |
| Interviewer | 0:00:22.1 | Yeah, right, that's great. So i just wanted to change (0:00:25.5) a little bit cause you are talking a bit about how... sometimes what the healthcare system isn't doing, which is they've been not measuring the environmental outcomes can be harmful* and i guess in the.. In the health initiative sector there is... There have been instances in the past where programs, despite their best intentions, were more harmful than good. For your organization, have you thought about ways in which your program can be harmful? |
|  |  | **0:01:01.1 [noise] 0:01:09.5** |
| P3. | 0:01:10.4 | Yeah, I mean, I guess if we were saying... Here's a good example. So, you know, we would like the healthcare sector to stop using (0:01:31.8) and mercury based preservatives that's used in vaccines, not all vaccines, but some vaccines. On multiples vaccines (0:01:44.9) to single dose. So, general we think it's not a good idea to be putting mercury into kids; its bad idea. Now if we were (0:01:56.3) about it, we'd say: "you have to get rid of (0:01:59.5) at all counts). But if we did that and that led to people saying: "we're not going to vaccinate our kids against somebody's deadly infectionate* diseases"; that would be harmful. So we don’t take that position, we say: "Need pharmaceutical industry to develop safer alternatives to this preservative". And wherever we can we should use single-use vaccines, so that we don’t have to use this in preservatives. That's an example. Mostly what we're doing is just... it's such a wasteful and ignorant sector around its own environmental footprint, you know, they're putting toxic chemicals in building materials that than all the workers are exposed to, you know, they're building... they have been building buildings with no natural life, so the buildings themselves are on life support, you know, not the people, but the buildings, and so it's not even healthy for their own employees, so it's such a huge learning curve for them to understand that actually the facilities should embody health, people should go in there and say: "wow, this feels really healthy. This feels good. This is where I'm gonna actually get* better". I mean it’s so bizarre how it's gotten so industrialized. |
| Interviewer | 0:03:31.5 | Yeah. |
| P3. | 0:03:32.3 | So, that's what we're trying to fix. |
| Interviewer | 0:03:34.7 | That's good. Hospitals are not very nice place to be in, and when you put all the issues to (0:03:41.8) the environmental aspects, it's even worse place to be. So... |
| P3. | 0:03:48.4 | You could die there. You get all these infections and these antibiotic resistant bacteria just in hospitals. Oh my God, Jesus, it's insane. |
| Interviewer | 0:04:01.9 | Yeah.. So P3, coming up to the final question now, I'm gonna end up on a high note... |
| P3. | 0:04:08.5 | That's smart. |
| Interviewer | 0:04:10.0 | In a completely perfect world what would help you do your work better? |
| P3. | 0:04:16.5 | Yeah... Okay... One, I'll have a lot more money |
| Interviewer | 0:04:28.6 | Yep. |
| P3. | 0:04:30.0 | Two, the governments of the world would mandate that their systems had to develop programs to both do climate resilient planning, climate and environmental mitigation programs so that it was requiring, in the same way they are requiring more and more quality indicators for healthcare and outcomes. They would require similar set of indicators and metrics around environmental performance. Why not. That's one. And then the second thing... the third thing in addition to having lots of money would be to make a* environmental health literacy core component of all future healthcare training. Doctors, nurses, other (0:05:40.0) gotta understand the link between the environment and people's health, I mean really, that' such a bottom line issue, and yet it's not mandatory in medical schools and nursing schools and that's insane; doesn't make sense. |
| Interviewer | 0:05:51.5 | Yeah, I see. Particularly with the last two you mentioned, so government mandates as well as (0:05:59.5) Is there anything that would... that you had, in terms of resources that would your organization achieve that? |
| P3. | 0:06:12.8 | Those two last things? |
| Interviewer | 0:06:14.1 | Yeah, the last two points. |
| P3. | 0:06:16.0 | Yeah, i mean, we can work with the government, any government, around how to do those last two things. Say, we want to develop standard set of metrics they’re gonna require hospitals to do around the environment, we could easily (0:06:33.8). So, in fact, we might be working with the World Bank to, sort of, say: "Okay, as they think about their loan, and programs to countries around healthcare, what could they mandate for those health systems in the loan problems?", that's a good example. And on the last point, there's* just a... couple of months ago, trough the initiatives of the Obama administration, they got 30 deans of medical schools to come forward and say: "Okay, we're gonna train the next generation of health professionals around climate". Like, that's super helpful. It's gotta be mainstreamed. We can’t be a (0:07:22.9) thing, it's gotta be... these things have to become standard. |
| Interviewer | 0:07:26.6 | Yeah. |
| P3. | 0:07:27.3 | So, you know, i think a lot of the material is there for all of those things. It's the political mandates that are needed at the moment. |
| Interviewer | 0:07:43.1 | Wow, yeah. I definitely can't agree more with that, cause there's often a lot of good ideas and good research behind them, and yet the political support isn't quite there, and it's so powerful to have that political support behind it, to make it come an amazing program. Even let people realize that it's a mainstream issue. |
| P3. | 0:08:06.0 | So we're gettin... |
| Interviewer | 0:08:08.0 | Yeah, we're getting there slowly, slowly. |
| P3. | 0:08:10.4 | Yes. |
| Interviewer | 0:08:11.5 | So P3, that actually brings us to the end of the interview and I just wanted to respect your time cause we did say it was gonna be a 30 minute interview, and it's been slightly overtime, given that we started late. So I apologize for that. |
| P3. | 0:08:22.7 | No worries, its important stuff. |
| Interviewer | 0:08:25.0 | Yeah. But it would be great if you could send me the... The Health to your Hospitals initiative papers, just to look a bit further into the metrics you guys have worked on before and any other papers you think would be useful; send them across... |
| P3. | 0:08:43.7 | Okay |
| Interviewer | 0:08:44.5 | I'm very excited about looking at that. |
| P3. | 0:08:46.6 | Great |
| Interviewer | 0:08:47.7 | Do you have any questions? |
| P3. | 0:08:50.1 | No... What's your... I guess I would like to see what you guys come up with in the end of the day. |
| Interviewer | 0:08:56.4 | Yeah, yeah, definitely, we'll keep you updated. We're going through several interviews at the moment and we collect them together into a report and have them presentable. And that will... I'm planning to have that done towards the end of the year and definitely by December, so i'll keep you updated on that. |
| P3. | 0:09:11.9 | Awesome. |
| Interviewer | 0:09:13.4 | Cool. Alright, thank you very much for your time P3. |
| P3. | 0:09:16.0 | Now, have a great day. |
| Interviewer | 0:09:17.4 | Yeah, you too. |
| P3. | 0:09:19.0 | See ya'. |
| Interviewer | 0:09:22.9 | Thank you, bye. |
| P3. | 0:09:17.2 | Yeah, you too. |
