# Supplementary material for: Supporting Better Evidence Generation and Use within Social Innovation in Health in Low- and Middle-Income Countries: A Qualitative Study
Source: PLoS One. 2017 Jan 26;12(1):e0170367. doi: 10.1371/journal.pone.0170367 (PMC5268497; doi:10.1371/journal.pone.0170367)
Supplement: S1 Dataset — (ZIP) [file pone.0170367.s002.zip › Data/Data - Interview transcripts/P4.doc]

| Interviewer | 0:00:00.0 | I want to respect your time, so how we get into the mid of things |
| --- | --- | --- |
| P4 | 0:00:05.7 | Sure. |
| Interviewer | 0:00:06.2 | I just have a couple of questions, should take about 30 minutes and can't wait to hear your thoughts. |
| P4 | 0:00:12.7 | Sure. |
| Interviewer | 0:00:13.7 | So i think we're gonna start of, i kindda know what you do, but if you could tell us in your own words, kind of, what you're up to, what's the problem you are trying to solve? |
| P4 | 0:00:24.8 | [REDACTED] |
| Interviewer | 0:02:02.1 | Great, okay, super. So, I can... Maybe (0:02:07.0) after our chat. But let's talk more about your other organisation. What type of... So you just offer all the various people you are trying to influence in the process you're undertaking to establish it as a best practice, just to scale this intervention. To begin, how... What metrics have you set up, or what types of evaluation have you set up to ensure... Or to test basically, whether this is the best practice and whether you're not causing harm, that sort of thing. |
| P4 | 0:02:41.0 | 0 Sure, sure. So, the principles of the program are to improve well-being, to improve wellness and to reduce healthcare related costs. [redacted]. All of this kindda drives the need for some change. In [redacted] as we established a pilot, we were looking just at what we could measure in a short period of time and we found... that we wanted... of course, demonstrated that we can operationalize this, but we wanted something that was harder than that. So we did first a... I mean, on the pilot we did a qualitative assessment looking at how the program affected both the beneficiers* - the clients and then how it affected the the people who we employ, we call [redacted]. But we also use the tool called the "WHO5 well-being assessment tool", which is five question instrument that could be administered and self-administered or administered by our [redacted]. and we did it a baseline and did it every month to the people who were receiving care and wanted to track the changes in well-being amongst the people who were receiving our programme services from a baseline of "no service at all". And we were very pleased to find that there was 47% increase in well-being scores amongst the entire population that (0:04:29.8) after one month of service and then (0:04:32.5) of the program. We were expecting a 10% increase after six months and so got nearly a 50% increase after one month. We also so a greater than 90% reduction in people who had scores associated with depression and scores associated with low mood. And so that the... Those were the hard measures. Now, as we go to [REDACTEDT], in addition to measuring well-being, we were looking at the number of hospital days, the number... You wanna look at the cost metrics, and really demonstrate that we can reduce the healthcare costs, and that would include, of course, people going to emergency rooms, being admitted to hospitals, people who're being discharged being readmitted to hospitals, but we think that we can provide the reduce the cost of providing service to older adults. And as we look the bills, you know, piles (0:05:28.7) we'll be looking for partners who can give us that data. So we're about to hear from (a company) whether they're willing to do this project with us, but (this company) would be the perfect partner, many expect, because they do have such robust data systems. |
| Interviewer | 0:05:45.4 | How's the intervention developed? What types of resources or knowledge did you all drawn when you were coming up with... [overlapping speech] |
| P4 | 0:05:56.4 | Okay, much of this was (0:05:57.0) on the " our organisation " model, so we took the " our organisation " peer-to-peer support model and said: "How would this work in a different population, a population of older people". And last year, when we did the pilot, we did it in [REDACTED] and we did it out of our organisation offices tapping into much of their knowledge. You know my experience with our organisation. I think what we learned, in terms of delivering the program will then be supplemented by what we've learned about taking our organisation to scale. How do you scale something once you've demonstrated it works? |
| Interviewer | 0:06:34.7 | Absolutely. So these early results you are talking about... Which sound just incredible, that's a great achievement, amazing and really encouraging. Were they... Are you now gonna use... Gonna publish that material, do you use that when you talk to [REDACTED] How do you use the resources that you do? |
| P4 | 0:06:57.9 | So, the next step is... We had an opportunity to present, to submit case study to the WHO. There was kind of a competitive process where the WHO is looking for best practices in ageing. [REDACTED] The interest is in using those results in resource... to raise interest in resource poor countries and to find either program partners or funding partners to take that forward there. In [REDACTED], well, you know everybody appreciates a good study; no one really appreciates a good study that wasn't done in [REDACTED]. So we've been able to use results to at least demonstrate that we can take an idea to the field, cause everybody's got ideas and the first trick is to actually demonstrate that your idea has some legs. So, we were able to demonstrate that has legs and the results were impressive to some and dismissed by others because they've been done in [REDACTED[. So, (0:08:41.5) interested in establishing another pilot to replicate what we've done on the well-being side, but also to extend it further and to demonstrate that we can do this in relation to wellness outcomes, and that's what I mentioned earlier, to reduce service utilization and ultimately costs. |
| Interviewer | 0:08:59.4 | So that first study, what was so... That was before and after or there was counterfactual? What was the design of that study? |
| P4 | 0:09:08.0 | It was a cohort study. We collected [REDACTED] a followed them from time zero for the duration of the year and that was the end of the study. And then we were unable to find sustaining funding, so that program shut down, sadly. |
| Interviewer | 0:09:24.5 | Oh... What do you think accounted for the... By someone who is (0:09:33.5) to do the fundraising now with your organisation .What do you think accounted for the difficulty with this thing in that program? |
| P4 | 0:09:40.6 | [REDACTED] |
| Interviewer | 0:14:50.2 | What type of... What do you think would convince more... What do they say, and what is your intuition (0:14:57.1)... |
| P4 | 0:14:58.4 | I think that... We've done probably between eight and ten lines in the water with health systems in plans that are nibbling. We just a call from [REDACTED] on Friday, (0:15:13.4) in giving us a million dollars to launch this. And we'll find out this week whether that drops. But i think that as soon as one drops, the others will drop. I think it's having the bravest kids (0:15:25.2) first, and then all the others fall out. |
| Interviewer | 0:15:28.6 | Great. All those studies are sort of group thinking. That's exciting |
| P4 | 0:15:33.7 | My friend says that it's like the first olive of a jar. |
| Interviewer | 0:15:38.3 | Okay. Just one? |
| P4 | 0:15:42.0 | Have you ever opened a (0:15:43.8) jar of olives and you turn it upside down and they don’t fall out? You just take that first olive and they all come through. That's a lovely analogy; I’ve wanted to use it since he gave it to me. Now I have. |
| Interviewer | 0:15:57.9 | Excellent, I’m glad. [overlapping speech] |
| Interviewer | 0:16:00.0 | Thank you. Great way to start the week, culinary analogies. [REDACTED]: "What you think about the case studies?" I think a lot of different funders or (0:16:24.0) organizations have different sort of evidence standards. Some are very clear apriori*, some not so much, come require RCT's, some are more interested in info graphic work 'cause they're answering different questions. What do you think they are trying to get out with the case studies and how much of the data you've put into it came from the (0:16:49.3)? How did you guys write it? |
| P4 | 0:16:52.7 | The case study all came from the covert we did. I mean, case studies are snapshots, and... In the world of the science in which i was raised, it's all about the RCT. And the RCT is the most credible evidence out there. But the truth is, very few... When you go to an industry, there are very few industries capable of really wanting an RCT, they just want, what I call industrial level proof. And you kill yourself and spend enormous sums of money and many months of time trying to generate an RCT, when really all somebody wants is industrial level proof. Some reasonable demonstration that what you proposing works, against some reasonable comparison group. And as we've talked to health plans and hospital systems, they don’t want RCT's, they just want something that they can grab onto and say: "there's reasonable evidence that this works". Case study is a snapshot and it's good as the criticals* reading it and we could, after a year with our project become, kind of, the poster child for an ageing program to be an only reflection of how poor the evidence is for other programs, and how good ours is, like that. It means... a lot of this community service organizations that are doing projects; they really just don’t have the capacity to put together a case of what they're doing, in terms that are more credible that we can. And these are organization that have been doing for ten, twenty years, you know, the fact is that I'm a [REDACTED] scientist and when you... what does that matter, you know, different (0:18:38.1), a sort of a community organization in Vietnam or Thailand are trying to do the best they can. So, I mean, if you asked them to be cynical, I just tell the story better than other people did on this occasion, doesn't mean my evidence is any better. But it's the currency that's available to most people. So, I mean, that's my cynical view of it. |
| Interviewer | 0:19:07.4 | Do you think it will be helpful if folks were more educated about... the sort of chronicle (bias) producing. Kind of actual methods that you are coming... from your background... or do you think (0:19:36.0) [overlapping speech] |
| P4 | 0:19:37.6 | I mean, the truth is, there are people out there trying to do good. I mean, they really are. We got pretty sniffy about datas* and the quality of the data and the evidence and the kind of evidence. And there are people out there just trying to make a difference in some people's lives. And I do appreciate how important is when you have resources to allocate, you want to allocate them to the most effective intervention that's out there. It's widely important. When the US government is putting up $15-45 billion for pep farm. I watched, in those first years they were just putting up money for inputs, "Give us a credible input, we'll pay for it." Because there were so few inputs. And then they were short and they said, "Let’s give money to the best output" Who's seeing the most patients? Who’s putting the most people on drugs? And then they got a little smarter and they said, "Well now we got to look at outcomes here. And let’s put the money in those organizations that have the best outcomes." And then finally they started to look at impact, and then they got really tough and they said, "What’s the contribution to the impact we're seeing because there’s so many interventions. We really wanna make clear--what's your part in all of these?" But at the beginning of it--of an emergency, people aren’t looking for hard evidence; they’re looking for just something. And its rough out there, the community service organizations that are just trying to get something done, and they don’t get funded because they don’t have evidence, and they don’t have evidence because they don’t have the money or the smarts. My first evaluation cost 3 times my budget. Now that is wrong, sorry. The population council did it through an organization in [REDACTED]. And by the time the population council organization in [REDACTED] took their money and paid the people who are very good, very smart scientists. They'd tripled our budget. |
| Interviewer | 0:21:52.1 | So do you think--I mean that's some horrifying story but also not an uncommon story... |
| P4 | 0:21:57.5 | Yeah and we've all been doing that long enough that we've kindda understand how it works. You really can’t do your own RCTs and you need somebody to come and designed it and consult on it to get it right. God forbid you should do a RCT wrong. With all the money people over time invest in it, you get it wrong and that's a real tragedy. |
| Interviewer | 0:22:30.3 | So I guess… two questions. One is, you must have seen a bloated enterprise, ‘cuz you’re trying to squeeze every penny you have and put it in the programing and make change and get things done, and then this evaluation comes in and triples your budget. Question #1 is do you think as a scientist yourself are there ways that you see that we can actually run an incredible evaluation? More cheaply, more efficiently, more in a timely fashion that would empower organizations to do things more rigorously themselves? |
| P4 | 0:23:14.2 | 2 Well I think we have to. If the RCT remains the Holy Grail of evaluation and we hold everyone to that standard. Either we're not gonna get the evaluation that we want or--it's interesting that the (0:23:33.6) that the New England's Journal obviously come out, kindda highlighting how many studies that were being done, are done in a way which aren't proper. That the data which being published isn’t credible. These things that are going to peer review journals, the data's being manipulated. And having been a reader of pharmaceutical related research for years, you see RCT that are being put up by the pharmaceutical companies which aren’t--where all the data is being manipulated. And any of us who've done this long enough know how to manipulate data. You know how to shade the numbers, you know how to shade the story to get the outcomes you want. I think the burden is on us to try and find a better way to get the evidence, to make it accessible to others. Certainly while we're in control we of the science community we can make up whatever rules we want in terms of what’s credible. Would it be nice if we could find a way that made it more accessible to people working in the field. Though there's no obligation, but because I'm one of those guys working in the field would certainly love to find a way to create a more user friendly way to generate an evidence base. |
| Interviewer | 0:25:00.4 | Yes I guess that's my second question then, of that story is, "In a perfect world what do you think would… what types of inputs or resources would help all community and organizations, social innovators? |
| P4 | 0:25:17.8 | We've talked about this in Japan; so often people do interventions just because there's a need and other only later go back to say, "Well we need to measure this." They don’t design the intervention as something that's going to have a future effect that needs to be measured. It's kindda after the fact they say, "Hey this works lets measure it now." And it’s very hard to go back and get a base line once you've started doing something so that you can get measurements. Our biggest challenge with (0:25:51.6) was what was the base line. How do you create a base line before you've actually started a project? I don’t know, I really don’t know. And because I’m not involved in research design in a big way, I’m not of the science, I would almost challenge the people who are of the science to think of, "How does this get done? How do you do, look backs on intervention that started as simple intervention?" A couple people getting together, trying something and then it works and then you try it a little bit more and it works, and then you think, "Cool lets raise the money and turn this into a program". |
| Interviewer | 0:26:35.8 | So basically you had to solve this puzzle then in some sort of way that... |
| P4 | 0:26:42.2 | Yeah. Just in conversation with you, how'd you go back? You’re two years into trying to do something and it works, let’s turn it into a program, and someone says, "We need some evidence that it works" (0:26:56.6) if you heard the expression "Let there be anecdotes." Because if someone said, "Give us evidence “we bring out someone who can tell a personal story, and the anecdotes are powerful. [REDACTED] And there's a donor based response to that and doesn’t respond to what we did in RCT and there were a 100 people in this group and a 100 people in that group, and know here's confidence intervals (0:27:40.1) |
| Interviewer | 0:27:41.8 | 8 I’m definitely sympathetic to that. [REDACTED]. But at the same time, then also knowing the literature, you see in things like the “Cambridge Sommerville” study or people who are just debating in (0:28:11.0) All these programs that were well intentioned, where you had this personal testimonials as well, people are asked to evaluate the program that are so positive and then yet when we looked at the outcomes for those participants versus counter factual and all of 3-4 of those programs. We saw harm, we saw people who were actually--they've been to jail more often. So not only with the program--not effective it was, it was more harmful. Given sometimes the conflict between sort of objective outcomes, I don’t if I really like that term, but objective outcomes (0:28:51.9) and these narratives that people spin about their own lives I’d say they’re unreliable narratives of their own lives. How responsible do you think is to use anecdotes or do you think there's a problem there? Do you think that, what most of the time… |
| P4 | 0:29:08.5 | Don't know. The burden is on all of us to both come up with better evidence and help people, give them the resources to come up with better evidence. You find that people who've done incredible RTCs, and years later you look back and despite all their RCTs you find they have embraced practices that have no value. All the value was compute from RCTs that were done in the best scientific rigor. And years later it's all rubbish. So I don’t really know, but I experience the frustration, not just on my behalf but on others as well-people who are trying to do good in the communities and seem to be doing good, and they just can’t come up with measurements that are credible to a scientific community. The scientific community is also trying to do good and using RCTs as the lever and 5 years later that wasn’t as effective as we would wished* to had been, but we believe it was based on the early evidence. I think that this is one of the challenges that we all face, and certainly the small organizations who are trying to get some traction, by traction we mean attention and money are hailed to the highest standards, and of course can’t meet them and so crush. (0:30:44.6) and I don’t know what the solution is. How do you free up people to innovate? How do you create that resource for people who have ideas that may get lost if they’re not embraced early? And how do you avoid embracing insignificant programs that are never gonna go anywhere? |
| Interviewer | 0:31:12.7 | Oh man, I wish we had a lot more time to keep discussing. I really... |
| P4 | 0:31:19.9 | I don't think there're answers. This is... Perhaps the debated isn't (0:31:26.1) It's nice to discuss this. How do you create meaningful evidence in early days programs amongst people who don't have the human resource and the financial resource. And then you promote innovation, how do you not extinguish this small community generated projects. |
| Interviewer | 0:31:50.5 | One thing... That's kind of, you know, this is why we were starting this conversation, you actually... I think this is gonna be one of those direct (0:31:58.6) [overlapping speech] |
| P4 | 0:32:04.3 | I've thrown some stones here, either please (0:32:06.7) [overlapping speech] |
| Interviewer | 0:32:12.3 | I think it's gonna be (0:32:12.4) it's... don't worry, like we said, it's in the information (0:32:17.0) anonimised*. |
| P4 | 0:32:20.5 | Please go check hospitals on this one, please. |
| Interviewer | 0:32:22.0 | Oh yeah, no, of course. Talking to people so often you get these beautiful articulations and that was just one; it was of exactly what we were trying to do here. Star that conversation and bring in... Not just within this group, but the (0:32:38.2) talking to the government. I think another funders think this is often... (0:32:46.9) happens if everyone works together and... cause these are (0:32:52.9), like you say, and but also, we clearly... Evidence isn’t the only thing, but it's also something we can do better. But if not clear, that happens. Anyway, like I said, I wanna respect your time, and again, thank you for such a great discussion and sharing your insight. And, yeah, this is gonna be an ongoing conversation, so we'll be definitely feeding back. Yes, some ideas people have about how we... Some of the answers that are generated for executive questions you just posed, it will anatomized, but hopefully it will be rich and we look forward to reconvening at that time. |
| P4 | 0:33:36.4 | Thanks a lot, you take care. |
| Interviewer | 0:33:37.5 | Have a nice day. |
| P4 | 0:33:39.1 | Goodbye. |
| Interviewer | 0:33:39.8 | Bye. |
