# Supplementary material for: Supporting Better Evidence Generation and Use within Social Innovation in Health in Low- and Middle-Income Countries: A Qualitative Study
Source: PLoS One. 2017 Jan 26;12(1):e0170367. doi: 10.1371/journal.pone.0170367 (PMC5268497; doi:10.1371/journal.pone.0170367)
Supplement: S1 Dataset — (ZIP) [file pone.0170367.s002.zip › Data/Data - Interview transcripts/P5.doc]

| Interviewer | 0:00:01.6 | [REDACTED] |
| --- | --- | --- |
| P5B | 0:06:22.0 | I think the important thing to add is that we work with multi-professional team, we have doctors, psychologists, nutritionists, lawyers and all of them have seen the whole plan and seen the development of the family, the needs of the family, talking to the family each month and seeing one test should be changed so that the family can achieve the goals. It's a very good support system. They're approved for the teenagers and the family, for the mothers with small children and P5A told you for vocational training also. And so, it's very holistic and the most important thing is we do everything listening to the families and understanding their needs and their challenges. |
| P5A | 0:07:28.8 | And I want to add something as P5B said, although we have a very important, you know, professionals, they... Many kind of professionals: lawyers, engineers to review the housing of the children, physicians and so on. Our work is based in volunteer work. More than one institution want to be like a movement of the civil society. As you know in [country], society is not as well as, you know, informed as well, as organized as in [REDACTED]. So, our institutions have a lot of (0:08:16.3), is a twin role, is not the volunteers they have there, because they have good... No, they change their values, listen to the people they serve. |
| Interviewer | 0:08:32.0 | Okay, thank you for that overview. I wanted to actually ask a little bit about... For more details about P5A was saying, vis-a-vis, measuring impact and the results. So you talked about a study with [REDCTED]. I guess my question is: One - How are you keeping... What sort of things were you measuring before you partnered with [REDACTED]? And two: What did that partnership, you know, enable you to measure or to examine that you weren't able before? |
| P5A | 0:09:14.8 | Yes, okay. |
| P5B | 0:09:15.3 | You have to answer this question please. |
| P5A | 0:09:18.6 | Yeah, I can answer. We have a very broad database system and in our database we've put everything about the family. In the first... In relation to the family we ask lots of questions about the five areas in which we focus and so we want to know about the living conditions, the house of the family, about abilities and how the family is generating income, if the children are in school if they are having problems at school, about the health of all the family and not only the sick child that came in the first place. About documents, about government benefits, about everything. Based in this initial evaluation and in a house visit, because when the families are sent to our organisation, the first of things that are done: the initial evaluation and a house visit. And based on this too, we such the build the family action plan. Every month, when the family comes to our organisation to check the family action plan, we ask some of the questions that are asked in the first place again. And every six months there is a periodic evaluation that's broader and has all the questions of the initial evaluation. So we can measure for instance: how many times the child was hospitalized in the year before entering the program and then we're measuring days of hospitalization every month. Or for instance, if the child was at school the year before and if the child is now. If the mother was generating income and if she's generating income now. We could evaluate what was being developed and the gains the family was having or sometimes problems that they started facing after entering the program. With [REDACTED], we could measure the long-term impact. So, three to five years after being released from the program; how are these families, were they still evolving in all those areas? And the great thing about the study is that it showed that the families continued the evolution and they were even better and the results were even better than the ones after the... around two years in the program. |
| P5B | 0:12:08.5 | I just to add, going to your question. What are the main results comparing what we were measuring before [REDACTED], and [REDACTED] adds measuring with the research. I will add some comment regarding what P5A said. That for example, they made a control group. They good hospital*. We find in real, very similar hospital without our organisation, so they could compare the results that children had, the whole family had being supported by our organisation, in another (0:12:57.1) has no support from our organisation. While everything is in our research, their research. And another thing that they (0:13:06.4) it was that we... normally what we measure, what are the basic measures that we had. We had a lot of measures, but the basic were: the income generation of the family before and after our organisation, The days of hospitalization before and after our organisation and the health of the sick child. What regular, civil, good in terms of clinical aspect. So those are, for [REDACTED], the main areas that we look for. But after, [REDACTED] added some other measurements. One of them was evolution of housing. That's very present because, for example, before our organisation, [REDACTED] |
| Interviewer | 0:14:58.5 | Okay. So, from these... How so you use these results? |
| P5A | 0:15:11.3 | Are you speaking about [REDACTED] or what P5B said about our database? |
| Interviewer | 0:15:17.3 | Well, I guess both. So, what functions...? Maybe use the database to improve your own programs within. Maybe you use the [REDACTED] results more for advocacy. What types of, I guess, uses did you make of the different data you are collecting? |
| P5A | 0:15:37.4 | I think P5B can add something, but it think... We had both... What information [REDACTED]. Which we entered this database system. We proved the quality of our work. That's our, you know, main perspective, regarding our database. To have accountability at exactly what we do. What can we prove? What are the areas that we can improve, the better way? So P5B can explain how we do that in our everyday inside this (0:16:16.4). Of course, the [REDACTED]... You know, the social aspect of humans. Everybody knew about the social aspect. About... Many people talked about this, but nobody... I think you people (0:16:34.1) prove the social impact of humans. The relation between disease and social environment. And I think we use the [REDACTED] results to do advocacy for our cause, to attract more people, more awareness regarding this (0:16:55.8). P5, please add some comment. |
| P5B | 0:17:01.3 | Yeah, I think one of the most important aspects of having this measurements is that we can see where things can be improved. Where the families need more help and we can work from this results, the ones from our database. But the [REDACTED] was very important to search potential partners, sponsors and they could see that we have an external evaluator saying that what we were doing was good and what we were doing had great results. And lots of people that didn't understand how powerful the social aspect of poverty and illness are, had a proven result. |
| P5A | 0:17:57.9 | And, I would like to ask something. in the beginning, people in the [REDACTED], when we founded our organisation, people used to say: "You have no focus. You walk in so many areas." And thanks to that we never gave up of walking five years. [REDACTED] If you want to really change people glad, you have to walk in a multidisciplinary way. Because this day, these poor women, they face a very multigenerational problem in their everyday lives. And if you only focus, for example, in housing project, is not enough. So, as P5B said, to overcome (0:18:47.0) of poverty, we work very hard in those five years. |
| Interviewer | 0:18:58.8 | In these conversations you are having with people who, you know, about the results of these evaluations... Who are those people, who are you trying to influence? |
| P5A | 0:19:12.7 | Well, we are trying to influence governments, so they can use the methodology. In [REDACTED] our methodology is already a public policy. And we would like more municipalities to use this methodology as a public policy. And we think that external evaluation is powerful tool to show them why could be good for them to use. Also talking with other institutions, for instance, in [REDACTED] now there's a lot of interest about our organisation and the methodology after P5B went there and give lectures and talked about this study. And so, it's a way for institutions that already work with this group of families living in extreme poverty to understand there's a way to help them to get out of the trap they are facing. And of course, private companies that can sponsor us or use the methodology to deal with the places near their plants and headquarters and to help the communities. |
| P5B | 0:20:45.4 | I want to tell you that, as P5A mentioned, we visualize that... A regularly became public policy in [REDACTED]). But there are 5000 municipalities in [country]. We are very careful to (0:21:03.4), especially in our time in [REDACTED] They (0:21:07.0) that we can trust and influence as we did with the help of [REDACTED] and we begin public policing in (0:21:18.9). Another thing that business people, there are a lot of companies all over the world surrounded by a very poor environment. As (Stephan Schmithand) used to say: "There is no success for company in a failed society." So maybe we could help the business people to implement the family action plan around their own company. Many are the (0:21:48.7) that poor families... They can't understand that our walk... Maybe they can use that. I and P5A we talked (0:21:57.6) in [REDACTED] in a homeless project. And (0:22:03.8) how we follow the family and this was very helpful for her to follow some ideas of our (0:22:11.8). And we are now in the process of creating... We ought to have some sponsor to that online platform, online learning platform. Basically in our, you know, social methodology, they can download some information for them in India or another place for physician, social worker, no matter they are business or (NGO) or government. They can download our... Some of our, some... How can I say...? (Issues) of our platform that doesn't exist already, but we planning to exist someday. So they will download. Some will be for free, some other subjects, they will pay for this learning platform. And they can come to [country] and learn in live experience for one week, two weeks. We're already born [Interviewer]. (0:23:17.3) and we are finishing. We are forming this (0:23:20.0) to become a center of excellence, of social (intrusion). We plan to have annual meeting, inviting people from all over the world if they want to come, to share their experience and to learn from our experience. |
| Interviewer | 0:23:41.3 | That sounds wonderful. That's so very unique and interesting way of sharing and learning cross different organizations. I guess a question then... When you're trying to figure out... Basically are there any big questions about what you do that you have not been able to answer? |
| P5A | 0:24:14.9 | I think, you know, that God... We live in a country, a continental country [Interviewer], as you know.. And really rich, many people, who have already... I don’t know the word about ([family grant]) (0:24:31.1). So we think that we are [family grant] with a way out, you know. [family grant] only gives money. It's very important to give money to a very poor family because they will die. The children will die next day, next month. But, in my opinion, my personal opinion is not enough. We have to deal with a family in a holistic way as we do. We have prepared to increase (government) in a large scale. But our challenge, as I told you, i don't want money from the government because there is a lot of corruption in [country]. What we want as a group, as board members, as staff, we want to replicate (0:25:17.9) experience in a large scale. But to tell you the truth, we don’t know exactly how. To have a center of excellence, maybe a group of people will study. How to get the right people to improve this; to have a huge impact to many lives in [country]? Another challenge, it is in the business area. How can we have advocacy for our cause to be more listened to in [country]? In our times, as you know, our president is running very bad*, our country pays a lot of companies closing... It’s a very difficult time for [country] in terms of companies, governments... but we know that it will pass. I don’t know when, but it will pass. Those are the areas that we think we have... We have a group study (0:26:20.8) our model and how can, you know, beccome a business in a model, in some way for a group of people inside. We can be a consultancy, as we did with (0:26:35.7) and spread our methodology wherever it needs. But that has to be... We have to... (0:26:46.1) helping us in that moment, how can we begin... How can we... People pay us, you know, to explain and to help all the groups of people, no matter if they are business, NGO or government to understand and to use our social methodology. |
| Interviewer | 0:27:12.9 | If there was one thing that could help you do your work better, to do this evaluations better, what would it be, do you think? |
| P5B | 0:27:28.7 | Shouldn’t give your idea. I have my idea that I want to share to. But keep your ideas because you (0:27:35.3) [overlapping speech]. Maybe we... If we could have more interaction with the families over [country] living this reality in extreme poverty, the ones we are not helping now in rural areas and other places. We could see if everything that we do in urban areas could be as effective in rural areas. So it would be good for us to be able to work with families in a different environment and see if the same things apply to them. In my case I think we need... We have five thousand hours pro-bono from [REDACTED] to help us measure the (0:28:31.8) database. And it was one of them. (0:28:35.8) we have this [REDACTED] to (0:28:39.0) and that was key for, you know, the (0:28:42.0) power. I would love to have [REDACTED] again, or another similar company. I'm telling about [REDACTED] ‘cause we had a great experience with [REDACTED]. Those four years pro bono were... I would like to have... We are in another level, we've already proven the social impact of our methodology. But now I'd like to understand how we can scale. As P5A mentioned, in [REDACTED] they want to spread, they know, they liked our methodology in a very profound way. Cause how can we learn how to sell our social... Like a business we sell consultancy for the people that want to implement our methodology. To pay for someone with business skill to help us to make business plan, how to spread our organisation methodology. |
| P5A | 0:29:44.9 | And that would be important. And how we could be (0:29:48.5) sustainable way, i think it would be important for us to understand because sometimes there are lots of people interested in our methodology, but they are not willing or can't pay for us to pass the methodology to them. And so, all who would be paying for us to teach this methodology for others, paying our expenses. It's not a company to have... We don’t want to hire--make money from that, we want to do that with our expense covered. So, I think someone to help us with this part of how we could develop a model that was sustainable would be great. I agree with (0:30:44.5) |
| P5B | 0:30:44.1 | [Cross talk], you know, they do in college in... Of course that you know, it is based in [REDACTED]. And [REDACTED], I don’t know his other name, approached us and some people from the (0:31:00.8) have come to [country] in August and they will make the research. They will learn a lot, but they chose us among many, you know, many (0:31:10.0) they have area. And they want to understand exactly what we do in order to think with us how to scale, in order to make another research. |
| Interviewer | 0:31:22.5 | That's exciting. How is that partnership developed? |
| P5A | 0:31:30.0 | I don’t know exactly. [REDACTED] told me that they had a very... [REDACTED] I can send to the mail the (0:31:37.4). He said there is a fusion list. They have modern (0:31:41.7), I don’t know exactly Institutions from all over the world, regarding innovations, helped and chose four. And among the four we have been chosen and found there in the moment that they will send some people from their college to come here in (0:32:01.9) to study more deeply. To understand how we work deeply. |
| Interviewer | 0:32:12.4 | That's exciting. I want to respect your time and it's been maybe thirty minutes, so (0:32:22.0), I really wanna thank you. It sounds like there's a lot of exciting projects and thank you for sharing. I think these types of partnerships between Mackenzie or different universities and often times outside of a (0:32:35.9) a few small organizations. There's not very many opportunities to set those up and it sounds like they were very beneficial to you and that's exciting to hear, ‘cause that's some of the feedback that... Maybe we can grow that or other things. So, thank you. |
| P5A | 0:32:52.7 | You are welcome. |
