# Supplementary material for: Supporting Better Evidence Generation and Use within Social Innovation in Health in Low- and Middle-Income Countries: A Qualitative Study
Source: PLoS One. 2017 Jan 26;12(1):e0170367. doi: 10.1371/journal.pone.0170367 (PMC5268497; doi:10.1371/journal.pone.0170367)
Supplement: S1 Dataset — (ZIP) [file pone.0170367.s002.zip › Data/Data - Interview transcripts/P6.doc]

| Interviewer | 0:00:00.4 | Thanks again. |
| --- | --- | --- |
| P6 | 0:00:02.2 | Are you gonna go ahead and record? |
| Interviewer | 0:00:02.9 | Yeah, yeah, I'm gonna set that up. So, I guess, just for the record... I'm quite familiar, but just for the record. If you could possibly start up by telling us all a little bit about what your organisation, how did you get involved with it. You kindda started it so what has happened and what's the problem you are trying to solve? |
| P6 | 0:00:27.9 | [REDACTED] |
| Interviewer | 0:04:01.9 | Awesome, cool, thanks P6. My first, more substantive question, I guess then, is how does your organization define success, what's your end-game? |
| P6 | 0:04:19.0 | Yeah. Again, this is a thing that's changed over time as we think more ambitious. Because it started out, you know, I thought it was gonna be [REDACTED] And it turned out to something that will be much bigger opportunity, but one important feature for us is that there have been a lot of different use cases and then on different health issues and in very different countries. So, some core use cases right now are (0:04:55.2) care and immunization and general care coordination. And there're different metrics for this different health issues. The high-level goal is to have [REDACTED] users in the next decade or so. And that will be in a variety of countries. We now have projects in about [REDACTED]. And so, kind of when... There is a whole (0:05:32.7) some countries will be able to work with ministries of health and really take these types of projects to scale nationally and also to be able to continue providing great resources for, kind of, smaller projects at an individual organization or "reach them all" sort of level. |
| Interviewer | 0:05:58.2 | So... Other than... Those are some awesome and very ambitious scale goals. With so many different use cases, how do you... What are your up come goals, I guess? And how do you track those across, you know; with the organizations you're working with all have such different orientations themselves? |
| P6 | 0:06:23.9 | Yeah. It has been work like the nitty-gritties of it, it's on a project-specific bases for us. And it is, part of, because the organizations are very different and so they have their own expectations and levels of technical capacity to do their own monitoring. One of the core challenges that we've really tried to address is that there's a (0:06:52.8) for an assumptional* field that... It's the technology that will have a certain impact and that we should, sort of, be responsible for knowing what the impact of the technology is. And then the task for routine monitoring, we'll just see how many people are using them. And our experience in the field, and also this is a big part of my research as a graduate student is about the complexities of how these technology projects work in practice. And some of the difficulties in understanding, kind of, a general or universal way, what the impact of a technology is. What are really these technology-enabled practices? For us, there are basically, like... We keep track of the things we've done or impute, like, so many people trained. And when you track out some indicators of use, so presented, what percentage of community health workers in this project (0:08:07.2) text messaged in the last two weeks. And then, where we can, and this part gets fuzzier for some projects than others, directly trying to tie that to the health indicators or things with good proxy indicators or outcomes. So like, number of (0:08:29.3), appropriately say (0:08:31.9) care appointments intended this month, number of immunizations received on schedule, stuff like that. So, yeah, that's the basic idea. to begin the use of technology for these kinds of projects, the big utter disgust thing is this, how come technologies are implemented but not really used. Or used, kind of, half-heartedly or used really successfully by some staff other than people you've trained. And so often the challenge is trying to work out the (0:09:18.7) and how the project is designed and then also doing retraining, things like that are needed just to get effective use of the tool, to be satisfactory high. |
| Interviewer | 0:09:36.3 | Good. What types of... You referenced a little bit in you answer, I guess, some research and measurements that you've been doing. Specifically... More broadly... How is the research agenda for your organisation set, you know, and who do you aim to influence or what do you aim to use that data for? |
| P6 | 0:10:04.9 | The main idea, the main way we think about it is just to improve what we're doing. There's a broader thought leadership agenda for the organization. And that is (0:10:22.7) with my research is concerned with, but that I wouldn't say is... Mostly it's like evidence-based, kind of, research that we want, you know, our thought leadership activities have been more around human -centered design and the idea that we can use technology to improve care for communities. And some of the high-level things about: "Okay, we need to think of these technologies as tools rather than a solution". Part of the interest in research process, just like what does the literature say about one of the best proxy indicators for successful (0:11:15.8) care outcomes. We don't have, you know, medical doctors on staff for the most part. So, figuring out how to get through the literature on that and pick the best proxy indicators, sometimes when our client will have different picture about what that is. That’s part of what we’re interested in and then when we’re documenting what has worked and what hasn’t, our clients and potential clients is really obvious audience for that. For us that also have to do with getting funding, like proving what we've done works. And so, one of the big questions for us is, you know, we can go somewhere and look at something and learn something that matters and is useful to us, but what level of the rig are constitute evidence that we can claim to have proved it to other people? It's kind of an ongoing concern. |
| Interviewer | 0:12:38.3 | What do you think would help you answer that question or what sort of things have you've been trying to do answer these questions? |
| P6 | 0:12:50.4 | So, for me personally, I mean, this is why I [REDACTED] I was feeling increasingly like we need to get better research and (0:13:27.6) to improve what we were doing and it was to significant extent to wanna be able to share what it was we were actually doing, because we could tell it was really quite different from how other people in our field were thinking about these technology projects, but describing that in a way and evaluating it vigorously enough that we could share that effectively, more broadly with something that we just didn’t have the capacity to (0:13:59.9). We've tried doing several research projects with a consulting project group (0:14:06.9) students and, you know, talk to the professors that are in our city about this or that project and haven't had great success in managing projects that was some sort of one of the common problems. And I put together a slide guide, kind of, research we tried internally and common problems and lessons learned, that would be helpful for you to look at. |
| Interviewer | 0:14:50.7 | Yeah, that is very helpful for sure, please. |
| P6 | 0:14:53.5 | Cool, I'd be happy to share that. One common outcome is just like, we can't find someone who's interested in studying what we wanna study or... You can always find a student who is interested in studying that, but then do they have any skills that are any different than ours, how much time do our staff spend, kind of, updating them, so one common problem is that you feel like you've only... Feel like you've spend so much time kindda handholding them to introduce themselves to an industry they're not familiar with and by the end of the project we could have just done it ourselves and the whole point it was that we didn't have time to do it ourselves. And in some projects it was like a finish and they'd be like: "Here's the recommendation". And we just think: "no, that's not what it is." We're just not convinced that you've grasped the complexity of what we're doing here. That's part of it. And then, one thing that have come up in other areas which was relevant here was I found repeatedly that it's extremely hard to hire someone to do something that you don't know how to do. And you have to have at least a passing level of competence in a field, like when you enter a new field the first level of basic competence is to be able to understand who's better at it then you. And when you really don't know what you're doing, you can't do that. And so, for things like hiring a good accountant to, you know, software developers or whatever. I needed to just do it myself for a long enough to get a level of competence where I get obsessed who is gonna do really competent work for us. And I do, you know, now having bridged for a couple of years between academia and our organisation, I feel much the same way coming in the other direction, like, I have a pretty clear sense of the academics who just don’t know what it's like doing global health work in the kinds of settings we work. But it takes some, you know, person with enough experience or expertise in both areas to make those assessments. |
| Interviewer | 0:17:42.5 | Yeah, fair play. |
| P6 | 0:17:45.4 | Kind of a concrete problems, like how do we find the right partner if we were to hire someone internally, that's still a problem, like how do we know who to hire, what are the measures that someone is competent for research, it's gotta be really relevant to us. What are the funding mechanisms for this kind of work and do we wanna go with professors and do they have career trajectories that enable them to do that kind of work that's extremely (0:18:25.1) to us or do they have really different demands? One of the things that gets talked about a lot is that you don’t wanna pick anybody who is too famous, because they'll, you know, only publish in certain journals and have certain standards of rigor for which, as practitioners we feel there's really serious diminishing returns and we'll derail our organization. So for example, like the expectation you and I have talked about before this. If it's a randomized trial, let the intervention be set when the trial begins. And you have to study a single intervention throughout the course of the intervention period. That's really a norm, you know, of rigor in some, you know, in many circles. And for us is just (0:19:17.4) to a rigorous design process where interactions of different standard of rigor. |
| Interviewer | 0:19:29.9 | Do you guys face a lot of resistance, or do people or funders or ministries or anything, look to... You know, they put pressure on you for those types of rigorous designs or is that pressure coming mostly internally trying to cope with those things? |
| P6 | 0:19:52.4 | So, lot of our clients would love to see more research. Like...so they will have heard of the land set*(0:20:06.7) but they won’t know beyond that. sort of the difference between...it might be PLOS-medicine; one of the big five journals; or it might be…Global Health Science & Practice, or Science & Policy. and most of our clients or partners administrate our health and stuff; will know that those are dramatically different journals in terms of the standards of rigor that are necessary to get published in them. Few people understand...if you’re talking about the difference between the clustered-randomized trial and one that’s just randomized, and why you might need to do cluster randomization. Nobody...either statistically or at a conceptual level...it’s not wide spread to know what those differences are and why it matters. in terms of rigor, we haven’t figure out how to apply for a funding, to do dedicated research. so part of our struggle is always trying to bootstrap a little bit of research on top of what is...like implementation funds, which just...it’s a tight budget, cause we have implementation to do with that. And for example, one of the workshop sessions, they added a big retreat at our organisation. And what’s the difference between monitoring and evaluation? And that was something that I think was really helpful conversation for us. It was like, a lot of light bulbs going off-people had really thought through that before. Where evaluation is more like a study, and you might include a control group or you might do another, some kind of method for controlling, for alternative explanations. And with monitoring it’s just sort of widgets in-widgets out; like that. And realizing that the widgets in-widgets out stuff aren’t research in the traditional sense. |
| Interviewer | 0:23:00.3 | Do you ever do any of these education conversations with ministry folks, or clans? Or is that mostly an internal conversation? |
| P6 | 0:23:23.0 | I know these conversations happen. Since I started focusing on research, I’m a lot less client facing than I used to be. So I know those conversations happen, and that’s like the premise of why I’m helping other people (0:23:43.0) point of view about this, but it hasn’t mostly been me. but it is the kindda...you know I kindda really been focused on dissertational work, especially for the last couple months, and now I’m getting to a point where I’m thinking about initiating new projects that are more form tic than for my dissertation. And thinking a little more long term about what are the potential current trajectories for me; like if I want to just work at our organisation in a research field, what are the funding streams available? Will I potentially have good access to NIH type funds for the kindda research that we wanna do? Or am I gonna have an affiliation with the university, the department? And still what is the 10 year hurdle look like? [REDACTED] And that sort of thing... so yeah a lot I’ve learned, and a lot I’m just giving...still have left to do. I think the ideal scenario is that...if you look at any major tech company; they have an internal RND department. And justifying funding for that it’s just not complicated because they make a lot of (0:25:26.4). And figuring out how to justify an internal RND departments at Global Health NGO’s I think its super important. there are some great role models, like partners in health* and I think the way that they’ve done a mix of supporting observational research, some randomized trials, and some really great kindda far reaching, social science and contributions to how we theorize this field, really it’s great. But there are so many role models like that. So it’s still kindda exploring. |
| Interviewer | 0:26:33.6 | If you guys...like what questions do you think you still have to answer? What are the most pressing questions that are on your minds right now? |
| P6 | 0:26:43.1 | I think for us, what would be really helpful it’s for this kindda...to emerge outa this conversation would be if there were sort of...are you familiar with the principals for digital development? |
| Interviewer | 0:27:02.6 | I’m not. No. |
| P6 | 0:27:04.9 | It’s a consensus statement. It’s one of those things that a bunch of organizations have signed on. [REDACTED] Big players in the field... I signed on to...about design principles and it’s great for us because it just really super legitimatizes what we’ve been talking about since a long time ago. It would be great to see something a kin to that for lain* research. I don’t know exactly what that means and there is some difference between traditional science-as its traditionally practiced and the lane* where iteration is and engagement rather than independence is sort of the main thing. so (0:28:03.4) without that skill, but I think it’s really exciting and both to you and Fred. I’m inclined to think that you do have enough of a foot in the practitioner and the academic worlds to come up with something that would be a good balance, and that we can say, "here’s this sort of most efficient click and dirty* study design that we can explain to our clients, people we're working with. And that it would actually get published in the journal that people will take seriously and get funders." we'll take seriously... ’cuz I’m less worried about clients and stuff taking it seriously if it’s a peer reviewed journal. But funders...so we can create career trajectories for people. That would be really cool. I don’t know exactly if that’s a consensus statement, or it’s a report, it’s a tool-kit of some kind. There’s an article, you’ve might have come across the toll farmer*0:29:11.8 co-author; a couple of clinical trials and global health equity. I mean I just love the poets’ auto*(0:29:22.5) it was spot on. If there was something kind of in that tradition, but with a little more detailed how to that we can say, like we did it....in keeping with this group, that be amazing, that be really great . And then...the other on going question for us, that I think we've got a lot better at but there are no clear answers is how to prioritize the relative uses and application of more scientific research where we wanna evaluate a particular intervention and more process oriented research. right now it’s just basically me doing the process oriented research, ‘cuz I think it’s important and I like it, but I’m getting to point now where I could build up more of a research team around that, and just figuring out how to integrate that and how to fund it. Basically those are the questions. |
| Interviewer | 0:30:50.2 | Neat. Oh sorry last question then. I want to respect your time and keep this to close to half an hour but...and [cross talk]I’m asking one last question. I guess one thing we didn’t talk about when we’ve talked about research was the possibility of harm. Do you guys think about ways in which your program could be harmful or attempt to measure that in some way? |
| P6 | 0:31:20.1 | Totally. That’s an area where I’ve talk a lot internally and also in a more public form like [REDACTED] I’ve really promoted observational research because with technology use and really focusing on how many different ways technology can be used unexpectedly or produce unintended consequence, or not being used at all when it seems very intuitive that it would be used. The number of variables affecting any one of our projects is dramatically higher than the total number of projects. And that’s the kindda situation in which most social scientist take, "Hmm, you might wanna do a case study." low in type studies. If we had full funding, like ethnographic research methods, like on site interviewing and participants observation in particular, are pretty well integrated into how people practice human centered design. and we never have much time for that as we like, and in particular people will kindda understand that we can justify that at the formative stage, but there’s wide spread, we use it...design it’s sort of separate first stage that comes before implementation rather than they’re co-evolving processes that need to...design needs to go continuously alongside implementation. so I think it would be great to have people who study and work practices observationally, go back and revisit every project that he points...someone who's somewhat independent from the designer and who has training in that kind of research method, to go back to other projects. We don’t have the funds right now. Or its kindda internal and external...at conversations and education around unintended consequences, but that’s about as far as we've made it so far. |
| Interviewer | 0:34:09.6 | Good. That’s appreciative. Thank you. Great. Now like I’ve said I appreciate your contribution in our larger discussion, and I definitely wanted, and was looking for, to sometime to chat. Just sorta wanna know about your challenges and ideas for them. like I said I want to respect your time and I don’t know if there’s anything else you wanna add, to tell the people at school, and in an ideal world you’d be doing X, but otherwise, yeah. |
| P6 | 0:34:45.7 | Yeah. Well, I wanted to mention just one piece of reading material that might be helpful. Design is one of those tricky words, because it gets talked about by so many different people at so many different meetings, that it can be hard to trace what's what. And still a lot of my research has been about...I’m picking the intellectual foundation and [REDACTED] of the contemporary practice of human center design or design thinking. [REDACTED]. And a lot of what he writes about is designers, but he's also writing about clinical psychiatry and about the practice of management and then design as it plays out in engineering and architecture. He writes very eloquently and really popularized talking about this dilemma of rigor or relevance. his argument is that there are issues with how we theorize practice, and if we have norms of rigor that are about detachment or independence from practice then we risk making this gap with rigor or this dilemma of rigor relevance more severe. I mean this has been extremely helpful for me in thinking about...like I use his work heavily in my research and there are also parts of it where...he's like a foundational thinker in the current igorative* 0:36:53.9 approaches that led to what we now have in design thinking and also in leam*0:37:00.7 which are also an agile software development. They’re all in a big category of methods that are iteration based. And he writes about on the spot experiments and iteration is kinds of rigor that are different but not necessarily less rigorous than the kinds of (0:37:25.0) that we can produce through objective distance, independence from the phenomena of study and kindda the application of logic. |
| Interviewer | 0:37:38.6 | That sounds phenomenal. I have to get deep into that actually. |
| P6 | 0:37:43.0 | Totally. I’ll send you the link and so for me in an ideal world we’d have outlets where we could write about innovation processes and I really like to see when people are doing implementation that they write about much as you published a study protocol that there’s something along the lines of implementation protocols. That that also be a topic of (0:38:21.0) like documenting how did the...not just what were the outcomes of the implementation, but how did we do the implementation, and what kinds of rigor can we built into that? And then any kind of consensus statement that would come out of a school health form group....can be incredibly powerful around... “Okay here’s some rigorous observational method that is here.” Here are the ways that you can implement controls without disrupting the course of implementation and some things like that, that we could latch on to. That would be really helpful. And then really its figuring out the true interjectories. [REDACTED] |
| Interviewer | 0:39:31.9 | Great. No small task. |
| P6 | 0:39:36.4 | Not at all. well I’m super excited to see that you’re doing this because it’s a thing that’s been a huge issue for me, and more so it’s like a necessary foundation to the research I’m doing for my dissertation for our organisation. But I think it’s amazing that it’s a more explicit research project for you and I think I’ve become part of your dissertation that the fantastic...i really wanna read it. I hope it goes really well. [REDACTED] |
