# Supplementary material for: Supporting Better Evidence Generation and Use within Social Innovation in Health in Low- and Middle-Income Countries: A Qualitative Study
Source: PLoS One. 2017 Jan 26;12(1):e0170367. doi: 10.1371/journal.pone.0170367 (PMC5268497; doi:10.1371/journal.pone.0170367)
Supplement: S1 Dataset — (ZIP) [file pone.0170367.s002.zip › Data/Data - Interview transcripts/P7.doc]

| Interviewer | 0:00:00.9 | The first question is just to set the scene. If you could tell us briefly, in your own words to know what does your organisation do, how did you get involved in the org. and what's the problem that you are trying to solve? |
| --- | --- | --- |
| P7 | 0:00:15.6 | [REDACTED] |
| Interviewer | 0:02:49.7 | That sounds great. |
| P7 | 0:02:51.7 | [REDACTED] But there is so lot of work to do. And we see from the lot of the research and from a lot of our conversations with people within the global health community when we were starting out, part of what the gaps are includes, sort of, what we refer to as the talent pipeline. And that especially for young people that there is with this need for not only opportunities for young people to be working in global health, but to be filling the gaps that are needed and to be able to figure out a way to be able to train and support as we recruit these committed, smart and brave young people into the field of global health. [REDACTED] |
| Interviewer | 0:07:23.4 | No, that's great. And, you know... [REDACTED] that's such a cool emphasis that your organisation has. |
| P7 | 0:07:51.5 | Oh, thank you. |
| Interviewer | 0:07:52.3 | As an M&E person, what types of metrics are you tracking, how does your organization, you know, quote on quote define success? |
| P7 | 0:08:03.2 | [REDACTED] we really think about impact and the short, medium and long term. Was looking at, sort of, short term impact such as what is the impact the fellow is having at placement organization, sort of, what has he or she done that year; how has he made a change and a contribution at the placement organization? And we get that information every year from both the fellow and the placement organization. So I’d say, that's part of the short term as well as, sort of, community building aspects of that [REDACTED] Their careers are gonna change and evolve and we really wanna be there with them providing the support and the networks and the experiences that continue to help them to be better public health professionals. So, we're getting back to that. You (0:09:35.1) like the short-term, what is that fellowship year will look like, but also have they been learning. What have we've been doing, starting to do a lot more network (napping) and so we're seeing at the end of the fellowship year what is his network and what does his training look like. And then I would say, sort of, medium and long term is thinking; medium is sort of, how engaged were they with the community, where are they and their public health careers, have they stayed in the public halls. And then long term is, sort of, where they have landed up, what sort of impact and role and influence are they having within global health. And to say in terms on method and methods how we're collecting this is, I suppose, a combination quantitative and qualitative... We do a fair amount of surveying of our fellows and our partner organizations. I'd say we're a relatively new organization, [REDACTED]. And so it's been really focused on the program in the sense of how our trainings are being absorbed and what sort of leanings are fellows getting from that. And then really making sure of our placement organization learning from those experiences, [REDACTED] As related (0:11:09.1) out of the start-up phase into a larger place within the NGO community, really seem to (0:11:15.8) beyond the fellowship year, you know, what are the ways that we should be continuing to be tracking and engaging and measuring our fellows as they complete their fellowship year and move into a variety of roles within the global health community. |
| Interviewer | 0:11:37.5 | What do you think your biggest challenge is with those types of metrics or answering the types of implicit... What impact would you like to answer, but you don’t think you've been able to yet or can, but with some trouble... |
| P7 | 0:11:57.2 | I think part of our mission is in talking about outlets, training and finalizing the next generation of global health leaders. And so, part of our impact is still yet to come. We need, sort of, another [REDACTED] years to say: "They've come to this fellowship, we continue to work with them and now, ten - fifteen years on this is where they're at." So I think, part of it for us is that we've been able to focus on this short and midterm, but we can set up the parameters of how we're gonna evaluate the long term. But we still have the long term yet to come. So I think that's part of, for us, is that we see and, sort of, let trough (0:12:45.2) different sets of process indicators and measuring the network and different pieces of what our alarms are doing now. We've been able to, in trough of a (0:12:55.2) that we did this passed year, how many fellows or how many alums are still working in global health and we had a 90% response rates that they're working in global health, which to us was amazing the fact that especially given that people come from different backgrounds, they have decided to remain engaged in [REDACTED] and more in public health. And to us is a great (interim) indicator. I think form a community perspective, we also promised a lot of nice survey; ask how many want to and continue to be engaged in our organisation, and that was that 98% percent. And I think, you know, for us, those are really great indicators of what we're gonna see ultimately from all long-term impact perspectives. 'Cause again, you know, sort of, this network peace, with the thought of being the more that you have relationships and have experiences and understanding and have gone through similar trainings. Whether thinking about being resource oriented or innovation or having a social justice perspective, the greater that we feel like as they head trough the careers, simply be able to tap into this network and make better and more significant change. |
| Interviewer | 0:14:21.1 | What do you say to folks that would... That might question rather stuff like some indicators that you pointed out or to resolve the selection bias, you know, bunch of people that want to work for global health are still working in global health. Like, (0:14:33.5), you know, counter-factual, do you guys think about when trying to say: "No, it's actually this opportunity that was transformative." Or do you feel like you can't say that yet; or maybe ever? |
| P7 | 0:14:47.3 | So I think of something that we have some initial data and ways that we've been tracking that, but then i think there's more that we can do. So, for example, we compared our finalists [REDACTED] and so we track downs through LinkedIn, through all the finalist [REDACTED] and compared that against the fellows who were selected and completed. And that’s where we also saw 90% of [REDACTED] continue to work in public health. Whereas all the 70% of those finalist who weren’t selected on working on public health. So that for us has been one indicator that we're continuing to capture year after year, after year. We fill shows and we used to (0:15:52.9) that we think have attributed to our organisation. |
| Interviewer | 0:15:57.7 | Great, interesting. That’s a cool methodology you guys are experimenting with there. That’s awesome. That’s the impact on the fellows and obviously their impact on the world of global health care. if you attempt to subtract post fellowship. But then in terms of...you were also talking about...during the fellowship and their impact on the organization they're serving and working with, and the patients or people within that organization as working. Do you guys...watching for that and even in general; do you guys ever consider the possibility of harm in your programs? Plus something that your program could actually be harmful, despite best intentions? |
| P7 | 0:16:49.1 | Yes. clearly wanting to do well, and i think it’s also part of the reason that how we're structured the way that we are and the fact that we're not setting up yet another vertical or separate organization, that we are having young people working within existing health organizations with partners who have expressly identified a need that a fellow should be filling, and sort of understand what it needs to be taking instead of follow. so i think for us it’s those relationships, that...because we're all grounded in the sense of knowing what is happening day to day and that they are the ones have needstellars*0:17:40.8 as i mentioned they're the ones that make the final decision on who comes to work with us; so that it’s not like: "Here’s your young person. Work with that, see how it is" but that they really have the opportunity to make the decision and have that...be their own agency so that it can be more of a situation where together.” And this doesn’t mean that fellows don’t have challenges, and that (0:18:08.5) organization doesn’t have challenge. All of those things have them, but i think for us to...we want to continue to have...i very much prefer to our relationships with our partners is high touched and that’s why we're very also considered about scale and we have plans to jump to [REDACTED] fellows in the next 3 years. Because we think it’s so important to make sure that we're working with the right partner, organization, and have a very rigorous selection process and similarly that we're selecting the best and the brightest and those committed and the ones who stand the best for the needs of our organization. |
| Interviewer | 0:18:48.8 | What types of things go into that; choosing of partners? What sort of thing do you guys look at in that process? |
| P7 | 0:18:57.6 | Yeah, great question. So there’s a variety of criteria. number 1 is organization that aligns with the our organisation mission; we really want to be working with partners who understand the value of working with young people and bringing them in and providing them opportunities and giving them a real set of responsibilities. The second piece is that there really is an identified gap that a set of fellows could be...really being able to contribute and they get a meaningful difference in the organization within a specific work area. We’re really open to what that looks like but we also really want to make sure that it is a gap that’s needed. That’s another area. We also choose...our selection criteria based on the advisors, or not advisors, the supervisors. We have seen that some of the most successful partner relationships are ones that have supervisors that really get the laws that really do, provide time and clarity on the work plan. the work that those are doing throughout the course of the year; that the fellows are not just consultants, they're not just interns, but they really are staff for the year and have a supervisor who manages that. And that for us has been a really important factor. We also then look at, sort of more from the aggravator perspective in continuing to want to diversify the kinds of organizations that we work for. we think that, as important to be working with vast groups, organizations as it is with larger international NGO’s or within the ministries of health, or the private sector. And similar that also goes to the pros-health issues*. We had a large focus on wanting to expand and diversify. so that includes everything from nutrition to water sanitation, to reproductive health, to TB*0:21:00.1, malaria. because we think the more that fellows are having these different experiences and are coming together throughout their fellowship here and beyond, the better it performs and understanding that they are of how different aspects of health are related to each other. So basically that’s sort of another aspect that we look at. So yeah, those are some of the main key criteria that we look at from our partner organizations and then there are other things like finances, and some major policies and things like that. |
| Interviewer | 0:21:37.1 | Great. Okay. That’s great and sounds quite intensive and would take some time to do well. When you're answering the harm question, i know i just laying it on so way*. I apologize this is just for the purpose of the study we're doing at. It’s not a critique I’m just...the point is to play some pressure. so i guess the...one of the things you said gave you confidence about harms, is this rigorous selection process that you went through with the partners and the contact... that you keep up with them and the margin*0:22:26.3 that you do. What are the harms that might be inherent in the structure of the program itself? Like the fact that maybe you're supporting someone to do the finances for aggressive sort of organization, but they change it over every single year, because it’s a one year fellowship, and that ends up being (0:22:45.4) and maybe worse than some other outcome. Are there any thoughts like that. Like how do...is there any testing of the inherent, i guess the assumption of the program structure is what I’m asking? |
| P7 | 0:23:00.3 | Yeah. So i think. It will get harder than...is having conversations with our partner organizations. i also wanna say to that point specifically and how having those (0:23:17.1) and all the...we've actually we've seen an interesting situation where every year approximately 40% of our partners offering full time decisions to our fellows, and many of them take that. so we're actually seeing that...sort of this increased level of [REDACTED] fellows that are now full time staff. ‘Cause i can say too, our partners are not necessarily saying that they want finances for say year after year after year. And that’s why we really go through this selection process every single year, so that we can continue to see where the gap is. Maybe it still is in finance or money train* evaluation and they want to take the place...of having a new fellow take the place of other fellow. But we've also seen that number organizations have been able to expand the department that they had fellows because now they've been able to have their fellow, be hired full time and yet...and now upgrade another fellow for the further work out [noise] evaluation. so i think that it hasn’t answered of rigorously tested, but for us...we also feel and trust in our partners from the sense of, if they feel that it is being burdened to the organization, have a new fellow come in and it doesn’t make sense for them, we don’t have an interest in wanting to partner with them either. We want really to be able to be something that we are dealing fellows where they are contributing value and making a difference. Does that make sense? |
| Interviewer | 0:25:00.6 | Yeah, absolutely awesome. So part of it...i mean you’ve read the information sheet about this, and part of this research comes from the desire to help (0:25:22.4) school center and others, to help school fellows, up their game when it comes to really digging into their programs to make sure the outcomes are good ones, and also to continue tweaking it along the way. i guess...so two final questions. One is, who do you use, i guess that first one is a double-barrel question...who do you use...this information that you collect to influence? And what have you found to be most influential? Like what types of statistics? or what types of takeaways? I’ll start with that. And also a second… |
| P7 | 0:26:14.0 | Okay. (0:26:17.2) are evaluation outcomes, who we share the information with and for what purpose. [cross talk] okay. i say we have a lot of parties that are involved and engaged in terms of sort of see the evaluation. So i say number 1, (0:26:37.3) from a programmatic perspective. All of our trainings...we're always doing evaluations of what fellows are thinking and learning and getting feedbacks. so to your point of iteration*0:26:49.5 every single year we have continued to make changes based on their feedback. So for us it has been really critical part of all things, our Program to really be meeting the needs of our fellows. So i say sort of the fellows have been a huge party that evolved in our evaluation as well as part of the internal (0:27:15.0) for the programmatic team operation. So i say also, clearly our donors are big audience that...and not...we're currently doing our annuals sort of impact and outcomes given. Which is really for the summary of what has happened throughout the year, and what have the fellows been doing, and contributing and the result of end of year fellow survey; something that donors use. we also have a fair amount of more sort of...not full i say keen studies; but in terms of what our (0:27:51.4) are doing right now, so i say that’s another set of data, information that we often use for communication purposes. So to say, this is what...this are people who have bought long health corps programs, where they are now. so really sort of assuming and/or on more the first fall as are those of the broader. |
| Interviewer | 0:28:14.6 | Awesome. And if there was one ...maybe multiple things; basically just in a perfect world what would help you do this work better; this evaluation of your work better? |
| P7 | 0:28:32.2 | Well i have to say [REDACTED] has just joined our team and within the last month that is what i have been asking and hoping for in the long term. Because for us i feel like we have a lot of data, a collectible lot of data over the years and then it all...as possible odds of everyone in the organization and staff to take it and adjust it and make it useful for us and for donors. but now that we have a full time person, it’s so exciting for me to really be involved, for us to...the end to some of these sort of empty professions and look at more at sort of trends of what we've seen of all of it in our past six classes and be able to set off in a more standard politrain*(0:29:17.7) evaluation and really sort of doing a assessment, sort of, relook at everything that we've been doing from an avenue perspective. Because i feel that at the end of the day it’s about human resources to have people dedicated to doing this work, and having the time to do it. |
| Interviewer | 0:29:37.4 | Human resources. Maybe in your organisation for your organisation [laugher] |
| P7 | 0:29:43.1 | Seriously. |
| Interviewer | 0:29:48.5 | Wonderful. Well those were my questions, so i guess...just if you have anything...further you want to talk about or mention or for the record? |
| P7 | 0:29:57.6 | No, I mean, I’m interested just to hear in terms of like where are you are in the process? And what’s next? |
| Interviewer | 0:30:07.5 | Sure, absolutely, and so... |
