# Supplementary material for: Supporting Better Evidence Generation and Use within Social Innovation in Health in Low- and Middle-Income Countries: A Qualitative Study
Source: PLoS One. 2017 Jan 26;12(1):e0170367. doi: 10.1371/journal.pone.0170367 (PMC5268497; doi:10.1371/journal.pone.0170367)
Supplement: S1 Dataset — (ZIP) [file pone.0170367.s002.zip › Data/Data - Interview transcripts/P8.doc]

| Interviewer | 0:00:01.1 | Let's get started then. Just for the record, you think you can start by just telling us what you do, what your organisation does and what the problem you're trying to solve is? |
| --- | --- | --- |
| P8 | 0:00:15.6 | [REDACTED] |
| Interviewer | 0:01:57.9 | Great, thank you very much. This is wonderful, you've laid out a great overview of the way the organizations scale, in sort of reference, few of the trials that you are a part of have run. And I'd like to maybe dig into that a bit more. So, I guess, the first question is: What types of... How does the organization think about success, basically how would you define the impact? You mentioned scale, is it replicability*, is it community ownership...? What are the metrics you look at? |
| P8 | 0:02:35.0 | Yeah. There is a sort of winning which... I should, sort of, generalize that. And there is a specific set of impact indicators that we use. Let's go to the specifics first of all. Here I'd like to refer you to page [REDACTED] of our... of the [REDACTED] impact report. And if you haven't got that, it is available like, (0:03:05.6) copy. But the impact report... Our impact has four key indicators. The first is access to treatment. The second is reported, reduced symptoms. The third is the ability to work. And the fourth is the ability to take part in community groups.. Going back to "access to treatment", we started with a baseline, [REDACTED] So in another words, I mean, the baseline is basically before our organisation and then after our organisation does its work. And then there's taking part in community groups, (0:04:29.7) the relationship to the ability to work, but it also has a relationship to mentally people being able to advocate for themselves, foremost stand ability, if you like. That went from being very low, reported percentage of [REDACTED] % of people in community groups, and then after our organisation became part of their everyday life, they're now reporting [REDACTED] %. [REDACTED] And so, fruit from my point of view, we feel this achievement overall, is that a person for example, can live at home, can work and can take part in the ordinary festivities or celebrations of its own community. And probably take place in important events of which a very good example would be marriages or weddings (0:06:11.1) [low-pitched tone]. It’s not the only example, but it’s an example of how somebody might take part, then they were not allowed to take part before because of the (0:06:24.0) and the feeling of exclusion that the person (0:06:30.8) [high-pitched tone] (0:06:32.1) [buzzing sound] |
| Interviewer | 0:06:35.9 | So you have.... That's wonderful, thank you very much. It seems like you have a very clear sense of what you would like to accomplish and have some systems in place to measure that. In terms of these percentages, this is before and after data... Do you use counterfactuals in your work? |
| P8 | 0:06:59.9 | Can you just define what you mean by that? |
| Interviewer | 0:07:02.6 | Here we have an improvement over time that coincides with the introduction of your program. |
| P8 | 0:07:15.6 | Yes, the model. |
| Interviewer | 0:07:16.6 | The model. |
| P8 | 0:07:17.1 | More particularly, yeah. |
| Interviewer | 0:07:17.6 | So the question is, is that improvement ever compared to areas where that model has yet to be rolled out or wasn’t rolled out either in a randomized control trial or just how [overlapping speech] match pairs, anything like that, I guess? |
| P8 | 0:07:39.9 | Yes, we have some... Because obviously there's, such underdeveloped area, or put it another way, because there are so many mentally ill people in developing countries, low resource settings. Obviously we are wary of randomized control trials where some people, they never get the benefit of the work, if they were in the trial or control group. We always are interested in... We're particularly interested in trials where... Yes, of course, they can be randomized, but in due course, the control group also if offered the benefit of the program, you see what I mean? |
| Interviewer | 0:08:32.9 | Yeah. |
| P8 | 0:08:33.5 | So that would be, I believe, it's called stepped approach or something like that. I'm not a research man, but I believe that that* is how that kind of (0:08:44.2) is managed. And so, the answer is basically yes we do. We have a reading next to about twenty studies which have been pre-reviewed or journal-reviewed and which are written by some of my colleagues and little by academics joining force with my colleagues. And I'm happy to share that with you. |
| Interviewer | 0:09:10.5 | Wonderful, thank you. So, different question about this research that you carry out is: how it's carried out, what portion of it is done, sort of, in-house ( eminy*) and what does that look like and then what portion is done trough, you know, academic partnerships, how does that come about? Basically, how do you fund and organize your monitoring activities? |
| P8 | 0:09:45.7 | Yes, well... One of the things that I was astonished by when I came into the field which was [REDACTED] when I started the organization was the odata, particularly at that time, of course. And also I was given very reliable accounts by, you know, (0:10:08.6) people of the poor quality of the data that exist. Most of the data was, kind of, simple counting of numbers, in another words, you know, how many people are seasoned in a given country. And a lot of it was effectively on (extrapolation) of international norms. And so it was... And so really and truly, apart from studies which have been made empirically in particular countries in particular times, there was still (0:10:45.9) at that time. So it was important to build into the (omicks*) of the organization from day one, pretty much, a system of data collection which started... If we started the organization in 2000, the basic collection system fit into place in about 2002 and was by that time beginning to be operational and certain objective was (0:11:14.8), so it was to make sure that we knew we were on course and we were reaching the people that we plan to reach and that we were able to report that for donor, for manuscript (0:11:28.9) purposes. That was the first week of collecting data. And robust stage has certainly proved to be very effective in that context. What we then, of course, realized whilst the data was came to be valuable to research colleagues; for research, for our own research and for research colleagues. And so, relatively early on about [REDACTED] we started to clean our data and begin... And the data has now gone through two cycles of improvement and cleaning and then improvement and collection. And the third cycle will allow us to collect data on hand held instruments at the point of discussion with the patient or (0:12:24.7) discussion with the community. At the moment data is collected on paper taken to the nearest office and then it is queued in and it is then sent to [REDACTED] where we have centralized our research capacity. And the research capacity is based upon [REDACTED] staff based in [REDACTED] and working full time on our data. So, realizing that we have important data, we then started to make partnerships and friends with a number of universities of which these are examples: [REDACTED] under a professor, [REDACTED] University where we have written a number of different handbooks and manuals based upon our data and based upon the scientific work of those of that university. Those books are on the website. Of course, variety of universities in [REDACTED], but particularly [REDACTED] There are others, but those are the... And those are the universities we mostly work with and the work is... Of course, it can be quantitated* in the sense of people can use the actual picture that we have collected and all go back and trace the data back to its source and then work with data there. But also, of course, it can be qualitatively* (0:14:17.5) and can be case-study based. We have produced an interesting case-study, for example, it was pre-reviewed by number of people and we shared the authorship with (0:14:28.6), he was a great guide in that occasion. And that was (0:14:35.8) published and you'll see that in the reading (0:14:38.2). So, we've been able to turn interest in a veracity of forms, but its true to say as we have become more social-scientific or scientific and more rigorous, of course we have depended (0:14:55.2) people who are known to, you know, who make that big career. And so, therefore we've gone out to a variety of different authors and researchers and some... We usually have the principle of one of our team, XXX would be an example, (0:15:15.4) would be another. At all, one of our program managers being one of the principal investigators in the particular setting, research set-up, and then we always have at least one or two other principal investigators who come from the field of academic research, etc. |
| Interviewer | 0:15:39.3 | Great. And those partnerships, that was your organisation approaching different universities, different universities hearing about your organisation and approaching you...? What was...? |
| P8 | 0:15:50.7 | That’s it. Both of that are true. And several other things have occurred which are interesting. So for example, I was at the conference where [REDACTED] We have been able to do a collaborative study with him in [REDACTED], where we have been looking at the Global Burden of Disease and training, you know, good people to collect the data and the (0:16:44.5) of our organisation that, of course, now this has been done by [REDACTED] itself. And of late, the World Bank, WHO, National Institute for Mental Health are all combining together to, basically try to influence the ministers of finance coming together in October and April at the World Bank and they are trying to put together appropriate and relevant paperwork to demonstrate the economic liability of working with mentally ill people and the need to be able to (0:17:21.9) treatment and rehabilitation to the lives of a large number of mentally ill people, having (0:17:30.0) to the treatment, particularly in low-income settings[REDACTED] You know, it’s a variety of (0:17:44.0) of research, part of base research, more academic research, but always obviously when the viewer is trying to bring about a better understanding of mental illness, particularly in their income settings. |
| Interviewer | 0:18:00.7 | Great. You mentioned this World Bank meeting and attempting to influence finance ministers vis-à-vis-vis (0:18:15.0) investments in mental health. Who does your organization aim to influence and in what way, sort of, is that changed over time from when you started, to now being quite a bit more established? If it has… |
| P8 | 0:18:41.0 | Yeah, no. I think it's a very useful question. The first point I think is, originally I think we were really seeking (0:18:59.0), what you would call "program evaluation". We were really seeking to influence donors who (infidentely*) included bilateral and multilateral donors. So that would be the obvious (0:19:14.5), and in the most obvious multilateral would have been the European Union. At first we were seeking to influence them. And of course, because we wanted to raise money from them, but actually, just as importantly we wanted mental illness, mental ill health, mentally ill people to go on to their collective agendas. And that has certainly proved to be successful in a sense that if you look at the all-party parliamentary group report on global mental health which has been published by the all-party parliamentary group on health, and the all-party parliamentary group on mental health, there are two of them, (0:20:01.6) report on global mental health. If you look at that and you look at the submission by (0:20:07.3), almost all of the money that (0:20:11.1) has spent on mental health, in the context of full society has gone to our organisation. Which isn't a lot, by the way, but it's what it is. And the only (0:20:26.6) research they have funded which is [REDACTED], based out of [REDACTED]. And [REDACTED] which is an acronym based out of [REDACTED] is that they make of this research investment, and we are one of the two NGO's on that documented piece of work. We certainly were able to achieve success and continued to do so at the level of funding and at the level of program evaluation. And then, in a sense, since you talk about purer research, we have also been able to now be part of evaluation, in the sense of academic evaluation, evaluation of... by academic and (0:21:16.5)... Academic and (0:21:17.6), if I can put it that way, to have assessed our work for its (infringing) merit, I think that's very important, in terms of laying down reputation. And then, finally but not least, the two streams of activity combined in us having the ability to now be able to contribute to the influencing of policy makers. And we have been very successful at influencing national policy makers, so there are number of instances where we have been involved in the influencing for or the writing of new mental health legislations and all new mental health program legislation. That means national program. So, either way, we feel that for now (0:22:12.2), we've been able to punch way above our weight, particularly in respect of data and it's management. |
| Interviewer | 0:22:24.0 | Do you have any thoughts as to why that is that you've been able to punch above your weight and your assessment, just given that there're so many other, even social enterprises recognized by (0:22:38.2) that have not had nearly, you know, haven't quite the same rigor to their outcomes, haven't had the same policy influence that you've had... To what do you (0:22:50.4) that type of success? |
| P8 | 0:22:53.7 | It's part being a personal thing, although that's only a... I would put that for modesty, but when it's part of an environmental thing, so the first thing is that our organisation is actually the ninth organization that I've started. It's the third one that I have myself directly operated, having started it. And I had become very conscious of the fact that in some of the other organizations that I have developed over the years and handed on to other chiefs and left in due course. I began conscious that the... It was not good enough anymore to, sort of, make general trains of success or general trains of the problem without being much more rigorous about the data. So I had a personal desire to build in this rigor which was then, in my... In what was then my next organization, other words our organisation. And also that was then... That wish was actually amplified by the fact that I became aware that they took us for poorly managed in this field. And I realized that it would give us a significant competitive advantage, which indeed it has done. And if you look at the... But first of all worth pointing out that the competition is not great in our field, in the sense of there isn't a lot, but if you look at the competition that does exist, they generally do not provide data in the same form, at least across the, sort of, three different chapters that I had illustrated to you earlier, pragmatic and financial, national and international if you prefer. And then coming to the environment, I think it is true to say that the data was poor when we started to do the work and so we realized that there would be a real advantage to the charity and as a result of that, a sort of social entrepreneurial drive and the, kind of, personal wish to be better that I had been before in other organizations and data management collections. Together with the poor landscape has contributed to this particular success. |
| Interviewer | 0:25:40.8 | If you had to provide some guidance to other organizations that have, for some reason, struggled to make this more of a priority or have not been this successful in attracting various partners or, you know, finding it easy to evaluate social interventions of the type yours is, what would that advice be? |
| P8 | 0:26:09.9 | If you are starting an organization, which of course more (0:26:14.4) are not because they are being recognized by one that has already started. But if I was starting, I would say: "Build in from the beginning if you can". If you have to do it now, then I think that you do have to accept that it's going to be costly, I mean, not excessive, but it's going to have a cost. And the cost is going to be mostly represented by manpower. Whether it's (0:26:43.1) of the basic comes two people that we employ in India and (0:26:54.4) actually, but it's really important to be able to invest in that. And give them authority, as third point. They have to have authority within the organization to collect data and to, in a sense, assume that the programs are going to provide the data. So, it's a (0:27:16.3) in setting up data collection, if the programs in the bureau don't accept that they have to make an effort to collect the data and provide it. So, it has to become part of the culture of the organization. And that's why it's helpful to have data collection done internally, as this thing from... As it were merely employing external data accesses or calculators or collectors. It's actually I think, if you want to build a culture of forth comers, then you have to build a culture of data collection. And that actually needs to be internalized into the organization. Although we are a highly decentralized organization, in the sense that, you know, there is a tiny headquarters in [REDACTED], it numbers no more than [REDACTED] bodies. And we have the majority of our assets [REDACTED] program. And we have our program developed in a unit based in [REDACTED] and our information technology based in [REDACTED] and our data management and evaluation services based in [REDACTED] It is still quite possible for (0:28:49.3) to be able to collect the data in [REDACTED] from around the world and she has an excellent relationship with the program management staff in the twelve countries I already mentioned. That she goes to have those relationships, only understanding that she has the authority and the responsibility to collect the data and to require it. And I think that's being a really important issue. There were sometimes resistance in the past and they have had (0:29:25.7), have had fine ways of reducing that resistance and making sure that the data was produced. |
| Interviewer | 0:29:37.5 | You mentioned intra-organizational difficulties collecting data. Are there any... Ever any difficulties in these twelve countries as you've scaled? Say, where the public system has a way of collecting data for mental health already, but it doesn't provide, you know, perhaps the depth trough the scope that (0:30:02.3) has in mind, so they want to collect additional data, but then, kindda, place this, you know, some difficulty or burden on the practitioners. Are there any, I guess, countr*... your organisation in country operation conflicts around data with the rest of the health system in which its integrated to some degree? |
| P8 | 0:30:26.6 | Yes. Now I do think there is. The difficulties that we've had are just natural difficulties of program start. And this is in the early days, and it was really more in the context of answering your question about how would I advise others to ensure they have this kind of system. There are basically barriers which are barriers that resist in the (0:30:49.5) context and they wear down for the simple reason that it's: A: essential as a senior management to insist upon it; and B, it becomes extremely useful and self evident that data becomes really helpful, or in variety of reasons for the program management start. Now, coming to your question, the last question... Actually the same applies to both national and also local authority level data collection systems. Because generally the programs are run in a particular county or district or province and those authorities that are in those counties, districts and provinces generally do not have the (0:31:41.3) to collect data about mentally ill people. And so, very often we hear our own data being quoted back to us by officials in those countries. And so, quite often it is the data that we provided by our organisation, which is then used. We always warn our counterparts in government or local government to be careful not to extrapolate the data. You know, (0:32:12.4). Just because, you know, we work with "Z" number of mentally ill people in district "X", doesn't meet to say that that's exactly the same for district "Y". And so, it's important to give guidance on how to use the data. But generally it's appreciated, and of course, we always respect and use wherever possible, data provided by the government etc. It's an open... It's not an open source because of the need to be, to respect confidentiality, but we are open with our intentions to collect data for management and research purposes. And that's always part of the program understanding that we sign off with the government before we start. |
| Interviewer | 0:33:04.3 | Great. Listen, I want to respect your time and move towards wrapping this up. So I just have a few final queries for you. |
| P8 | 0:33:14.1 | Sure, go on. |
| Interviewer | 0:33:18.4 | The first is, when... I guess there's a bit of attention right in saying that this rigorous data collection allows your organisation to have competitive advantage, but at the same time there's a lot of... There seems to be a lot of education that your organisation does with ministry, folks, vis-a-vis. in the importance of data and how to use data. So I guess... Yeah, can we explore that a little bit? How important... How has having rigorous data made a difference with funders and ministry, or to what extent has... Have those people had to, I guess, be educated to understand the degree to which, you know, your collection is different than someone else's data collection? And how is that played out? |
| P8 | 0:34:16.9 | So, of course... In seeking to influence policy makers, for example, to understand the overall... The likely overall number of mentally ill people in the country, maybe the number of people with schizophrenia of the number of people with depression, it could be by illness type, in seeking to help them to understand that. And then in the next breath seeking to help them to understand the adequacy of their own response to the government, we would use data as means of backing up the argument, as distinct from producing data as an argument in its own right. So the data becomes meaningless, unless is being contextualized into a policy advocacy process. I think that's an important point to make. And so, we are responsibilities (0:35:28.0) to make sure we have rigorous data collection. And all of what I have already discussed with you is external responsibilities that are to make sure that governments look after mentally ill people, in other words, their mentally ill citizens promptly. And so, the data then, in the sense, comes into (0:35:48.3), and that's where it's really helpful to have studies. For example, we have a study done in [REDACTED] showing the effectiveness of our model and our approach. One of the things that are really important here is that... And don't forget that although the organization is important, what's become more important is our product, which is our model, the XXX model. And that's the central rehabilitation product, in the sense. Now the importance here is that it's very easy for policy makers to find themselves comparing apples and pears, so when they are looking at costs for treatment, they often think that costs which are related essentially or which are approximate essentially to the patient rolling out to an (outpatient) hospital, to an (outpatient) department at the hospital and seeking maybe four or five minute diagnostic treatment, and maybe being put on a pharmaceutical, on pharmaceutical treatment of some kind. Where of course, with us it's a five module model and that assures that the mentally ill person, yes of course, seeks treatment as they need it. But that they also move through a variety of different processes, which allows them to understand what the illness is, it allows their parents or their loved ones to understand what the illness is. Which means that they often belong to a self-help group; about [REDACTED] % of our patients (0:37:37.5) or in self-help groups, which means over 200.000 people. And of course, allows them to become involved in earning, particularly a lively hood option which suits them and which is, you know, part of the lively hood options that are open to the rest of the community. So, they have a normalizing effect as a result of that. So, costing, of course, can be different. If you're costing a simple trip to the clinic versus the cost for the person of the whole of that model, there is a need for a lot of education of policy makers and for them to understand. In the one example just going to the clinic, often the mentally ill person goes there and sits on their ass and is basically stigmatized as much as they were before. And in a system of rehabilitation, the person makes social as well as medical improvement. And therefore, the stigma begins to clock away. And so, the cost although a little bit more, actually then begins to be much more valuable, both for the person, the family and the country. With something like 2.5 trillion dollars being taken out of the global economy every year with mentally ill health, it's very important for national policy makers to understand the potential loss of their sustaining; particularly given the mental illness is often noticeable in younger generations. So, unlike dementia, which of course, generally comes at the end of life or towards the end of life, mental illness, schizophrenia, depression, anxiety disorder... often come at the early part of life, the late teens and early twenties? And that's, of course, just when people are becoming most productive. And to have as much as 6% of your population going down with those, with a combination of those illnesses, it's really very (detrimental) to economy. And those are the kind of our arguments, backed up by our data which makes the most difference to policy makers. |
| Interviewer | 0:39:57.1 | Thank you. So, final double-barreled question... Then we can call it a day, really appreciate your time. And perhaps the answers are related. Firstly, have you ever thought about ways or do you measure ways you're being, your organisation, in which the program could be harmful? And are there any questions and maybe that's one of them, that you don't think you've actually been able to answer with your evaluations and do you have any thoughts about what might help you do that evaluation work better or at all? |
| P8 | 0:40:45.5 | It's true to say that, on the first question, that not everybody stays in the program and that is typical for all development programs. And there are a variety of reasons for (0:41:00.1). Our dropout rate is quite modest and usually comes before the person has settled to their diagnosis and made efforts, either spoken or pharmaceutical treatment, made efforts to go through the treatment or where the treatment is not particularly effectively rendered. That would be one of the reasons. (0:41:34.7) several of the reasons why people drop away and perhaps one of the most important reasons when (0:41:41.7) I should say, when the program could be harmful is if the government in the partnership, cause it is usually the government, is not able to provide the treatment options that are absolutely necessary for the diagnosis that have been identified. And it's particularly harmful, for example, if people go on treatment, let's say, if they have schizophrenia and they go on treatment for schizophrenia, and then the supply of drugs run out because the government provider has gone out for a month or something like that. If that happened more than once, twice at the most, it actually means that the person can get more ill, rather than be (0:42:31.2) as they already were. And that, of course, is very prejuditional* to success from my point of view, as from the point of view of families in question. Of course, we work as having (0:42:47.9) experiences in the early programs, we, of course, work hard to make sure that that doesn’t happen. But it can happen and those would be good examples of alarming intents of detrimental impact. If we studied, by the way, in the sense of be understand we know about (0:43:10.7) and we do mitigate the risk of it by holding stores of drugs, with them have to be donated to the government, who can then prescribe them. We don’t generally hold with the believes that we should be prescribing drugs ourselves, all that we should be providing diagnosis, because we fear if we do that, then their very great dependency will fall apart, as we'll not be able to execute effective (0:43:39.7). Coming to your second question, there is an almost infinite number of different ways in which we could study our work better. And one of the simplest ways would be to mine, as in data mining, would be to mine our current store of data, in a partnership arrangement, so that we were aware of all of the different parameters which the data had. And that would be an effective start, it seems to me. There're also real opportunities to be able to understand how our model, our product, our model for mental health development has done in different settings. There are some excellent examples of people going back and meeting self-help groups, groups of people that we've put together, six years later in one case, six years later after the program has exited and still finding resilience and sustainability in the form of those groups still operating. So, there is some anecdotal information, which leads us to believe that it would be very interesting to go back to where we have exited from to understand how the work has endured and if so, for what reasons, and if not, for what reasons. So, there is plenty of work to be done, which I think would be useful. And perhaps the most remained to us right now would be to have a mutually agreeable, internationally recognizable way of describing the returnable investment, or value for money. And of course, we've got a lot of data which... And some efforts of that... But it would be good to get that, kind of, into a more globally accepted format. And though we're not alone, I won't think that, WHO is seeking that number of, you know, responsible bodies are interested in that kind of social return on investment and return on investment kind of discussion. |
