# Supplementary material for: Supporting Better Evidence Generation and Use within Social Innovation in Health in Low- and Middle-Income Countries: A Qualitative Study
Source: PLoS One. 2017 Jan 26;12(1):e0170367. doi: 10.1371/journal.pone.0170367 (PMC5268497; doi:10.1371/journal.pone.0170367)
Supplement: S1 Dataset — (ZIP) [file pone.0170367.s002.zip › Data/README.rtf]

Rough interview transcripts from a research project on barriers and facilitators of implementation research in LMICsCreators:Madeleine Ballard, BA, MSc* madeleine.ballard@gmail.com Centre for Evidence-Based Intervention, University of Oxford, 32 Wellington Square, Oxford, OX1 2ER, United Kingdom Jenny Tran, BMedSci/MBBS, MSc 	jenny.tran@gtc.ox.ac.uk 	The George Institute for Global Health, Oxford Martin School, University of Oxford, 34 Broad Street, Oxford, OX1 3BD United Kingdom Fred Hersch, B.Sc (BIT), MBBS, MPH 	fred.hersch@sydney.edu.au 	Saïd Business School, University of Oxford, Park End Street, Oxford, OX1 1HP United Kingdom Pamela Hartigan, BS, MPH, DPhil pamela.hartigan@sbs.ox.ac.uk Saïd Business School, University of Oxford, Park End Street, Oxford, OX1 1HP United Kingdom Paul Montgomery, BA, MSc, DipSw, DPhil paul.montgomery@spi.ox.ac.uk Centre for Evidence-Based Intervention, University of Oxford, 32 Wellington Square, Oxford, OX1 2ER, United Kingdom Funders:Skoll Centre for Social EntrepreneurshipProject Title:Supporting Better Evidence Generation & Use Within Social Innovation in Health in Low- and Middle- Income Countries: a Qualitative StudyProject Dates:April 2015-March 2015Kind of Data:TextualType of Data:QualitativeResource language:EnglishBackground and Objective:While several papers have highlighted a lack of evidence to scale social innovations in health, fewer have explored decision-maker understandings of the relative merit of different types of evidence, how such data are interpreted and applied, and what practical support is required to improve evidence generation. The objectives of this paper are to understand (1) beliefs and attitudes towards the value of and types of evidence in scaling social innovations for health, (2) approaches to evidence generation and evaluation used in systems and policy change, and (3) how better evidence-generation can be undertaken and supported within social innovation in health.Methods: Thirty-two one-on-one interviews were conducted between July and November 2015 with purposively selected practitioners, policymakers, and funders from low- and middle- income countries (LMICs). FILE LIST:Documentation	•	README.txt - Introduction to the dataset and methods description	•	Filename key.txt - Explanation of transcript filename codingData	•	F1.doc - Interview transcript 1/32	•	F2.doc - Interview transcript 2/32	•	F3.doc - Interview transcript 3/32	•	F4.doc - Interview transcript 4/32	•	F5.doc - Interview transcript 5/32	•	F6.doc - Interview transcript 6/32	•	F7.doc - Interview transcript 7/32	•	F8.doc - Interview transcript 8/32	•	F9.doc - Interview transcript 9/32	•	F10.doc - Interview transcript 10/32	•	F11.doc - Interview transcript 11/32	•	F12.doc - Interview transcript 12/32	•	M1.doc - Interview transcript 13/32	•	M2.doc - Interview transcript 14/32	•	M3.doc - Interview transcript 15/32	•	M4.doc - Interview transcript 16/32	•	M5.doc - Interview transcript 17/32	•	M6.doc - Interview transcript 18/32	•	P1.doc - Interview transcript 19/32	•	P2.doc - Interview transcript 20/32	•	P3.doc - Interview transcript 21/32	•	P4.doc - Interview transcript 22/32	•	P5.doc - Interview transcript 23/32	•	P6.doc - Interview transcript 24/32	•	P7.doc - Interview transcript 25/32	•	P8.doc - Interview transcript 26/32	•	P9.doc - Interview transcript 27/32	•	P10.doc - Interview transcript 28/32	•	P11.doc - Interview transcript 29/32	•	P12.doc - Interview transcript 30/32	•	P13.doc - Interview transcript 31/32	•	P14.doc - Interview transcript 32/32
