# Supplementary material for: Supporting Better Evidence Generation and Use within Social Innovation in Health in Low- and Middle-Income Countries: A Qualitative Study
Source: PLoS One. 2017 Jan 26;12(1):e0170367. doi: 10.1371/journal.pone.0170367 (PMC5268497; doi:10.1371/journal.pone.0170367)
Supplement: S1 Dataset — (ZIP) [file pone.0170367.s002.zip › Data/Data - Interview transcripts/F1.doc]

Interviewer:	I guess the first question is just could you tell us a little bit about your foundation and what’s the aim, what’s the vision, problem you’re trying to solve?

F1:	It funds organizations that have a scalable solution to the basic needs of the very poor and that includes healthcare and other health interventions.

Interviewer:	And how do you select those organizations or assess the potential scalability of their solution?

F1:	We recruit them through our network so we don’t take any proposals or anything. We go and find them. And when we find them, we give them unrestricted funds, and scalability for us have to do with whether the organization has an intervention that is cheap enough and simple enough and adaptable enough to go big having identified who the real implementer or what we call doer is at scale. So, we look at how somebody intends to take what they do to scale and… whether we think their model can actually go that route.

Interviewer:	When you talk about your network, do you mean [Y] or other sort of…?

F1:	I mean the [X] network. They’re not appreciatively different. [Y] is just kind of the funder side of our overall foundation network, but mostly we have our portfolio organizations of our fellows, alumni who really give us good, new leads.

Interviewer:	Is that lead-giving, so to speak, systematized? Like do you ask for that when you have an organization that you fund and they give you a report, do they have to fill out something about “here’s some cool folks doing something in our area” or it’s more just an organic thing that happens?

F1:	It’s the latter. Nothing’s systematized.

Interviewer:	Okay. When you get these organizations and you are assessing what they’re doing, do you look at particular outcome variables? Are you looking at financials? Are you looking at organizing? What are the key, the bucket so to speak of what you’re assessing?

F1:	Well, all of the above. We’re looking for is a scalable solution in terms of impact and an organization that can deliver it, so we’re looking at whether we buy the model in terms of an analysis of the behavior they’re trying to change towards the impact that fulfills what we’ve identified as their mission with that, and then looking at things like financials, looking to see whether the business model, whether it’s a pure philanthropy business model or a revenue-based business model, whether they’re going to be able to support the impact generating activities with the business model. A lot like a D.C. firm at potential investment.

Interviewer:	What types of evidence do organizations usually provide to you when you’re going through that vetting process? Are they giving you… process indicators, outcome indicators? Are they giving you randomized trials? Are they giving you some sort of lucky studies, vignettes? What’s typical?

F1:	Well, what we want to know is that they have a systematic way of measuring impact that fulfills the requirements of measuring the right thing and getting high correlating numbers and making a case for retribution and that can look different at different stages of the organizations. We think of organizations as going through typically a set of stages, namely the idea stage when there’s nothing on the ground, an R&D stage when there’s work on the ground, trying to figure out what the model is, proof of impact stage where the model is stabilized and we’re seeing if it really has promise to have impact and then early replication and scale up and as you go further toward the right-hand side of that graph, you get more and more rigorous in terms of real proof of impact and we think that the RSCTs really should be happening when you’re getting ready to scale up and they’re kind of a clumsy tool for early stages where you need quicker kinds of trials.

Mainly, we’re interested that organizations have an impact measurement and process measurement and behavior change measurement, that it is incorporated into operations over time and that we’re getting a movie of useful information rather than occasional snapshots.

Interviewer:	So on that organizational growth... spectrum, you guys would typically be funding folks just at the time when a catalytic grant could help them scale. Whose funding them in the earlier more grassroots stages? Do you follow that or take any look at…

F1:	We probably would be characterized as funding more in the early stages, but what’s a little unusual about us is we’ll fund from the beginning through indefinitely, so we’re happy to fund anywhere along the spectrum to scale, the path to scale. In fact, we like to be able to do the system. There’s just not enough stuff that goes to scale really.

[REDACTED]

F1:	We don’t see any endpoint in that offing to that relationship.

Interviewer:	We’re speaking with some folks as you know from whatever school health network in so far as that’s a thing and some of the people that we are talking to that your foundation funds include various health organistions. Are you able to break down where those organization were at when you found them and where they’re at now?

F1:	Yeah. Just through the list, so a health organisation... had completed a project with good outcome data. This was kind of unusual—they had good outcome data but as an organization were very young. Another health organisation, we got involved with them when they were really doing work on the ground that doesn’t look at all like what they’re doing now. Another health organisation was actually working at scale when we got involved with them. Another health organisation was well along the way but wanted to do some specific work on redesigning their model so we got involved with them because of that. Who else did you have? Who did we miss there?

Interviewer:	A health organisation.

F1:	Yeah, I just talked about that health organisation.

Interviewer:	Sorry. This other health organisation I think is the one. Yeah.

F1:	Yeah. I think we were involved with that one from very early on. I don’t know… the first couple of years of its existence.

Interviewer:	And once you select an organization to fund, how do you manage grants over time?

F1:	We have an annual milestones process. We identify, like, 10 priority milestones with them through the year ahead in terms of implementation and impact and organization-building and we use those to follow, to check in over the year, and then to make a decision in the year to come and when we’ve done a couple cycles of that, usually we’ll do a multi-year grant with annual reviews… and we go to the field quite a bit so we see most things every year or two as well.

Interviewer:	How did your foundation develop that milestone process?

F1:	Basically having a growing portfolio and a small crew, we just tried to figure out what is the most... and a general philosophy that we didn’t want to hassle people, that just sort of evolved into the most efficient and effective thing we could find. It really developed over some years.

Interviewer:	And you mentioned vis-à-vis impact, your expectations of an organization obviously change as that organization ages, grows. Is that reflected in the milestones, that they change? Is that sort of… and in what way or is that more I guess in your qualitative assessment of that organization?

F1:	You look at your milestones of somebody and you might say to them, “I don’t know why you’re being so timid this year. It seems like you should be a little more ambitious” or “We think you’re trying to take way too big a bite here and you’re probably going to bomb if you don’t get a little more realistic here.” It’s really a process of getting to know them and kind of helping them come up with milestones that are ambitious but realistic. And nobody ever fulfills all their milestones. You have to take it all in context [inaudible 00:12:21] war or drought or something wrecks everything.

Interviewer:	Right, right. Do you do any type of—as an organization—sort of aggregative cross-organizational analysis or is it really you’re focusing just on each organization by itself in a sort of one-to-one?

F1:	We do. Really, it’s following each investment we make separately, but we do kind of keep a state of the state of the portfolio in mind. I don’t think… No. We don’t look at our health portfolio and say, “How’s our health portfolio doing?” We have a sense of it. I’d say we have a pretty acute sense of it at any given point in time as we follow all this stuff so closely, but we don’t do a formal assessment.

Interviewer:	When you’re looking at the portfolio and you speak about the health of the portfolio, what does that mean? How are you guys defining success, impact?

F1:	Well, if you picture a graph of impact over time and you imagine you’re looking at a upsweeping curve, not a hockey stick per se, but a curve that captures that sense of exponential impact over time. We look at organizations and say, “Do we really think this could get there or not?” and if we don’t think it could get there anymore, we get rid of it.

And when we think of the health of our portfolio, we’re really thinking about do most of our organizations feel to us like they’re going to get there. In looking at them individually, does the aggregate start to emerge as we’re on the right track or is it cause of alarm? That’s probably the closest we get, but that... way of thinking about them has proven really useful because there’s a lot of qualitative and quantitative data that goes into that kind of Gestalt judgment about each organization and you go through a bunch of them and you realize we’re uncomfortable with a significant percentage of our portfolio, that would be worrisome.

Interviewer:	What are some of those gut-check moments or warning signs, danger signs, where you’re thinking, “This is not panning out the way we expected or hoped?” Is it different or are there commonalities?

F1:	Well, there’s some broad categories. One is they just couldn’t raise the money. Another is that the thing didn’t work. Another is that the scale-up idea is unrealistic. That’s the biggest problem in this sector because it’s a really inefficient sector so success doesn’t bring you the resources that it should and other organizations are not as… eager and willing to do a good job of implementing a high-impact model that they did not themselves develop. So, it’s just a super inefficient sector so all kinds of stuff happens that you really wish didn’t.

Interviewer:	When you speak about that inefficiency (and I definitely agree), but what would you say your role being on the funding side is in terms of agenda setting and in terms of attempting to overcome some of those inefficiencies?

F1:	Well, I think (1) it’s assembling the best portfolio you can and then (2) leveraging it with using your connections and position as a funder. In an inefficient market for impact or a complete absence of a real market for impact, that’s about all you can do.

Interviewer:	So…

F1:	You’re really trying to assemble the best groups you can, you’re trying to help them make the most progress they can, and you’re trying to leverage your funding for them.

Interviewer:	So there have been some groups, like in the UK for example, big government programs where they say, “We’re gonna find the 20 best programs for youth to make sure they don’t re-offend and we’re gonna set evidence standards in order to [pre-up Apria? 00:17:42] to determine what we mean by best.” There’s a famous one recently that said, “You’re gonna have to be measuring harms. You’re gonna have an independent RCT done on you. You’re gonna have to…” et cetera, all these metrics of organizational health. Does that sound like a good idea? If that doesn’t, what are other ways in which as a funder, as somebody who’s in that space trying to make sure the folks who are having impact are getting the funds, how would you create a market for impact?

F1:	Well, it starts at the funder level. It means the funders actually care about it and our attempt to rigor around it and commit to fund for it, and so the percentage who really do, are committed to it, is pretty low still. Even if people, those who care are trying, there’s not enough who care yet anyway.

Most organizations don’t effectively measure their own impact because the funders don’t ask them to and the funders don’t ask them to because they don’t really understand it well enough or it’s actually not in their best interests themselves to ask for it. It’s a really weird, weird culture around that. And then of course, there aren’t really agreed-upon norms. We have tried to create a system simple enough that it could provide at least a qualitative standard for what good impact measurement was, but that’s just little ole us.

Interviewer:	When you say it’s not in the funders’ interest, why would that be?

F1:	I don’t know if you can think of any foundation head who’s ever been hired for lack of impact, so why would you even measure it if it isn’t really that important? If it was determined you didn’t have impact, you’d get in trouble. In the sense that it’s not a norm, you might as well keep your head low.

Interviewer:	How do you think that could be made a norm? Or, at the least (sort of double-barreled question), what do you think would be the best avenue for say… You mentioned funders not knowing what to look for, for educating funders about different ways of measuring impact?

F1:	Well, I think the first would be to make funders accountable for it just like a venture capital firm is accountable to its investors for profits, so you just starting treating impact like profit and you try to figure out the best ways to create that analog and you make managers accountable for getting it. I think all the dynamics of risk and opportunity start to apply. This sector has simply been put hopeful money end and be unclear on what you get out.

Interviewer:	I’ve heard you talk about this and I’ve seen blog posts and discussions starting in a small way around improving this, but it’s been... And you see with some larger funders, like [inaudible 00:22:11], this other funder, they’re talking a little bit or differentiating at least between process indicators and outcome indicators and they’re moving slowly in that direction. What do you think accounts for that? Well, first of all, do you agree that that is a trend? Do you think it is but it’s moving too slowly, it is but it’s inevitable, it is but it’s only being held up or propped up by certain things that are happening that might not continue to happen? What’s your sense of... I hate this word, but the space vis-à-vis funders?

F1:	I think it’s getting better and better. I think the more people care and more people are thinking in smart ways about it. Of course it’s slower than we’d like, but there’s a lot... I know a lot more smart funders now than I used to and it’s not just because I know more people. It’s because there is a certain groundswell of interest in and commitment to impact and there are smarter people coming into this, especially as business people for example who made their own money and instinctually see that way in which profit and impact can be seen as analogs of each other and the same business approaches that can produce profit can produce impact if applied to effective organization by effective organizations with a good idea, so I think it’s getting better.

Interviewer:	If you had to pick something that hasn’t gotten better, that you’re not super impressed with, what would that be?

F1:	I think, in general, I’m not crazy about strategic philanthropy. I think that the notion of funders deciding what priorities are and trying to develop solutions and drive solutions is a much less effective, efficient way of making things happen than investing wisely and energetically in things that work and trying to get them scaled up. That just comes from somebody whose perspective is one of the investor camp and mainly because we don’t have the resources to indulge a more strategic focus, a more strategic approach.

Interviewer:	Do you think there are pitfalls to sort of the grand challenges approach versus a more your foundation style ‘seek what works and fund that’ approach?

F1:	Oh, yeah. I think that the grand challenges and the prize and all that stuff is largely useless and I’ve never seen any real academic work certainly to the contrary. I think the onus is on the people who push that stuff to prove that it’s really the best way to make things happen. I don’t buy it.

Interviewer:	When your foundation is assessing potential solutions, are there any ways in which you look for potential harms, like proactively look for potential in the context of a history of well-meaning programs that actually sometimes have unintended, unfortunate effects? Is that part of your vetting?

F1:	Sure it is and we have enough experience to know what some of the obvious traps might be, but also we take a behavior approach and what we’re looking for is what kind of behavior changes are you trying to create and are they likely to last, and I think taking that perspective, you end up seeing a lot of unintended consequences coming.

We also fund only people with really good monitoring systems in place so that untoward consequences, unintended consequences, should be picked up fairly early. I think at some point… I don’t anybody who has a foolproof way of avoiding unintended consequence. That’s why work should be iterative, but you look at some of the stuff that’s just had these terrible consequences like the whole scandal of mosquito nets and overfishing in African lakes and somebody just wasn’t paying attention. People weren’t really following... If you give out nets, you really should figure out a way to know what’s happening with those nets 2 years, 3 years down the line, and we really try to make sure that that kind of thing is happening.

Interviewer:	Last question. What would you characterize... You mentioned good monitoring evaluation system. How would you characterize those?

F1:	You’re asking the right questions about the key elements of delivery and behavior change and impact in that you’ve got a systematic, efficient information gathering, imaginative systems that are incorporated into operations and feed back into both populations an iteration of model and strategy, so you deeply embed it. Monitoring is not an external function block. It’s basically watching the temperature gauge and motor.

Interviewer:	Alright. Great! Well, our 30 minutes are up. I’ll just say thank you for letting me pick your brain for a little bit. It’s appreciated and just also give you time if you have anything you’d like to say vis-à-vis, “Hey, this is how we could be doing evaluations. This is how we could be communicating better” that’s on your mind that I didn’t ask about before we close?

F1:	No, I think you did a really thorough job. I don’t have any… burning issues that we didn’t get into there.

Interviewer:	Great! Super.

F1:	What are you trying to produce from all this?

[end of recording: 00:30:08]
