# Supplementary material for: Supporting Better Evidence Generation and Use within Social Innovation in Health in Low- and Middle-Income Countries: A Qualitative Study
Source: PLoS One. 2017 Jan 26;12(1):e0170367. doi: 10.1371/journal.pone.0170367 (PMC5268497; doi:10.1371/journal.pone.0170367)
Supplement: S1 Dataset — (ZIP) [file pone.0170367.s002.zip › Data/Data - Interview transcripts/F10.doc]

Interviewer:	Okay, F10, so just for the record, can you just state your name, position and organization?

F10:	Sure. My name is F10.I’m a managing director of our Foundation.

Interviewer:	Okay. And we’re going to start off broad first. I just wanted to know a bit more about your foundation. I have read up on your website, but just for the record on the interview, what’s the big mission at the your Foundation? What’s the big question that you’re trying to answer with a lot of the activities you undertake?

F10:	Our Foundation, we’re focused on finding funding, supporting early-stage social innovation at a very high level. We work both domestically in the U.S. and internationally. We have about a 50/50 mix, a broad set of issue areas: health, education, workforce development, poverty alleviation, agriculture, clean water, social justice, on and on.

What really makes us distinctive is the stage of growth with which we operate. We work with early-stage organizations so those are organizations that are 1-3 or 4 years of age at the beginning of a grant, half a million dollar budget U.S., plus or minus a handful of employees. They’ve proven a little bit of something programmatically, but they haven’t achieved any scale at all so we tend to try to help them kind of scale their impact.

Interviewer:	Okay, okay. With the social entrepreneurs or the organizations which you choose to provide funding and support for, how do you go about selecting each of these?

F10:	Our selection process is fairly robust and then we have kind of a rolling admission. Most our organizations get referred in from a broader network of stakeholders that we work with. But, kind of an open application—anyone can apply— so it’s a simple online application.

If you get through some initial screenings, we do three levels of diligence with our organizations. One, we kind of get to know the leader incredibly well. Our belief is that we bet on people first and models second. Second level of diligence is kind of getting to the team around the team, so that’s the senior team, the board, the funders, the formal and informal advisors. And then, third is what we call “outside attendant”: people that don’t necessarily know the leader or the organization, but actually have a strong subject matter expertise on the space that we’re talking about. Collectively, that can mean anywhere from say 25 to 40 phone conversations or some in-person meetings mixed in there.

It takes anywhere from two to four months, depending on how quickly we can actually have those conversations. Not a lot of people go the distance. If you do the distance, highly likelier that we’ll fund. One of the reasons why we’re doing the diligence is because it also helps us create what our three-year learning agenda and where we’re going to lean is, so we have a fairly robust metrics and milestones and learning agenda document that gets developed along the way so that we don’t start from Day 1 saying, “What are we going to do to be helpful?” We have what the roadmap looks like on Day 1.

Interviewer:	Wow! Sounds like a very, very robust process. … So you’ve talked about you get some leaders or organizations from referrals, from stakeholders, and then there’s also an open application process. Can you kind of compare and contrast what are the success rates for each of those entry methods into your selection process?

F10:	Pretty straightforward, actually. Things that get referred in—highly likelihood. If you just look, we’ve probably funded since 2002 somewhere north of 90 organizations and I think of which only two came in through over the transom, unsolicited. Not unsolicited, but like un-referred in, so the people who refer in to us tend to be early-stage funders, later-stage funders, academic partners, later-stage entrepreneurs—either people who have gone through our programming and have received funding from us or people who have not—[and] some policy folks as well.

So, we have a fairly active network that helps us develop good pipeline but also helps us during portfolio support and also during diligence.

Interviewer:	Okay. So for, say some of the open applications that you receive that haven’t been successful, what are some of the characteristics of those, like why have they not been successful?

F10:	… We’ll look at probably somewhere around 700-800 organizations a year that come and apply and we’ll end up making say 15 to 18 grants. We’ll get from 700 down to 200 pretty quickly for a number of reasons, a lot of which people apply that aren’t qualified. They’re either too late-stage, they’re pure research, there’s a religious or faith-based bent to what they’re doing, they’re place-based organizations only; they’re not looking to scale.

Again, we’re trying to work with not necessarily just the botF10 of the pyramid, but surely people that are disadvantaged so we’re not going to be funding cultural institutions, so to speak, so there’s a lot that drop-off because they’re just not qualified.

Interviewer:	Just not appropriate. Okay.

F10:	Correct. Yeah. Then, the drop-off is... It gets a little bit harder because we’re... If you qualify, we have a conversation with everybody so everybody has an opportunity if you qualify. Something that I think organizations are a lot of times too early-stage. They find us probably at the wrong time and we have a fairly intensive three years together and I think if you’re too early-stage, you can’t fully metabolize all that we’re going to bring and then it’ll be a painful grant for you, perhaps.

Interviewer:	I see what you mean so it’s also about protecting the leaders or the organizations who come to you as well, making sure that they’re getting the appropriate support at the right stage of their organizational development.

F10:	Correct.

Interviewer:	Okay.

F10:	And another one, why things will drop out, is just the leader and the organization have a systems change orientation or not. If folks are doing their work and they just want to do more of it but they’re not really thinking about, “Can I be a proof point for something larger?” or “Can I advocate for some type of policy change because of my work?” or “Can other people emulate what I’m doing and do I become a technical assistance partner?” Like, having an idea of what your impact is beyond just your direct impact. If you don’t have that, that’s another reason why you would drop out of the process.

You would also drop out of the process if the leader is... I guess “cultural fit” is the… There are a lot of people that are not coachable or they don’t have a learning orientation; they feel like they have all the answers. Or people who just want our money, but not all of what comes with being part of our foundation, I would say those are other reasons.

I would say probably financially if people are on their last leg and they’re having trouble either generating earned revenue for their product or service or if they’re having trouble raising philanthropic capital, again you’re not going to have the time to focus on all that we’re hoping to bring to your organization. If you’re having trouble raising money, you’re kind of in a little bit of a trap.

Interviewer:	Okay, so something that is often perceived by particularly practitioners on the ground in the space of health is that it’s often the support that you get from both funders and policymakers is based on who you know than what you actually do. Sometimes these kind of network referrals are perceived as a bit unjust by some organizations. Do you have a viewpoint on that?

F10:	It’s interesting. What I would say is if you are… It’s tough to kind of say “‘cause if you know one funder, you know one funder” ‘cause there are some phenomenal place-based organization where there’s, like, an extraordinary leader whose from a community who doesn’t necessarily have access ‘cause he or she didn’t go to the best schools, isn’t as well-traveled, but they’ve developed a damn good program that’s actually having a tremendous impact. So, how does that person get funding from your foundation? I actually have sympathy for people in that situation.

What I will say for our foundation, we are specifically not place-based funders even if you actually run the best healthcare organization that’s serving a population in [REDACTED] or something like that and you’re doing *tremendous* work and you’re growing the number of people that are being served by your program, we would not fund you. Not because you’re not a great organization, but you’re not in alignment with what we fund. We’re looking for people to kind of scale their impact, meaning go to another geography, create the playbook for other people to implement, be a technical assistance partner, advocacy, policy change, something like that.

… And some of them are some of the better organizations that are out there. But back to your question, is the deck stacked against certain organizations? Potentially, yeah, if they don’t have exposure or access to funders. How do you get in if you don’t know somebody? So I would empathize with some of those organizations.

Interviewer:	Okay. It’s quite interesting that you put such a big emphasis of this idea of scaling and helping organizations who are at that induction point where they’re kind of are quite robust in what they do already and looking to create system change. I’m wondering about your foundation. How do you guys define success and impact as an organization?

F10:	It’s kind of interesting. A couple different layers, but just on impact of organizations, we want all of our organizations to be thinking about 1) direct impact and 2) systems change impact. Clearly, you actually have to have it there programmatically and you’re using it as a proof point, but you’re also a realist and you know that your organization will not scale to solve the problem. But, there’s something that you’re learning through the impact that you’re having in your discreet area that can be leveraged by other organizations, other individuals, government, whatever, and we want you to be a global citizen in that regard.

So success for us is people that actually have the ability to have that systems change. … This is kind of a live conversation that we consistently have and depending on who we’re talking to, our fundamental intervention with our grantees is that we want to help our organizations have scalable impact and for that, the role that we play is we want you to be able to attract and metabolize later-stage funding.

And so, if you look at the components of our portfolio support, it’s in alignment with that so those are we help you build out a board, a revenue strategy, making sure that you have financial systems in control, making sure you’re using data to capture your impact, learning how to message “What do you do?” in English and not non-profit jargon so being an effective communicator, out of town at the right time, making sure you have the right work structure for your organization especially if you’re going to be pivoting from direct service to like TA or something like that.

Not any of it has to do with “Is it the right program?” We’re not going to tell you this is right dosage or the right delivery mechanism or whatever because that’s not where we’re an expert. We’re an expert in building businesses around program models. So in that regard, we’ve used success— “Are you able to attract later-stage funding or not? How do you develop the skills that are needed?” We’ve put out since 2002 somewhere around 40-45 million dollars and those organizations since we’ve left have been able to attract over like 950 million or something like that… but that only tells you one thing. You could also say, “Wow! You pick people that are actually good at fundraising.” Yeah, you’re right. Any of these metrics that I’m going to share with you are going to be, there’s holes in it to a certain extent.

… We want you to scale your impact so each organization, actually we have a small dashboard that has what the key performance indicators are and unfortunately you can’t aggregate them across the entire portfolio like you can ‘dollars raised’ or something like that. We’re seeing a 50% CAGR (compounded annual growth rate) on those key performance indicator so if it’s a certain population that you’re trying to change the outcome on, you’re actually doing that at an accelerated rate. If it’s reducing under 5 child mortality, you’re doing that again at an accelerated rate. So, we look at each organization kind of programmatically and impact-wise. Are you doing more of what you’re doing more effectively, more efficiently?

And then, the third area where we evaluate ourselves is on our portfolio support, like in those six areas that I mentioned we do a pre-during and then post testing in terms of... and again this is very you could say subjective. We do it and other organizations do it in terms of where are they—basically score 1 to 10. Where are they in terms of having a revenue model? Or where are they in terms of board development? Where are they in terms of communications or adding talent?— before intervention, during, and then after as a way to are we seeing… Are we helping build better running organizations?

I think if you just picked any one of the three, meaning like dollars raised, increasing impact and increase in organizational development, there’s holes in any one of the three, but we hope that collectively it’s telling a story about if we’re being effective or not.

Interviewer:	That makes perfect sense. It sounds like you guys do try and collect data on the more holistic picture and really do look at not just the funding side, but also the level of support and sustainability of the organization after you guys provide that structured support system. With this in mind, I think you’ve touched on it very briefly, but are there specific metrics that you use or you require your organizations to report in the follow-up after you’ve decided to provide them with support and funding?

F10:	Yeah. And each one’s kind of different but they kind of fall into a couple buckets. There’s a couple that tends to be… Programmatically, there’s two or three things that we look at. When it comes to financially, we’re always interested in the revenue mix and the expense piece.

Organizationally, like the size quality of staff, are we adding in the positions that are needed, and also organizationally the board development piece. We spend a lot of time doing a board map with organizations. What does your ideal board look like in terms of number of seats at the table, in terms of mix, skillsets and diversity and experience? Then, we kind of help organizations migrate from the board they have to the board that they need.

But it’s interesting— there’s not a universal set. We have the overarching programmaticals and metrics that we use: financial, organizational. When you get to the organizational ones, yeah, it tends to be like the mix of employees and the growth of the board. Financially, it’s obviously the revenue, the revenue mix, number of months of cash on hand. We’re always tracking the expense as well.

And programmatically, a lot of degrees of freedom there because again where we have so many different kind of issue areas that we cover, we really try to work with the entrepreneur for them to tell us what are the right programmatic metrics. We don’t want to be prescriptive there because again you’re telling us if you’re being successful or not. Not on outputs, but on outcomes, and what are the near-term things that we should be looking at to see if you’re on track to have those outcomes?

Interviewer:	I see. Okay. Given that, it sounds like there’s a lot of data that you try to collect from each of the organizations at various time points and this requires a lot of monitoring and evaluation resources as well as expertise. Do you specifically provide funding for monitoring and evaluation? And if so, how? If not, why?

F10:	A lot of what we were just talking about is really data that we capture on our own organizations; the lift for them is very little. We actually kind of take that on a lot ourselves. It’s again the information that they need to report on ends up being 8 or 10 numbers every six months, so it’s not a massive [thing] for them. Clearly, like upfront when we’re doing our diligence, when we’re kind of setting up the dashboard for them for sure, we hope that the payback is the 3-year grant.

And our money is unrestricted too. I think one of the things that kind of may get lost in there [is] we have a one-size-fits-all grant. It’s $100,000 a year for three years. It’s unrestricted. We take a board seat with every organization that we work with. For the most part, we’re on bi-weekly calls with our organizations to try and be helpful ‘cause again... and I’m on daily email traffic with folks as well, again around the organization build out and our hope is between the unrestricted capital and effectively getting a percentage of an FTE, that that’s worth their effort… Well, hopefully, if we’re doing our job.

Interviewer:	Okay, so I’m going to change tactics a bit and I’m going to ask a specific question. Sometimes particularly with early-stage organizations, the activities that they undertake can cause unintended consequences despite best intentions. At your foundation, do you at all think about how programs could be harmful and how that could be prevented? And how do you do this?

F10:	Can you repeat the question? I’m sorry.

Interviewer:	Sometimes organizations starting out can cause unintended harm in their activities. At your foundation, do you actively think about harmful practices and how to prevent them and how do you do this?

F10:	The unintended consequences on the population they’re serving?

Interviewer:	Yeah. Mainly on that, but other harms as well that are just not thought about at the beginning when they’re devising their model, when they’re piloting their model.

F10:	We try not to be punitive. Obviously, we do a whole lot of research on an individual to make sure that they’re being altruistic in what they’re doing and they come at it with the best of intentions. If there’s a challenge that they’ve created, we’ll try and help them navigate through that challenge.

In fact, actually, we view ourselves as successful if we are the first or second person they tell about it. We’re really sitting on the same side of the table with our grantees. We’re not there with the “I gotcha!” question or to pull funding if they’ve done something wrong. However, because we sit on boards with all these organizations, if there is a problem, we want to make sure that it gets eradicated quickly in the most effective way.

We don’t kind of abandon grantees because they’ve made mistakes. Social entrepreneurship is just that— it’s testing things. It’s innovation. It’s trial and error. And sometimes, you don’t get it right, especially if you fund the well-intentioned, well-traveled Westerner going to the developing world. A lot of times they’re going to screw things up because they don’t have local context. I think what we learn from that is making sure that the organizations that we’re funding in fact *do* have indigenous talent at senior levels so that you can kind of mitigate the risk of harmful activity just based on not having local context.

Interviewer:	I see what you mean. Okay. Yeah, that’s a really good point that you raise, like because you guys provide such close support, it’s ideal that every step of the way that organizations keep you updated. That’s not something that’s usually seen from the funder and practitioner kind of relationship, so it’s really great that you can try and enhance that relationship. It’s quite unique.

F10:	Well, we hope so, right? It’s interesting… Again, like I said, we view ourselves as successful if our grantees can be vulnerable to us as opposed to kind of hiding things from us and worrying what’s going to happen if they screw up. That probably comes a little bit from our roots, like our money comes from the venture capital community where you make a lot of bets and a lot of times things aren’t going to work out and that’s okay.

So, we try to kind of bring that same mentality to our organizations at the early stage. If they had it all figured out, they wouldn’t need our help, so again maybe this is where we’re a little bit different but we actually like to hear about the challenges and like to roll up our sleeves and be helpful when that happens.

Interviewer:	That’s great. We’re coming up to 30 minutes now and I did want to respect your time, so final question. In the perfect world, what do you think would help you do your work better with the your Foundation?

F10:	Internally or externally?

Interviewer:	Both.

F10:	Externally, it’s kind of an interesting one. If there was more transparency among funders, ‘cause I feel like a lot of the change that needs to occur is on the funding side and not on the grantees’ side. Funders don’t operate always in the best way and I don’t think it’s intentional. I think there’s a lot of bad behavior on the funders’ side: when they do single year and when they do restricted, when they take a lot of people’s time and make them jump through all kinds of hoops and then don’t use the information at all for anything. That’s something where I’d love to see funder behavior change.

I’d like to see kind of a funder continuum, like some organizations have more resources and it’d be great to see them fund at that later stage where it’s a valley of death once you get to be like a 5 or 8 million dollar a year organization. It’s tough to raise money because everyone wants to give $100,000 or $250,000 or something like that but no one wants to write a seven-figure check.

Externally, also, I would love to see governments once something’s been proven with world-class research to work, the governments would free up resources to actually implement what works very candidly. For that to happen, I feel like government needs to be involved along the way so when we’re working with our health portfolio, we make sure even in early days that they’re not running a parallel system to the government, in fact that they’re working in concert with the government, *even* if they don’t agree with them, because at some point in time the end game for health organizations has to be government at some level.

So, I would like to see governments operating differently, like if there are organizations that are proving to work and have an impact on health outcomes, that governments would free… Obviously, there’s a high burden of proof for government but once that proof point’s been made, that they would free up resources.

I kind of think internally… Sure. There’s a number of things that I wish we could do better. Do we need to be as robust in our diligence? Maybe, maybe not. Is there a way that we can kind of codify and be more efficient in our portfolio support? Yes, and it’s something that we’re actively working on. The six areas that I talked, three of them are well built out and we do a very good job of because we’ve honed it internally. The other three are things that we would aspire to be better at but reality is we’ll probably never hire a staff person or two or three to run that so we really need to identify those outside relationships and vet the vendors that can provide that world-class support, so we’re in the process of doing that right now and kind of piloting ourselves.

I would say lots of things to kind of comment on there. … But I do think the biggest change on the philanthropic side, I’d love to see organizations also not prop up organizations that are underperforming. I think it’s okay to let organizations pass away or kind of fold into other organizations. I feel like social problems are huge in nature and larger institutions are what is eventually needed. I still think there’s a role for innovation to kind of push the thinking at the larger institutional level, but I don’t think a small innovative organization needs to be the majority. I think it needs to be in the minority.

Interviewer:	Okay. Wow! That’s a lot of stuff that needs to happen to make your ideal world.

F10:	Oh, my ideal world? I could keep going then.

*[shared laughter]*

Interviewer:	No, that’s perfect. F10, thank you very much for the interview. It’s been an absolute pleasure talking to you. Really, really enjoyed hearing your perspective and hearing some of the reasons behind what your foundation does and how they go about doing it. It’s been absolutely fascinating so thank you very much, F10.

F10:	Awesome! And, it would be helpful like at the end of all this... and I bet you’re probably doing this anyway, but I’d love to see what you come up with.

[end of recording: 00:31:18]
