# Supplementary material for: Supporting Better Evidence Generation and Use within Social Innovation in Health in Low- and Middle-Income Countries: A Qualitative Study
Source: PLoS One. 2017 Jan 26;12(1):e0170367. doi: 10.1371/journal.pone.0170367 (PMC5268497; doi:10.1371/journal.pone.0170367)
Supplement: S1 Dataset — (ZIP) [file pone.0170367.s002.zip › Data/Data - Interview transcripts/F11.doc]

Interviewer:	Alright. So, F11, just for the record, can you just state your name, position, and organization?

F11:	Yeah. My name is F11. I’m the social entrepreneurship portfolio director for our Foundation.

Interviewer:	That’s great. I just wanted to get a bit of background about the your Foundation. I have looked at your website and I’m real excited to be hearing about it from you, but can you give me an overview? What’s the big problem that you’re trying to solve in your Foundation?

F11:	Well, we don’t have one big problem. It’s a family foundation and we have three portfolios—each with their own unique hypothesis. We have a local portfolio that’s focused really grassroots in East Palo Alto which is the neighborhood community from where the family grew up, and within that portfolio, we’re really taking a grassroots approach to education so working with the various schools in East Palo Alto in the Ravenswood School District as well as the school superintendent, figure out what the needs are within the educational system.

And then, we have two other portfolios. One is focused on [REDACTED], as well as a global portfolio that is focused on the developing world and those two portfolio we take more of a venture philanthropy approach so we make early-stage investments in social entrepreneurs with innovative, scalable ideas, and specifically we look in the fields of education, livelihoods, and then in the global portfolio, in health.

We’re really looking to investment in organizations that can scale up—grant investments—so it is similar to venture capital in the sense that we’re looking for the next big thing and making higher-risk early-stage investments but there is no return. They are grants and so our return is really the organizations that can replicate their model and increase their organizational size and breadth of scope such that they’re serving hopefully thousands if not millions of individuals.

Interviewer:	Okay. So, a question about the organizations in all three portfolios that you end up funding, can you tell me how your Foundation selects the organizations to which you provide funding for?

F11:	Yeah. The criteria is different across local versus our regional and global portfolios, so the local portfolio is really working in partnership with the schools and the school super to assess what the needs are and what the opportunities are to serve those needs.

Within regional and global, we do much more of a top-down assessment so we’re looking at the social entrepreneur. Are they dynamic? Are they capable of harnessing resources? Do they have a compelling vision? We’re also looking at their proposed solution. Is it scalable? What is the end-game of that solution at scale? Does the organization have the potential for long-term sustainability? What is the impact on the end beneficiary? Is it a compelling story or is it just incrementally changing things? So, we’re looking for transformational impact in a end beneficiary’s life.

And then, we look at whose funding it, what’s the potential for future funding. Is it always going to be reliant on philanthropy or do they have an earned income component or an ability to partner with other organizations or with government at scale to really pay forward or subsidize the model at least so it’s such that private philanthropy plays a smaller role?

And then within each of the portfolios, we’re looking at the sub-themes under education, under livelihoods, to understand or to get an understanding of how innovative or and how game-changing the intervention might be.

Interviewer:	Okay, so it sounds like you take quite a comprehensive approach in trying to look at organizations as a whole in selecting which organizations to eventually fund.

F11:	Yes.

Interviewer:	In your view, what do you think are the more important criteria? What do you place more emphasis on?

F11:	I think it really is on the entire organization because we are making unrestricted investments, so we’re investing in the organization not in a specific program or programmatic outcome. The most important things for us really are that entrepreneurial leadership both in terms of that visionary leader and a strong leadership team.

And then, program delivery that can really go to scale, that can make systemic change and/or be replicated directly with ease such that the organization may be small when we start to fund it but that it has a potential path to affect millions of lives.

Interviewer:	So just in that point—creating scale, creating systemic change, something that’s replicable and with a very clear potential pathway, what kind of things do you look at to assess how things are going to be scalable?

F11:	Well, we’re looking for the organization to be able to articulate that path to scale and we’re looking for evidence that the model is scalable, so simpler interventions that can be of a reasonable short duration and that’s relative to whether we’re talking about education, health or livelihoods, but something that is a quicker, more transformational intervention such that the organization can then move on to serve other people, and just what they’re doing around partnerships or how they are testing what they think the pathway to scale is, so even organizations that are relatively young, are they piloting interventions with government or are they setting up their model such that it is a compelling argument for government to become the payer? Are they experimenting with other people, doing the work such that they can move to a more technical advisory or other non-profits can adopt the model? What are they doing to assess how this might look like at scale?

Interviewer:	I see. Okay. So as an organization, as your Foundation, it sounds like you have very clear criteria in choosing the organizations you fund and then also thinking about their potential. How do you guys define success or impact as an organization?

F11:	Success is funding organizations that eventually can leave the nest, so to speak, organizations that grow into sizeable, sustainable organizations impacting thousands if not millions of lives.

And then in terms of how we define impact, it’s such a loaded term. We let the organizations themselves define their impact. *Our* impact is something that we’re still trying to figure our because we don’t have our own specific theory of change within a programmatic ethos so it’s not we’re only funding organizations that are trying to get access to college or only organizations getting at agricultural livelihoods, so our impact is so much more blended across different themes. Fundamentally, we are hoping that our role can be catalytic in contributing to organization’s longer-term scale as opposed to having the attribution; we’re focused on the contribution.

Interviewer:	I completely see what you mean. Okay. So, once you’ve selected organizations which match up to what your criteria are, how do you manage the grants?

F11:	We have different stages of funding so we have early-stage funding or pilot funding where we’ll do a one year grant and see how the relationship develops, see how it resonates within our portfolio, how the relationship develops with leadership and how it just fits within the our family because we’re still very much a family foundation so there is that intangible feeling and fit piece.

And then we do from there multi-year funding to help organizations achieve their proof of concept and that can be a minimum of three years and then go year on year out from there for a fifth and six year. We’re actually in the middle of finalizing—or close to finalizing, I should say—our third stage of funding which is growth and replication for organizations in the portfolio that have achieved that proof o concept and are now articulating a roadmap to really scaling and replicating it and getting to a new threshold of organizational sustainability and programmatic impact and that will be another four year commitment but of a more sizeable nature, kind of more Mezzanine funding.

Once an organization’s in the portfolio, we have a very grantee-centric philosophy, so how we define that is really trying to level the playing field between the non-profits that we’re funding and the funders themselves so we don’t have a proposal process. We use a lot of existing materials that are created internally for organizations or proposals that are created for other foundations, and similarly on the reporting side, we ask for existing materials as opposed to creating things just for our Foundation, and then we engage in dialogue, both with the non-profit and then internally to determine where we need to focus more of our attention or get additional information and we use that to make assessments on the organization going forward.

Interviewer:	Okay. It’s quite interesting that you use existing materials that the organizations have already, which of course is intended to reduce the workload that the organization has in terms of meeting requirements, but do you have any specific reporting requirements? … It sounds like it’s a constant dialogue between you guys and the organizations you work with to try and figure out a good in-between.

F11:	Yeah. The organizations, we will work with them to set organizational milestones for the year and those are holistic milestones so they look at program delivery, any augmentation to program design, expansion, organizational health and HR key hirers, fundraising, and the organization themselves sets the milestone. There can be anywhere from 10 to 20 milestones that they’ve set, but we try and have them set smart goals and work with them to make sure that it is a really 360 view of the organization.

Then when we check in with them, we ask to check in on those milestones, so we ask for any written information they might have so that we can deepen our understanding going into a conversation but then that conversation is guided by those organizational milestones and it is really a dialogue around how things are going so that we can get that broader perspective of what’s working, what’s not working.

We can add value in areas where things are maybe more of a challenge and it does, on the whole, inform our funding over the longer term, just in terms of how successful the organization’s been, have they been able to progress their vision, and where we see our role, ‘cause again we see our role as being very catalytic so if the catalyzing factor is not there either because the organization is just not executing well and it’s a bottomless pit or at some point the organization has hopefully gotten big and mature enough to leave the nest so to speak, those are the types of conversations we have internally to see what our appetite for future funding is.

Interviewer:	I see what you mean. It sounds like with the milestones that you set with the organizations, you try and have this constant dialogue where each of you are putting in your perspectives. Do you provide funding specially for evaluation and monitoring activities and if you do or you don’t, why?

F11:	Our funding’s unrestricted so if they want to use it for that, that’s great. We want to see them doing monitoring and evaluation and that is what we’re looking at is organizations that intrinsically want to measure their impact and be able to articulate that. We feel like that that is now required by the field to scale and it’s just a healthy habit to have to make sure that you are having the impacts that you want to be having or at least that you know what impact you’re having.

In terms of why we don’t fund it specifically, it’s just because our ethos has been around the organization knowing where to deploy their capital… best for highest return on investment and sometimes it might be through increasing their monitoring and evaluation. Sometimes it might be through hiring. We want them to be able to deploy effectively.

Interviewer:	Okay. We’re going to change track a little bit to a bit of a different question. In the health space, particularly with early interventions, sometimes despite best intentions programs can be harmful to the communities that they’re serving. As an organization, have you thought about ways in which programs could be harmful and how have you thought about that and dealt with that?

F11:	Well, within the health space, we are really focused on the distribution of health services and goods so in terms of the harm aspect we haven’t spent a lot of time thinking about that. Certainly as we look at organizations and talk to them, there is a higher level assessment of their overall benefit and so harm would be rolled into that, but we don’t specifically get down to a granular detail to see those negative cause and effects. We’re betting on the organization and the smart organizations take that into account so we don’t want to want to butt in and to kind of overreach in terms of what our own vantage point is. We’re hopefully funding organizations that are net impact positive but certainly recognize that there can be certain unintended negative consequences.

Interviewer:	Okay. So, F11, just wanted to back-track a little bit. The question that I wanted to ask you specifically is can you tell me how you go about identifying organizations to assess against your criteria? I noticed that you don’t take unsolicited referrals. I’m sorry. You only take referrals, but you don’t take unsolicited applications.

F11:	We don’t really have an application so if people come to us unsolicited, we took a look at them for fit, but the probability of fit is typically low based on what we’ve seen. Typically it’s the referrals from known foundations and known agents who we’ve spent a lot of time discussing things with and they know when organizations hit our sweet spot. Does that make sense?

Interviewer:	Yeah. One concern that is often raised by some organizations is that without already being familiar with people in the funders’ space it’s difficult to get into that circle where you can get a referral. How do you get this? How do you find those solutions that could be successful but they might not exactly have the networks or the access to networks?

F11:	I think part of our theory of change is really around that social entrepreneur and that ability to aggregate resources so there are compelling organizations but they lack the leadership, that visionary leadership and that dynamic leadership to really scale and replicate their model, and those types of organizations really aren’t a fit for us because we really are looking to make that venture investment that scales massively, so it’s a little bit self-selecting in that way.

I think we continue to look for avenues whether it’s accelerators or incubators so that it’s not just one path. We’re at this Forum and there’s a lot of opportunities to find organizations there. We’re looking at country-specific incubators and accelerators [REDACTED] and we’ll look at organizations that come out of that and maybe aren’t as polished as some of the organizations coming through other funders, some of the typical pathways to us. So, we’re expanding that, but ultimately, these organizations have to find their way into some sort of filter that’s looked at them and said, “Yes, this has massive potential to grow,” and then is doing that angel round of investing to get them in front of other organizations.

Interviewer:	I see what you mean. Okay. So, F11, moving onto the final question now. In an absolutely perfect world, what do you think would help you do your work with your Foundation better?

F11:	More people? *[chuckles]*

Interviewer:	Okay. What do you mean by that?

F11:	Well, I oversee two portfolios that has a total 30 to 40 entrepreneurs and I’m looking to add, so it’s just hard to have enough hours in the day to really look at every organization. I think that if organizations, the non-profits themselves get better at thinking about what their impact is and how they’re going to scale it and replicate it even if that replication is four or five years out, the more they can articulate that piece, the easier it is for organizations such as our Foundation to evaluate them.

And even if they don’t know, even if they’re looking at one of three options, the earlier they’re thinking about that piece and the more articulated they are in understanding their own impact, the easier it is for us to make an assessment on our relative fit.

Interviewer:	Okay, so more organizations thinking better about their impact and having a more forward-thinking plan to articulate.

F11:	Yeah, to do pilots, lower-risk interventions that then use data to kind of drive their hypothesis.

Interviewer:	Okay. Alright, F11. That actually brings us to the end of the interview. Do you have any comments that you wanted to add?

F11:	No, I think it’s great. I hope it’s the type of information you’re looking for...

[end of recording: 00:24:36]
