# Supplementary material for: Supporting Better Evidence Generation and Use within Social Innovation in Health in Low- and Middle-Income Countries: A Qualitative Study
Source: PLoS One. 2017 Jan 26;12(1):e0170367. doi: 10.1371/journal.pone.0170367 (PMC5268497; doi:10.1371/journal.pone.0170367)
Supplement: S1 Dataset — (ZIP) [file pone.0170367.s002.zip › Data/Data - Interview transcripts/F12.doc]

F12:	[REDACTED] We have around 15 foundations and those are only the foundations that work in developing countries so it could be the U.S. or foundations from developed countries but working for development cooperation or those could be the foundations from the South as well.

So basically, the network was established at [REDACTD] established three years ago here and the objective was really to create a platform for philanthropic foundations because there was kind of a recognition that foundations are becoming more prominent actors in development cooperation in general.

And the network, we serve kind of as the broker between the foundations and the government because if you say that the foundations are increasingly financing public policies and foundations are private actors, somehow those two need to talk more because this was not the case because historically, for example, many foundations had been working more in autonomy so they were not necessarily working with governments. … They were covering some public good that were not financed or covered by other actors such as governments, et cetera, so we are just like brokering today. We are brokering a dialogue and also facilitating partnerships between the two— between the governments and the foundations— and it takes place at the very global level.

For example, right now when you have all those negotiations leading to the adoption of the SDGs (the sustainable development goals, for example), so this time foundations and private sector were more associated to this process than it was against, for example, for the [inaudible 00:02:44] dialogues and this is something that we do as well. For example, we are having site events at this global policy dialogue event just to make the two talk to each other. This is at the very global level, so we are kind of lobbying for foundations to be included more in those kind of discussions, but then also we invite governments to consider more foundations even before asking them for some money. So, this is at the global level.

And then at the country level as well, we are trying also to connect foundations with government so we have some country pilots in several countries where we facilitate dialogue, [REDACTED] where we basically have at one table the government and the foundations in some specific sector.

For example, in [REDACTED], it’s about the child and maternal health, but that’s not the case for others, so basically first is a diagnosis of the relationship between the government and the foundations and I can tell you that even though it seems very obvious very often the two just don’t talk and they don’t know… like the government does not really know what the foundations are doing in some specific sectors in their own country, so it seems quite useful. This is just a few examples of the work that we do.

What I should say is that we are a sector agnostic in the sense that we also do a bit of research and publication so there were already two publications, but we are not working on any specific sector because our foundations they work on many different sectors. Before this conversation, I just had a look at the list of our members and I think there is, for example, amongst 15 of our members, there is only one who is very specific in health and maybe also I could put you in touch with them because I think it might be useful for you to talk with them. It’s the this Foundation. That is based in [REDACTED],, but they are working in developing countries on health-related issues.

Interviewer:	That’s understood. No, thank you.

F12:	Actually, I don’t know if you are also on the loud speaker because I cannot hear you that well.

Interviewer:	Okay. Here, I’ll just move closer to the speaker. Is that better? Or not really?

F12:	Yeah, a bit better. Yeah.

Interviewer:	Okay, cool. I will also try to speak up and speak clearly.

F12:	Okay.

Interviewer:	Just a quick point of clarification, when you’re speaking about these foundations, do any of these foundations do any implementation or they’re really all just people who distribute funds to grantees who then carry out some type of work?

F12:	So your question was, if I have this correctly, if those are operating foundations or are they only doing advance funding? Is that your question?

Interviewer:	Exactly.

F12:	… We don’t know. The majority of our foundations are advance funding, but I think that for example this other Foundation, it’s not really the case. For example, what they do, their work is really about finding novelty solutions for health problem in developing countries’ context and so very often or what they do is they actually take like months before they’re partnerships with the private sector, with the government as well.

For example, they have been working on a project of [tele-message? 00:06:52] in [REDACTED]. This project is quite well known today because there was a pilot in some several regions in [REDACTED], and actually today they are thinking of scaling it up to the whole national level, so it will be scaled up by the government because they’ve been working with the government from the very beginning and they built a national program.

So, it’s not really grants. It’s not really operating because they are operating the programs. They are not advance funding. They are setting up also different partnerships. Basically, you have very different types of foundations. You may also have, for example, but this is not the example of... Well, the example that they have is not in health. But, for example, you also have foundations who work a lot today on market-based solutions so supporting social enterprises or social entrepreneurs who have come up with some novelty solutions to development problems.

And what they are doing, they are kind of also facilitating and customizing those solutions. For example, trying to create a [inaudible 00:08:09] environment that would enable those people to create markets for the product or services that they propose. This is the case for this other Foundation so that’s why this kind of differentiation is... There are many different models right now for philanthropy but I would say that still [a] majority of the work is through grants.

Interviewer:	Okay. Typically, I think (as I’ve understood in the health sector) you would have implementers operating in a particular country and they would receive funds—potentially from foundations—and that practitioner would then be responsible for coordinating, especially because health is a public good in a lot of countries, they would responsible for coordinating what they’re doing with the Ministry of Health’s larger plan.

What then is the extra benefit of bringing foundations into contact with the Ministry or with the government more broadly? What was the problem to begin with and what have you seen since convening those discussions that’s sort of worked a bit better?

F12:	I’m referring to interaction, but I really cannot hear you well because I feel like I hear the background noises a bit... so I don’t know. Can I call you at some number? Like a second number? I don’t know if you’re using the loud speaker or not.

Interviewer:	Here. Just give me two seconds and I’ll try to add a microphone. If that doesn’t work, then I also have a U.K. number but that’s long distance so I’m not sure. *[brief pause]* Okay. Does that work any better?

F12:	Yes. Yes, I think so.

Interviewer:	We can see. Okay. I guess I was asking very basically practitioners typically are the ones, at least in healthcare, that meet with the Ministry and coordinate with the Ministry the various activities that they do with the Ministry’s plan. What is the added benefit of then also bringing funders into that conversation? What changes?

F12:	What changes? The fact that foundations are also in contact with government and not only practitioners. Is that your question?

Interviewer:	Yeah, exactly.

F12:	Well, I think it depends, but I think that when foundations engage with government and what I mean by “government” is the host country’s government… I think that often they will try to achieve some larger scale impact because basically it’s not only about… when it’s sufficient to work this government. It’s more like informing them about what this foundation is doing… If foundations engage with the government from the very beginning, I think in development cooperation there is something very important about the ownership of the government for the programs that are financed nationally, so I think this is something very important.

And then also, foundations are looking to kind of coordinate most of their programs with other [inaudible 00:12:33] whether there are needs for this specific program or also if there’s a like pilot program for some novelty solution, they will often look for the possibility to scale up. Once a program’s successful, then the foundation will probably try to scale up and this is something that foundations as an organization need to discuss from the very outset of the program and this is not only practitioners because… This is not only about their respective health issue, but also government and many others.

For example, the example of [REDACTED], is that they were also conversing with universities, with the private sector, so probably somehow they had the capacity to coordinate and facilitate a dialogue between the government and also other actors, not only practitioners, but others who are involved in a particular problem.

… I think it’s not the same thing, I would say, so it’s not the same objective. If it’s the funder who’s in contact with the government.

Interviewer:	Yeah, no, understood. I want to turn now to… Sorry, microphone. I want to turn now to some of the specific tools that your organisation has developed, say for example the philanthropy compass. Do you think you could talk a little bit about how that was developed?

F12:	Okay. Could you just tell me what do you mean by “philanthropy compass” because this is something that maybe we will develop but we haven’t yet or do you mean The Guidelines for Effective Philanthropic Engagement because compass is not yet something that we did?

Interviewer:	Sorry. You’re right— I misspoke. The Guidelines.

F12:	You mean The Guidelines for Effective Philanthropic Engagement or is it something else that you have in mind?

Interviewer:	No, sorry, the Guidelines. I misspoke. I’m looking now again.

F12:	Okay, but this was funny because I don’t know if someone mentioned to you the fact that we maybe will work on the compass, but… we haven’t yet decided and developed. The system’s still under our decision.

So, on planning The Guidelines for Effective Philanthropic Engagement… Just to put it very simply, I would say that this was an effort from the our organization to try to develop something, let’s say similar to the Declaration on Aid Effectiveness. I don’t know if you’re aware of our principle for the philanthropic sector. Basically, the Declaration on Aid Effectiveness— this was all the principles that were supposed to help with aid effectiveness for government, but those were not discussed and those were not signed by the philanthropic sector, by the private sector, et cetera.

So, our organisation went to add them here at the [REDACTED],, but basically it was just to put on the paper some very basic principles on how philanthropy should engage with the government in developing countries. You have to [inaudible 00:16:20] type with the [inaudible 00:16:22] group of principles around dialogue and around data so knowledge sharing and then third one around partnering with government.

So if you go through those principles, those are like the [inaudible 00:16:39] ones which basically said if you want a developing constitution talk with the government you should the dialogue. Second, you should exchange data on what you are doing, on results, on validation, et cetera. And then, third one, to [inaudible 00:16:56] possible you should coordinate with other actors, coordinate with them, [multiplication? 00:17:01] and subtract, et cetera.

This seems like a very simple thing, a basic thing, but as I said before, really this was not the case—at least not that obvious for the philanthropic sector because those guys just didn’t work together and talk together before. So, those are voluntary. Those are non-binding and an interesting fact may be that, for example, you will not find anything on accountability. I mean, there is some things on data, but foundations… Because we of course consulted those guidelines — those guidelines were consulted — and they opened a dialogue with many different partners, with many different organizations. Those were also consulted with other foundations and clearly there was some principle, for example, that it was not possible to put it in the guidelines.

So even those are voluntary and non-binding, those are very basic but I still think it’s a step forward in better effectiveness and better cooperation between foundations and government.

So, yeah, I don’t know if you have any other questions on how those were developed, but I’d say this is basically it and also maybe from political points of view it was important that those guidelines were... mentioned during the high-level meeting of the Global Partnership for Effective Development Cooperation which is like a platform, which is a continuation of the Paris Declaration talks and then there was another conference in [inaudible 00:19:00], in Busan, and this is in Busan in 2011 that people decided to create this global partnership which includes not only government but... I don’t know. (If you know what I’m talking about, you just [inaudible 00:19:14].)

This was basically validated during the high-level meeting in Mexico in 2014 and then Ford has been given the mandate to actually implement those. And by implementing those, what I mean is what we’ve put in place a country pilot so basically to test practically on the ground how this cooperation works and this is what I mentioned to you earlier so those are the pilots that we are now leading in Mexico, in India, in Miramar, and soon in South Africa.

We use those principles… and we’ve created a questionnaire based on those to have a diagnosis of the situation a relationship between the government and the foundation, so there are two questionnaires that I’ve sent to government, different varieties in Ministries, and then also to foundations and then our [donors? 00:20:19] analyze the statements of the situation at the moment and those people who worked with the recommendation, so this is what we are doing in several countries right now.

Interviewer:	… How do you typically initiate contacts with a government? It seems often that governments, while interested in working with larger foundations, have many different obligations and many different coordinating meetings that they already attend and hold, so how do you initiate when you’re trying to convene, let’s say the this particular Foundation and the government of India, Liberia—wherever they work? How does that initiation begin?

F12:	First of all, let’s say that because of where we are hosted, where the network is hosted, at the [REDACTED],, it makes it easier because the [REDACTED], is an international, inter-government organization so that’s why we have the easy access to government. So, the thing is that the [REDACTED],, those are only like in upscale countries so but the [REDACTED]is quite special because the specificity of this place is that we have a separate government party which is composed of the [REDACTED], countries but are sitting at the same board.

The [REDACTED], is working on development policies and not only on the development aid as this is the case for the [REDACTED] passport now and that committee. That’s why, being based and hosted by the the [REDACTED] we have like 49 countries sitting on the governing board [REDACTED] so all those countries, we have representative government sitting at our governing board so we have quite an easy access… In terms of access, at least we have access to those governments.

And then, I understand your question because… actually, this is the situation. Let’s say there are some countries in which even though we have that easy access to government, initiating those pilots are not easy because as you said those governments are already either overwhelmed by the coordination of own donors and not to mention implementation, so they may be interested but don’t have the data capacities and they’re not really interested.

… I would say countries that are not interested are those who need the foundation’s money and they just simply know, either because there’s not a lot of development cooperation aid or because of emerging countries or in-between. For example, there are countries that do not longer receive development cooperation public aid and so they turn to foundations because this is like an additional burst of financing that they know simply by coming and asking for the money it’s good to establish this relation and to start a dialogue, so for them it’s a good way of starting this kind of a dialogue.

So those type of governments—and this is for example the case of South Africa, you don’t need a lot of argument, a big argument, to convince them that it’s a good idea and simply very often the governments are... We facilitate it, we help them, and at the end they may have some kind of a mapping of what the foundations are working and in which sector, so it’s all to help them to start the coordination process with the foundation.

We very often turn to our contacts on the governing board or sometimes we have direct contact from the government or the foundations who are working in those countries, so it must be either a demand... We always need an expression of interest either from the government or any particular Ministry, or from the foundations knowing that we always try to include as much as possible the national, the local foundations and not only international operating in those countries, and of course it means that often there are some countries in which the country situation is not yet natural enough to contract... We cannot push through to this title everywhere.

There are also some countries in which the situations of philanthropy are deteriorating, for example in Kenya, where there are some regulations that restrict funding—international funding for example— for the foundations’ districts. So, yeah, I would say that depending on the national context and the sub-agency for that government to connect with foundations to get this additional financing for public policies, we have more or less traveled with convincing the government to work with us on this. Does that answer your question?

Interviewer:	Yeah, it does. Yeah, it does. Thank you. I imagine these relationships are complex, obviously.

F12:	Yeah.

Interviewer:	Okay. Well, listen. I want to respect your time. I know I’ve only asked for 30 minutes so I always wish these interviews were a [stevy? 00:26:23] longer, but also appreciate nonetheless some of the insight you’ve been able to share. The last question we have is actually just about other contacts. We’re just trying to push from one to the next to the next person. Since you work primarily with the [REDACTED] Foundation you mentioned but then also with country governments...

If this is not allowed by your protocol, that’s fine obviously, but if you would have any personal relationships with different policymakers in Ministry, specifically the Ministry of Health, any connections with this other foundation … take 20 minutes to shed some light on how they are making decisions and how they’re working with these foundations and how they’re considering the various options available to them?

F12:	Yeah, definitely, sure. For sure, I can put you in contact with the this other Foundation. I will contact [inaudible 00:27:45] but what I think would be most interesting for you would be Julie Moore who is responsible for evaluation at the [REDACTED] Foundation and probably what we know about that evaluation, I’ve been [inaudible 00:28:04] before joining the [REDACTED]. I’ve been doing evaluation of public policy as a consultant, but just what I wanted to say is that they work with foundations so evaluation is not our main issue but to say that foundations are getting increasingly interested in impact, evaluation… There is a lot of talking about this, but for sure compared to the public sector there is still very little practice.

Some of them evaluate—and I mean luckily for you, but you know this. Those involved in health, probably for some reasons, they do more [reverse? 00:28:49] research and evaluation of the problem so that’s why [REDACTED] Foundation I think would be an excellent contact for you because they do basically a good job. You will also hear much on this as well that many of the data is simply not incredible. Even if they do evaluations, they’re not publicly available very often, so I would say this is the main... I mean, they are interested, increasingly interested, but compared with the donors and with public, the Ministries, et cetera, this is still not yet a systematic practice amongst foundations.

So basically, I cannot really tell you the details of how it’s found working on both issues, but [REDACTED]for sure is a good contact for you. Then concerning the Ministries, once again, we are not sector specific. We do not really have contacts at the Ministry of Health in those countries very easy, I would say. The only one that we could have had but we can’t… unfortunately it would be in there because this is where our pilot is focused on health-created topics but basically the pilot is run by an association of foundations and we don’t really have a strong invite in from government side so we are struggling to get them onboard so unfortunately I don’t have any specific contacts for the Ministry of Health, but the [REDACTED] Foundation contact would be more helpful.

Interviewer:	No, that’s great. Thank you so…

[end of recording: 00:30:32]
