# Supplementary material for: Supporting Better Evidence Generation and Use within Social Innovation in Health in Low- and Middle-Income Countries: A Qualitative Study
Source: PLoS One. 2017 Jan 26;12(1):e0170367. doi: 10.1371/journal.pone.0170367 (PMC5268497; doi:10.1371/journal.pone.0170367)
Supplement: S1 Dataset — (ZIP) [file pone.0170367.s002.zip › Data/Data - Interview transcripts/F2.doc]

Interviewer:	For the record, sir, could you please tell us what the problem that your foundation is trying to solve and what it’s oriented towards?

F2:	Sure. The mission is to improve the well-being of communities across sub-Saharan Africa which is obviously a really broad purview. Specific dogmatic areas and problems we’re trying to solve are to increase the amount of sexual and reproductive health progressive information that’s available to community members and then access to services around sexual and reproductive health.

The second dogmatic area which maybe isn’t as germane to this conversation is around youth productivity, so the big high-level indicator there would be the ratio of employed youth and the high-level indicator on the SRH side is a couple using protection, of contraceptives.

Interviewer:	Alright. Cool. So, F2, with both of these themes in mind, how does your organization sort that to organizations to which you provide funding?

F2:	A couple routes, not nearly as clean as I’d like it to be. The initial touch point is they’re unsolicited and more of a referral process. We do have an online application form on the website that we accept unsolicited proposals. Just in terms of the input, we probably get... I’m not sure where we’re running this year, probably between 700-800 applications over the course of a year.

The hit rate—the success rate for people who we do not know and do not come with a strong referral from a trusted partner or funder friend—is really low, something like 1 to 2%. The Board of Directors for the Foundation however desires for us to keep that unsolicited mechanism open, so we do. The much more successful rate is referrals from trusted partners and funder friends, as I mentioned. We do actively ask current partners for recommendations around specific geographies and dogmatic areas. We provide a 30,00 ft. level… Well, actually, I’ll explain our geographic focus. Maybe that’ll be helpful.

We focus mostly on East Africa, the five countries of the East African community, along with the eastern DRC and Malawi. So of about 70 countries, about 90% of our very large portfolio of 180 partners are working there. A team on the ground also actively tries to identify good organizations in our dogmatic areas. We have a team of 5 across East Africa, including 4 Africans and one ex-pat.

Interviewer:	Okay. So regardless of whether it’s referrals or the accessed online applications, do you have a specific selection criteria that you use?

F2:	Sure. A little bit about our portfolio, it’s sort of constructed in 3 parts and we think they’re… important, that each of them exists; sort of three legs of a stool. The first one is something called “grassroots innovators place-based holistic service providers,” mostly locally led. So you think about a typical rural clinic that realizes that to achieve health outcomes it desires, it needs to intervene in the classroom, in the field, in micro-finance groups and so they sort of morph with these holistic groups. This is our grassroots innovators.

The second bucket would probably look more like goal-orienting and maybe a little bit weird. They’re folks we call “system innovators” so rather than an restricted catchment area, they’re working to take an idea broadly to scale, something like one major fund that’s the fully formed version — this health organisation and all those folks on your list, another health organisation, and so forth — those are folks we would term system innovators. We try to catch them on an early stage because our money needs to be meaningful. If not, our average grant size is between $60,000-70,000 on an annual basis so obviously that is not as meaningful to one health organisation as is might be to another health organisation (while they’re growing by leaps and bounds).

The third group is something we call “learning partners.” This is essentially INGOs. These are the larger organizations: various organisations. These folks we actually… I should backtrack. The other two we don’t apply any earmark. We, for the most part, provide general operating funds to the grassroots and system innovators. For learning partners, our money is not going to be very meaningful for them unless we’re able to get something out of it, so we apply a learning earmark and a mechanism specifically to share that with our smaller partners. Just like those guys have big research shops, they bill you to carry out our RCTs, the sort of assessment that isn’t affordable for most of the other organizations in those two pockets.

Interviewer:	Okay, okay. Question, F2. When you’re talking about the referral pathways, who are those trusted partners? Why do y’all trust their judgment and do you apply the same criteria to organizations that they suggest as organizations that come to you without a referral?

F2:	… The trust and reciprocity comes from experience and familiarity, I guess, so when I talk about trusted partners, they are folks that we have worked with for multiple years that have sort of hit their marks with the milestones they establish for themselves and that we track through our reporting and that we built personal relationships with, either a team on the ground or if they have a US-side representation in the US… For example, I just pinged someone from a health organisation on Thursday about a healthcare organization that was recommended to us actually by another trusted partner. They sort of triangulated on that.

…	We ask in our application process, we ask for some of their important major funders and if we recognize any of those names, we’ll reach out and get a referral from the funders as well. So, I guess it’s just time and familiarity that helps us build that relationship of trust.

Them, the second part was do you treat the guys the same—unsolicited versus referral? The answer is yes for the most part. A referral will probably get you a call at a higher rate, which is sort of the second step in the diligence process —the phone call— at a much higher rate. You sort of get fast-tracked to that point rather than an unsolicited person, we’re going to likely ding them at that first level of diligence, which is just a path through their application form.

Interviewer:	You mentioned your awesome staff doing their own sort of research, ‘cause I guess do you ever worry that the referral mechanism you end up funding people who already have funding and that can have a transformative effect when a bunch of people coalesce or start to pour money into a particular organization, but it can also have the effect that “before you’re noticed, you’re not noticed” so you give money to people who already have money?

F2:	Gotcha. It’s a bit of an echo chamber.

Interviewer:	Yeah! So how do you guys balance those things?

F2:	You know the funders too well… I think it’s always a challenge to identify a talent that hasn’t already been identified, but I think we don’t compared their damage to other small and medium-sized institutional funders, including many of which attend conferences — the Big Bang Philanthropy Awards.

We have folks on the ground and so those guys really... Our peers have a larger challenge in identifying early stage or African-led organizations because they have to parachute in once every six months or so, spend a few hours on the ground, and they go and get a lay of the land. So, I think that our team of five on the ground are cross-worked. I’m sure this is a *huge* advantage and it helps us to identify smaller or African-led organizations or other folks are able to.

[REDACTED]

Then, pinging our local partners about local rock stars. It’s something that is built into our reporting and site visit diligence forms… We’ve sort of shifted from a simply organization focus to more of a country-level focus so as an example, in Burundi, East Africa (which is a francophone country), Burundi doesn’t get any money because long story short it has a much less attention than other countries in East Africa, including its sort of stepsister, Rwanda, from funders and thusly INGOs and organizations outward. We went in there and we found an incredible local, guy named Dedo—one of our staff—to run the program and we created our own incubator ‘cause we saw a dearth, a sort of ecosystem, that was supporting early stage social enterprises and visionaries.

That was the one case we didn’t have a referral network. We didn’t have smart folks we could lean on. We weren’t finding the types of organizations that we wanted to. Just had to build it ourselves… and that’s been a really cool and challenging program. We have not done a lot of program designs so that was an interesting sort of maiden voyage, but we’ve seen great results. We’ve seen an incredible return on investment in terms of…. on the fuzzy qualitative side, helping empower the visions and visionaries there in Burundi. On a more quantitative side, we’ve made a lot of connections and tried to leverage funding towards the incubatees and we’ve seen a 10x return… Comfortably, I can say 2 million dollars leverage from connections that were made directly in the incubator against an investment well under 200 over the last couple of years, 200,000 hits. That was a long story, short answer. Sorry about that.

Interviewer:	No, that’s really good. So, you talked a bit about return on investment and some of the metrics you used trying to find which organizations to choose and how you define success. Can you tell us a bit more about some of the other metrics that you use to define success?

F2:	Sure. So, with those two buckets—with the grassroots innovators and the system innovators—smaller works are either heavily rooted in communities or have a big early stage idea they want to take to scale. Those are organizations that we’re focusing primarily on indicators of organizational health and organizational capacity development so things like if they have chief documents in place. [REDACTED]

Interviewer:	Yeah, that’d be great.

F2:	And we actually run it ourselves with a subset of partners every year, about a dozen who are sort of the exemplary folks who we’re really excited about. It’s a two-day process and it’s really fun to begin pre-organization so neatly.

Anyways, so some of the things that are captured there and then you can sort of look at a longitudinal, circle back in a year or two, and see if their assessments change are organization documents, strategic plan, financials, financial reserves, things like that. Whether or not they have dedicated paid full-time employees across different areas including M&E, finance, accounting. The composition diversity of their board as well as their senior leadership. It’s pretty important that even if you’re an ex-pat organization, that African voices are really heavily informing your work as well as women, which is a challenge that we’ve seen in East Africa — to find an exemplary, early stage organizations led by African women — so we’re certainly doing some affirmative action when we’re thinking about selecting projects there.

And certainly, growth of their revenue over time, and we attribute that… We do have this leverage funding metric that we track for ourselves... One of my staff is sort of doing some desk research on this because it’s really unsatisfying. If you guys could actually point me in a great direction, I’d be happy. It’s really unsatisfying how a lot of funders say we track the funding that we leverage get from other funders towards our grantees but how they define it and track and then sort of think about leverage funding is super fuzzy. I just haven’t found a meaningful way of thinking about it yet.

Our basic idea is that do we think that… Firstly, we only take credit for first commitments, not for follow-up funding. And then things get even fuzzier, how confident are we that this money would’ve gotten there if we had not intervened with a call, with an email recommendation, by convincing a funder to join us on the ground for a site visit? So, we’re starting to think a little bit more smartly about that and unfortunately, I just haven’t seen a lot of good thinking or writing about it so we might be trying to create some new things here. And if we do ever figure it out and feel like we’re smart, we’re going to share that with funders as much as possible.

Interviewer:	Okay. So you talked a bit about the OCAT tool that you used to try and define some of the metric. Can you tell us a bit more about some of the other ways you try and track evaluation and monitoring metric?

F2:	Sure. As I mentioned, we do have those two principle CPIs at a high level: youth employment rates and a couple using protection. We do our best to aggregate the numbers for both jobs created for youth or small businesses created as well as a couple using protection by our health partners. that’s a challenge as you guys well know because people track stuff differently across borders and across organizations, so we’ve kind of had to be a little heavy-handed into forcing partners to submit information the way we like it, which has meant that our reporting... It’s kind of been an interesting last couple of years.

In an effort to sort of ease the burden, we sort of spent a couple years really pairing down our reporting so that it was… It used to be twice a year and a heck of a lot of info we asked for and we got it down to once a year and a lot less info and then we just transitioned to Salesforce so suddenly we’re kind of ballooning again to asking them for a lot more info and I think we’re gonna have to go through another pairing down process…

When people report once a year, which is essentially two months out before the cessation of their grant period, they’ll provide that info according to how we what them to bucket it. Kind of asked for the least bad formulaic way to think about things, so on the contraceptive provision, we just ask for quarterly numbers ‘cause that’s what most countries seem to be asking for for the government we’re reporting, but it’s not always how our partners collect it depending on the circumstance so it can be a little painful.

Interviewer:	Yeah, okay.

F2:	Theoretically—and I say it’s theoretical because our Salesforce is not a finished product yet— theoretically, that’ll all be spat out really nicely in a polished report so that we can actually digest and be able to form our own thinking about our partners and how we support them.

Interviewer:	Yeah. Okay… Just a bit more about the reporting requirements. Do you use different reporting requirements based on the different organizations that you work with or that you choose to support?

F2:	Yes. We track based on SRH and on the youth productivity piece and those reporting requirements are different for different partners. We do have some health-specific organizations, but we have a lot of fuzzy stuff where people are doing multiple dimensions. We’re trying to capture that, and like I said, I think we probably captured too much of the diversity right now and we need to sort of be a little bit more selective in what we want, but because the CRM is in its early days, I think we’re not ready to do that yet… and we’re busy.

Interviewer:	So how do you choose which metrics to track, specifically? ‘Cause there is potentially a lot of [inaudible 00:18:15] there you could collect, but you can’t collect everything. The resources are limited.

F2:	Yeah… I don’t know which metric to ask for. Well, I guess it’s sort of been a long iterative process as we’ve discussed as a team and tried to figure out what’s meaningful for us in terms of financial data, programmatic data, impact data, and then sort of data around the team and the composition thing I mentioned.

We do want those aggregate high-level indicators [inaudible 00:18:48] in jobs created. And we do want to track financial trajectories over time, be able to compare them across partners, and the way we bucket partners is mostly by... So, we do that sort of candied but rough drop-downs for help and use and then “other” and we sort of find ourselves cutting and parsing by country level quite often…

I guess we’ve done it sort of looking at our historical partnerships, looking at fundraising trajectories over time against when they became a partner with us, which we don’t think is all that meaningful in terms of attribution, but sort of interesting food for thought. We cut and parse partners based on if they’re ex-pat led or locally-led, men or women, whether we think that one of their top two to three thought leaders is African, and try to look at data from those perspectives to figure out which types of organizations are sort of succeeding in our portfolio and if there’s anything that we can do to support the ones that are struggling.

Interviewer:	… So you talked about standard indicators that you collect vis-à-vis contraception and impact things. Do you guys ever probe a little bit into potential harms that the organizations you fund might be creating, like just given the history of a lot of well-intentioned social ventures that either go awry and don’t show impact or actually cause some sort of setback for participants?

F2:	Interesting. Let me think about that… My first answer is not really, but then I wonder if we’ve thought about it but just haven’t touched it in...

Interviewer:	Those terms?

F2:	…deleterious effects of our partners. I guess not really, systematically. We’ve come to some conclusions that a lot of folks probably had well before us about the types of partners that we like. For an example, we moved away pretty concertedly from supporting orphanages, which was when I came onboard as the first executive director in late 2010, we had a number of folks who were institutionalizing OVCs and I think the team on the ground is pretty uncomfortable supporting those types of organizations and I think we’ve looked at the examples of institutionalizing OVCs here in the US and elsewhere seeing that it’s probably not the best work relationship.

So, we’ve moved pretty concertedly to home-based care as an alternative to orphanages and figured out how community-led efforts to support those households can be much more beneficial for the kid, for the community. But that was really a one-off realization… I guess in a sense too the work in Burundi is another sort of one-off example. A little bit more background on why we created this incubator for Burundian-led organizations was not only was there a dearth of funding but there was a real lack of agency on the part of Burundian organizations.

As I mentioned, we had a struggle to find partners that we liked and were excited about and one of the things we saw was there was a lot of people with a mission statement that was like a page long, so what happened was they were very much following the donor trend du jour so you had people who did LGBTQ rights. The year after, they did food security. The year after, they did village things and loan associations. It was like they were all these sort of missionary organizations and so one of the things we hoped for with the incubator would be to find some Burundian voices and some Burundian visions so that there could be locally-led development. We saw it was problematic how aid was working—or rather, not working— in Burundi.

… One of our outcomes there that we wanted to see was more unrestricted funding on the part of either funders or INGOs who had passed their funding and we’ve actually seen that quite a bit. We’ve seen [REDACTED] who actually came on, asked us to become a partner on that incubator, and we’ve seen them shift more of their resources and (1) to more local organizations, (2) with less restrictions than in years past.

Interviewer:	Moving onto another area which is often forgotten about from the funder’s perspective as well as possibly evaluation-monitoring. Do you ever provide funding for organizations to undertake evaluation and monitoring activities?

F2:	Sort of. On an organizational development focus, we’ve largely not… As I mentioned for the most part, we’re providing unrestricted funding to most of our partners so they can undertake and allocate as they wish so we don’t dictate. However, we’ve also provided some additional funding on occasion for systems building so actually we underwrite a lot of Salesforce customizations. That’s probably the chief thing we do. We’ve got a cute acronym—KSS—which is Knowledge Systems Support. (We’ve got a lot of acronyms.) At any rate, we’ll pay for them to hire their solutions or another Salesforce customizer to try and get their data systems working a little bit better which you know by and large, it’s a common challenge, including on the funder side.

And then I guess in some of our learning partnerships have been directly… The learning partners are those big guys, right? We have funded specific impact assessment there, so an example would be this health organisation. We’ve provided a grant that looked at adolescent health promoters so basically you know this health organization does a particular model in Uganda and elsewhere. It’s largely women in their 30s, 40s, 50s — respected members of the community — doing the Avon model. This health organisation wanted to tinker with it and see if they couldn’t do more peer-to-peer stuff, so getting 18-year-old women, 21-year-old women to be providing health goods to other young women.

So, we underwrote that and we tried to really dig into the results which were sparingly paltry unfortunately, and then tried to share those with our partners to do some of the convenings that we do.

Interviewer:	Okay. Earlier in our talk, F2, you mentioned some of the questions you guys still had vis-à-vis your own impact as a funder and you mentioned one question… What’s the landscape of questions that you guys don’t think you’ve answered about what you do and what you want to do…? What efforts are you making into them? What kind of injection of people or cash or some other magical thing would help you guys those important questions?

F2:	Sure... We had a pretty cool theory of change exercise and it’s actually part of that capacity. It’s just when we do the partners and we just ran it on ourselves in January or February. One of the things that we want to believe is that we can be meaningful as a community builder and that, in fact, it does make sense to have these three buckets which there’s a heck of a lot of overlap across them, but they at least help us to be sane about how we think about our huge portfolio and we think that it’s important to have these three components and in fact there’s support.

So, there’s these grassroots folks who have all the trust, reciprocity, and localized knowledge within their communities. You have these systems builders who are these ambitious, big idea folks that are early stage fragile; they’re also risk takers. And then, you have these learning partners, these established folks, who can kind of check the work — the systems innovators — and then we sort of think of the fourth pillar being like-minded funders.

One of the things we want to believe is that in fact bringing these four groups often in the form of convening — like our annual meeting which was just wrapped up in July that had about 300 folks including about 60 members of the funding community alongside our partners’ representatives — that we can invest in that and that’s going to be meaningful and have impact. So in terms of those convenings, one of the things we try and track is that leverage funding number so what resulted is the result of the one-on-one’s that occurred during our annual meeting and that we brokered oftentimes very specifically by saying like, “You should be talking to those people,” which is a pain-in-the-butt a lot of work, but I think ultimately pays off.

Where we’re seeing the gap is that we can see more funding going to people *we* think are good partners within our community. (We’re trying to mobilize funding toward our favorite partners from our favorite funders.) We’re seeing that success, it feels like sort of the cost-effectiveness and how we spend our time, it feels really good. Then obviously, the big leap is tying that to some of that aggregated numbers that we talked about on those high-level KPIs a couple years in youth employment.

There’s a big chasm there about if we’re seeing a meaningful needle movement at a national level in any of the countries we’re working in and I think that’s where we need to do more thinking and we’re starting to do that. We’re starting to think about how we convene people in countries and not just within our network and maybe that’s a way for us to stretch our dollars more. We’re almost sort of shifting towards an advocacy lens around some of these issues.

And so just to wrap that up, I think we’re in the process of thinking that through. I guess the big picture would be how do you tie in organizational development indicators, like increased revenue, and to feel like our partners are stronger themselves to actually national level outcomes, and I think that that is something that we’re excited to explore, but we’re still early days and we’re not sure how to do it.

Interviewer:	Okay. F2, might be one of the last questions, but you did mention some of the organizations which you do fund and we specifically wanted to ask about some of the smaller organizations that you’ve been funding to get an idea about how you came to fund each one. Maybe not individually, but as a group of organizations, …? Can you tell us a bit of background about how you came to fund each?

F2:	Sure… Actually, probably a few through a large network. This health organisation, that was proactive research of conference attendees. Another health organisation was through another organisation actually way back in the day. Another health organisationwas a trusted referral from a board member. Shoot! Who else are we talking about?

Interviewer:	So last one was this health organisation.

F2:	Yeah, this health organisation was another organization referral back in the day… and I wouldn’t say it was a referral. It was us proactively mining their semi-finalist list.

Interviewer:	Okay. these other organisations?

F2:	Yeah, this health organisation, I think might have been a conference connection so again that was proactive mining of a conference attendee list. This health organisation? That was a funder referral. That was other members of the Big Bang network, probably these other foundations; some combination thereof.

Interviewer:	Awesome! Last question and this just kind of ties back to your theory of change, I guess. Why the giant portfolio for a family foundation? Why 180 organizations? What’s the vision there? Or how much of it was inherited? One of the two.

F2:	No, we’ve had a tremendous growth so I would say we had a really heterogeneous portfolio of about 45 organizations when I joined in late 2010. We’ve grown 4x in the last five years… and probably of those 45 organizations, 10-15 are still on our books. In other words, we’ve sort of gotten a bit more focused than that dogmatically and geographically and shed some of the stuff that didn’t make a lot of sense.

The practical answer is that the Board of Directors really likes to keep the turnstile moving and they get excited about experiencing new projects and new rock stars leading those organizations. Another practical reason is I think, to be honest with you, I think we’ve had our cake and we’ve been able to eat it too because… I mentioned that subset of folks that we do the intense capacity assessment with. About 12 of our partners, so 6-7% of our portfolio each year undergo that. The formal title is “targeted capacity support.” So, that targeted capacity support is the [inaudible 00:33:22] day capacity assessment followed by an institutional capacity grant that does have that earmark. That’s for something like a Salesforce customization or building an indicator framework with a consultant’s help.

Then, I also get on a call and walk and just sort of dig really deeply in their fundraising. Our coms manager digs into their story-telling. And then, we kind of try to supercharge it throughout the year so anytime I’m making recommendations, those targeted capacity support partners are at the top of the list. They’re folks that we’re trying to proactively push out. So, in that manner, we’ve had something that looks a lot more like the high-touch relationships that a lot of our partners have with their folks, someone like this Foundation that takes [boardroom seats? 00:34:07] and [Homing? 00:34:11] has like 30 or 40 partners across a staff of 6, probably close to our staff size. We’ve gotten that element with a few partners that we’re excited about, while a good chunk of those grassroots folks, frankly, they’re not moving very quickly and I think that means they require fewer touch points and less time on our side.

So, we’re stretched pretty thin as a staff, especially the Africa-side team, but I think we’ve been able to keep the meaningful relationships and have a pretty good finger on the pulse of most of our partners: from reporting, from site visits, from convenings. So, it definitely causes a lot of raised eyebrows, but I don’t think we’re running off the tracks, despite the large number of partners.

Interviewer:	No, that’s awesome to hear. Cool! Well, I don’t know if Jenny has any more… questions for you.

Interviewer:	No, that was really good. Thank you so much for sharing with us, F2.

Interviewer:	Cool! And I don’t know if you have anything else you want to say, F2, just vis-à-vis...

F2:	No. Just what are you guys trying to deliver openly?

Interviewer:	I think what we’re looking…

F2:	Effective in their own research and just trying to track down other people’s work, established work, there’d be a lot of improvements. That being said, we’ve gotten so many requests like, “Can you just build a database of best practices? Can you fund that and then just…?” There’s a database already out there. It’s probably free of charge. What don’t you guys use that?! And it’s been frustrating with people wanting to be led to the research and just best practice information, but there’s a lot out there and everyone kind of wants to build a new thing.

We found success or at least we’ve decided that what we can expect ourselves is just to serve as a convener. For the most part, this information is passed on the basis of certain personal relationships, so we need to just get people in the room, underwrite the bar tab, and let smart people do their thing and exchange idea with a little pushing from us. But, we’re not able or interested in underwriting a database or a library of resources… without a mechanism to proactively push out that information in some curated fashion. I just don’t think that those are going to be utilized all that well. Anyways, that’s my gripe.

Interviewer:	No, that’s why we’re trying to get a landscape and also a lay of the grapes, what do you want to see happening and how could Skoll support that or others? So, that’s awesome. Thank you so much.

F2:	Well, at Skoll… but there’s also a “do no harm” thing like we kind of got into earlier. Unfortunately what donors do is they underwrite and they incentivize a lot of cynical, crappy partnerships among their grantees, including stuff that’s actually born of... Skoll’s well-informed at least. I don’t actually know all the stuff of the foundation’s work the other 363 days of the year, but the forum’s a really excellent convening place. I do like it how, for the most part (and maybe it’s because I don’t attend a lot of actual content), Skoll sets up a great space and gets out of the way. In its simplicity, it’s the most clever thing that anybody can do.

But still, you see even from that forum, from CGI, a lot of heavy-handed partnerships and that’s something that we’ve been really cautious about is saying one partner to implement a program and another partner because from our very non-technical and lack of expertise view, it makes sense. And grantees will likely not say no, but they might get something that’s really not meaningful for them. Sort of like a “note of caution” on the funder side. We try to always be conscious of that, that if it’s something where they’re feeling compelled to do to enter the partnership or even just attend a conference that we underwrite and it’s not likely to be valuable but they feel like the long-term partnership with us is threatened if they don’t. Tough! Tough power balance [inaudible 00:38:30].

Interviewer:	Indeed.

F2:	Ok, guys. Thanks.

Interviewer:	Yeah. Thank you.

F2:	Have a great day. Talk soon. Madeleine, I hope to see you at some point.

Interviewer:	Yeah, definitely, definitely. Have a great day, dude. See you soon.

Interviewer:	Thanks, F2.

Interviewer:	Alright, bye-bye.

Interviewer:	Bye.

*[sound of Skype phone dialing]*

[end of recording: 00:38:54]
