# Supplementary material for: Supporting Better Evidence Generation and Use within Social Innovation in Health in Low- and Middle-Income Countries: A Qualitative Study
Source: PLoS One. 2017 Jan 26;12(1):e0170367. doi: 10.1371/journal.pone.0170367 (PMC5268497; doi:10.1371/journal.pone.0170367)
Supplement: S1 Dataset — (ZIP) [file pone.0170367.s002.zip › Data/Data - Interview transcripts/F3.doc]

Interviewer:	Okay, so we know you’re from this Foundation. Just for the record, can you tell us a bit more what your Foundation does and what your specific role is?

F3:	Yeah, so our Foundation is just a ... Just to back up a bit, our foundation itself is a healthcare investment organization so primarily the larger organization is engaged in the business of financially enacting commercial entities within the healthcare space, however we have — within the organization— a foundation which is more charitably directed. That’s funded partially by employee contributions and partially by a portion of the profits from our investment funds, so that’s kind of where our [amenable? 00:01:03] proceeds on an actual basis come from.

The mission of the foundation is primarily focused on increasing access to healthcare for underprivileged children and so we’re active. We’re a [redacted] based organization so we try to be active within the [redacted] area, probably about half of our funds annually and then the other half are more internationally based and support projects within the mission and a variety of geographies. Is that helpful?

Interviewer:	Yeah. No, that’s perfect, and can you just briefly describe what your particular role is?

F3:	Oh, yeah, my role. I am on the board of the foundation. Actually the way this works in our foundation as that the board rotates every two years and so employees of our foundation can be elected to the board on a two-year term so we’re involved in kind of diligence-ing potential new entities to fund and also whether… by determining whether we ought to fund organizations we’ve worked with in the past, and so we ultimately make recommendations to the broader group on which projects ought to be funded and how much and [inaudible 00:02:31] and that’s how we factor our grant annual funding cycle.

Interviewer:	Okay, so does the Board or the foundation have much over with the selection committee who eventually decide which organizations get the funding?

F3:	Well, they’re basically the same. I mean formally there is a vote of all employees at the firm on which programs to fund, but basically the board/selection committee makes the recommendations and historically I think they’ve always been ratified, so it’s basically the same body.

Interviewer:	Yeah. Okay. Thank you for that; that’s very clear now… So, correct me if I’m wrong. Your Foundation is kind of the subset where you fund particularly healthcare initiatives, mainly based around children. Half of the funds go towards local projects in [redacted] and half of them go to a more international scale. Is that correct?

F3:	Yeah, that’s about right.

Interviewer:	Can you give me a bit of background as to why the your Foundation has decided on this particular mission?

F3:	… That’s a very simple question. I think broadly we view ourselves as in part the advancing healthcare portion of it is something that we broadly view as a role of this organization and try to [inaudible 00:04:08]. We have a variety of different issues. I talk about our foundation and our investment fund— they’re all kind of aligned around leveraging our expertise in the healthcare space, hopefully to make the world (not to sound too idealistic) but make the world a better place in the sense that we can. We all have a background in healthcare. Most of us are doctors or scientists by training so I think that the working in healthcare space part is really just what we do.

The part of the children actually… I don’t know. I can try to find out for you if you’d like. The organization’s been around a lot longer than I’ve been involved with it, so I don’t actually know what the original thinking was behind focusing specifically on pediatric needs but I can find out from people who have been around longer than me and let you know later.

Interviewer:	Yeah. No, that’s fine. I just wanted to get particularly your perspective on things. In the time that you’ve been with your Foundation, is there a story that you can tell us about the impact that the foundation has had that you’re particularly proud of?

F3:	Yeah, sure. I think that … One that I like, it’s… Maybe the impact is not on a huge number of lives in the one that I’m going to relate, but I think it’s one that’s been kind of personally meaningful to a lot of us at the firm and that’s a project we’ve been working with this organisation for a number of years. It’s an orphanage in Haiti. We’ve been working with this organisation in Haiti for a few years before this project began, but in 2010 when there was the earthquake there, a hospital was destroyed and within that hospital had been a group of 60 or so orphans, many of whom had kind of physical disabilities requiring a lot of care.

There was really nowhere for them to go with the hospital having been destroyed so we funded a significant portion of construction of the orphanage which is home for the orphans and so they’ve been living there since 2010 and we continue to assist with the operating budget. We’ve—many of the members of the group— have traveled to Haiti and had an opportunity to meet the people personally that our foundation is trying to help and because we’ve been doing this for a few years we’ve seen of them begin to grow up and some of them have began to go off to University and begin to transition into their adult lives.

Increasingly, the orphanage is also becoming more self-sustaining. They have a variety of agricultural projects and things that are beginning to provide revenue for the remaining kids at the orphanage. So, we’re pleased to see the personal impact that this project has giving for the kids and as an organization we also like when possible for a high-[inaudible 00:08:01] investment to lead to a sustainable solution for people and so I’d like to see things move in the right direction as well, help with making a lasting touch. That’s one that springs to mind, but I don’t know if that’s helpful to you.

Interviewer:	No, that’s a great story. It’s really great that you had started working with the community even before the disaster struck so to speak and you continue to assist them and help them transition back into that [inaudible 00:08:34]. It’s really beautiful to see what impact you have made. No, it’s a really nice story. Thank you for sharing that with us.

F3:	Sure.

Interviewer:	I just wanted to step back into the more… administrative side of being on the board of the foundation and being part of the selection committee. Can you tell me a bit about how you actually select the organizations which you provide funding for?

F3:	Yeah. Each year, we have kind of this informal application process, I suppose, which basically… Essentially, it consists of organizations expressing interest in potentially being funded along with sort of understanding in general what our mission is. We’ll generally at least take a first meeting or a phone call with almost anybody who we hear is on the surface to be a potential interest. And so, there’s usually an initial screening like that in which... We encourage the organization to… Mostly, we vet a number of characteristics of the organization such as how sustainable they appear to be based on along with where they’re at historically and what the makeup of the leadership is and what their other donation sources have been.

We also try to assess (to the extent we can) physically what they’re trying to accomplish aligns with the type of projects that we’re hoping to fund within our mission. As I said, I think that… As I mentioned earlier, sustainability is important and also this is mostly new organizations I’m talking about, because it’s not a characteristic we look at as kind of our level of confidence in the ability of a group to do what it is they’re setting up to do and that’s harder with organizations that are new to us. Many organizations we work with we have funded in the past however and so we kind of gain confidence on that ability to execute metrics from our past experience.

So, that’s kind of the first checklist kind of interview. Assuming that a group of peers on the surface to meet all of the criteria in principle, we then usually solicit a specific project idea. Most of our funding for new organizations is what I guess what you call a “restricted funds” and it’s to be associated with a specific project because we find it easier to measure the… successful execution and act of sort of specifically define that.

For some of the organizations we’ve worked with for a long time, we’ve started to give more unrestricted funding because we trust that they will know how to use it, but let’s say for earlier organizations, we usually have a one project basis approach.

Interviewer:	I see. Okay. Okay, so between that first screening informal application, is there another stage before you solicit the funding to the specific project?

F3:	Yeah. There’s an initial meeting usually by phone. As I said, for anyone who kind of moves on to the next stage, we’ll have them… Usually, there’s a single liaison that’s kind of working with the organization and so we’ll work with that individual to frame the outlines of a project that might be appropriate and they will all come in for a personal meeting, speak to the broader board and describe the organization to the board, and typically give the general project and proposal that they might have in mind with some general description of what the project would be and enact some sort of initial budget.

Then, at that point, the committee looks at the candidates and our available funding for that year and selects a short list of projects that we probably *will* fund, then we request a formal written proposal from the organizations describing in more detail what they plan to do with the funding, what they require it for, if that’s not enough funding to cover the entire project, how they anticipate getting the remaining funds, and also what metrics they will use to more or less verify success or initial progress on the project. Usually we get a report twice a year. (That’s what we ask for.) Just briefly updating on progress against those things.

Interviewer:	Yeah, okay, alright. I just want to come back to metrics and reporting, but just before we move onto that, I’m just interested to find out… It seems like you target both new organizations who you’re unfamiliar with as well as organizations you’ve worked with before. Just specifically with newer organizations, how do you reach out to them, like how do you find them? Or how do they find you?

F3:	Of course, there are the ones we’ve already funded before. We always speak to them every new funding cycle. The other ones, I think… We’re a relatively young foundation & have been around since 2008 so until now I think we’ve kind of largely been working on a sort of a personal referral basis either from some other group has connections to heads of other foundations and other foundations who may recommend ideas they think are interesting. Others may just be—especially the local ones in New York City— things that come up in the lives of members of the firm so it’s been a fairly informal process and the amount that we have to donate has been growing and so we’re kind of working on coming into the more formal process, but I would say that’s still a work in progress so it’s really just personal network and referral by word-of-mouth essentially I would say so fairly, fairly informal.

Interviewer:	Okay. And the referrals, do most of them get through to that first screening stage when you try and look at, like, sustainability, leadership, and other donation sources?

F3:	Yeah. I would say almost anything that sounds on the surface of it to be potentially worthwhile we’ll at least get an initial conversation. I think if they had been personally referred like for example by one of the organizations that we already fund and have been working with for some time, that raises our confidence level and so it’d probably be beneficial… But, I [suffice? 00:17:23] to say that those who come with a personal recommendation probably we pay closer attention to, sure.

Interviewer:	Okay, okay. And so how specifically… You’ve talked about some of the metrics or criteria that you use, but how specifically… What do you define as success or impact in the organizations that you decide to fund?

F3:	… That’s very much variable by situation. I don’t know if there’s a generalizable answer to that. Sometimes it’s just kind of obvious, like sometimes it’s a project involved in constructing a piece of infrastructure like a medical clinic or a well or something. In that case, it’s fairly straightforward to determine whether or not the object has been constructed. In other cases, it’s a bit more nebulous and... it is difficult in many cases where things are more qualitative really to know. I know you were kind of referred to us by a health organisation.

Interviewer:	Yes, that’s right.

F3:	That’s an organization that I think has been particularly impressive in really setting out from the outset to determine what kind of root causes of the problem they’re addressing are and prospectively measuring the impacts of intervention on those root causes. As a funder who’s not on the ground in these places and doesn’t necessarily have insight into those things, it can be difficult to sort of guess our priority what the most important things to focus on are and so I think it’s a really strong approach from organizations who’d like to be funded to think about those things in the context of their own climates because if those metrics can be provided to us by the organization, it gives us a great deal more confidence in understanding what we should be focusing on and being able to measure progress.

Interviewer:	So with the metrics for each of the organizations, so say for this health organisation, how much input do you have into what metrics they use to measure or verify their success? Do you help them define it or do you leave it completely up to them?

F3:	I would say that… I think that if they… It would be our preference, I would say, for them to be able to tell us what the most important elements of their project are to measure and provide updates on those. I think they’re more expert than we are in general in their particular area of expertise and so if they can provide those things, that would be our preference. We’re not interested in trying to force an organization to conform to some idea that we have about how they should best do their work. We would like them to think about it though and be able to give us something to… some concrete thing to understand what’s happening with the [inaudible 00:21:18].

Interviewer:	Yeah, sure, and that’s why you’ve got the twice-yearly reporting requirements for each of the organizations.

F3:	Yeah.

Interviewer:	… Do tell a bit more, so are there specific requirements for the reporting?

F3:	Yeah. Other than being a twice-yearly written report, I think that’s probably the only common requirement for all the organizations. There’s specific elements of the report that are agreed upon at the time the funding is committed and they vary from…

Interviewer:	Okay. And then once you receive these reports, how do you use the data from these reports?

F3:	Well, I think that hopefully we’ll see the group meet or exceed the objectives that they set out for us at the beginning of the funding cycle. If they have, that’s great. That raises our confidence in continuing to fund the group going forward. If they haven’t, obviously that makes us nervous and if (in a couple of instances at least) if there’s been a chronic failure to achieve stage goals, then we’ll stop funding the organization so doing well in those metrics can increase your funding rounds and doing poorly can reduce them.

Interviewer:	Yeah, okay. Okay. So it sounds like the reporting requirements, you do kind of give the organizations quite a lot of liberty into what they want to put into it and it does have some impact on whether additional funding will be provided with the next funding round.

I guess one of the key things to make the reporting efficient and effective is to have monitoring and evaluation systems in place to be able to measure metrics. Do you ever specifically set aside funding for organizations to carry out monitoring and evaluation?

F3:	I don’t think that I’ve seen us do that… I don’t know if that level of granularity is there. If part of their budget proposal includes budget line items for monitoring impact, I’m sure that could be part of the monitoring. Many organizations don’t have that level of detail in the precise use of the funds, but we certainly don’t have any objection to some of the funds being used to measure impact. I think that as you could sort of tell, I think, from my answer to one of the earlier questions we’d really like to understand what impact we’re having but it’s very difficult to quantify it at times, so we certainly have no objection to use of some of the funds doing that and doing that carefully. We’d love to see more organizations pay attention to meaningful, concrete quantitation of their work.

Interviewer:	Yeah, yeah, okay… So, just a question that comes a bit left of center, but for some organizations—despite best intentions— some of the activities that are carried out can potentially be harmful. Have you on the board of your foundation thought about ways in which your program could be harmful or the programs that you fund could be harmful?

F3:	That’s a good question. We certainly think about ways in which the program could be unsuccessful, risks to achievement of the goals that have been set out. I don’t think as part of our process we have a formal consideration of whether there could actually be harm done. That’s a good question, but I have to confess. I think that’s not something that we have thought about in the past, specifically.

Interviewer:	Yeah, okay. And final question: in a perfect world, what do you think would help do your work for the your Foundation better?

F3:	Well, are we talking about specifically measuring impact?

Interviewer:	That would… Yeah. If you have a specific answer for that, that would be great, but then also in any other case like for instance whether improving criteria or various other things which would impact on, say, how you select organizations or you manage grant funds, so generally as well specifically with evaluation monitoring.

F3:	Yeah. Well, I would say for our fund, the ideal project for us to fund because we have a meaningful number of dollars to contribute but not an overwhelming number of projects so what we’d like to fund the most is a large project at an inflection point where there’s a pretty good chance that it’s going to have a benefit and it can kind of leverage our investment to prove that benefit and potentially get into a larger scale.

And so, I think it would be helpful (as I alluded to earlier) if organizations focused on—before they do anything on the ground— just characterizing 1) what the key problems they’re trying to solve is, 2) that the key steps to getting there or some problems that can be quantitatively measured, and then ideally running a pilot intervention in a small area that’s very manageable and measurable and then... That’d be my favorite point at which to see organizations — when they’ve done something on a small scale that kind of had a clear impact and then, say, we would be at the stage where you can try this intervention on a larger scale, so that would be the perfect place. I think for what I’ve seen for far many organizations either don’t think very systemically about what they should measure to either show an impact or if they have not shown an impact they want, then how to iterate that data and modify their approach. Often, that’s just kind of a large implementation of an idea without clear testing of it beforehand so… A little bit of a rambling answer, I guess.

Interviewer:	No, no, no. That’s a good answer, yeah.

F3:	I think that thinking systematically about the design of your implementation and testing it in a way that shows that it works and then coming to us for sort of securing funding, that’d be terrific.

Interviewer:	Yeah. So that inflection point you were talking about, was that just after the pilot intervention has been implemented and the data’s been collected and there’s clear results to show it does work and you guys come in when there’s an opportunity to scale the project? Is that right?

F3:	Yeah, I think so. I think really the most efficient way for all philanthropic organizations just to see the ideas that work get hailed to the point where they can have a real impact without wasting philanthropic capital on an idea on that scale that hasn’t been tested.

Interviewer:	Yep. And at that point of the organization… You talked a little bit about some of the metrics like sustainability and leadership. Are there any more metrics that really, really steer you towards supporting an organization to scale?

F3:	… Well, that tends to be kind of idiosyncratic. To again, I guess, to get back to the example of this health organisation, which is a really good example of how to do this in my opinion. That group had a clearly definable end to problems that they were trying to impact, which in this case was early childhood mortality, and before they did anything they spent some time studying why there was a failing in this end point: a high child mortality and identified several specific failings in the healthcare system, namely a timely identification of patients in need and timely treatment of those patients and access, access to clinical care. They had three sub-problems to measure and then they began an intervention on a small scale and identified ways in which they’re improving those specific things and ways in which they weren’t and how to go back because they knew what they were studying and what they were looking for [able to? 00:32:15] sort of iterate the process and improve it.

And that group went so far as to actually publish a peer review paper on their experience which was really, really impressive, so it’s a specific example and I think that the exact metrics will be different in every case but the key is to have an understanding of your particular problem of what things leading to the problem are and what you ought to be measuring to tell if you’re [inaudible 00:32:46] and also learn how to do it better.

Interviewer:	Yeah, for sure.

F3:	I’d say to answer your original question, as a funding organization, I don’t think there’s a specific set of measuring [inaudible 00:33:00] by everybody so much as having… every organization ideally should have thought about what the best metrics for their particular problem space are and to be able to defend those to us.

Interviewer:	Yeah. No, that makes complete sense. Just one final question. Just picking up on what you said about the peer-reviewed research, a lot of smaller organizations find it difficult to conduct such thorough research when they’re at such a small scale ‘cause they’re quite limited in resources, but it is very impressive when it does happen. I think this health organisation is probably one of the few who have done that.

For you, how do you kind of think about the balance between resources and conducting really good evaluation and monitoring/ research balanced with actually implementing it and showing that the pilot works? Do you sway one towards the other? I know it must be different for different situations, but what are your initial thoughts?

F3:	Yeah. … I think it’s pretty important really to gather evidence that what you’re doing is having a positive benefit rather than spending slightly more on an approach that may or may not be working. I’d much rather (as I said earlier) I’d much rather see something implemented on a smaller scale with maybe a bit more resources dedicated to making sure that it’s helping and then I think you can attract a lot more investment later with that evidence for a scaled-up version than you potentially could with one that starts at a larger scale but never really shows that it’s something good.

It’s a tough... There’s always limited resources so there’s a question how to allocate it, but my guess would be that you gather strong evidence about your [attack? 00:35:21] early. You’ll be able to attract a lot more investment in the later stages of successful intervention… Really that would be our perspective in our organization.

Interviewer:	I’m very biased myself, but I definitely agree with that. It’s great that you do hold that perspective because I think it’s a tough world and a lot of organizations sometimes might sacrifice some of the evaluation monitoring in creating that evidence that will build up the organization in the long term and they’ll sacrifice that really early on and it’s a shame to see the evidence not gathered up from the beginning because baseline measurements can only be done at baseline and it’s very difficult to go back.

That actually takes us to the end of the interview. Do you have anything else that you wanted to add or any questions?

F3:	No, I don’t think so. Hope that was helpful. I haven’t really done any interview like this before.

Interviewer:	Yeah, that was incredibly helpful. I found it really interesting. I hope you enjoyed the process as well.

F3:	Yep. Thanks a lot.

Interviewer:	Yeah, no. Thank you very much for your help, Nick.

*[audio break/move to next audio]*

F3:	These organizations don’t pay half attention to that in general. I think the ones that do, they’ll have the satisfaction knowing they’re doing something that works, but also I think will ultimately be larger and more successful than those that don’t [stay in? 00:00:18] longer.

Interviewer:	Yeah, yeah, I can’t agree with you more. Yeah, hopefully that will keep improving in the future.

F3:	Yeah, okay.

Interviewer:	Yeah. Thank you very much for your time. Appreciate it.

F3:	Alright. Thank you.

Interviewer:	Thank you.

[end of recording]

[total time: 00:37:21]
