# Supplementary material for: Supporting Better Evidence Generation and Use within Social Innovation in Health in Low- and Middle-Income Countries: A Qualitative Study
Source: PLoS One. 2017 Jan 26;12(1):e0170367. doi: 10.1371/journal.pone.0170367 (PMC5268497; doi:10.1371/journal.pone.0170367)
Supplement: S1 Dataset — (ZIP) [file pone.0170367.s002.zip › Data/Data - Interview transcripts/F4.doc]

F4:	Okay. So here in [redacted], our parent organization doesn’t have a foundation. We are a department inside the company and we donate. We donate money to NGOs in [redacted]. The first one is… So, we choose things to choose that we want to support here in [redacted] based on the values, on the money, that they tell us how much we can invest. That’s made all based on three pillars that the company has. They’re very global pillars and they are improving the lives of women and children, preventing disease in peoples of vulnerability, and strengthening the healthcare workforce capacities.

So, based on those three pillars, we choose projects and the money comes from us. It’s a corporate through [redacted]. That’s made by a fund that’s called [redacted], so they are partners with us on those investments. That’s the first way that we support projects.

The second way is we support projects with the money from the business in [redacted], so we choose a project. They are all aligned with [inaudible 00:02:06], but we just report them to the global team, so they don’t… I’m going to use a very poor term. They don’t have a vote so the whole relationship is made from the [veil? 00:02:31] to [redacted]ian NGOs.

The third way that we do it is that we use tax-incentive projects. I don’t know if you’re familiar with that.

Interviewer:	You use what? Sorry.

F4:	Tax incentives.

Interviewer:	Okay.

F4:	… Instead of paying the taxes to the government, we choose institutions that are validated by the government and we invest money on them. [pause] Is that clear for you?

Interviewer:	Yeah, so how does the government identify those organizations?

F4:	Those organizations, they subscribe to the government - the project. Not “subscribe.” Sorry about that. They develop a project and they… How should I say that? They send the projects to the government, the government evaluates it, and if they pass, they are allowed to receive money that was supposed to go to taxes, so there are four of those. It’s social responsibility. One is related to the well-being of kids and children and adults. The other one is related to the well-being of elderly people. The other one is related to cancer and the other is related to people with special needs. So, we ended up knowing about those projects over the Internet or over an agency—specialized agencies—that bring those projects to us.

Interviewer:	And when the government evaluates projects, what types of metrics do they look at for the organization when they’re doing those evaluations?

F4:	It depends on each law and depends on each project. If it’s an educational project, it’s going to be more related to how many people were impacted. If it’s a project related to healthcare access, it’s related to how many people received the healthcare. There are some projects that are related to researchers, medical and clinical researchers. There are projects related to the well-being of people that are old or with clinical diseases that measure the treatment or the people that are treated in [inaudible 00:05:59] houses and functions where they can get the best treatment, so it depends. That’s what I wanted to say.

Interviewer:	Gotcha. And then once… When you and your team are deciding of these many projects to pick, how do you decide?

F4:	That’s a good question. We have a [redacted]ian committee that evaluates all the projects that we want to support. This committee is formed by three managing directors that we have here in [redacted] for each one of the companies that are part of our parent organization family of companies and also governmental affairs, communications, compliance—healthcare compliance, and the legal department. We get together about two or three times a year and we decide the stretch [inaudible 00:07:08] we want to follow here in [redacted] and also the projects that we are going to support.

Interviewer:	So at that meeting when you’re discussing various projects, why does one project get chosen over another? What types of things do you evaluate or consider about the specific projects?

F4:	Well, the first thing that we evaluate is the alignment with our strategic pillars. (That’s the first thing that we evaluate.) The other thing that we evaluate is how it connects to the business. That’s not a factor of decision itself but influencing the decisions of...

For instance, if HIV is a question, it’s a disease that’s really relevant to the business, then probably we’re going to take a different look at HIV projects. Here in [redacted], we have decided that everything that we do related to social responsibility should be related to a bigger cause and this comes influencing the fight against child mortality, child/infant mortality, so that’s something that we’re always going to be evaluating. It is related to the pillars. It’s specifically related to our bigger cause—that’s the fight against child mortality— and the business.

The third thing that we always evaluate is the NGO that does the project itself. Is it a legal entity? Everyone that sits on the NGO board, are those people… Do they have a problem? Do they have a legal problem? Is there a word for it in English?

Interviewer:	No.

F4:	Yeah, okay. I’ll just let you know really soon here... Sensibility.

Interviewer:	Okay.

F4:	Does it pay respect? Is it all good? Then also, how the NGO performs, so is it an NGO that we track? Is it a NGO that we know that we can study, that does a good job? And for them we do internet searches. We talk to the NGO.

We also evaluate the project itself. Is it a project that we believe in? Is it a project that we think it’s right, that we think it’s important to society? So, yes, those are the four points that we evaluate.

Interviewer:	What types of things make you understand that a project works? Do you go visit the projects? Do you look at studies in a journal? Do you talk to a beneficiary? How do you determine that it works?

F4:	We try to visit all the projects, but not always is that is possible, but yes visiting the project is one way. As I said before, we do all the searches that we can over the Internet to see news articles or blogs or anything that talks about the necessity of the project and also about previous experiences of the NGO.

We do have longtime partners that we already trust in. For instance, the NGO, Saúde Criança, we’ve been with them for about 10 years now, so there is a part that’s based on trust.

Interviewer:	How does an organization maintain your trust once you start funding them? Basically, how do you manage the grant over time?

F4:	First of all, they send a report and we look at those reports to see if all the proposals are being accomplished. I personally try to have a close relationship with all of the partners in order to be closer to the project— to the projects and to the organizations, so we are always talking about what’s going on in the field and what are the results and the next story that we have on it. So, I think that staying close to the project is the best way that we can see if it is working or not, more than the metrics itself.

Interviewer:	Okay. Do you ever, when you provide funding to organizations, is it for specific things or it’s for them to use in ways that they think will be most helpful to achieving their mission?

F4:	Sorry. I didn’t understand the question.

Interviewer:	When you give the money to these organizations, do you tell them how to spend it or they sort of decide how to spend it?

F4:	They tell us how they will spend the money. We look at it and if we have any questions or doubts, we talk to them in order to know… to understand what they are proposing and if we don’t agree, we ask them to change it.

Interviewer:	Okay.

F4:	But that decision is theirs.

Interviewer:	Gotcha… What do you think the role of funders is in generating evidence and sharing evidence that a particular program works?

F4:	Could you explain the question better?

Interviewer:	Sure. When you fund an organization and they give you reports, what responsibility do you think funders like you have to make those reports public or to make those reports simple or things like that? In what way are you influencing that organization and their time through the types of reporting that you make them do?

F4:	I think it’s not a responsibility of the company to show this, but it’s a partnership… We have to work together to bring this information to the public. But, it’s not just about the company. As I said, it has to be a partnership; we have to work together. We can use our channels to [inaudible 00:16:40] our reports and our websites to put all the information there, but it’s not a company’s responsibility.

Interviewer:	Okay.

F4:	It’s nice when we do it because it helps the cause of the NGOs and also it helps the company to increase its reputation, but I don’t want to say that every company has to do it. It depends on the company, the strategy. It depends on how much the company wants to be perceived by this funding, so I guess it’s a more complicated question than it seems.

Interviewer:	Okay. When you’re doing your work, how do you decide or determine whether you’ve been successful in the types of organizations that you fund?

F4:	The metrics are always relevant in this analysis. That’s the main point.

*[call drops]*

*[Skype re-dials: gets voicemail]*

*[re-dials]*

Interviewer:	Hi! Sorry. I think the call dropped.

F4:	Yes. It’s alright.

Interviewer:	Sorry. I was just asking you basically how the foundation, when you’re doing your work, how you determine whether it’s successful?

F4:	The first part is the metrics, the evaluation of the metrics. The second part is evaluating why the metrics were achieved or not achieved because there are many factors, not just the project itself. The third part I think is relevant is evaluating… the qualitative evaluation, so maybe the project it didn’t achieve all the metrics but it made a very big social transformation in the community or a smaller number in people’s lives so that can be successful, too. That’s not measurable. We just have to get out there and get to know the stories in people’s lives. Is that clear enough?

Interviewer:	Yeah. Do you ever look for… Basically, sometimes projects that are started with a good intention to help other people, sometimes they don’t work or sometimes they actually cause harm. In what ways does your organization make sure that the people it’s funding aren’t causing? What types of diligence do you do on that?

F4:	I haven’t had a situation here yet… I don’t really know how to tell you this, but if a project’s causing harm, we’re probably not going to keep supporting it. [crosstalk 00:21:29]

Interviewer:	But how would you tell that?

F4:	I’m sorry?

Interviewer:	How would you tell that it’s causing harm?

F4:	As I said, I haven’t seen anything like it, but for instance, if we’re supporting a project that wants to improve a hospital and the money that we are investing, it’s not really going to the hospital, it’s going to [inaudible 00:22:04]... I don’t know. I’m just trying to create a… to understand the situation, but like if we know that there’s a corruption of the money—that for us is causing harm— so we’re not gonna be supporting this project again. And if necessary, we might take the legal measures that we have to take.

Interviewer:	Okay.

F4:	But I have never had a situation like that.

Interviewer:	Okay. Final question here because I want to respect your time. I said 30 minutes—it’s almost 30 minutes— so I just...

F4:	Go ahead. I have time. Don’t worry.

Interviewer:	I was just wondering do you ever look at… So an organisation just had a study done with Georgetown University at different organizations do different types of evaluations. How important is it to you that the organizations have done a rigorous evaluation, like something with a control group or something with some academic partner or something that takes place over many years, how do you use that information and is it important?

F4:	That’s very important for us to trust that organization if there is someone outside the organization talking about the work and proving that the work that they’re doing they are creating an impact. It makes it easier for us to understand the work that they are doing and also to trust the work that they are doing.

Interviewer:	Okay. But what if they’re… this organisation has obviously been around for many, many years and they’re quite big and they do lots of different work. What if it’s a small organization that’s just starting and they don’t really have the time or the data yet to prove what they’re doing is good or not, do you fund those types of organizations or you only fund people that are maybe have accomplished more?

F4:	That’s not a rule that we have to support organizations that have been out there for a long time. Of course, the bigger the history, the easier it is to create trust but sometimes we do find a very interesting project with an innovative point of view, innovative ideas and we take that into consideration too.

Interviewer:	Okay. And when you’re considering that something’s innovative or some sort of idea that sounds good, is it just a feeling, like a gut feeling, or how do you…?

F4:	It has to be an organization that it’s working already. We wouldn’t support a project of a non-existing NGO that’s going to be created just for that project. Then, we do all the healthcare compliance work and the real work to see if the organization’s fine. We try to see whatever their organization has published or if necessary, talk to some of the other organization’s partners. But definitely, we are supporting an organization that’s 30 years old. They have a nice proposal and I made a research to understand all the other projects that they were doing to see if they were coming out that they were telling us and I spoke to a few of their partners and saw many things about them on the media. I spoke to a few doctors that worked in the Sudan to understand if the organization is fine.

Interviewer:	Okay. Well, I think those are the questions I have for you this afternoon... Just to leave you with one final thought, or to get one final thought from you, I’m just wondering if there was anything that would make your ability to evaluate different organizations easier going forward?

F4:	Sure, it would. It would be difficult but if there was maybe another NGO or some kind of ranking about the NGOs, that would make our lives better. Some organization that evaluates the work that the organizations do… That would be very valuable.

Interviewer:	And do you have an idea of what, when you say ranking or evaluation, do you have an idea of what they would be ranking or how they would be evaluated?

F4:	There would have to be a few points like social impact, internal structure, capacity... all those questions related to compliance. It would be a scan of all NGOs, but that’s really complex because there are too many NGOs in the world for that.

Interviewer:	Okay. Ambitious thoughts! These are good. Great.

[end of recording: 00:29:18]
