# Supplementary material for: Supporting Better Evidence Generation and Use within Social Innovation in Health in Low- and Middle-Income Countries: A Qualitative Study
Source: PLoS One. 2017 Jan 26;12(1):e0170367. doi: 10.1371/journal.pone.0170367 (PMC5268497; doi:10.1371/journal.pone.0170367)
Supplement: S1 Dataset — (ZIP) [file pone.0170367.s002.zip › Data/Data - Interview transcripts/F5.doc]

Interviewer:	…you can give us, so do you think we can just start actually by just having you tell us what you do and then what the foundation does specifically?

F5:	Okay, so I manage the health portfolio for the our Foundation. Our Foundation is a philanthropic foundation that funds four key program areas which are health, education, early child livelihood, and water and sanitation, and then there is a small portion of the budget that’s kind of allocated to humanitarian emergencies.

Interviewer:	Okay, and so when you’re looking for these organizations to sort of fit that, how does the your Foundation actually select those organizations?

F5:	We don’t take unsolicited funding requests, just besides we can’t do that. We will either… kind of depending on which sort of chest you were kind of following up on, so independently look for global organizations within a kind of country context or program area that are operating and then do due diligence around there or we will have recommendations from co-funders who we’ve kind of worked with who have programs of interest they want us to look at, or some kind of non co-funding partners that are NGOs. Sometimes organizations come through that way… and sort of tried and tested and long-established partners and implementing partners and NGOs will sometimes recommend organizations that they’ve seen on the ground who they think are great and they think will align with the institute.

And then the final way in which… some of our kind of... Not a multitude, but some of our interesting projects have come about is because our company is a [people? 00:02:12] company so sometimes staff will travel to Nigeria or Tiria or wherever it might be on business and have come across organizations that they think the foundation might be interested in or heard something about and then we will come follow up that way, so there’s different ways.

Interviewer:	So once an organization comes across your desk, what sort of characteristics or metrics are you looking at to decide, “Okay, yeah, we’re going to move forward with funding these people” or “No, maybe this isn’t a good fit?”

F5:	So, unfortunately, the answer is it depends. We’re not industry funders so we have an ability to be kind of quite flexible with the organizations that we’re funding and if something is brilliant that we think is really interesting and have great potential that’s going to skate over application or have other bigger donors looking after it but not able to respond so quickly because of their funding gap, then we may take them onboard for a 12-month period because we don’t want to lose that opportunity.

There’s a process so the first stage of the process would be a… without having those details on the project, of course, [inaudible 00:03:39] organizations are looking together: its strategy, what is its process, where you’ll look at an organization’s financials. You’re going to check their registration information… You’ll do a compliance check on the board members at HQ and if they have a local board there and local board members.

And then, look at who their funders are and we’ll ask them to provide two referees as well who would advocate for the work the organization does. Is it somebody who works for the organization or is it a board member? Usually, people either select co-organizations or they select co-founders or they’ll select research figures that they’re working with, so that would be the first stage.

Then, the second stage is that within the health portfolio, there are different areas that I am looking at in terms of organizations recently developed 2014 strategy and they are quite broad in many ways depending on how you interpret both words so we’ll look at funding things that are at the pilot stage and there might be kind of high-risk investment for other funders, but we won’t fund something at the pilot stage *and* fund something at the replication stage because if we funded it at the pilot stage, then here is someone else looking after it to see what the results are going to be and take it on…

We’ll fund programs that we think are kind of innovative without falling into the trap of pilot vices. We’ll fund programs that work particularly in that sort of maternal-newborn child health and particular to adolescent sexual reproductive health, but then we’ll also fund programs that are working in like a NTD space, kind of unparticular, and then under that, all of that fits under an umbrella that is also kind of broad so you have to un-pick it case by case of, like, grants that are kind of health systems strengthening or looking at job supply chain, looking at capacity building and developing the skills of pre-existing health workers or community health workers, so it’s not an easy one to give you a one-to-five answer ‘cause it really does depend.

Interviewer:	No, certainly. A couple questions about that, though. When you’re talking about these referrals or recommenders, you mentioned that in some cases a co-funder, in other cases it’s a research affiliate, and what type of outcome data do you find particularly moving or… persuade you?

F5:	Are you talking about for a referee or are you talking about for a concept pilot, ‘cause for referees, it’s… maybe they’ve done a couple projects in various countries and they worked doing some of the M&E or KPI at [redacted]. So, in that instance, that’s just kind of checking the basics. It’s that affiliation experience and what their experiences have been working with the organization and getting the kind of data that they need. In terms of looking at... You’re talking about indicators?

Interviewer:	Yeah! You talked a lot about registering, like just making sure it exists; it’s not phony. It’s doing some sort of, on a very basic, intuitive level…

F5:	[crosstalk 00:07:24] to an extent, yeah.

Interviewer:	Yeah, but then when you sort of charged it deeper, in looking, “Okay, so there’s a bunch organizations that all meet those low-level criteria.” What differentiates in terms of outcomes or other things the organizations that you would pick up or not pick up?

F5:	… Depending on what the project is, one is understanding what their exit strategy—what an organization’s exist strategy is— and an exit strategy could be, “We’re not sure that we have one. We work [inaudible 00:08:02] and we’re looking at it in 10 years.” Or, an exit strategy could be, “It’s going to be 10 years because we kind of dig deep, holistic programming and that’s our module,” but the main points for us is to understand how an organization had thought about it so I think that’s a key part of the initial discussion process.

The other kind of key part for us, before looking at distinct KPIs is that they have a broad or a broadening [inaudible 00:08:37] during the lifetime of the grant funding base and that the organization is not putting itself at risk of being increasingly disabled because one funder pulls out or has a reduction in budget. I think that’s kind of the key ones for us.

And then in terms of looking at, anticipating KPI achievements that would make a project really exciting…? Really sorry. It depends. *[laughter]* You might have a NTD master administration program. That is always going to be an incredible ‘bang for your buck’ and your cost or beneficiary piece is going to be kind of very palatable for the board there and you have a kind of three-strategy that’s kind of astute in overall prevalence and you kind of got a long-term impact there and you might have other programs that are *far* more expensive on a strict cost or beneficiary model but they’re looking at developing a pediatric nursing cadre that will then go on to train colleagues back in the country of origin or set-up specialists of knowledge health.

So, yes, we’re definitely are looking at impact measurements and yes, we’re definitely looking at supplier beneficiaries and end beneficiary, but the different types of impact that we’ll fund will just depend on the nature of the project and sometimes you’ll have projects that don’ have... They can’t measure the impact. It’s too early on in the program in the life cycle of the broader project but you can look at what the outputs and hopefully an outcome or two.

Interviewer:	So obviously there’s lots of different, like you say, outcomes depending on the nature of the project, who they’re working with, what the goal is, but do you ever differentiate between types of evidence like if you’re looking at a particular outcome, “Well, that number was arrived at... using longitudinal tracking or that number was arrived at...” and actually compare it with a counter-factual, like a control group, where that number’s compared to a randomized control group? Like do these more academic levels of rigor, do they factor into the decision or it’s not thought about as much in that way?

F5:	It’s not done so much in that way, so what happens is if we’re supporting a project that we know what that project needs to establish a strong evidence base, and they might be doing it through a RCT. They might be doing it through a mixed methods approach, like an M&E heavy programming because there’s a proof of concept that they want to do as an initial proof of concept or a proof of concept that the idea is scalable and replicable, so you might be looking at different pieces.

Then, I think then for us, very happy to be involved in generating a stronger evidence base and very happy to really think about how much that costs. At the same time, not happy to be funding the kind of project that wants to do a pilot but that evidence exists already, so you have to kind of provide a rationale for why you are wanting to generate an evidence base that already exists. It’s just that that service isn’t being delivered in your context.

Interviewer:	Okay.

F5:	And I think that’s a key issue, in the health portfolio anyway. I’m kind of particular to un-pick between different applications as saying they want to do pilots or want to generate that kind of more robust evidence-based proof of concept when that concept has been proven. We know it works. So, is it the best use of funds at this point to spend that money on another RCT with another P-value that’s robust to demonstrate that this works or is actually about implementing the program and them monitoring it and evaluating it in a different way?

Interviewer:	Okay. See, we were actually introduced by someone from this organisation.

F5:	Yes.

Interviewer:	Chatham House Rule! Can you describe that process a little bit, just coming to fund this organisation— learning about them, learning about their work, and then what made you decide, made your Foundation decide to provide some backing?

F5:	Yeah, no. I can tell you the backstory. I wasn’t here then.

Interviewer:	Sure.

F5:	So, if I remember correctly, this person from a health organistion… That’s another way that it’s happened a couple of times—with conferences or what have you— is that this guy I think met our CEO *either* at this conference or the other conference.

Interviewer:	Okay.

F5:	And so that was the initial point of contact and the initial discussion process.

Interviewer:	Once you have an organization like this one or others on the rolls of the foundation, what types of reporting do you make them do? How do you create an accountability relationship and what’s the process for potentially, like you said, renewing funding or deciding that that won’t be happening?

F5:	If we decide to kind of go ahead and support the project and Chatham House Rule… We just kind of awarded this organisation a multi-year grant for the first time for the next phase of their expansion. Then, they’re going to go through two processes, so one is for each...

If you have a 12-month grant with us, you’re going to submit two reports and those two reports are going to be submitted against the targets and indicators that we agreed when your application was submitted and the next chance of funding that you get is dependent on the report that you submit before that next payment date is made, so there’s a degree of monitoring that happens there and then there’s the final report that’s submitted at the end of the grant giving the thoughts and overview and then the queries are going to go back and forth to look at that.

That, at the same time, will cross-check with other co-funders’ experience at having funded a project through the same cycle. I can’t visit every project. Often, there may be co-funders or different types of partners who have funded who have been able to do a project visit during that period or I have a network that may be working in a completely different space in Mali but might be able to tell me what the impression is on the ground from people working in the sector, just for an anecdotal triangulation.

Once you have excellent success to have a 12-month grant with us, then you are eligible to apply for a multi-year grant, which was how it worked with this organisation. But we’re at a point now where because we are more flexible in when reporting dates have to be, which kind of KPI an organization needs to report *within* reason, if an organization has got funds in from a bigger beast of a funder, then we are happy to align our reporting deadlines and KPI with another funder if it makes it easier and less paperwork-intensive for an NGO.

So, we try to and do that where possible, but there might be other times where there is definitely kind of KPIs aren’t... Well, it’s debatable whether it’s strictly KPI but you want that report done, so with this organisation of example, if we’re scaling up, if we’re funding them in a multi-year to scale up the model, then one part of the argument is we’ve established a proof of concept. We have an evidence base. We work to generate evidence and test models and then feed that back to the Ministry and then advocate for a policy change at the Ministry level because we’ve demonstrated that our model works. If that’s what you’re articulating and that’s what you believe is happening, then in the Ministry in Tiria you have to show how the Ministry is starting to get onboard with something rather than just, “This is the number of people we trained. This is the reduction in child mortality. This is the improvement in facility-based deliveries,” whatever your indicator may be (or indicators may be).

We then also need to see, if you said this was going to be the exit strategy, then that’s not seeing how that’s going to look. Even if that Ministry’s involvement is five percent is their cost being covered through donations in kind, whatever it may be, we need to start seeing that profit.

Interviewer:	So that’s interesting what you were saying about aligning reporting. A lot of practitioners—we’re talking to practitioners too as part of this research—and of course there’s that typical complaint of like, “Oh, man! Like all these donors, what they want is not harmonized. The time scales are different. Their outcomes are different. Couldn’t there be a way where this would be more streamlined?” and it seems that you’re sort of doing that already. Informally…

F5:	Well, trying to. It’s easier said than done as well because you’ll have to… We definitely are trying to. We’re aware of it, but every funder has their own board they have to report to and you have to kind of report in certain ways, but a market of monitoring tools allow you to do that. I certainly don’t see any reason… If everyone’s funding the same project and taking different pieces of that financial pie, why can’t you have the same KPI?

Interviewer:	… Can you talk a little bit about examples where that’s actually been quite seamless, you’ve been able to do that, or examples where “hey, we tried to sort of align ourselves but this other foundation wasn’t interested” or it didn’t work out? What’s been the difference maker?

F5:	Sometimes, it’s actually down to the organization, the grantee. It’s like people [apart? 00:20:26]. Some think it’s great and really helpful to have the reporting schedule all happening about ‘cause it’s kind of the same date too. You kind of clean up one. You figure out, un-pick what happened in the last six months of your project’s cycle management, look at what the challenges were, blah, blah, blah, and then kind of submit the information to both. Others find that idea really stressful and don’t want to have to report to the donors at the same time. They’d rather have your report be part of the product.

Interviewer:	Okay. When you’re looking at these KPIs, so a lot of them are sort of… You are seeking out evidence of effect or evidence of no effect. What’s the “How many kids were trained?” Either you hit the target or you didn’t quite hit the target or you exceeded the target, but often KPIs are not structured around possibly trying to identify unintended consequences or harms or adverse effects.

Do you ever, as a foundation, think about the potential ‘cause we’ve had high-profile cases in the past where we have these very well-intentioned programs that actually, unfortunately, not only measure our ineffect but actively cause harm? Do you ever think about how to proactively identify that or look into that?

F5:	We don’t have a system of proactively identifies that in a KPI, per se. Depending on what the project is then you might incorporate a kind of KPI that either looks at that in the KPI or asks that to be clarified in the application stage of what kind of conflict mediation there might be. And often, that is something that comes under a reproductive health grant and whether one course of action affects family planning, how you’re making sure that’s not creating domestic disharmony and putting people inadvertently at risk, that that’s been factored in.

Then in terms of reporting, we ask that it’s subjective and it’s a subjective report… Not subjective like… a kind of free-text section—sorry—to report on what kind of challenges that they’ve had or lessons learned and that’s and opportunity for an organization to see impact. But essentially as well, I think one of the things you have to accept when you’re funding projects— and I’ve drawn many argue it— is that reporting on an application to its owner is an honor-based system.

Unless its owner goes there and goes through the books and has a look through it and actually kind of go through and cross-check the numbers, you’re reporting it and you’re reporting it honestly and its owner hopes that you’re going to report it honestly… and the only real way that you can get a benefit really if you’re going to be really harsh and massively cynical about the project, about understanding what truly happened with the project on the ground, is paying for an independent evaluator.

Interviewer:	Okay, so that’s interesting too.

F5:	In a past life, I used to be the person that was writing the reports to donors. You report back the M&Es as best you can and there might be gaps in something that went wrong and maybe it’s because there was a drug supply issue because you were short-staffed on the logistic team and therefore there was a hiccup, but does anyone write that in a report? No. They talk about the seasonality and access. You’re not always going to get the absolute truth and it’s not necessarily… Well, it’s not always tricky necessarily, but that you’re going to get every ounce of truth, you get reports over 40 pages long and no one has to time to read 40-page long reports. You’ve got to find that balance in there somewhere.

Interviewer:	So, often we talk to practitioners and we ask them, “Why do you do research?” Obviously, they’ll say things like, “We want to…”

F5:	Sorry. You’re breaking up. Say it again.

Interviewer:	Oh, sorry. I said when we speak to practitioners, often when we ask them why they do research, you get answers like, “Well, we want to make sure we’re having…”

F5:	I can’t really hear you.

Interviewer:	Let me try moving.

F5:	How about you move into [Jack-heave? 00:25:14]?

Interviewer:	Okay.

F5:	I think you’re just [inaudible 00:25:19].

Interviewer:	Is this better? Not really? Potentially?

F5:	Still can’t quite hear anything if you can hear me.

Interviewer:	I can hear you fine actually. Just quite clear...

F5:	Ooh, ooh, there you are! You’re back.

Interviewer:	Okay. Great. Sorry about that.

F5:	Go for it.

Interviewer:	I was going to say so we talk to practitioners often and we ask them why they do research and they say things like, “Well, we want to make sure we’re having an effect. We want to improve our programs,” but often they say, “We do what we’re asked to do, either depending on who the audience is, whether it’s the Ministry of Health or whether it’s donors.”

So given that, how much responsibility do you think… or in what ways do you as a foundation think about your responsibility as a agenda-setter in terms of what types of data gets collected and how that’s used in decision-making?

F5:	I think from our perspective, we try as best we can to actually not put those demands on organizations. The way in which we work is actually an organization will apply for funding for a project of which we’ll fund a bit, and then the funding that will go for that project is unrestricted within the project. We don’t care which points you want us to fund. It’s up to you as an implementer to decide what you’re going to measure and if we have any questions about it, then we’ll ask you, but it’s not for a donor to prescribe how a project is implemented. You either kind of believe in a concept or a model or you don’t.

Interviewer:	Okay. Two final questions because I want to respect your time. We said 30 minutes— we’re just about there so quick ones. One is just as an organization with this sort of varied portfolio: different KPIs, different projects in different places doing different things, how do you define success? What’s the aim and how do you know that as a foundation you’re doing what you set out to do?

F5:	That’s an easy question. Thanks! I think it depends and I think it’s something that we’re trying to work on at the moment in a slightly different kind of way, but one of the things that we look at internally is if you’ve been funding projects for 10 years or 12 years or however long that period of time is and you’re been funding in various areas and various contexts, how do you cascade what you’ve learned back rather than kind of keeping it in an internal report that goes to your board?

Your team is confident in their various contention on the [inaudible 00:28:29] of carriers, but you do have a forum, per se, to take that back into unless it’s kind of a one-on-one discussion with co-funders and you’re sharing information, so that kind of feeds a little bit into that question.

I think in terms of being able to measure broadly how the programs that you fund kind of have an impact, you can’t lump them together and you can’t play a game of saying, “Well, we’ve reached this many people this year” because that doesn’t really mean anything. It doesn’t tell you anything about quality or the depths of reach. It’s actually something that I think is really hard to do in a meaningful way rather than metrics being, “Here are some numbers that got reached,” and if you have multiple program areas, then it’s more difficult to find the line then between the kind of metrics that are being used.

But, I think for us, one of the ones we like to look at (but it’s how you measure that, which is a different kind of question) but like to try and look at is what proportion of their combined portfolio is looking at that sustainability piece and working with local authority so whether it’s a Ministry of Health or a district of Water and Sanitation, but what’s that piece looking like? Because the number of projects that are very pretty and ones who have expensive RCT and the results are incredible is just completely unrealistic so that’s going to be implemented at scale and if it’s something the Ministry of Health is interested in implementing at scale because it can’t afford it is a challenge. So, trying to find that balance between an expensive research model that is proving their concept, then having that research model replicated and less kind of labor-intensive, more kind of operational research, realistic assessing so they go to a next stage of what’s that going to be at scale up and what’s the reality of that being implementable by someone who’s not Save the Children or The Wellcome Trust or whichever a big research organization are? It’s definitely a challenge; it’s not easy.

Interviewer:	Final question. Maybe this one is slightly easier ‘cause it’s...

F5:	I don’t believe you anymore. [inaudible 00:31:19]

*[laughter]*

Interviewer:	No, no! Basically what, in your wildest dreams, what would help you evaluate organizations better, like what would help the foundation sort of find organizations that meet that description and have some sort of valid way of saying, “Yeah, they’re actually doing this?”

F5:	In my wildest fantasy dreams?

Interviewer:	Yeah.

F5:	Local organizations. Kind of clarification, definitely. Too many big, international NGOs kind of going in and doing the work that needs doing—absolutely—but not particularly collaborating or strengthening local organizations other than a kind of tokenistic way. I think that would be my sort of bug there and also… (I want to articulate better.)

And then within that as well, more direct, personal ties with different kind of people in the Ministry who are receiving the capacity building element of a grant, whatever that may be, because actually when you kind of get to the forum and speak to a lot of these guys, the capacity-building has completely inappropriate. They haven’t utilized existing capacity and they haven’t left anything behind after a two-year, three-year program has finished, but have improved the work that was being done without that facility base, that international team there, and I think that’s a brilliant miss-up and if I could have the funding and the time to do a tiny bit but every grant I have to find that out. That might be one of the things I...

Interviewer:	Do you guys fund M&E specifically?

F5:	A couple of times, we’ve been kind of M&E specifically, but that’s only really if it’s like an encouragement grant, kind of top up on an existing grant, so for example I’ve got one I’m looking at the moment with an organization that’s been funded for a project in Zimbabwe. It’s been funded for three years. I think what they’re doing is great. I think they’re terrible at measuring it and there are ways in which that they can measure it that aren’t rocket science. You either kind of know how to do it from that MPH type grant where you look at questions you’re trying to ask. The classic kind of family planning level one of serving this number of people, how HIV is transmitted compared to numbers previously and we gave out this many condoms and this many people are coming for HIV testing, but nothing on actual… nothing on equalization or negotiation skills or data collection, those kind of things and those kind of [inaudible 00:34:44] a couple where you could go out and you meet the projects, you meet the team on the ground and have a big conversation about what would be helpful information to generate so that they can finagle other funders with a bigger evidence base then we’re not there forever, then that top-up might target any needs in particular. But, otherwise, it’s unrestricted and they can assign it to M&E or assign to… whatever they need to finance.

Interviewer:	We’re good. I think I need for a second here before I actually let you off the phone, but just as someone who’s doing this on evaluation, that’s interesting what you said. You said you *know* that they’re doing good stuff but they’re just not measuring it well. How do you know they’re doing stuff if they haven’t measured it? What’s that…

F5:	So what that’s kind of based on… discussions with different partners on the ground about how this project is operating, kind of speaking for some of the other service users about the value that’s placed upon it and looking at the numbers that are attending and how those sort of numbers grow and are growing. It’s a bit like in that kind of scenario, we’ve got strong, collaborative partnerships with different organizations and local CBAs to kind of deliver it so those pieces are working well and doing it quite cheaply which is also helpful, but seeing that they’re answering maybe some of the ‘what’ but not quite being able to answer the ‘how’ and not perhaps really understanding all of the kind of [inaudible 00:36:45] of the project that they’re delivering which might in fact lead to a slight change in how they might deliver the project or any useful information to share with the education sector that they’re kind of working closely with but aren’t; they’re not an education NGO so there’s that.

Interviewer:	Cool.

F5:	And actually many may prove that what you are thinking is there isn’t there at all, but the question hasn’t quite been asked yet so it’s just not clear. One part of the puzzle was clear but the second part of the puzzle wasn’t completely clear.

Interviewer:	Cool. Well, thank you so much. This has been a really lovely conversation. Thank you, thank you, thank you and we’ll sort of…

F5:	I’m sorry so many answers were “it depends” because that’s really hard to analyze in your research.

Interviewer:	No, but I think that that’s… I don’t know. We have to see, like we like things that just… I think nuance isn’t necessarily a bad thing as long as it’s not used to deliberately off the skate, like if things are complicated, then they’re just complicated. That’s just real.

F5:	Yeah.

Interviewer:	Yeah.

F5:	Did you know, by the way, the Global Health Network?

Interviewer:	Yes! We did. We’ve been trying to track down some folks in Up the Hill in that field for a bit so, yeah, you’ve actually just reminded me to follow up on that. It’s just so spectacularly…

F5:	Do you have an email address, ‘cause I’ve got one for Judy Lang. I can forward it to you if that’s helpful.

Interviewer:	Yeah. She’s actually who we’ve tried to contact and so maybe in a couple of days we’ll just say, “You just came up again!” *[laughter]* People are busy so we’ll give her a few days to respond. Yeah, thank you. It’s a spectacularly fragmented university. Maybe all of them are, but… I thank you very much for pointing that out. Really appreciate it.

F5:	No worries. Well, keep us posted in the next few days [inaudible 00:38:43] of the recent year because there’s two or three email addresses for them and I have them. It might be helpful. They were always kind of… We’ve kind of entered discussions with them about funding some of the work that they were doing, but it was too purely research network to fund. It’s a bit too far away from what we mandate to fund, but what was really interesting ‘cause the kind of tools that would have been a bit of a godsend out in the field, but it was that “how do you get local health providers to generate some of this evidence?” and “why does some of this evidence have to be quite so stringent in terms of how it’s done and what’s done depending on what you’re actually measuring?”

You can be as stringent and robust and John Hopkins, London School-esque about getting that kind of data collection. At the end of the day, you’re getting local staff to collect that data in the field so it’s not always as wonderful as you think it is, so it’s an interesting kind of alternative. You could actually get your local healthcare providers who are looking at a problem within their own clinical setting that they want to try and research and think about doing it themselves because maybe the approach you get collecting that data is different and it’s kind richer for it because they have this local network. I don’t know. I think it’s interesting. You really reminded me of them when you sent me the email because it made me start thinking about them again. But, I hope you get to speak to them.

Interviewer:	Yeah, us too… I kind of was like you. I was an implementer and now I’ve crossed over temporarily into academia and it’s like a little bit... everyone’s concerned about the P-value. It’s like, “Will this actually…?” Not taking as much of a holistic view on how we measure things, so yeah, and use measurement.

F5:	One of the things I love is that we’ve got the P-value and the confidence interval is 25%. I’m like, “Great! It’s still one in 20.” *[laughter]* Like that’s not significant. There’s other ways to find that grant’s meaning. But, is there any…

[REDACTED]

F5:	[inaudible 00:41:53] for itself again. They were… a bit like this other organisation in some ways, but with the M&E things you were talking about earlier. They were really interesting when I got out there because you have these incredible brains who are coming up with this sort of peaceful M&E and data collection and now I think it’s got figured out a bit more.

But at the time that I got there, it was just like this is all peaceful and it’s brilliant. The Ministry of Health can’t do this! *[laughter]* This isn’t like peaceful, love-y, wonderful, rich, you can prevent it. You can kind of show that your model worked, but if you’re talking about sustainability and scaling, you’ve got to work out how that plugs into the DHS and which bits will plug in because you might be thinking about that in two years’ time. And it’s that kind of balance, which I think is always quite interesting to notice them ‘cause everyone’s encouraged… It’s really nice to understand a little bit more and a little bit more and a little bit more about your program, how you can make it better and you have all this information. It’s like, “Oh, that’s nice to know but I haven’t done anything with it because I can’t because that’s not what my program is. My program is ‘X’.” It’s an interesting one to try and get that balance, I think.

Interviewer:	Yeah, definitely. It’s hard to scale up for the public system and you have to do it, but it takes longer and… Yeah, it’s tough.

F5:	Who else are you trying to get interviews with as far as funders or implementers?

Interviewer:	What we’re really actually struggling with is we’re trying to talk to the Ministries of Health. I think we have a good set of practitioners… the research was funded by an organisation and so it’s a lot of these franchises and people who are sort of in that radius of people, so like these two guys, and then trying to connect them to people that they have been funded by so like you, but then we’re also trying to get the Ministry perspective because... It’s so shocking to me a little bit about things like this large Forum is you have these people all come together to talk about health and it’s like well health is a right, it’s a public good, and there’s not a single public sector official sitting in this *room* and yet we’re all talking about scale up!

F5:	Oh my god! Do you know who you need to speak to?! Have a look at Google and then I can do an email chain. Have you heard of…[REDACTED] It’s a kind of public-private network with this name?

Interviewer:	No.

F5:	It’s is kind of programmed by this slight legend [REDACTED] and I think it’s in the process of becoming its own 501 so these people have been creating this platform and this network of bringing together public-private sector and looking at the role of mobile phone technology and training full access to accelerate, improve quality of care for frontline health workers, but what she *does* have is a great kind of network with different Ministries so I attended a meeting looking at ACT and... [inaudible 00:45:59] systems and just rebuilding of what they currently do, and around the table with us there were about 50 people there—NGOs and [inaudible 00:46:08] some sectors by telecom, the IT magical things I don’t really understand, and a bit that’s actually funded, but you also had live representatives of the Ministries of Health, from Guinea, from Mali, Liberia, Sierra Leone, Ghana… So I think she might be a useful way to kind of have that introduction.

The other group — have a look at Google and tell me if it’s helpful— who always have access to different Ministry members is a network of funders

Interviewer:	Okay. That’s great. I’m sorry— I’m just writing both of these down. Yeah, I will give both a Google. Now that you’ve mentioned it, so yeah, I think [REDACTED], actually, that is launched back in Liberia but I haven’t looked too deeply into them. I definitely will now. And the net folks, I hadn’t actually heard of, so that’s great. Thank you so much.

F5:	I hadn’t heard of half these people before I started in this sector!

*[shared laughter]*

F5:	Yeah, those are the two I can think of off the top of my head. I’ll have a little think, but if I was going to try and track somebody down, I’d probably go to one of those two to help me get some kind of inside scoop.

Interviewer:	Great! Great.

F5:	Doing this kind of research piece is interesting, but if you don’t have the Ministry’s input, it’s a one-sided bias research piece, how many these things can be.

Interviewer:	Yeah.

F5:	These conferences, what have you… Okay, great.

Interviewer:	Cool.

F5:	Well, have a look and let me know and I’ll see what I can do. I can’t promise, but I can send an email.

Interviewer:	Yeah, no, really appreciate it. Thank you so much. I’ll do that today. Cool. Alright, well, I’ll let you get back to it.

F5:	Okay.

Interviewer:	Thank you so much.

F5:	Okay. [inaudible 00:48:27]

Interviewer:	Alright, cheers. Bye.

[end of recording: 00:48:40]
