# Supplementary material for: Supporting Better Evidence Generation and Use within Social Innovation in Health in Low- and Middle-Income Countries: A Qualitative Study
Source: PLoS One. 2017 Jan 26;12(1):e0170367. doi: 10.1371/journal.pone.0170367 (PMC5268497; doi:10.1371/journal.pone.0170367)
Supplement: S1 Dataset — (ZIP) [file pone.0170367.s002.zip › Data/Data - Interview transcripts/F6.doc]

Interviewer:	Great. Okay, [redacted], so we just wanted to start off with a couple of easy questions just to get a feel of what you guys are doing. Just for the record, can you tell us about what you do for Your foundation?

F6:	I head the global health group, which is quite small. We’ve been essentially learning from close to 600 fellows that work in healthcare and gradually evolving a vision, if you like, about how healthcare needs to change or the health sector needs to change, partly because healthcare systems largely fail in the 3 or 4 billion people at the bottom of the economic pyramid and because the people in the upper part of the economic pyramid, their health systems aren’t designed to deal with multiple chronic disease, and that tidal wave is going to sink all of those healthcare systems if it doesn’t change.

The core effect, of course, dealing with those are prevention, behavior change, proper diet— things that hospitals and doctors aren’t really very good at— so we need new delivery models that are cheaper. We need the whole leeway, so the whole set of new toolsets and new service models and we have found good examples of those among the fellows. We’re now organizing them into an ecosystem that could actually demonstrate these new models and our goal is actually to tip the system, or to be part of tipping the system into something that we call “*vitalness”*: wellness, not illness care.

Interviewer:	Okay. Wow, that’s really great! You mentioned lots of different things and it sounds like it’s a very holistic system that you’ve created with Your foundation. I was just wondering if you can give us a one line very pithy summary of the mission of the global health group that you work with.

F6:	Well, Our foundation believes in what it calls “frame change,” which the way you actually move the world into a better place is to change the frame—the mental framework that people think about a problem— and then it gets easier to actually see how that happens.

And so, our frame change is we need to stop thinking about illness care and institution-based solutions and we need to enlarge that vision to think about a much more… a healthcare that’s centered on the whole individual and it’s really not just about illness care. It’s not even just about avoiding illness. It’s about people being able to function at the top of their ability which we call “*vitalness*.” If you want to actually see a fairly coherent but quick sort of picture about how this works out and things, you can go to our website which our landing pad for engaging both fellows and now companies and thought leaders in this transformation.

Interviewer:	We’ll definitely look at that. Thank you very much for that. Okay. Sorry. It just seemed to cut off a bit. Can you still hear me?

F6:	[inaudible 00:03:37]

Interviewer:	Hello? Hello?

F6:	Yes?

Interviewer:	Yes, can you still hear me?

F6:	Yes.

Interviewer:	Okay, great. Sorry. Can you give me a bit of a summary… I’ve looked at your site, very briefly, but just in your words, can you give me a summary of how your organization goes about trying to promote vitalness and making it possible and creating this frame change that you’re talking about?

F6:	Well, there’s not particularly a perfect roadmap. One way to think about it is Our foundation has been identifying and supporting and promoting social entrepreneurs now for 30 years [REDACTED]. And sometime in the last decade, that whole concept went mainstream, so in some sense that’s the frame change, right? We essentially said it’s not just the companies that can do innovation. Individuals can do innovation that improves society. Everybody can be a changemaker. Excuse me one second. *[goes to answer phone]*

Interviewer:	Yeah, that’s alright.

F6:	Hello? Hello? Sorry— cold calls.

Interviewer:	No, that’s alright. Go on.

F6:	So, you can take that as a frame change. I was personally involved in [REDACTED]

Essentially, our goal is to demonstrate the new ecosystem, demonstrate that these things work, that they’re affordable, that they actually have better outcomes. Get [lancer? 00:06:06] articles published by academics that study what we do, get journalists and other media figures to pay attention to it and write about it, and then figure out where there are inherent business models that can help it scale and we actually think that we know them.

I’ll just give you an example. It turns out that if you focus on wellness interventions, let’s say with the employees of a big company, some of the immediate benefits are actually kind of captured by the company in the form of higher productivity, better employee retention, fewer accidents, whereas the longer term benefits are captured by the individuals. They don’t get diabetes. Therefore, the company (once they understand this) has an incentive to expand the program and support it and scale it.

In effect, healthcare becomes an investment, not an expense, and insurance companies are beginning to understand that… They already sometimes try to coordinate care so that they limit their risk— the price risk. … They’ve been eliminating risk. Now they’re beginning to understand if they take a more active role, they can actually reduce risk and therefore it’s in their interest to be promoting wellness and they don’t know how to do it yet, but practically every insurance company now has a wellness website. So, we’re trying to partner with insurance companies and show them how to do it. And the same thing works at the level of schools, if you talk about private schools. They have to compete for parents, for students, so if you can do nutrition and e-warming and simple things like that... I’m talking about schools in developing countries now. And guess what? The students learn better and they’re more active and their parents notice and the enrollment goes up, so other private schools wants to do it too.

And so, again, it’s just like the employers. Some of the benefits are captured at the institutional level and some at the level of the student, so you have a business model with private schools that can help the whole thing to scale. So, we try to identify that where there are inherent partners, that once they understand can take the whole model and run. Those are some of the pieces of how we hope to be part of tipping the system.

And the thing of course is there’s a whole set of new technologies that are actually gonna make it possible: very low-cost diagnostics that people can do themselves and don’t have to go to a laboratory, cloud-based analytics, and personal coaches which sometimes will be frontline health workers and sometimes will be a smartphone, particularly a smartphone that can be your reminder, your coach, your... the thing that gathers all the census data and sends it into the cloud that reminds you that you haven’t had your shot today, whatever. Every phone is gonna be a smart device in three or four more years so that’s actually very convenient.

… And some of our fellows have been developing advanced nutritional supplements that actually work, not the kind you can buy over the counter because they’re mostly fake. But, [paleo? 00:09:53] available supplements really do work and have amazing health outcomes, wellness outcomes ... There’s some new ways to measure a person’s nutritional status very quickly and simply so that eventually we can see consumer demand beginning to turn around the food industry too, which will be key to this.

And if you’re talking wellness, you have to be worrying in developing countries about clean water and sanitation. That’s actually part of the wellness solution too. So, essentially, we’re trying to break down the pillars that both the medical profession and the international development community use to think about this problem because they get in the way of the real kind of solutions we need.

Interviewer:	Thank you for giving me that overview of the breadth of work that you do. It’s really fascinating. It’s really great that you guys are covering...

F6:	So at Our foundation, we don’t run projects, but we can organize them and write proposals for them and help fundraise them and then our fellows or other partners can actually carry them out, so you can think about that we’re an intellectual activity which is really about perceiving patterns and organizing people in ways that can actually demonstrate fundamental frame changes.

Interviewer:	I see and that’s incredibly important. It’s great how you guys are bringing people together and really building capacity for people who can do the work really well, so that’s really great. I just wanted to build on a couple of things that you mentioned. You mentioned that you help fellows and you help the people you work with get [lancer? 00:11:49] articles published. Now, how do you…

F6:	Yeah, that’s part of the strategy. We haven’t managed yet.

Interviewer:	I just wanted to get your thoughts on particularly published research which can be a bit scarce, particularly in the global health field, particularly when projects are starting off versus quite maybe informal evaluation or not much evidence for projects starting up, so what are your views on why have you decided to take this strategy?

F6:	One of the problems has been is that if you think about how most development projects are done, there’s no such thing as real-time data. They’re evaluated years afterwards, if at all, and the data are a little bit sketchy or else if you really do a randomized clinical trial, it’s incredibly expensive and I think randomized clinical trials are going to disappear very shortly because there are much better ways to do it, but data is important. Published results are important because the institutions make decisions on “cover my ass” published data and so one of the reasons that we think the mobile revolution is so important and mobile diagnostic tools and tablets with protocols and smart behavior change things on them and so on like that is because it allows us to actually gather real-time data.

Imagine what we call doorstep health or homecare or whatever name you want to put to it, but imagine that a health worker goes and sees the patient at their home or their school or their workplace every month and they have mobile diagnostics tools attached to that tablet so they gather vitals and other data from the patient in real-time and it goes to right to the cloud, that field entry. They’re following a protocol on that device that tells them exactly what to say and what to do and has branching points. At this point, it says, “Call the doctor. It’s an emergency,” and then the tablet becomes a teleportal and the tablet captures data on the performance of that frontline health worker. We know from GPS if she went to see the right houses. We know if she finished the protocol for each patient. If she didn’t, her supervisor calls her the next day and says, “Go back and do it again.” And so, you actually have operational data and clinical data, all in the cloud, in real-time and…

Interviewer:	Which is great, yeah.

F6:	And if you have that, and we’re actually trying to build the IT system that can do that that has a very open source, front end, easy to customize and a real data-analytic back-end. We still need another million dollars to finish building that, but in effect, many people could use and the data all gets to the same place and then you can really start to do analytics and figure out this works better here and this works better there and this medicine worked and this one didn’t and so on.

And so, part of our strategy is to do any pilots that we do or field activities that we do is to really get good data and then we have an academic partner ideally in every case that has done a research design that will analyze the data and has the credibility to write the [lancer? 00:15:34] article. That’s our strategy and we haven’t yet done that in a particular case. We’ve tested pieces of this in the field and it really does work, but…

And then if you have a digital device like that, well, then you could do some very sophisticated behavior change and patient education things too. For example, one of our fellows has built an electronic game for teenage girls that works even on low-end phones and to win the game, you have to make the right decisions for each month of pregnancy. By the time you’ve played it 20 times, guess what? You know what to do and it’s now been adopted by the governments of two different [redacted] states and three East African countries… for their [inaudible 00:16:28]. That’s a really crude game, but if you think about getting the gaming industry to really build games that are behavior change, patient education games, what a great way to engage teenagers and get them to really take charge of their own wellness, so we’re talking to the gaming industry for just that kind of reason.

*[audio drops at* 00:16:52*, resumes at 00:17:21]*

F6:	Well, we’re not so interested in scaling individual fellows because most of them don’t have all of the pieces. They have a particular piece. But if we think… Our foundation uses the term “team of teams.” We’re creating team of teams of fellows and frankly other social entrepreneurs too who Our foundation hasn’t anointed as a fellow but they’re good social entrepreneurs… and so typically, we would have a service provider or a couple service providers. We would have people who do nutrition. We would have people who are good at community engagement models that build the right mobile diagnostic or the IT tools, and we put them all together as a team and jointly they would actually carry out a project.

So, we’re designing one like that with a [REDACTED] insurance company, which will play out in [redacted] for taxi drivers and truck drivers and people like that whose work doesn’t give them time to seek healthcare… who do something that takes physical work and can be dangerous.

The idea is we’re going to deliver the care at the petrol stations. We’re working with Shell on this as well and so the idea is that while you’re filling up your rig, which takes 20 or 30 minutes, you go in and there’s a kiosk and a frontline health worker and you have her take your vitals and get encouraged to go over and buy a healthy snack rather a Coke and get some feedback. Maybe we give you a disc to play in your truck while you’re driving that has local songs, but also has reminders about HIV or things like that.

And all of those are pieces that other fellows have developed but we put them all together and we think we can show quite dramatic gains in productivity as well as improve the chances that that person doesn’t get diabetes or have a stroke nearly as soon as they otherwise would.

That’s an example of how we would… and then we will have an academic partner that gathers the data and writes it up. We’ve done similar things by following pregnant women and seeing the health worker sees them every month. We encourage them to use nutrition, to have institutional births, to do all the right things they’re supposed to do, and behold! The outcomes are dramatically better.

Interviewer:	Wow, that’s fascinating!

F6:	The cost of these doorstep health models are part of what’s attractive, so we think at scale the cost of the per patient interaction in [redacted] is about 50 cents, counting the IT costs as well.

Interviewer:	Wow! Gosh.

F6:	Means a pregnant woman, if you’re going to see her six times ‘cause she’s supposed to see a doctor at least three times so it’s every month, six months so that’s three bucks. Most families, even poor families, could afford that. And in addition to that, then guess what? The people that sell water filters and nutritional products are going to pay us because our health workers are going to have those in her backpack and sell those too.

Interviewer:	Wow!

F6:	And so, we think that we have a delivery model and a version of that will work at the kiosk or at the workplace or whatever and actually they’re going to be needed in rich countries too ‘cause older people don’t want to go to hospitals and we can’t afford for them to go to hospitals. So, somebody comes and sees them every month and they actually talk about all their problems… a nurse or nurse practitioner, whatever, and there’s a doctor somewhere in the background, but fundamentally, the cloud will be better than the doctor at saying, “Oops! This is getting out of balance. Better do something here.”

Interviewer:	I see. Yeah.

F6:	But it’s easier to test these models in poor countries because you’re not fighting the MA and all that kind of stuff *and* because when people are poor, they’re more likely to be malnourished. They’re more likely to have undiagnosed conditions that are easy to fix and so the gains happen more rapidly and are more dramatic and it’s easier to show.

Interviewer:	I see. No, it’s fascinating. It’s great how you guys are making such an impact and particularly in such a diverse range of areas as well.

F6:	Well, we’re not making as big an impact by any means as we’d like because we haven’t been able to raise money for this.

Interviewer:	Ah, right. That’s always an ongoing issue, isn’t it?

F6:	It’s a real problem. [inaudible 00:22:47] foundation if I say, “I want to do a frame change in healthcare,” they’re going to look at me and say, “Duh?”

Interviewer:	Yeah. I guess drawing back to that, like resources are quite limited and with particular initiatives that you want to get funded in your case, how do you define success? You obviously take part in a wide range of different projects with the fellows who you support, but how do you define success, like whether it be overall or whether it be with different projects that you’ve worked with? How do you measure impact and think about whether scale or community-based ownership is part of that equation of success?

F6:	Well, at any given project, there are likely to be specific outcome metrics and it’s partly on the funder and what they’re interested in too, but the real goal is actually tipping the system. When more and more people are starting to talk about wellness and not about illness care and when there’s more and more awareness in professional circles, in the media, and gradually at a middle class consumer demand level, this is what we want. Then, we’ve won and we can go do something else because the system has enough momentum to change itself.

But, we’re not there yet and we’d like to accelerate that process by getting our own… And in fact, one of the ways we’re trying to engage big companies is create a sort of round table which is focused on outcome metrics. How would you measure vitalness? Or well-being? What are the outcome metrics we want to measure because if we don’t have clear outcome metrics, we’re not going to get there.

Interviewer:	I see. And how far have you come on that?

F6:	We haven’t come nearly as far as we want because we haven’t been able to get to enough of the big companies, but we think that it’s a good pre-competitive space where you could actually have competitors around the table discussing this and it serves our purpose as well getting them to think about the transition to wellness in addition to... Excuse me again.

*[goes to answer phone, text omitted from 00:25:16 to 00:26:13— irrelevant to discussion]*

F6:	Sorry.

Interviewer:	No, that’s alright.

F6:	Anyway, we think that having a metrics discussion is a good way to get people thinking about where the health system’s going to go to and it’s a safe thing that competitors can talk about without giving away any of their IP and so we’re trying to convene some forums where the discussion is, where people pay a little bit to be part of that but the idea is that it’s a cooperative activity to try to really define, “What are you going to measure in nutrition? What are you going to measure in behavior change? What do you measure in attitude and mental energy?” and those are all important questions because if we can’t measure it, we’re not going to get there.

Interviewer:	Yeah, very true. Just on the topic of measuring as well, going back to how you define successes, tipping in the system and making people more aware of the need to tip the system, how do you measure your progress on trying to reach this end goal?

F6:	I don’t have a good way to measure it explicitly. I suppose the gauge I actually use is that I keep track of what I see in the media and I see more and more—at least in the [REDACTED] which is where I sit—it’s very clear that consumers themselves are moving rapidly away from processed food and to demanding better diets so that seems like, “Good! Something’s starting to happen there.” We probably know 30 or 40 social entrepreneurs in the [REDACTED] who are working on better diets and fresh farmed food and healthier soil so that there’s more micro-nutrients in the food, but clearly something’s starting to move there. We think it would be accelerated if you had a little device where you could actually measure the nutrient density in an orange before you buy it and we think that consumer device is about five years away.

So eventually it will happen, but we see movement there already. We see more and more of the insurance companies because they’re seeing what Medicare is going to demand of them, scrambling to try and figure out what to do, but they don’t really know what to do. We see pharma companies starting to talk about beyond the pill, but what they actually put their money towards is still about more pills. We see the… I had another quick example and I forgot what it was.

In any case, we could try to keep our finger on the pulse of what’s happening, but I suppose (and the most practical way) is if we got significant funding either from a major foundation or a group of corporations to do this work, that would actually be an important indicator that we were making some progress.

Interviewer:	Okay. Are there any particular ways with the projects that you’ve engaged with that you can measure progress towards this general change that is happening in the system and look at what impacts this particular project you’re working on is making on the system change?

F6:	Well, in any given field project, there would be clinical outcomes that people improved, so that’s easy and we would do that because we want to generate good data. The pilot is to demonstrate in a credible way... I’m not trained as a scientist, so I think in those terms. We want to generate real evidence that things work, but a pilot doesn’t tip anything and you need dozens of pilots to begin to convince large organizations and ultimately governments that this is a better way to do things close and we’re not close to that scale yet. We just haven’t raised the right money. We know what to do, we think, but we don’t have the money.

But on the broader thing, I think we’ll know when we start seeing major organizations, whether they’re for profit or non-profit—it probably doesn’t matter— who are starting to steal our language and our ideas and do it themselves. In effect, when we become irrelevant, that’s when we’ve succeeded.

Interviewer:	I see and you can’t really measure that very well.

F6:	We can’t measure that *yet*. Our foundation is doing this. It’s more advanced in doing this in what they call empathy— the idea that every kid should learn empathy because it’s key to being part of a team and that teamwork and everybody being a changemaker is the world we’re moving in to.

[Redacted]

Interviewer:	I see. So I just wanted to change track a bit with the types of questions that we’re asking about and I wanted to really zone in on the formal evaluation process and evidence gathering. I’m just wondering with your work so far, what evaluation activities does Your foundation currently undertake and what resources do these evaluation activities take?

F6:	In effect, we can only devote the resources to that that a grant gives us and typically they don’t give you much. If it’s from an international development agency, they usually require an evaluation and they’ll sometimes explicitly tell you how much you have to spend in doing it, but Our foundation doesn’t take money from governments or international government-like agencies so we usually get stuck with that. So, pretty much what we’ve done is what we can afford to do, which is to collect data and analyze it… and it’s at the clinical level basically. To some extent, at the operational level because we were eager to show that these things are much more cost-effective than traditional ways of doing things.

But more broadly, I think… I mean I do understand the amount of serial. I’m a social entrepreneur myself so I know how the impact investing community is all big on evaluating impact and I don’t think any of the systems out there actually work. To do really good evaluation, you have to do what the [Redacted] or those kind of places do and it’s very expensive. They essentially independently look at the whole process and do their own control groups or ‘before and after’ and it’s just... You’re not going to be able to afford to do that for very many projects.

And short of that, I think it’s about simply being intelligent about what’s a meaningful marker that you can actually capture. In health, it’s easier than in many sectors usually because there are behavioral things you can measure. So, for example, the outcomes we looked at in we tracked 600 pregnancies with one of the early doorstep models. We looked at, “Did they elect institutional birthing?” We got 100% of them to elect institutional birthing. The average in that community is about 50%. We measured obviously maternal deaths and child deaths. We had zero maternal deaths, 2% child deaths. This is rural [Redacted]

Interviewer:	Wow! Gosh.

F6:	We beat all the metrics so we looked at the obvious metrics and we believe it’s because of that personal contact of that health worker coming every month and chat with you in your home was actually the secret sauce. That’s what we did that was different so we feel that’s a pretty strong endorsement. It’s not a randomized clinical trial, but it doesn’t take a genius to see that was certainly part of the reason for those good results.

… Depending on the problem you’re trying to solve, there’s usually some fairly good outcome metrics that make a difference. For example, I was involved in a both health and clean water project in [redacted]. The first order of metric for water is how many families sign up and pay for getting their water everyday so you can talk about penetration and percent’s in the community and that kind of thing and typically we get 30-40% of the households to sign up. That’s pretty good.

The next level is to say, “Does clean water make a difference in health outcomes?” and we actually had a Group that’s the equivalent of the Poverty Lab spend some time… They spent a year on the ground looking at that and they concluded that in fact it was clear that the incidence of childhood diarrhea measured either directly—“Did your kids have diarrhea this last month?” kind of thing— or measured by school attendants which is actually a harder number what went done in families that had water as compared to those that didn’t. That’s a reasonable measure for that. … At one point, USAID was going to give this group half a million dollars to go do a randomized clinical trial of exactly that same thing and in the end balked and said, “Look, you can't randomize this. If we get a community to agree to let us build a water plant, we can’t then assign them to a pool where we do or we don’t or we delay it six months. That’s not clinically possible. It just doesn’t make sense to do.” Geographically, randomization doesn’t work. It’s not like a pick of people where you can give them a placebo or not.

And so, they never gave the grant and it was a good example of trying to force things into this clinical trial mode that just won’t... force. But I think the thing that they did do before another larger organisation got involved was actually more meaningful. There was clearly something happening and since we know that dirty water is the main source of childhood diarrhea... and we have a large, then very likely indeed we were measuring that we were having an effect.

Interviewer:	I definitely agree with you. It’s about being sensible on the ground and seeing what works in that particular situation. There are huge challenges to conducting formal evaluations or formal randomized trials which are just not possible. On that topic, you’ve mentioned money as challenges in performing big, formal evaluations and also just being sensible on the ground. Are there any other challenges that you see being able to conduct these things?

F6:	The biggest challenge is… Well, I think there are two. One is lack of good data. Having an ability to really capture both outcome data—whatever the outcome you’re trying to achieve is—and processed data, operational data, is really key and until the advent of cheap, mobile devices, it was prohibitive to do. But now we have cheap, mobile devices and you can instrument damn near anything. For example, on these water plants, we had telemetry so we knew right away that day if the plant isn’t working or if suspiciously the number of apparent subscribers went down but the amount of water produced *didn’t* go down. That meant that guy was cheating on us—the operator.

So having good data, the real-time data of all different sorts is actually key and with cheap, electronic stuff, it’s now possible to do that but very few people build that into the design of the project they’re doing. That’s why we’re trying to build independently this mobile IT system that anyone could use. It’s interesting in that regard. Gates has spent 10 million dollars building a mobile IT system for mental health in Ghana and then they spent another 10 million building one for the same purpose in Bihar. And for about two million dollars, we could build a mobile IT system that anybody could use for any health activity or any field activity without an IT person on their staff.

Interviewer:	Wow!

F6:	The front-end is the open-source and you can configure it really simply and the back-end, it crunches your data for you any way you want and can be randomized so other people can study is as well. We know how to build that. We’ve already done the needs assessment for the alpha group of users. We just don’t have the two million dollars. But, it’s crazy because that kind of data, if every project captured good data, then third-party people can study and learn a lot, and to me, that’s the most useful kind of evaluation. You can’t evaluate something where there’s no data and if you have to set up a separate process to collect that data, A) it’s very expensive and B) you’re disturbing the thing you’re trying to do. Social entrepreneurs just don’t have bandwidths with bureaucrats to be honest.

Interviewer:	Yeah, that’s fair enough.

F6:	I think those are the real problems and that’s why I think spending money to create data gathering systems that can be generically used by many, many outfits, that would be the most valuable investment to improve evaluation.

Interviewer:	I see, so…

F6:	[inaudible 00:43:19] mixers, telemetry systems, open-source software... Those are the things that would make a big difference.

Interviewer:	I see. Was there a second point that you wanted to mention in the challenges of conducting?

F6:	No, no, I mentioned it. It was one that you perturb the system; you’d ask the entrepreneurs to do things they just don’t have bandwidth to do. Remember that the hardest thing in VOP activities/enterprises is recruiting and keeping middle management. You’ve got a committed entrepreneur. They’re smart, they’re going to do this, but that’s not the same as their supply chain manager or their sales manager. Those you have to hire competing against multinational corporations. It’s very hard to find them. If you need to hire an evaluations manager to manage this process that these people who give you the money want you to do, you can’t find that person. *That’s* the real problem. Every social enterprise scaling is impinged because they can’t get good middle management. And evaluation is just another middle management.

Interviewer:	Fair enough. I can’t agree with you more.

F6:	That’s why I think if you focus on enabling good data collection, which enterprise needs for its own purposes, then that actually gives you the things you need to evaluate it too.

Interviewer:	[redacted], I just have two final questions to ask. Now this can be a bit of a curveball, but with a lot of health initiatives despite best intentions sometimes they can cause harm to the recipients of the activities that the organization decides to undertake. … Have you thought about ways in which what you do with Your foundation could be harmful and have you found ways to evaluate that?

F6:	I have, but I’m actually not very worried because most of what we’re doing is actually preventive and I don’t think you hurt people. You may fail to change their behaviors, to prevent their illness, but you’re not going to hurt them. We’re essentially working in adult chronic disease, in childhood health and malnutrition and in maternal health—maternal/infant health and malnutrition. Nothing we’re likely to do with those mothers and babies or with the school kids is likely to hurt them.

We’re not doing surgical procedures. We’re not introducing new drugs. If we’re giving healthier food and encouraging behaviors, I don’t think we’re going to hurt anybody. An adult chronic disease… If we need to refer them to the medical system in a hospital, we will. We don’t cut off legs or do things like that. Our focus is actually on prevention and risk reduction and behavior change and through nutritional supplements and encouragement and software tools and so on.

So, I actually don’t think there’s a lot of harm we can do because precisely we’re not trying to replicate the illness care system. Is there some possibility that people prescribe the wrong medicines for somebody? Sure, but most of the medicines we’re talking about are not... They’re not chemotherapy.

Interviewer:	Yeah, fair enough.

F6:	I actually think in this space that’s not a serious issue.

Interviewer:	I can see what you mean.

F6:	There would be lots of places… And you do get into things like... So one of the things we think is important in adult chronic disease is to screen for depression because it’s usually a co-factor and there are places where being identified as having mental illness is still a stigma, so that might be not so good, but it’s not a fundamental harm and we’re not broadcasting it. It’s up to the person to decide what they say about it. We understand HIPAA laws and things like that.

So, I actually don’t worry about it very much in the areas where I work. There are places where you might worry about it, but in sectors that I think are really critical which is in healthcare, in safe drinking water and sanitation, in off-the-grid energy like solar lanterns for replacing kerosene, we don’t think there’s much potential for harm to be honest.

Interviewer:	Okay. So, [redacted], coming up to our last question now. In the perfect world, what do you think would help you do your work better, specifically what would you help do things like evaluating and collecting data better to generate the evidence that’s required to support?

F6:	You won’t be surprised if I say what I need is the money to build this mobile IT system that will collect data from anybody who uses it.

Interviewer:	That’s not surprising at all.

F6:	… I was talking with Samsung yesterday so maybe they’ll be interested, but they really sell devices. They don’t build software systems. Who knows? But if we had a couple million dollars of unrestricted money, the first place we’d spend that is on our mobile IT system.

Interviewer:	That sounds like a perfectly sensible idea given your explanation so far.

F6:	… We are going to do projects in the field, but what we’re actually trying to do something more sophisticated and complicated which is orchestrate the activities of quite a large number of people in ways that actually change the field. And it’s very hard to raise money for that. Even it’s hard to raise money for the pieces of it. So, that’s been my main struggle is you go to Foundation A, B, or C and you say, “I want to raise money for a frame change in healthcare,” and they look at you… and they say, “Well, how many patients are you going to treat? Well, fine.” Or Gates wants to do nutrition, but they only want to do it between the ages of 1-5 and they want to talk about how many million kids you’re going to reach and don’t talk to them about needing to de-worm them or get safe drinking water into those kids!

Interviewer:	I think it’s a very common story amongst people in the field.

F6:	Yes, it’s a very common story and the pillars that… You know yourself. My wife’s a diabetic and she also has lots of other issues and guess what? The specialists won’t talk to each other. And so, coordination of care has to happen at her level and she has to do it herself and that’s crazy, but that’s the world we live in.

And if you need the three different ministries to do something cooperatively in a developing country, forget it! Not gonna happen. The people who give money are pretty much… They want to do healthcare or they want to do job creation and “please don’t bother me with these messy other things.” If we’re serious about solving these problems, and that’s what I’ve been spending my career on for the last 25 years, you have to deal with the messy problem.

Interviewer:	Yeah. No, definitely true. Well, [redacted], unfortunately we’ve run out of time now and I did want to respect your time. It’s been a pleasure talking to you— a very enjoyable chat— and definitely a lot of material that we can use to enhance our research here so thank you so much for taking the time to speak with us. It’s been really interesting.

F6:	Yeah. Glad to do it.

[end of recording: 00:52:50]
