# Supplementary material for: Supporting Better Evidence Generation and Use within Social Innovation in Health in Low- and Middle-Income Countries: A Qualitative Study
Source: PLoS One. 2017 Jan 26;12(1):e0170367. doi: 10.1371/journal.pone.0170367 (PMC5268497; doi:10.1371/journal.pone.0170367)
Supplement: S1 Dataset — (ZIP) [file pone.0170367.s002.zip › Data/Data - Interview transcripts/F7.doc]

Interviewer:	Okay, great! So, F7, can you tell me a little bit about… So, the Your Foundation. What’s the big problem that you’re trying to solve? What’s the mission of your organization?

F7:	Of the organization as a whole… The mission is to give everybody the possibility of a healthy and productive life.

Interviewer:	And what do you think is the big question you want to answer about the impact of the programs that you end up funding?

F7:	… I’m assuming you know a bit about the foundation and how it’s broken down into different kind of sector areas and the area I work in health and so I speak to the health work that we do.

Interviewer:	Perfect.

F7:	But what we’re trying to achieve there is just trying to maximize the impact that we can have with the endowment that we have here and to kind of crowd in additional money, always to kind of maximize impact, and the metric that we use probably more unofficially than officially is the DALY (the disability-adjusted life year) so we’re trying to reduce the number of DALYs in the world.

Interviewer:	Okay. Do you have a favorite story about the impact that you’ve made as an organization, specifically with your portfolio in health?

F7:	Well, I think everybody looks to this particular project. I think that was our first big, big investment and it predated my time at the foundation, so our donors gave a donation of… I don’t know what it was at the time. It was at least a quarter of a billion dollars, but this is going back to 2000 and we’ve remained a majority funder of this project.

I’m actually involved in doing the estimates of the impact that THIS PROJECT has in terms of audits, resources pulled together and the vaccines and the countries that receive those vaccines, and so I think that’s probably what we would describe as our most impactful investment. If you look across the portfolio of vaccines being delivered through THIS PROJECT, a really good return on investments, so we’re talking about things that come in between 10 and 100 dollars per day averted so a highly cost-effective use of resources.

I think Our head boss over time has kind of softened his statements. He used to say that vaccines were the best investment in global health. I think they’re one of the best investments, but that’s probably the most prominent investment that the foundation has made and the biggest passion for Our head boss in particular, global health.

Interviewer:	As you’re quite aware, there’s lots of different things to fund in the health space and there’s often a lot more projects that could be funded than there are resources for funding. For the Your Foundation, obviously this one project is one of your big projects you’ve invested in from the beginning, but for subsequent health projects, how does the Your Foundation select the organizations which you provide funding for?

F7:	The organizations or the programmatic areas? Some, I guess it’s a bit of both.

Interviewer:	A bit of both, yeah.

F7:	Yeah. There is an interesting story here to tell you about the World Development Report in 1993… which supported that which was the Disease Control Crisis Project and that was the first edition of that book and Dean Jamison was the lead author. The foundation obviously wasn’t around at that time, but that book… I’m not sure if you’re familiar with it, but it kind of identified the best buys in health and legend has it that our head read that book and was kind of horrified to see that there were things that cost literally pennies per vaccine. He then used that information to stimulate his interest in philanthropy, in particular in the area of health philanthropy as well. We now fund… Well, we funded the second and the third edition of that.

Sorry. So going back to answer your question, I think Our head’s always been very much about the data, trying to drive where we should make our investments. It hasn’t been done as systematically as you might like. I think at the level of Our head boss, every meeting that you’re in, he will ask you to justify that investment— what is the cost per DALY of that particular investment? I don’t think that happens at the level of the directors perhaps to the same degree, but they have to be prepared to answer that question. It’s always been there, but perhaps not kind of out in the open to the degree that you might like.

A shift that has happened in the last five years was the arrival of someone called Trevor Mundel who manages our health team now. He’s President of Global Health. He used to be at Novartis and he has introduced a tool that tries to now assess systematically all of the investments that we have in the R&D spaces, so things that are much further upstream. (They’re not yet being licensed and pre-qualified.) But, there, we have perhaps more than 100 products and each of those products now we are systemically going through and trying to value them and saying, “What impact would they have if they were successful? What would be the cost-effectiveness?”

We’re in the process of developing this tool and rolling it out so there’s a lot of questions around whether it is actually useful, will our senior—and ultimately will our head bosses—sit down, look at the portfolio of products we have in the R&D space and say, “Some of these are just not set up to succeed and we, therefore, are going to kill them… and others are looking very attractive and so we should seek to try and accelerate their development.”

So, just to say, I think—yeah—we’re now moving toward being much more systematic, much more explicit and transparent about making these types of trade-offs using the best data available, so that’s actually a positive thing about the foundation. Now the little thing… I’m sorry I’m talking a lot, but I’ll say one thing that I think is important to know about the foundation. I’m sure you know this: this is a family foundation. So, ultimately, they decide what the parameters are, and so you will see some things that I think on occasion people say, “Well, actually, what we’re spending our money on perhaps doesn’t tally with the economic evidence,” and so probably the best example of that is polio eradication.

But, polio eradication is hard or any kind of elimination/eradication program is kind of hard to justify in terms of a cost per DALY argument. There are a lot of more intangible benefits that are tried up with those types of programs as well, so… Yeah. Just to say, that I think is almost a legacy play as well for the foundation rather than “that’s the best way in which I could maximize health.” I’ll stop now.

Interviewer:	No, no, that was really good. Thank you very much for that summary. It definitely sounds like there’s a very strong culture of the importance of data in your organization. Now, I just wanted to clarify. With the tool that you’re developing for the products that you’ve got, is that products that you’ve taken onboard as projects that the foundation is supporting or are you looking for new projects all the time and you’re going to use this tool to assess whether it’s worthwhile investing in them?

F7:	It’s more the former, but I think we’re also then gonna run ideas as well in a very high-level fashion to see whether or not we should proceed with some investments. … The underpinnings of that toward for most of the work that we do is the Global Burden of Disease data, so I’m going back to the DALYs data. That really is what Our head boss would inform a lot of our investments so…

And then, we would apply a couple of other lenses to that so we would ask, “Are other funders working in this space and if they are, is there a role for us?” and oftentimes there won’t be, so we’ll back away from those, but I guess neglected tropic diseases has often been a good example of a sizeable burden. No manufacturers are working in that space given the incentives not being aligned and so we see a role for us to step in and try to correct some of those market failures.

So, yeah, the burden data will kind of give us some indications of where we might want to go and we will begin to pressure test some of those possible interventions through a more cost-effectiveness lens and say would that make sense or not. I guess in the past our signature investments have been vaccines...

Interviewer:	Yeah.

F7:	And as I said, I think Our head boss has softened his approach to ‘all vaccines are highly cost-effective’ because that hasn’t been borne out by some of the investments that we’ve made. I think Hookworm is actually one of the examples that’s cited around here where we did make a substantial investment years ago. It was not progressing as we liked and so ultimately I think we were going to kill that program (or that PDP). I’m not sure who finally stepped in, but we decided it wasn’t for us.

Interviewer:	Right. Can you elaborate on the Hookworm story? What kind of metrics or impact measurements were you using to decide that it wasn’t a project that was worthwhile continuing?

F7:	This is stuff I’ve heard second, third-hand, so I think these decisions were taken before I joined the foundation. I’ve been here for about five and a half years, but somebody recently told me in a world before we had this tool, which is called the Integrated Portfolio Management tool—the IPM tool— there were these kind of ad-hoc examples of us using data to make big decisions and people cite the Hookworm as an example of data was used and a decision was made to, in that case, kind of close down that program.

I’m assuming it was based on the probability of technical and rudimentary success for the vaccine and it did not look like it had a great chance between 0 and 100% and the possible impact it would have so I guess that’s something tied in with the targeted portfolio. It just was not on track to have the right level of effectiveness and duration of protection, and ultimately then the kind of cost side as well and the cost-effectiveness, but I’m speculating there based on conversations we have these days.

Interviewer:	Yeah, sure. Okay. And just going back to what you were talking to with pressure testing possible interventions with the program areas that you decide on, you talked in particular about the cost-effectiveness lens. Could you expand on that a bit and are there any other major areas as part of this pressure testing which you focus on?

F7:	Definitely the cost-effective piece. Definitely that part I mentioned just now on the PTRS—the Probability of Technical and Regulatory Success. That’s something else that we are tracking through the different phases of development of these products as well. The TPP as well— the Target Product Profile. That will be established very early on in the phase of development and then kind of refined over time, but if that is looking like it’s not going to deliver a product that is deliverable, then that also will kind of give us pause for thought as well.

Those are things that I think of really… those are mind shifts that kind of happened more recently though, so again I think we’ve often been criticized for trying to find that silver bullet and not being fully aware of the challenges of then delivering those bullets if we manage to find them, so now we work both in the R&D and the delivery space much more hand in glove so we will not, I think, and I’m not sure how strictly we apply this, but I think it’s rare for us now to greenlight a major new investment if it doesn’t look like it’s going to have the type of profile that will facilitate its delivery in hard-to-reach parts of the world.

Interviewer:	Okay, great. Okay. And so, the metrics that you’ve talked about so far: DALYs averted, return on investment. What would you say are the other major impact metrics that you look at?

F7:	We’re probably overly reductionist in terms of how we approach this. I’ve never met anyone who is as hyper-rational as our head, so it probably does boil down to those. That’s not a joke. I really think he does walk that walk as well and talk that talk, so it’s those.

I think Melinda has now introduced some other impact lenses so we now have an emerging area around women and young girls, so that’s a bit different. I guess it’s a gender angle now that we are introducing, an equity angle as well although I think we’re struggling to understand what we mean by that, ‘cause I think the way in which we’ve approached that in the past has been taking a country as a unit of wealth whether it’s poor or not, and we haven’t been as smart in terms of all of the poor people in middle-income countries. We haven’t targeted our investments within countries yet as much as I think we might do in the future as economists kind of develop and many countries graduate from low-income status to middle-income status, and so that will be something that I hope we are a bit more nuanced about.

What else? I think obviously price and affordability are ones that we care about as well, so I guess they’re the flip side of the impact. We want to reduce those as much as possible. Yeah.

Interviewer:	Wow. That’s great.

F7:	… They’re different kind of factors, but if you’re asking about the impact factors, I’d say it’s probably those so they are some kind of multi-criteria that we’re applying, but those other ones are a bit fuzzy still for us.

Interviewer:	Okay. I think, from what you’ve said so far, it’s quite apparent, but for the record, can you state how the Your Foundation defines success or impact?

F7:	I think it’s achieving scale. That’s important for us as well. We want to move beyond lots of small pilot projects, so it’s impact at scale—measureable impact as scale—so measureable meaning we can kind of estimate that impact or measure it in terms of DALYs averted and I think the impact really is… also it has to be kind of value for money and we would define that in terms of a reasonable level of cost-effectiveness.

If you had asked what that level is, what that threshold is, I think that’s still such a debate within the foundation [REDACTE]

[Our head] mentioned DALYs and then he actually for the first time publicly disclosed his threshold and it was around 300 dollars per DALY averted. I wouldn’t hold him to that ‘cause I think obviously it’s his prerogative to move that up and down over time, but around 300 I’d say is probably what the benchmark is for what we consider good value for money.

Interviewer:	It’ll be quite an interesting process to go through, to define a benchmark. I imagine it wouldn’t be very easy at all. Do you have any preliminary thoughts about what kind of things you’re going to include in that decision?

F7:	Yeah, a few ideas. You’re right—it’s going to be very political and I’m not sure whether we’ll make this threshold put out in the public domain. I mean, I’m sure it will get leaked so we have to think carefully about how we do this because then there’s always the potential for gaming and we see that with this company in the UK.

So, some ideas are for us to actually go through and review some of our past investments and try to quantify the cost per DALY of those investments as well and see whether a kind of de factor threshold emerges from historical investments. And then, I think looking forward, I think ideally it’s going to be a bit informed through some interviews of course as well and trying to elicit from some of the major decision makers of the foundation what they believe their threshold is as well.

It’s complicated though because we might play a relatively big role in terms of developing these new products but in terms of the delivery of them, we’re a minor player as well so it’s hard to kind of understand where that line... The lines are very blurred in terms of our threshold versus THIS PROJECT’s threshold versus the country’s thresholds and it’s ultimately country’s thresholds that we want to support as well. We want to be sure that they’re making the right decisions.

Interviewer:	I see. I see. Hello?

F7:	Hi.

Interviewer:	Oh, sorry. I thought we just cut off there. Yeah, I definitely agree with you. That’s a very good point. So, I’m going to move onto a little bit more dry material now just in terms of how you manage and organize organizations who you fund, so once you’ve selected an organization which has a potential solution, how do you manage the funding that you provide them… in terms of, like, what do you expect from them in terms of reporting? What kind of support do you provide them?

F7:	… Well, it’s not as much as a case-by-case basis, but there is quite a bit of variation in terms of how we manage investments here and there’s variation between the programs as well. I work on health. I actually now sit on Global Development. We had a big re-organization of the foundation that kind of splits in terms of upstream and downstream functions, so those two divisions will probably have different ways in which they manage grantees as well.

My grantees are not developers. They are people who are doing a lot of technical assistance, like this company or universities, so there will be different ways in which I try and hold them accountable versus a developer. So, what do we do? Every year, particularly around now, we begin to put a pipeline, a new investment together. Those need to get approved by our superiors and then we kind of make that grant. And when that grant is made, that typically takes almost a year.

There’s a lot more bureaucracy here than I think there used to be. Things don’t always move as fast as I think people expect them to in the foundation, but then that grant agreement will have a table of milestones as well.

… Let’s say it’s a 10 million dollar grant over 5 years, then the easiest thing would be a 2 million payment every year with a first payment made upfront and some of those payments will be contingent payments. They have to deliver a satisfactory progress report and a report on their financials. They send that to the program officer who reviews it, says whether or not they’re happy with that, and if so, it releases the new payment. Some of those payments will be no-go decision points as well. [inaudible 00:22:52]

Interviewer:	Hello, F7? Hello?

F7:	Hello.

Interviewer:	Hello?

F7:	Hello. I’m still here.

Interviewer:	I think you just cut off there.

F7:	Can you hear me?

Interviewer:	Yeah. Thanks.

F7:	Sorry. Can you hear me now?

Interviewer:	Yes, so you were just talking about the milestones—some of them have a contingency set for them and that goes back to you guys and then… Sorry. I didn’t catch the last bit you said there.

F7:	Yeah, and some of those are really even stronger than that, so there’ll be no-go decision points as well. With some of the manufacturer’s investments, which we typically do through these product development partnerships. If we’re not seeing enough progress with those, then... and this doesn’t happen that often to be honest from what I gather. Then, we will kill that grant. That grant will stop then.

So, those are some of the ways in which we do it. I think the reputation the foundation has is that we’re a little bit micro-manage-y when it comes to our grants as well, though, unlike for example, this large Trust which will probably require a high level of work upfront in terms of getting in the proposal but once you’ve got it, they leave you be. I’m kind of stylizing a bit much there, but whereas the foundation, I think we have a reputation oftentimes for unfortunately… Meddling’s maybe the wrong word, but we don’t just step away and let the grantees get on the work.

Interviewer:	It sounds like you keep a good tab on, as a foundation, the organizations you work with. I’m quite interested in the milestones in the grant agreement from the beginning. What kind of things do you look for to try and assess whether the organizations have met their milestones?

F7:	Yeah, This is where there’s a lot of variation, I think, because we would like obviously the grantees to have a strong theory of change and a strong theory action as well, for that to be laid out in terms of a results framework. And then, for that results framework to produce a results tracker and that then kind of identifies what is this vision of success and what are some of those leading indicators that suggest to us and that grantee that they are on track to meet that vision of success?

It depends really on the nature of the investment. But, starting with a really strong theory of change, I think that’s where a lot of the grants frankly are lacking. I think I’ve seen them improve over time, but it’s that and that requires a lot of time upfront and I think often... Sometimes it might be overkill if it’s a fairly defined piece of work that they have a lot of control over…

Interviewer:	Hello, F7?

F7:	...but where there’s a series of, “If X happens and ‘Y’ happens, what happens then?” Ultimately, you’ll get this. It’s helpful then I think to have a well-thought out theory of change.

Frankly, we don’t have program officers who are extensively trained in M&E and we do have a small team of M&E specialists who support the program office here, but again it’s a small team and so they have to be pretty selective in terms of who they work with.

Interviewer:	So this small team of M&E, do they provide direct support to the organizations?

F7:	Yes. Exactly. For example, I’ve been making a big investment this year with this company and so I had some colleagues really working quite closely with this company to get their grant to take shape.

Interviewer:	Wow, that’s great. Can you give me an idea of how many people and how much money it costs to run the M&E team at the foundation?

F7:	Well, I think right now…

Interviewer:	Hello, F7? Hello? Hello? Hello? *[long pause till end of audio]*

*[begin second recording]*

Interviewer:	…brought that up. Okay, sorry about that. So you were talking about the M&E team that you have. How many people and how much does it cost to run it?

F7:	The costs, I don’t know. I think it’s probably only about half a dozen in the central team and there may be the same number embedded in some of the program teams as well, so we have a pretty loose approach to M&E at the foundation and I think right now they’re revisiting that function as well. For some reason, I’m not sure… It doesn’t seem like the function internally is valued as much as it should be.

In terms of how much, I don’t know, but the staff I imagine if it’s just looking at salaries you’re looking at a million, two million a year. Something like that.

Interviewer:	Yeah, cool. That’s fine.

F7:	Which is peanuts compared to… It’s mind-boggling that we don’t build that muscle more in the foundation, but listen. I’m a health economist and I’m always amazed that they are so few health economists in our foundation that’s trying to spend 4 billion dollars a year, so there you go.

Interviewer:	Exactly right. I couldn’t agree with you more. So, we’ve got two more questions, F7. I just want to ask a question about harm and how the foundation conceptualizes harm. Sometimes, particularly new health initiatives, when they first start out, despite best intentions cause harm to the population they’re working with. As a foundation, is this something that you’ve thought about in terms of the foundations that you fund? And if you do, how have you dealt with that?

F7:	That’s a great question. It’s possible that we have thought about that, but I have never heard any discussion of this. I’m sure it’s come up in meetings about the unintended consequences of some of our investments, but I don’t think we’ve ever thought of more seriously as a foundation and thought about putting maybe a bit of a physician paper or a policy in place. I don’t have a good answer for that one, Jenny. I’m trying to think of examples when this happened.

Here’s a good example and it’s a tragic one, but obviously with the polio health workers in Pakistan and there were a number of them that were obviously murdered in recent years. My parents have asked me, “Well, what does the foundation do in those situations?” because we are obviously one of the biggest supporters of the polio eradication program and I don’t know. I assume that might be an extreme example where — and I’m again totally speculating—I don’t know if we do things behind the scenes. To do something in a very public way might actually be even more disruptive and make them bigger targets. Great question. I don’t know…

And then, there are all these kind of controversial areas the foundation works, so obviously in agriculture we do a lot of work in to the GM as well which obviously divides opinion as well. I just get the sense that I haven’t seen this kind of forum back down on some of these things so it’s our view versus other people’s views and then we mobilize our communications team to kind of support us. … We’ve got a fairly business-like approach to some of the investments that we do here.

What else? I think there’s been some interesting ones recently around contraceptives and HIV, which I’m not sure if you’ve seen some of those so there’s a big debate going on right now about whether some of the modern contraceptives might actually increase the chances of HIV acquisition. The only thing I can say on that one is that the foundation was going to fund (I’m not sure if we did) but a definitive trial to try and get evidence either way on those risks and… So, yeah, I think the example I’ve given you will be a bit extreme, but do we try and understand, fund research through our grantees to try and better understand those harms, those unintended consequences? I’d love to think so. It’s just something that is not discussed openly.

Interviewer:	I see. That was a perfect answer. So, I do want to respect your time, F7 so final question of the interview. In a perfect world, what do you think would help you do your work better?

F7:	One thing?

Interviewer:	You can name multiple if you’d like.

F7:	[inaudible 00:05:22] Yeah, I don’t know. On this again, I don’t know whether you’re going to speak to other people in the foundation, but I’d encourage you to try to because I’m one out of 1500 people here and so I have a view on all of these things, but it’s just my view really.

I don’t know. I think better data for the type of stuff I do would be obviously very helpful. I think better capacity in countries to… well, at kind of every point really in terms of knowing which questions to ask and how to collect the data, analyze it and use it as well as stronger institutions in countries and that’s the area that I’m trying to support right now as well with this company to try and strengthen the capacity of institutions in developing countries to try to kind of evaluate evidence.

So, yeah, I’d probably say those top of my head… I’m sure if I had a bit more time, I’d come back with a bit more focus, a more thoughtful answer for you, but I’d say those: better data and then just stronger capacities. Again, we haven’t got a very explicit position on capacity building at the foundation. We clearly do it, but sometimes I think we try and bury within our other grants as well and the reason why it’s buried is because I think Our head boss has legitimately asked about the return on those investments. I think it’s kind of hard to measure it and it’s a long-term investment, but that’s clearly really, really needed if our work’s going to have the benefits and the impact that we expect it to have.

I guess the other kind… It’s a bit of a glib kind of answer maybe is stronger health systems as well so that’s something that we obviously have recognized a bit later than everybody else. That is an absolute kind of must if we’re going to achieve our goals as well. Does that answer your question? I can’t remember now whether...

Interviewer:	That’s perfect, yeah. That’s fine. Definitely those three things would definitely make this kind of work a lot of easier and I hope that we can get there one day.

F7:	Yeah, absolutely.

[end of second recording]

[total time: 00:36:41]
