# Supplementary material for: Supporting Better Evidence Generation and Use within Social Innovation in Health in Low- and Middle-Income Countries: A Qualitative Study
Source: PLoS One. 2017 Jan 26;12(1):e0170367. doi: 10.1371/journal.pone.0170367 (PMC5268497; doi:10.1371/journal.pone.0170367)
Supplement: S1 Dataset — (ZIP) [file pone.0170367.s002.zip › Data/Data - Interview transcripts/F8.doc]

Interviewer:	I want to respect your time and keep this fairly brief at about maybe 30 minutes, but I do have a couple questions. So, this is some research that we’re doing on better evidence generation in healthcare and we’re talking to practitioners, ministries of health, funders and then folks who do research and you’re kind of at the intersection of the latter two— being both a professor and also now working for your Foundation. To begin, do you think you could tell us just a little bit—for the record—what your job is at the your Foundation and the problem that you’re ultimately trying to solve?

F8:	I’m the Director of the vaccine delivery program at our Foundation. We have a goal as the vaccine delivery program for the decade of vaccines which includes preventing more than 11 million deaths before the end of the decade. That alliance is what this project trying to achieve, et cetera. So, our work is driven in support of that goal and as well trying to support polio eradication.

We try to do it by accelerating the introduction of new vaccines and by support to improve coverage, training systems to ensure high and equitable coverage of all those vaccines that we’ve invented, and we obviously believe that if we do both of those things we’ll not only help eradicate polio but we’ll drive a significant decrease in child mortality and there’s going to be an increase in equity.

Interviewer:	Great. When you’re talking about creating access to vaccines or delivery mechanisms, what types of evidence do you look to to sort of evaluate the various ways in which health systems can be strengthened and delivery of vaccines made more widely available, more efficient, those sorts of things?

F8:	Let’s take the systems and the delivery stuff to start with it. The truth of the matter is somebody coming from academia, I was a little surprised that there’s not as robust of a literature or a rigorous evidence base of what works for raising coverage. There are obviously examples of countries that have high-sustaining coverage that people look to. There are places with low-sustaining coverage that people look at and say, “There’s another one. That’s something that we don’t want to replicate.”

But if you compare it to something like if you’re an oncologist or in cardiovascular medicine or something like that, it’s not like there’s systematic reviews and meta-analyses of a series of trials. It’s a “in patients that look like this, you should expect a 5-8% increase of treatments that look like this.” There’s not that level and clearly a more robust evidence base in terms of implementation science and operational research I think would be very helpful.

Secondly, one of the observations of less, maybe inadequately leverage I think for making decisions right now and those are just systems modeling, so because experimentation in systems can be difficult to do... It’s hard to randomize. It’s difficult to intervene knowing that that’s the only intervention that you’re making in a system that has a lot of contextual changes around it, et cetera. It’s somewhat surprising in a way that we’re not using simulation models more to try and say, “All other things being equal, if we tweaked this part of the system, here’s what we think would happen to the other parts.” That would be fraught with a lot of generalizations and assumptions or whatever, but it could also help us organize those assumptions and be the basis for systematic empiric evaluation.

So, those are a couple of the areas where I think it’s been a little surprising to me coming in from a more academic background that those two pieces are not more robust in this area and that a lot of what we rely on with some degree of success… It’s not as though the whole field is desperately unsuccessful and has no idea what to do, but we tend to use routine monitF8g and the inference by experience and looser observational evaluation than some of the rigorous stuff that I was thinking might be available.

Interviewer:	I think that those sorts of gaps, I think a lot of folks who noticed some things and would agree with you… and have offered various theories on why that’s the case and how they would fix it. What do you think accounts for the gap in operational research and lack of systems modeling? What do you think put that back on the research agenda?

F8:	Well, let me say a couple things ‘cause remember I was a lifelong researcher before I got here, so researchers… And I was one, my dad’s one, I love them, et cetera, but research is chapter and verse about what’s wrong with what you’ve got today and as much as I love these people—these are my peeps—they’ll give you chapter & verse about what’s wrong with it and what they could do to fix the things that are wrong with the rigor, et cetera.

What’s often difficult for researchers though and this is often in the rub between the research policy stuff is they can’t be guilt of misplaced precision, that in fact the values or observations or precision that policymakers require for change is different than what researchers aim for or sometimes get agitated about. The trick is to see if you can find that middle.

So, as a recovering academic, I could take you through the burden of disease estimates for any country in the world around their pneumonia or their pneumococcal or their Hib estimates or whatever and I could take apart all the things that are inconsistent or less than perfectly accurate in that and I could prescribe for you a series of epidemiologic investigations that would help with the precision of that. I could do that. And I would be happy to take your money and do those things ‘cause I love doing that, right?

What I learned over time at the intersection between the epidemiology and the policy-building is that oftentimes the policymakers aren’t viewing it with the same level of precision that we are. They’re like, “Give me a number” and in their mind, there are vague numbers, really small numbers, and a bunch of numbers in between. And so, if our level of precision is only going to change the numbers from being one of those in-between numbers to one of those slightly more precise in-between numbers, it’s not actually going to change what the policymaker wants.

The point I’m trying to make here is I can tell you a whole bunch of things that I think that as a researcher I would like to see them put more precision there. I think the trick is to find the balancing point between improving the rigors so it should have reasonable confidence in what we’re estimating or projecting or whatever with... If you gotta tap policymakers’ minds, really what are the levels of precision that they need to swing them from Decision One versus Decision Two? And then, secondly, and this is… You’re a doctoral candidate, right?

Interviewer:	Yeah.

F8:	Yeah, okay, so I share this only with trepidation because I sound like an old, cynical man now instead of the... I used to love my PhD students at Hopkins ‘cause they brought about this sort... I loved the energy and “why can’t we?!” and I feel like this cynical man, but the truth is a lot of policy is just not driven by data as much as epidemiologists and policy people in Congress would like it to be. It’s just not. And so, I think the trick for us is really to find that sweet spot of whatever the decisions where evidence is really going to be helpful, how precise do they need to be, and then drive that through.

Just to give an example, in delivery, I would argue that you could probably go to a place like Cuba and take an analysis and say, “Listen, if you were to basically outsource all of your services to third-party logistics, organizations, and for-profit companies, we could potentially reduce the costs per life saved in your program X” and not change your outcomes at all. In that political environment, outsourcing to for-profit companies is not one of the values they have and no amount of analysis to show them that that’s a more cost-effective approach is going to change that.

So, I think one of the challenges as we go into this is to try to get the dialogue between the policymakers and the research academic quantitative community to try and find out what is that sweet spot, where are the places where they’re going to make a difference. This is a very long way of answering your question to say part of the reason why I think we haven’t had as much of that kind of research is because in some places the type of research that we’re asking for is not actually influential on decisions—even though they should be— are not as quantitatively made as we’d like, or the level of precision that the research and academic community is insisting upon is actually misplaced precision for the policymakers themselves.

… So that’s the critique, okay? Why don’t I stop there and then I’ll give you a couple of thoughts going forward.

Interviewer:	Yeah, sure. Don’t worry about offending my delicate sensibilities. I was a manager in Liberia for a remote healthcare program for several years actually before I came back here so I keenly sense the sort of... basically, what you’re … getting at that sweet spot is kind of why I came to do a PhD. I’m going to leave the academy when I’m done probably because I think why we’ve heard that echoed a lot...*a lot*.

Researchers do stuff with rigor that just actually solves a program or takes too long or it’s just mind-boggling expensive. We also know as researchers that the status quo probably could be improved and a lot of people are kind of angry at a... not a straw-man. I mean, something that’s real—RCTs do take a lot of time. Maybe they’re inappropriate method in some cases. They are definitely expensive in the way they’re typically run, but… Yeah. I would love to hear your thoughts on, “Okay, but how do we get a little closer to that sweet spot?”

F8:	I actually think the simulation models are one of the first places that we can go for the sweet spots because they do potentially solve a number of critiques, although they leave you open to some others completely. The thing about them is once you’ve built them, you can run them relatively quickly and inexpensively. You can go to Cuba and say, “Listen. If you changed your system in these three of four different ways, here’s what our model suggests would happen.” You could also then determine what are the really key drivers of the sensitivity analysis, like which are the ones that basically say, “Within a plausible range, the outcomes I expect to see are between A and B.”

And so rather than necessarily doing large randomized controlled trials, what you could potentially do would be to say, “Listen. We think if we change the whole country this way, we’ll have these big improvements. But that projection of benefits depends upon these three or four empiric things being right, so let’s try this in a couple of places and even in a non-randomized controlled way but in a carefully measured way, measure these three or four things, and then believe that if we measured those three of four things and they’re within the plausible range that we are going to see the rest of the systems changes that we hope for. Let’s try it and then monitor.”

I think that that is one way that that could work and we’ve had some very positive experiences with that. [REDACTED]

Many of the vaccine supply chain logistic systems in developing countries have four of five layers. They follow political hierarchies which are not necessarily the most efficient way to distribute medicines and vaccines, so what we demonstrated in the system modeling was you could basically improve your vaccine availability in country and reduce your cost per delivered dose if you went with a system that skipped a layer from five to four, four to three. (I can’t remember if they were five or four when we started.) And by re-organizing your distribution in transport to adapt to that fewer layer change.

Now in [REDACTED], they had a Minister of Health who looked at this and said, “This is great! This is what I want to do. Let’s go ahead and do it.” Not every country is going to have a Minister of Health who’s just going to look at a simulation model and say, “Yes, I’m ready to take on all the political changes that need to be made in order to implement that,” but I think those are the kind of examples of where simulation modeling can be helpful in some of those places.

I think of one of the other areas where we can improve is just by improving the precision and accuracy, the rigor of routine measurement of systems because that’s not as good as we’d like it to be, to be absolutely honest. The WHO/ UNICEF best estimates of inter-nation coverage are *best*, but they are still estimates and they are fraught with problems. Now some of the underlying root causes of these problems [REDACTED] we actually don’t know denominator numbers in many of the places where we want to work, so trying to measure something through changes and rates or proportions in which you don’t know the denominators, it’s very, very hard.

And so, researchers are like, “Oh yeah, I’ll just go and do a census and then I’ll know what the number is.” Well, as you know, politically, there are many reasons why entrenched power may not want for a census and accurate denominator number that go *way* beyond what your accuracy of your immunization coverage rate is. They’ll influence the distribution of power, resources, everything else in a country.

And so, that’s another of the sort of underlying constraint values we just have to deal with, but what I would like is if we could—and this is one of the areas that we’re trying to work on—if we could improve the accuracy and timeliness of routine data collected at a district level, one of the things you would do is you would empower district-level health workers to essentially tinker on their own. (Again, this will probably make sense to you if you’ve been in Liberia.)

The pointy end of the system are district-level managers: people who have between one and six facilities that they need to run generally, serving a population size that they can get their arms around. When they have timely, accurate data and a culture of data, what they do is they look at those data and say, “Well, why is immunization coverage flat in villages A and B? Someone go over there and figure out what’s going on.” And you go over and find out there’s been some challenge with the community or there’s been a problem with the stock-out or whatever it is and you solve that and then the next month you see coverage tick up and the district manager goes, “Okay, I solved that problem,” and then you look over at Villages G and H and you say, “How come we haven’t had any reports from them for two or three weeks?” and you investigate over there.

So, what we would like to do—and I think will actually help to improve performance wildly, if you will, and everywhere of systems— is just improve the district-level administrative data that people have available and use to support decision-making. And so in some ways, additional immobilization signs and things like that will be helpful as well, but if you really want to change everything everywhere, just try to improve district-level data and the ability of district-level managers to use that data and make intelligent changes in order to improve coverage is probably the killer [app? 00:21:56] in this whole system.

Interviewer:	Those were a bunch of suggestions about what types of what we should do and what types of data collection ought to be undertaken. Operationally or structurally, how do you think we make that shift such that that… So we know what should be done. How do we actually get there, in your opinion?

F8:	I’ve described a couple of different shifts. I think when we start with the last one… the answers that I don’t know yet but we’re working hard and investing in trying to do this. Now obviously things like DHIS 2 were an attempt to try and put some data in the hands of people [REDACTED] hat was a health information systems that’s been promulgated widely in places like that that was an attempt to try and get the countries to adopt the use of data a little bit more. It’s a decent start.

I think there’s even more robust ways to build the information systems, but what we’re trying is basically… In a couple of countries, what we’re trying to do is work with the country and try and understand from the country what is the thing, the architecture that they want, and then they’re willing to scale and support. To work with the users of the data to find out as parsimoniously as possible, what that minimum set of timely, accurate data, the kind of data breakdown do you need in order to be able to take this sort of sensible managerial decisions that you would like to be able to take to monitor and improve your coverage, and then to support the countries to be able to make those changes.

So, I think on the improving district-base data, in summary, it’s about engaging with countries to understand what data they want and need, will use for managerial level decision-making and then to support them to build those systems to achieve that. Part of the building of those systems is to do it in a way that’s scalable and sustainable by the countries so it’s better than bringing in the gold-plated system from the outside and sort of frugally innovate, sort of figure out, ‘Yes, we may want to bring some disruptive innovation in here, but make sure that even if they’re disruptive, they are still scalable and sustainable by the country.”

There’s a body of work that’s getting started on that and different programs are basically coming at it from different ways, but all arriving, I think, in similar destinations ‘cause malaria program need that, HIV programs need that, non-communicable diseases will need that, so that’s starting to come together around each of them understanding how a common health architecture supporting the same set of district managers will be helpful, so that’s one family of work. Once that’s up, it’s really a matter of empowering district-level managers to be managers, to use data to make informed decisions about how to correct their programs.

I think as we move up the ladder a little bit from there and ask ourselves more about the sort of research and evidence base that might be needed to convince policymakers to adopt new or different things in the systems area. I do think there’s obviously room for more implementation science… There are constraints on trying to get implementation science funded right now in the U.S. [REDACTED]

In the U.S., almost every biomedical area or domain of science has funding through the National Institute of Health, from dentistry to mental health and cardiovascular disease to infectious diseases, but there’s no National Institute of Implementation Science. So, implementation science or operations research is actually quite difficult to get funding in that structure because it’s not obviously in one place or another.

… I’m not calling for the creation of an institute, but I think trying to figure out how you fund stuff that’s academically rigorous and very valuable in spite of the structure that we have right now. It would be a worthwhile activity for people to think about in place of NIH.

And then, I think on the modeling piece, I think that really it’s just about starting to exercise that muscle a little bit more, to use those systems models more frequently, and I think there’s essentially a domain, a part of the enterprise that we could develop over time which is kind of an expertise in systems design where when countries are thinking about, “Hey! I really wanna consider the effectiveness and equity of my system, the efficiency of my system. Who do I call up and go to? Oh, it’s these systems design people and they have quantitative models and can give me an idea of ways in which I might be able to tweak my existing systems or create new ones that would be more likely to be as or more effective, as or more efficient, as or more equitable depending on what my challenges are to my system.”

But kind of creating that class of people, I think is a sort of evolutionary change that would be really good for our field, so those are just a couple thoughts.

Interviewer:	So you mentioned NIH funding and I think a couple other folks have mentioned that as being difficult, but we’ve also heard the claim that for researchers... basically that operational research, implementation research, delivery science—whatever you want to call it—just isn’t valued in the academy so for young, post-doc people who want to work with people who are doing some sort of health program somewhere and want to evaluate it, it’s just not a thing that happens based on how it’s actually structured. Would you agree with that? Do you think there’s a way that [inaudible 00:30:11] could change?

F8:	Well… Yes, I can say that that’s a legitimate critique and one that we’ve heard a lot. I can only speak from the one academic institution that I was a part of. What I can say there is that was certainly the conventional and prevailing wisdom when I arrived and continues today to be a concern for young academics who want to pursue this direction.

On the other hand, in fairness to [THAT INSTITUTION] which can be as or more academic than the most academic, they have communicated both to faculty and to their Appointments and Promotions committees that it values three things in faculty, research, teaching, and public health practice and they have now developed a way you can summarize your public health practice achievement and submit that as part of your application to the A&P committee in ways that the A&P committee can then evaluate that and incorporate that in their evaluation of you for a promotion.

Before I left [THE INSTITUTION], I was promoted to full professor and I put this together and the feedback I got when promoted was what they called “the public health practice portfolio” that I had included was a significant part of the basis for the agreement to promote me. So, that’s still not sort of standard and part of the problem is that I think young people— they hear that, but they don’t really see it and they don’t believe it— so they sit there and think, “Ugh! What am I going to do with my time and how I going to do this, et cetera? Well, they’re telling me that if I do public health practice, I can put together a public health practice portfolio and get promoted, but there’s only like two guys here who have ever done that and the rest of them have done it just through hardcore first and last author papers so that’s what I’m going to gamble on.”

I think it’s a change process that needs to happen, but in fairness to the academy, at least at [THE INSTITUTION], I think it’s begun to happen and reinforcing that is one of the ways that you can start to stimulate that change.

Interviewer:	No, that’s great to hear. I hadn’t heard about that. Well, listen. Like I said, I want to respect your time. We’ve gone just over half an hour. This was great. Thank you so much for sharing your thoughts. You’re one of few folks who kind of have your feet in a couple different worlds and I think that’s exactly where we want to be taking this, so it was great to talk to you.

F8:	Great. My pleasure.

Interviewer:	Alright. Have a good day.

F8:	Thanks. Take care.

[end of recording: 00:33:56]
