# Supplementary material for: Supporting Better Evidence Generation and Use within Social Innovation in Health in Low- and Middle-Income Countries: A Qualitative Study
Source: PLoS One. 2017 Jan 26;12(1):e0170367. doi: 10.1371/journal.pone.0170367 (PMC5268497; doi:10.1371/journal.pone.0170367)
Supplement: S1 Dataset — (ZIP) [file pone.0170367.s002.zip › Data/Data - Interview transcripts/F9.doc]

Interviewer:	So just to begin, I’ve obviously looked into your foundation but for the purposes of the record, do you think you could just start by telling us what you do and what the goal of your foundation is?

F9:	Yeah, sure. Our foundation was forged by a large organisation and we’re a public-private partnership with various members and others from the private sector.

The goal that we have is to use mobile technologies to help support and improve the performance of frontline health workers and mainly those who are working in rural settings in sub-Saharan Africa and South Asia. We do this in three ways. We have a strong country program where we’re supporting organizations to scale up existing health interventions, but we also help organizations design and start implement programs that we can then either support with funding or with strategic advice. And then, I think our main area and where we have particular distinctions to our content platform, ORE?, where we’ve created an online library of open-source content and we’re really focusing on six key health domains at the moment that are around women and children’s health.

What we’re doing is we’re beginning to collect video, audio, and other resources that can be used on mobiles and can be adapted and translated and used anywhere in the world actually. We only launched in June but we’ve already got about 300 resources in the library and up to 16 languages and so we’re just reaching out to content developers to really grow that over the coming year and get the content re-used in programs … in the countries we’re working in.

And then, the third area that we do is around research and global learning and really working through our partners in universities and other institutions to understand how mobile technology is being used and understand better the barriers to scaling health programs and then to share that information through our website, through conferences, webinars and so on. And then, all of that is underpinned through our communications and advocacy strategy, so I guess in a nutshell that’s what we’re doing.

Interviewer:	That was a very clear exposition. Thank you so much.

F9:	Okay. You’re welcome.

Interviewer:	Obviously, [I recalled? 00:02:41] the chunk about research so I guess to focus on two parts of that: when our foundation is choosing or attempting to find partners with which to partner and support and help to scale up existing health interventions, what do you look for in terms of being able to differentiate strong or promising programs?

F9:	Yeah, that’s a really interesting question because it’s not always straightforward. We have a list of criteria which includes things like government buy-in, however that might be defined from simply the government is fine with the program going ahead right to this is a government-owned and leased program they have asked for support with	and there’s a whole kind of range in between those two things.

So, the track record that the program implementers have in working with government is something that we look at very closely. We also look at whether or not there are similar efforts going on elsewhere and whether a program might be better to become part of a consortion rather than to do something on their own. We look at the content that they’re using and however that matters to frontline health workers and whether or not the technology they’re using is something that’s introducing a new dimension to a program and therefore what’s the sustainability model and what’s the business model for keeping going after the grants are finished?

Usually the argument’s stronger where existing technology is being used and existing infrastructure is being leveraged for a program so then you get a sense that there is the potential for it to at least sustain beyond the initial grant. And we just look at the track record as well of the organization. I have more confidence in working [REDACTED] than somebody who’s a very new entrepreneurial start-up organization where they may need additional investment and support just to manage themselves as well as to manage the program, so there’s a whole range of things that...

Sometimes it’s just a gut feeling. It might be something very new. It might be untested, but you kind of have a sense that there’s real excitement. There’s real engagement with the community. They’ve done their homework and so we also could invest in those kind of partnerships as well.

Interviewer:	Okay, so there’s obviously differentiation between a project that’s on the verge of beginning or just beginning and then one that’s been around for a while and it maybe one that’s being conducted by a trusted partner. Do you, when evaluating or looking to partner with these organizations, look at outcome indicators, and if so, what types? How do you assess the mediating power of mobile phones versus just the existence of the frontline health worker in and of itself?

F9:	Yeah. I think it’s been one of the issues in the m-health space as to why things haven’t gone to scale because people aren’t really sure about what it is they’re measuring and I think there’s always an over-expectation from some of the big donors that you will be able to demonstrate how many lives have been saved, for example, which it’s *very* difficult to do in a three-year program when there are so many variables to take into account.

So, we don’t really look so much as what does the m-health bits of the program achieved. We look more as to what are the health outcomes that you’re hoping realistically to achieve with your program and does using a phone help make that more possible, make it happen with better quality. Are you likely to achieve your goals more quickly and efficiently because you’ve used a phone? And then, there is the aspect. Did the phone actually work? Did the SMS message go out? Did the video work or was the broadband connectivity too poor? So there are the functions of the phone that you can test in a very simple way.

And then, we would look. If someone was doing data collection, you can use the analytics that are built into the functionality to just look at how often was something used, how often was it viewed, by how many people, so there’s synthetic, basic stuff you can do. But, our foundation is a very small team of 2 ½ people, about to be 6 which I’m very excited about, so really what we look for whether we either invest time or we put seed funding into programs is what is the evaluation that our partner is proposing to use and how well does that align with the methodologies you’d expect a program to be using to measure its outcomes.

But, we tend not to go for the ‘lives saved’ metric because I think that’s just too difficult to really show in connection with some of these particularly smaller programs. Sorry, that’s a bit ramping off.

Interviewer:	No, no. I think it is complex, so I do appreciate you taking the time. So, point taken about existing projects. You also mentioned that your foundation conducts research or at least sort of consults on research that’s taking place in the academic space and partners of the academic space, so what are the main... You alluded to ‘we have real struggles with how do we actually design [or bate? 00:08:40] what’s going on in a particular program and what sort of inputs, what sort of supports, are making the difference for these frontline health workers. What are the big challenges that you face in doing this research and what kinds of questions are you presently asking?

F9:	We’re looking at three or four main strands of activity. One is around the learning and information needs of community health workers and the definition of course is incredibly broad so the research on that is also very broad, but getting a sense of what are their needs and how are they currently being met with existing training and refresher training so we did some research on that. (I think it was either last year or the year before.)

Anyway, we’ve published a report. [REDACTED]

And then, we did a series of consultations. We did 6 countries in 6 months, bringing together private sector, NGOs, government ministers, health practitioners, academics to look at learning and information needs of CHWs, and from that did a report on what’s needed, where are the gaps, and can those be addressed. So, that was a big cluster of work around community health.

We then did a literature review of how frontline health workers—also broadly defined— are using mobile technology to provide better quality services and that was published in a peer-review journal a few months ago. We’re partway through now doing a literature review and some on-the-ground research in [REACTED] to look at nurse leadership and how community health workers are integrated into the primary healthcare system in [REACTED], so findings from that research will be disseminated early next year and there will be some kind of event in [Luth-falka? 00:10:57] to talk about the findings.

But for us, what we do, is we take that research and then we use that to inform thinking about how do we invest wisely and not just in pairing, but when I’m working with [REDACTED] helping them to shape their thinking about, “Given the findings, how do we then start to invest in these programs?”

And the barriers are pretty much the same whether you look at health or education or agriculture extension workers. It’s about government buy-in; it’s about relevant content. When you look at the m-piece, the mobile piece, it’s usually about the relegating framework, it’s about connectivity, it’s about the cost. There are always arguments about, “Do you provide health workers with phones or do you use the phones they already have?”

So, all of these kinds of things are swirling around in the conference and workshop and webinar debates that go on about how to really go to scale and I think until we really crack the data access and the costs of accessing and downloading data, it’s very difficult for programs to go to scale.

Interviewer:	And this work, is it mostly descriptive or qualitative? Has anyone ever involved counterfactuals, like *these* groups or this group of frontline health workers receiving clinical supervision from a nurse versus this group didn’t enjoy this group? Very basically like had mobile phones versus group that reported using paper forms, things like that?

F9:	Yeah. Most of the big NGOs carry out that sort of research and in fact, I met with somebody from [REDACTED] and she was talking about some RCTs that they’ve been doing in West Africa, looking at training that they’re giving private sector health workers including pharmacists and retail shops that sell various commodities, and so they’ve been doing some training with them and then comparing the effectiveness, knowledge-retention, and behavior change compared with those who haven’t had the training.

[REDACTED] had an independent evaluation done of the program they run in [REDACTED]. The impact on maternal and child mortality was incredible, just within three years of the program being implemented and that report is available on their website.

Interviewer:	Yeah. There’s been a couple of systematic reviews that came out a year or two ago on global health interventions. A lot of them say actually “more research is needed” or “we think that they improved quality of care,” but like you said there’s actually quite a few questions vis-à-vis implementation.

One thing that we’re asking everyone whether they’re implementers or funders or where they are in terms of health innovation is whether given the history of social programs that despite being designed with the best intentions have sometimes had unintended consequences or caused harm. In what ways does your foundation think about harm or measure harm within partner sites or more generally just vis-à-vis particular types of implementation of mobile health technologies? Or do you think about that at all?

F9:	No, and I think that’s a really excellent question because those unintended consequences are often dramatic. I think this is where it’s difficult to do RCTs in health situations because if you start to see there’s a beneficial impact to the community or to individuals in the community then how can you *not* provide service to other communities that aren’t currently included in the program?

So, one of the things that we look for when we’re investing in a program is what is our partner saying about the impact for gender? What might be the impact if you’re giving women a phone when that might be seen as a commodity that has a cost attached to it? If you’re sending messages to women, are they actually getting to women because quite often in places where partners are working, it’s the men in the family or the community that has the phone and not the woman?

I think there was an interesting program in [REDACTED] that [REDACTED] was doing where they wanted to give the community health workers phones but all the health workers were women and the women weren’t allowed to have phones so they then had to look at how do they include men in the program who would accompany the health workers on their visits, their household visits, so usually it’s something that our partners think about very carefully before they do programs and we would look for that when they present their proposals to us.

Interviewer:	Okay. You mentioned that your foundation both provides grants but then also being as it is a part of a larger organisation... or works very closely with the larger organisation, you also kind of try to make things a little bit clearer for them as well in their other grant-giving undertakings. … Once a group has been selected for support, how are those grants typically managed? What type of reporting is required? What’s the goal?

F9:	It varies considerably depending on… and the sort of grants we give are really small and typically, they’re to help an organization unlock other funding. For example, [Diff-ent? 00:17:36] may say to an organization, “We’ll fund you for $500,000 but you have to find some match funding” and so we’ll contribute maybe something between $10,000 and $25,000 just to help make a match funding contribution. So our reporting, we try in that situation to mirror what the larger donor requires so we’re not putting undue pressure on what could be already an over-pressured projectee.

In terms of funding that we get, it can vary from needing to report in to a donor every month. Sometimes they might only want us to report in at the end of the year and give them the sort of analytics that they ranked that they need. Some donors want weekly phone calls and monthly formal reports, so it really, really depends who we’re supporting and who’s supporting us and [REDACTED] tend to have a quite formal reporting requirement and they’re usually on a quarterly basis and then a more detailed report on an annual basis.

Interviewer:	One of the obligations levied or characterizations of policymakers is that they often privilege context over outcome data, so for example if this program was very successful in [REDACTED] and [REDACTED] tries to then bring it to [REDACTED] and then [REDACTED] will say, “Well, that’s great… once we do a pilot *in* [REDACTED].”

Have you seen that? Does that mirror your experience of context being very important or do you think that some of these tools and approaches are a little more generalizable? Like, for example, you guys have ORB. That might suggest that we can take tools in use them in different contexts. How do you approach that tension?

F9:	I’ve certainly seen that—“Well, since it wasn’t designed in our country, then it’s not going to work here.” I think that’s really beginning to change and I think that’s partly influenced by global financing where there is increasing expectation from donors, big donors like the [REDACTED], that resources will be re-used and adapted.

However, the funding landscape still tends to favor everything being done from new. There’s not so much focus on rebuilding on what already exist and there’s a lot of rhetoric about collaboration and about re-use of content and models , but then so much of the funding is asking for “innovation” and the new and the shiny, so I think there’s a real tension between people talking about using open-source code and open-source content. Donors actually funding things like new and innovative and don’t know what’s already there.

What’s really beginning to change that is what happened with the response to Ebola. I’ve been really impressed by the way the [REDACTED] have been working in West Africa for the last 15 months now really, really pressing the global health community to collaborate and to re-use and create interoperability between systems and in fact just before our conversation now, I was on a weekly phone call that we have with [REDACTED] and technology designers and implementing organizations in West Africa and every week it’s like, “What are you doing? What are the workshops you’re running?”

People are expected to share information. They’re expected to share data with the U[REDACTED] There’s a backed fund in the next couple of months, a real push towards interoperability between health information systems, not just within countries but between countries as well and they will be funding collaborative efforts. They will not be funding one organization as full prime with supports.

So, there’s a real push towards re-use of data and creating platforms with KPIs that will be able to speak to each other. And what I’m finding now, even though we only launched off in June, that there is a real appetite for taking content that exists and then contextualizing it so we’ll move away from the, “Well, it was designed in Ethiopia so we can’t use it in Senegal” to “Okay. Here’s some great material on hand-washing, breastfeeding, and antenatal care, whatever. Now let’s look at how we take that, adapt it, run data and use it in Senegal and create a learning pathway around that content that’s very relevant for our context.” And so, the contextualization then becomes at a program level rather than at a content level. Does that make sense?

Interviewer:	Yeah, no, absolutely.

F9:	So I think it’s slowly moving that way, but until the donors really take responsibility for requiring their grantees to use existing content and to share new content that they create, I think things will move forward quite slowly. And one of the roles our foundation has, we’re a very sort of neutral organization, if you’d like, so it makes it much easier for us to talk to the different big donors and the big organizations and encourage them to think in this way, so I’m really hopeful that we’ll have some influence in the thinking going forward around open-source content and open-source code.

Interviewer:	Yeah, so that’s an interesting tension— speaking to practitioners who say, “Well, our push to research and development of tools and things like you say really is very much influenced by what donors seem to want” and then oftentimes we have donors that we then hear say, “Well, we actually let the folks we fund guide what KPIs we should be using and we’re actually quite suspicious of [switching to? 00:24:10] philanthropy, et cetera, et cetera,” and yet here again you’re saying, “No. Donors do, for better of for worse in some ways, set agendas vis-à-vis approaches to implementation, whether we’re doing something new or whether we’re using something that exist.”

… You’ve mentioned some efforts on the part of your foundation in terms of talking to donors, but on a structural level, how do you think that that changes? How do you think that donors confront their agenda-setting role in a way that’s proactive but in a way that’s not sort of suffocating?

F9:	Okay, interesting. I think the unhelpful answer is “it depends.” Some of the smaller foundations we work with to encourage them to fund programs that we’re interested in take quite a light touch and they’ll have certain metrics that they look for and they’ll want to be able to talk to their trustees about impact from the funding that they’ve invested, but they will be more influenced by the program and the program’s robust way of being able to indicate the impact that the money will deliver whereas if you talk to larger funders, they’ll have a very clear strategy. They know what they want to invest in. Their RFPs are very prescriptive and evaluations are structured to deliver good results, frankly, ‘cause if you did have good results, then you’ll possible get more money.

I think it depends on the size of the donor and it depends on whether the donor has built a relationship with the program. When you look at various organisations, these are really thoughtful programs, they have a good relationship with the donor, and I think they work together to understand how they’re going to measure impact for investments. But, to some extent, the huge donors don’t have time for that relationship building and they’ve got to report to the U.K. government or Congress about impact and so they approach it, I think, in a very different way.

Interviewer:	I’m just running through a whole series of allegations with you. No, but just to get your thoughts ‘cause I think often what happens is everyone points the finger. The practitioners say, “Well, it’s because the funders don’t do X,” and the funders say, “Well, we can’t do X because there are no practitioners doing Y,” and policymakers say, “Well…”

So, it all goes around in a circle. I think part of the idea behind this project is we actually sort of take some of those claims and have a comedic conversation, so to speak. Another thing that I think we hear often and we see it in the literature, we hear often from practitioners, that there is that the huge amount of intervention that happens in global health is quote “scaled in an evidence-free zone,” so organizations don’t measure their impact because funders don’t ask them and funders don’t ask them because they don’t really understand or it’s not in their best interest because who wants to know that they’re not…?

Obviously, none of these groups are homogenous and there’s a lot of variation in between, but do you agree with that assertion or partially agree? And if not, why so? What do you think would help us do better in terms of identifying impact in a real way?

F9:	I think there is increasing expectation that proposals are built on evidence of what works and if something proposes a new idea, then there are at least some kind of proxy indicators for potential impacts and there’s logic models and theories of change and et cetera, et cetera. I find most of those completely tedious, to be honest, because they’re just unrealistic about how the impact is related to the investment.

Going back to what I was saying earlier about it, they can say, “Yes, we saved the lives of 5,000 women.” I just think that’s really hard to show that one program has done that on its own. There are usually multiple other programs going on in parallel or even overlapping with projects, but I think there is beginning—certainly evidence bases beginning to develop, to show that mobile offers an opportunity to do things more effectively, more efficiently, faster, reach more people, and I think where proposals are based on that kind of evidence, they’re more likely to be the ones that are invested in.

I think it would be unusual for a program to get much money that wasn’t able to demonstrate some evidence of why they have the methodology that they were proposing. I think where it’s difficult to show evidence is behavior change. So, there are lots of S&S programs that are very effective in showing how many women they reached or how many families they reached. What’s harder for those programs to show is what behavior change is the result of those messages being received. I think the evidence base is growing. But as I said, I increasingly don’t expect to see proposals being framed within the evidence of what works.

Interviewer:	… I want to respect your time. We’re just about 30 now so I’ll just ask one final question of you. Evidence! We haven’t talked about evidence as kind of a monolithic concept, right? There’s evidence and there’s evidence, but of course there are different types of evidence. There’s certain questions that can be answered using qualitative evidence, certain questions that can be answered using numbers—numbers over time, numbers at one point in time—and yet, oftentimes...

You just mentioned we have programs that say, “Well, we reached X many people” as opposed to some sort of implementation outcome as opposed to “this many people (N) changed their behavior.” In your experience, is there among funders a nuanced idea or a nuanced understanding of what’s an outcome? What’s an output? How is this being measured? Is that a good way of measuring it or just “if you think so, then that sounds good to me”?

F9:	Yeah, there is a bit of that. I think there is a fairly sophisticated understanding amongst the donors that I work with about the difference between outputs, outcomes, impact and so on. Quite often, grants don’t have sufficient money to do really good, quality, qualitative research and I think the easier way to do the quantitative and then just present numbers and of course you could present numbers in a way that looks as if you had impact. I think there’s a growing understanding that…

Let’s just take this specific example. Just because women went to more antenatal care visits doesn’t mean that they got the right information when they went there or they had a quality service when they got there. So, I think there’s an understanding that there needs to be more qualitative research done to understand if programs are really making a difference and behavior really is changing.

I heard yesterday for example about a program that demonstrated the health workers who had training in this particular health domain, their knowledge increased dramatically, *but* when there was mystery shoppers who went to see how they were applying their knowledge in practice, nothing had changed. So unless time is allowed and funding allows for that qualitative research, then it’s cheaper and faster to do the quantitative stuff.

Interviewer:	[crosstalk 00:32:49] Yeah, no one likes to look at how things are actually implemented and that’s key, but more difficult definitely. Okay. Well, I thank you so much. Like I said, these are… They’re just…

F9:	I enjoyed your questions. I think the work you’re doing is very important. I look forward to reading the report that you publish once you bring all your findings together and do your analysis, so thank you for inviting me to talk with you. I’ve enjoyed it.

Interviewer:	Thank you for participating.

[end of recording: 00:33:23]
