# Supplementary material for: Supporting Better Evidence Generation and Use within Social Innovation in Health in Low- and Middle-Income Countries: A Qualitative Study
Source: PLoS One. 2017 Jan 26;12(1):e0170367. doi: 10.1371/journal.pone.0170367 (PMC5268497; doi:10.1371/journal.pone.0170367)
Supplement: S1 Dataset — (ZIP) [file pone.0170367.s002.zip › Data/Data - Interview transcripts/M1.doc]

M1:	[REDACTED] As such, we are a small unit that was created to help the [REDACTED] become sustainable or be more sustainable way back in 2008 – 2012 and our funding comes from two organizations so that we can try and drive that across the sector. Does that work as an introduction?

Interviewer:	Yeah, absolutely. How you define sustainability?

M1:	I would define it in a similar way to the Sustainable Development Commission, that it’s within environmental limits that’s [inaudible 00:00:47] which reminds me of five pillars… and things around the Commission too… a healthy, fair, and just society where with the right kind of economy, within environmental limits, threshold participation in the right way, and that uses evidence and social research — you know those five.

Actually, it was the previous government in the [REDACTED]who defined it as such and we still use that a lot… If I can just remember exactly what the words are… Sorry. I’ll come back to that ‘cause I normally have it and I should have it on my tip of my tongue but I don’t have it at the moment.

Interviewer:	Oh, no worries. Yeah, we can come back to that. What would you say the biggest challenge facing that department/ your organization at the moment is?

M1:	Well, so far, there are a lot of challenges when you take quite a broad definition to sustainable development and then there’s a lot of opportunities. I think one of our biggest challenges is that we’re dealing with a sector that is *huge*. Just our ministry has [REDACTED]million employees. We cover a population of [REDACTED] million. There’s probably another [REDACTED] million employees within social care and then there’s all the public health angle, so trying to tackle it in a comprehensive way is quite something. I think the advantages are obviously about this is a health issue as far as we’re concerned so that helps because we should be related to core business and how we take that forward. The difficulty with core business and healthcare is that we often get waylaid by short-termism and thinking that the emergency is everything and so when you’re trying to deal with longer-term issues, it’s harder…

So that’s a dual-side coin — the two sides of the coin — that we’ve got a good entry point because it’s about health, but on the other hand, it’s quite difficult to discuss longer health issues when people are fighting in the axis of the emergency department for today’s light, if that makes any sense.

Interviewer:	Yeah, it does make sense… Two questions. First of all, how do you learn about potential solutions to health problems? And then…

M1:	Sorry. I’m not hearing you well. Would you be happy to repeat that?

Interviewer:	Yeah… You talked a little bit about the interplay between your people who are actually delivering healthcare and you. Before you get to that point, how do you learn about potential solutions to various sustainability problems and decide that this is maybe something the your ministry should take up?

M1:	Well, I guess one of the things is we’re a really small unit with [REDACTED] people. We try to make sure that we scan the horizons of what other people are doing to get better ideas from elsewhere. We learn a lot from within our own sector from what people are doing so we do it by running awards. For instance, we get a lot of information about what people are doing and good case studies and we scan for what other people are doing…

Some of the things that we’ve done have because we’ve seen them either done in a similar way or we’ve stretched it across from a different sector. For instance, we developed a route map for sustainable health. We picked that up as a consequence of seeing the World Business Council for Sustainable Development’s route map for a sustainable world and noticed that health, what did we really figure in it, and felt that health really needed its space so we developed that.

Interviewer:	What types of metrics do you look at? So maybe you hear about the organization’s implementation tool or best practice from other people in the field, but when deciding “this is right for us in the [REDACTED],” what type of process do you use or types of outcomes do you look at to evaluate?

M1:	Well, at the moment, we’ve generally found that… we feel as though we’re ahead of the curve on things that are going on in relation to the health sector because we’ve tried to take a view where this is about everything that we can influence. It’s not just about energy, waste, and water, for instance, and so we’re trying to push at those boundaries quite a lot ourselves and we want to look at health outcomes alongside some of the social value elements as well as the environmental impacts and how we bring in those externalities.

So when we are looking at something— a good idea from a different sector, we might be looking at it from a perspective of, “Well, they’ve made it.” We’ll look at the change process they’ve been through, about how they’ve reached out, whether their messaging has spoken to people, whether it’s something that addresses one of our barriers or one of our opportunities, but seldom have we ever seen it described in ways that would be entirely practicable for ourselves either in terms of healthcare and outcomes to health. I can give you my definition if you would like again. I’ve found it.

Interviewer:	Great! Perfect.

M1:	For me, sustainable development is defined by you need to live within environmental limits, ensuring a strong and just society, achieving a sustainable economy, promoting good governance and using sound science responsibly. And that’s taken from *Sustainability Principles*: *Securing Our Future* described in 2005.

Interviewer:	So given the large mandate of your unit, what are some of the ways in which you evaluate your progress towards that definition?

M1:	We’ve got some things. The first thing that we did was to establish a carbon footprint of the our ministry, including [Scoop 1-2-3? 00:08:27] and we’ve been tackling that every 2 years since 2004 so we’ve got some carbon footprints of our ministry going forward all the time, so that’s one measure and it’s probably the easiest one.

… We’ve done things. We developed a route map of sustainable health, as I mentioned, and we use that as a framework to measure our progress so that we’re trying to cover all the different aspects so some of that’s been about collecting what we think organizations are doing within our systems, so we map, for instance, all of the data that’s available to us in relation to estates and facilities so that might be all the waste water and other elements. We’ll also have energy units.

We’ll also look at [inaudible 00:19:19] — when a big blur of organizations have got a sustainable development and management plan and approved at board level in their organization ‘cause I guess that’s an indicator of process. We look at organizations’ annual reports and how well they reflect this in annual report which is publicly available, and then we do a collection of things that we think are happening at national level that might be pushing these things forward. For instance, there’s our “Sustainability Day” run now for 4 years where we would be looking at the uptake every year on that and how that’s progressing.

We run some public opinion surveys every 2 years, so we utilize that as some evidence, and at the moment we’re collecting people’s views on how they think we’re doing against this route map, so we try and do a mix of quantitative and qualitative data on that.

Interviewer:	Do you ever use counterfactuals in your work?

M1:	Sorry. I didn’t hear that very well.

Interviewer:	I said do you ever use counterfactuals in your work when assessing different, potential ways to reach the same goal?

M1:	Counterfactual? Is that what you said?

Interviewer:	Yeah.

M1:	I don’t know what you mean by counterfactual.

Interviewer:	For example, let’s say you want to lower the carbon impact of the your ministry and different policies have been used in different places. Do you ever say, “Oh, we’re going to look and have one set of hospitals do this, one set of hospital do that,” and compare at the end? Or do you look at that type of evidence where it exists elsewhere?

M1:	No, but we…

Interviewer:	Just basically the use of comparisons to sort of see if things were going to improve anyway or whether it’s the direct result of a new policy or a new change.

M1:	No, so we haven’t been systematic like that. We do have a good corporate citizenship tool where people can assess how they’re doing on different elements and we have gotten access to that data so we can look at that. We do know that some hospitals or people do a lot better, for instance, on food and others a lot better on waste and others a lot better on other things and we’re still at the stage of trying to promote it to everybody and take those things forward.

We have done things like asking chief executives which of the bits do they find easiest to tackle and the bits that they find harder to tackle… because we can’t tell anybody to do this or to do that. It would be very difficult to say to those 10 hospitals do it this way and those 10 hospitals do it that way, because in a way, each hospital or each organization we work will chose to do it the way that makes sense to them.

Interviewer:	Okay. And how did you get connected to this health organisation?

M1:	Well, I think they might have found out about what we were doing at some point and got in touch with us. Yeah.

Interviewer:	Okay.

M1:	I think that’s how it happened. Yeah.

Interviewer:	Great.

M1:	And they process a slightly different point of entry to us in that they’ve really gone further in describing the harms in relation to climate change or toxicity and other environmental impacts… Our point of entry initially was very much carbon reduction in relation to the [REDACTED] Act and then we broadened it to sustainable development and now we very much advocate that you need to enhance the positives at the same time as you reduce the negatives so we’ve got our own model for sustainable healthcare that we have put in a strategy that we launched in 2014, which really is recognizing that you need to work on reducing the burden of disease, reducing inequalities, and reducing environmental impact while you’re working on enhancing the natural environment, enhancing and building on social assets and enhancing independence and well-being… and that’s how you end up with more sustainable, resilient and healthy people and places.

Interviewer:	You mentioned not being able to tell hospitals what they should or shouldn’t be doing.

M1:	Yeah.

Interviewer:	So how do you attempt to… When you’ve keyed onto something that you think, “Hey, this will make a difference. We should be pursuing this path,” how do you exert…

M1:	Encourage people to do it?

Interviewer:	Yeah! Influence, like what do you find to be the most helpful in doing that or what’s your general approach?

M1:	We [inaudible 00:14:31] so we recognize people through awards and that’s one good way of achieving recognition. We’ll highlight case studies on our website and we disseminate it through to people to say, “Look, this is a good idea. This is what people have achieved.” We’ve done things like include a number of the evidence of interventions in a [REDACTED], for instance highlighting work. These interventions can save this amount of carbon and save this amount of money based on the amalgamation of evidence.

Or, if it’s a concept or an idea, for instance a moment that we want to test out a little bit further, we might hold a roundtable where we’ll try and disseminate it further with a group of people or experts in the field to see if we can reflect a little bit further and see what the next steps might be. Or sometimes we work with networks of hospitals to discuss a particular topic that they’re interested in or that we want to push, we might hold a roundtable around that to try and take it forward.

But really, the way that we try and encourage people to do more so there’s the element, the shtick that we’ve got is not by telling people to do this because we’ve got our ministry contract that states that people should be doing these things and how much they do and how much that gets discussed in a contractual process we’re not always sure of, but it gives them a basis for doing it and then we try to encourage good practice by highlighting it and understanding why it is that it particularly works or what the lessons are behind it.

Interviewer:	Okay.

M1:	Does that make any sense?

Interviewer:	Yeah, that does sense. Do you think you could give us maybe an example just to illustrate everything you’ve been speaking about, of some innovation that’s actually been scaled up by a number of hospitals your unit has identified and then lobbied for and helped support as it got enacted?

M1:	Yeah. Let me think what would be a good example… In the energy-efficiency realm, there’s been quite a lot where we’ve identified practice, for instance, with uptake loss that are doing something around behavior change but they’ve succeeded it really well and how that might scale up to the our ministry and try to disseminate that much more widely and that’s been taken up in a lot more hospitals. I think that’s not just to do with us, but it’s been to do with the fact that the hospital that did it were very good at advertising it as well and I think the [inaudible 00:17:35]. We’ve been doing things, for instance, around identifying that inhalers are a big part of the carbon footprint here in the [REDACTED]and because we have discussions with the pharmaceutical industry, that’s led to them recycling and trying to recycle the inhaler devices, to be able to collect them and recycle them, and also change their pricing structure to make sure it was easier for us to change it from a inhaling propellant to a pulverized form.

You could include this as being a node around identifying best practice, around what people are doing in terms of smaller, core sustainable food that’s also healthier… by highlighting that too. We bring people into something. We might do some leadership development courses. We bring in some good case studies and highlight how people have gone about doing that. Then, we have networks across the regions where people share their good practice. I think one of the things that we do encourage quite a lot is that good process within organizations by which they identify what they need to be doing as an organization to take this forward and how they might present a plan to the board and have a process for reviewing that on a yearly basis and putting that into the annual report, so this is the concept of the fact they can evaluate where they’re at, ascertain the gaps, and develop a plan that’s got full board approval, and can provide some proper accountability towards it and the way they’re dealing with it is quite important.

Interviewer:	Okay… When you’re recommending things like behavior change or doing different policies that should be enacted, oftentimes those things have unexpected effects like we encourage physicians to wash their more and then waiting times increase, something that’s not a sheer relationship that exists but…?

M1:	That you want to do no harm?

Interviewer:	Yeah.

M1:	I think it’s too early to say. I hope not, but one of the things we’re still trying to push is we’re not necessarily very good at measuring during the overall elements. For instance, at the moment, we’re doing quite a lot of changes around the model way we deliver services and if you just did a measurement around travel, you could think that there’s an unintended consequence, that by having less hospital admissions, you’ve got more travel because people are travelling around the place and delivering care at home and we’re trying to make sure that we’re developing methodologies and make sure that we take into account the carbon footprint of all that, the fact that you would be using less energy and products in a hospital versus intensity and you might be abusing these products or using them in a different way than actually they are.

It’s quite a complex picture, so with that what we recognize is that sometimes when we do think that there’s unintended consequences, we’re actually not very good at doing the pluses and minuses, the balance overall. So in relation to care pathways, for instance, we’re trying to develop some guidance to help people be able to determine that a lot more effectively. For instance, what is the environmental impact of an A&E [inaudible 00:21:27] or an operating procedure or a day in hospital so you could start comparing things more effectively than the way we’ve seen it compared to some other...

‘Cause we’ve heard people say, “Ugh! You should do, move these services. You’ll have an increased travel impact and it might still overall be better because you might use less medication and you use a whole lot less of improved products that you’re buying that you might use through the hospital.” I think that we recognize that there isn’t yet the adequate methodologies to do that well.

Interviewer:	What do you think would help you do that…

M1:	Better?

Interviewer:	Better, yeah. What types of resources?

M1:	… For instance, we’re going to be releasing the methodology in October and we’re hoping that many people can use that more. That will bring us more data and more… You have to understand and evaluate these impacts a lot better and we’re also having some discussions about how once you do know that you can actually include that in decision-making or counterbalance it with financial decisions, for instance, and I think for me having the clarity of thinking so that we know that we’re utilizing recognized processes around the economics and finances and are planning similar approaches to environmental, social sustainability so that you can actually make the right decisions... numbers that matter and that people recognize and do it in the right way is really key and I don’t think we’re really there yet. Anywhere. I haven’t seen it anywhere.

For instance, one of the things that we’ve been really quite keen on is saying that for carbon footprints that we’ve done for the our ministry is across all areas. It’s an absolute. It’s not a pro-activity or propelled or a pro-patient. It’s an absolute whereas maybe some companies might say, “Well, they’re reducing the carbon footprint of their products by 50%, but nobody’s counting the fact that they may be selling twice as many.”

So, I think there’s a lot of stuff out there that’s not real or balanced out in the right way so I don’t know if any sectors got that right yet. I think we’d like to find a way of making sure it could work with health where in the balance we’ve got to throw in health outcomes.

Interviewer:	Okay.

M1:	Does that make sense?

Interviewer:	Yeah, absolutely. For the record, do you think you could repeat—sorry— the name of your unit? I’m not sure now I’m looking over the notes that I recorded it correctly.

M1:	It’s a Unit.

Interviewer:	I’m seeing it’s okay.

M1:	And that’s for our ministry.

Interviewer:	… What exactly is the hierarchical relationship, the relationship between it and the your ministry: it’s part of the your ministry, it consults to your ministry, it’s funded by…?

M1:	We are funded partly by our ministry and partly by another body and our remit is… We’ve got a function for the whole health system to try and improve sustainable development, so for instance, when we do that structurally, we can suggest why it is important for these things to happen. However, with the current political climate, nobody is able to say, “You *must* do these things!” so we need to give them the evidence and a case for change, for them each to decide, “Yes, this is worthwhile and doing,” because we’re dealing with over 600 organizations that have their own individual legal entities who can decide their course of action.

Interviewer:	Okay.

M1:	Our way of operating is yes, we are part of the system, we’re for the system, but we need to influence by demonstrating that the case for change is undeniable.

Interviewer:	Great! Well, thank you. Listen, I don’t want to.... I want to respect your time and so I think those are the questions that we had. Do you have any final comments just about the use of evaluation and your work or questions for the research team?

M1:	Well, I guess it would be useful to understand a little bit more about where you’re planning to take your research and what you’re hoping to achieve with it.

Interviewer:	Yeah, absolutely.

*[audio break/move to next audio]*

M1:	I think there’s so much branding or need for it to be successful that there’s not always the recognition when it’s not successful as one liked, like audits that are so much on the margins compared to some of the other big-scale changes, which is quite difficult to establish a voice.

I guess for us being able to establish a voice that this is material, it’s not immaterial, is really important. And then to be able to hire, you have to trust and respect from people that we do know how to… that the measurement and evaluation is as robust as any better known, even in different areas, is quite key.

Interviewer:	Yeah. That’s encouraging to hear certainly even though as you mentioned those challenges, both on the uptake and the research side.

M1:	Yeah.

Interviewer:	Wonderful.

M1:	So when and where — when you’re finished this — where is it going to go and will you share it with us when you’ve finalized it?

Interviewer:	Of course. Yeah, definitely…

M1:	It’d be interesting to ask.

Interviewer:	Yeah, absolutely. We will be sharing it. I think we’re aiming to be circulating something sort of in this near to December so we’ll be circling back to everybody we’ve chatted with at that time.

M1:	That’s great. Okay, well thank you very much. Let us know if you’ve got any other queries or if you want us to send you examples of things we’ve done. Yeah.

Interviewer:	Yeah. Wonderful! Thank you so much for your time and have a wonderful end of the day.

M1:	Thank you very much. Okay. Goodbye then.

Interviewer:	Bye-bye.

[end of recording]

[total time: 00: 29:12]
