# Supplementary material for: Supporting Better Evidence Generation and Use within Social Innovation in Health in Low- and Middle-Income Countries: A Qualitative Study
Source: PLoS One. 2017 Jan 26;12(1):e0170367. doi: 10.1371/journal.pone.0170367 (PMC5268497; doi:10.1371/journal.pone.0170367)
Supplement: S1 Dataset — (ZIP) [file pone.0170367.s002.zip › Data/Data - Interview transcripts/M2.doc]

Interviewer:	Okay. So, we’re doing a study looking at how different interventions have been selected and scaled up and I liked to understand how do you know. How do you evaluate when the Ministry has been…? How do you evaluate the progress towards your goals? What M&E tools are you using? How do you know when you’re reaching the goals you’ve got?

M2:	Normally we use three important things: the usual reports and the feedbacks and also the super-participatory regions as main pillars of monitoring and evaluation. So what happens with the information system is, for example, through the HMIs and through CHS and some of the other systems that information come in. We usually analyze this information coming in and reports come out like every month, every quarter, every 6 months and every year, and we use these reports as a means to monitor how we are performing against other, say, targets. This is one and also… on a related thing, we do also some surveys at times to see if we are progressing well against the important targets and goals.

The second one is support and supervision. That we do every quarter and every 6 months, so what happens is you have a team drawn from the federal and the region going down to the district, to the government level, to see whether or what is reported is actually even happening on the ground. This is not simply [M&E software? 00:02:16] find, but also an approach of our country, providing support to the areas identified to be strengthened.

The other thing is the feedback system. So whenever you receive something, you look at all the important reports and they give you feedback, and this happens at every level. Like [inaudible 00:02:47] giving feedback to the government liaisons, to the [same inaudible 00:02:51] in regions to the zones, federal to the regions, and so on. So, there is this feedback system and there’s other important things. And besides, the Ministry holds meetings every 2 months with the regional health bureaus and the performances are presented and discussed and directions put forward and so on, so there’s other important monitoring evaluation system that we have in the country.

Interviewer:	What’s the biggest challenge to assessing whether or not you’re having impact?

M2:	The biggest problem is quality data because unless you are receiving consistently quality data, there is no way that you know whether you are doing good or not… There is a problem of underreporting; there is a problem of over reporting. If the data is not actually showing you what’s happening on the ground, then you have to wait for your verification and so on, which is a huge burden, and which is not an easy task because you cannot be everywhere… when you do these kinds of activities, you at some point go to some areas and you do that verification and so on.

So, this issue of data quality has been a real challenge and timeliness of data, the accuracy of data, the [analytically? 00:04:44] of data and so on… How do we know the issues?

Interviewer:	When you’re thinking about new interventions, where do you come up with new interventions? How do you learn about new programs or potential solutions that you might want to see implemented in the country?

M2:	New interventions are basically selected based on certain things. One, if an intervention is proven to be effective somewhere in the work, especially in similar settings, and if there are very strong evidences and so on. It’s very likely that the country takes up this intervention without doing too much kinds of things and there are some interventions that you need to do a pilot in some of the areas and if those interventions are proven to be effective after the pilot, then you go for them…

Generally, with regard to health, there are usually global recommended standard kinds of interventions, recommendations, by the WHO for resource-limited countries and so on. Usually what happens is you have this packet from the WHO and then we kind of adopt those kinds of things to our country’s situation. But at times, as I said, there is a need to do a pilot. You pilot, you test it, and if that’s going to be okay, then we scale it up across wider geography. If the interventions are home-grown, they are home-grown and they home-grow somewhere. That is why we call it based experience, sharing and scale up, so we kind of design that. Once that best practice is doing something fantastic in some area, then that is packaged and shared to the other area through different forum like the Joint Steering Committee meetings that we have with the regional health bureaus every 2 months and through the other annual review meetings.

By the way, on the M&E, we also have these review meetings, like we have the annual review meeting which happens once a year and we have the program-specific review meetings like for HIV, for RMNCH, for disease and so on, so these program-specific reviews are also important to do the M&E.

Interviewer:	So for interventions that you learn about the outside world, you sometimes learn about them from WHO standards but your country tends to do things faster than a WHO standard. What are some places that you’ve learned about interventions that you decided that you would try here before WHO would adopt? Is it things like journals or going to conferences or having other partners pitch you on ideas or suggest ideas?

M2:	Yeah. At times, we come across some very important, interesting kinds of things, like for example Community Based New-born Care—is a very good example. … It is designed to target diseases due to neonatal sepsis, especially for children born in the community, at homes. This is something that our country did a pilot in two regions through the combined trial and then we built our own evidence so through roll-out of this CBNC in these pilot areas, the neonatal mortality has by decreased by close to 35% so this is something that the literature has not recommended or has not doctor- recommended it to resource-limited countries, but our country has adopted this, piloted it, and tested it and we are scaling it up across wider geographies across the country. So, sometimes we do that.

Interviewer:	Why did you do it for that? What convinced you that it was worth doing a pilot?

M2:	Because neonatal mortality is a huge problem and maternal mortality is a huge problem, so we constantly look for any better means of curbing these mortalities, reducing this matter in neonatal mortalities in our country. Still, even though the institution of delivery has seen a very significant trend over the last 5 years particularly and now it’s at 60%, still 40% of mothers are delivering at homes which increases the risk for the illness—the neonatal sepsis, neonatal infection, so on.

So at that time when we decided to go for the CBNC, the institutional delivery was lower. It was close to 15%, so 85% of mothers were delivering at home so the risk of neonatal getting sepsis was very high so we’re like very much craving for any kinds of intervention that could address all these problems and so on so this Community Based New-born Care idea came about so we looked at it and we piloted it.

Interviewer:	Where did it come from?

M2:	It’s from an organisation’s project. It’s Saving Newborn Lives.

Interviewer:	So did they approach you and say this is an intervention project?

M2:	Yes. They approached us with this packet of CBNC and we looked it at it. It was very tough because they… One thing. This is implemented through the Health Extension program and the health extension workers are supposed to give gentamicin for 7 days and amoxicillin straight for 7 days. So, the issue of health extension workers giving gentamicin injection to neonates was a real issue and there was an intervention arm and there was a control arm and there was a huge ethical delayment that we had to deal with, so there was a little bit of a resistance at the beginning, but later on that was piloted and proven to be effective and when the combined trial result was out, it showed that this intervention actually helps to reduce neonatal mortality by 35%. When you compare the intervention arm with the control arm, those in intervention arm had neonatal mortality decreased by 35%...

So, that was a case and after that combined trial evidence came out, we went for the scale up of this intervention across a wider [inaudible 00:13:20] geography.

Interviewer:	Now when this organisation brought it you, did they come with data from other places showing that it had been efficacious already?

M2:	Yes. I think they had some evidence from another country, I think, and they presented that data, but the fact that it works in another country doesn’t mean that it works in our country — the setting’s different and so on. We actually liked the idea, but wanted to pilot it before we went to scale. So, yes, there was some evidence that it came with from the other countries and it was very interesting and there were some evidences that it actually contributed to reduction of neonatal mortality. That was why we were actually interested as the Ministry to go for that intervention, so we went for it and I think that was rewarding and it’s being scaled up.

Interviewer:	Was it important that it came from this organisation, from an organization that you had a relationship and knew had a good reputation? Or was that not a factor?

M2:	I mean relationship is always good, but it’s what you bring, which is actually important. Who brings it… If you have a credibility, a good track record with the Ministry then that helps, but what you bring—the quality of product that you bring— is very important and the amount of evidence that you bring is very important and the impact this intervention had in other countries in evidence of it is important. So, it’s not only the question of who brings it, but what is inside of it and what contribution could it make and so on and the evidence that it brings is very important.

Interviewer:	How about for the home-grown ideas? How do they bubble up to the top? How do you find those?

M2:	For example, you have a certain intervention which is working very well for example in another location, then what happens is using this Joint Steering Committee platform, this other location shares basically this experience with the other regions and they present it, relating it to some of the other important targets of mortality reduction and improving health and so on, and [if] the other regions would be interested to look at this experience. So, those regions which are interested would take an extra step and go to this other location to look deeply into the issue and take it up so that is what usually happens and the platform is the Joint Steering Committee, but there are also some other platforms that regions use to share experiences and so on.

A very good example is the Maternity Waiting Home. This is something that was first initially piloted in another country in one particular hospital run by the Catholics. So due to availability of that Maternity Waiting Home, mothers who are supposed to travel long distances to get delivered in health facilities started coming to give birth in these health facilities because they can stay in maternity homes a few days before delivery and a few days after delivery and so on and that helped that specific district to have the institution of delivery go up, so that experience was shared in the other regions. Now, it’s like all over. If you go to *any* health center across our country, then you find a maternity waiting home basically constructed by the communities — financed and everything by the communities.

Interviewer:	So it sounds like when an intervention is going to be scaled up, you need some evidence that that it’s worked — outside of the country and inside of the country — if it’s an important intervention, that it has to meet a big need. Otherwise, the Ministry won’t push for it. Then, it also sounds like it has to be agreed on and approved at regional and zonal levels so that there’s a groundswell of support to scale it up…

When you work with NGOs in the health space, what are some of the most successful aspects of working with health NGOs in the country and what are some of the biggest challenges as they come up with new interventions and want to help implement them?

M2:	Most of them come up with some innovations… most of the time. They have some capacity, whether it’s technical or financial, so that’s like kind of opportunity… and most of the NGOs would like to really align what they do with what the government’s doing and they want to align their priorities with the priorities of the country and so and that gives a lot of energy to the implementation of these priorities.…

The opportunities… They have innovations, they have capacity, they have resources and so on. But on the other hand, some NGOs actually do things which are not aligned with the Ministry or with the plans and so on and there is very much haziness, un-clarity on what they do and what they are doing and so on. We do resource mapping every year, forward-looking, and we usually try to trace these NGOs and so on, but this is not always successful and tracking what they do, what they are doing, and amount of resources they have and so on is not clear, so that is the challenge.

Interviewer:	What would be the best scenario for an NGO with a new intervention that they believe to be very efficacious to solve a known problem that you have, what would you like to see so that that would be most useful to the government and then eventually to the people?

M2:	What should happen in my opinion is actually for those NGOs to come direct into the Ministry and have the discussion with the relevant directorates. If it is, for example, an MCH intervention, then come into the MCH directorate, presenting what they have actually, and how this could be integrated with what the government is doing and so on and see and also to be transparent in what they have, actually. Be transparent in how much resources they have for that initiative or for that intervention and so on and the flexibility of accommodating the feedback and the comments from the Ministry is very [inaudible 00:21:59].

So, continuously engaging with the Ministry and consulting with the Ministry and being transparent with what you have and so on, how you plan to do it and so on is very important. Once you have this consensus with the Ministry, then cascading it down to the lowest level is not going to be difficult. But sometimes, they go somewhere without informing the Ministry and they... I can give you a very good example. Our country has a very clear stand on result-based financing, so an NGO went somewhere without the knowledge of the Ministry — and in fact the region — and engaged with a zone. They started doing something which is contrary to the RBF (result-based financing) position of the Ministry so that ended up being suspended and they have to do everything all over again, which is a disaster for that NGO and a disaster to the health sector because that resource is simply spent in vain in something that the Ministry doesn’t appreciate it, to have any contribution.

Interviewer:	Great. Thank you.

[end of recording: 00:23:33]
