# Supplementary material for: Supporting Better Evidence Generation and Use within Social Innovation in Health in Low- and Middle-Income Countries: A Qualitative Study
Source: PLoS One. 2017 Jan 26;12(1):e0170367. doi: 10.1371/journal.pone.0170367 (PMC5268497; doi:10.1371/journal.pone.0170367)
Supplement: S1 Dataset — (ZIP) [file pone.0170367.s002.zip › Data/Data - Interview transcripts/M4.doc]

**ROUGH ENGLISH TRANSLATION OF INTERVIEW CONDUCTED IN A FOREIGN LANGUAGE:**

How did you hear about this health organisation? Why did you decide to work with them?

Answer: I knew the this health organisation during the presentation of the project to the National Team Program against Malaria (NMCP). We decided to work with them because of project content whose activities are consistent with the visions of NMCP especially taking part in community case management of malaria.

What is the greatest challenge of the Ministry of Health?

Answer: The reconciliation of the people quality care to reduce the burden of communicable diseases in general and malaria in particular

How do you learn about possible solutions to health problems?

Answer: We use the results of research (operational, Action ...) carried out in our country and in other countries.

Are there examples of innovations or interventions that the Ministry of Health has a scale of the whole country? Why did it-they were chosen?

Answer: Since 2011-2012 the Ministry began implementing the SEC Policy (Care Essentials in the community) in the country. This choice was guided by... the results of the experiences of other countries.

What kind of evidence did you confidence that this health organisation done a good job?

Answer: field visits to the reports of my team members, the year-end results that this project and especially the result of operations research that has been published.

6. What tools do you use to evaluate the progress of the Ministry of Health & this health organisation? How do you use the data?

Answer: In country tools PRODESS devaluation standards are used to evaluate any health program or project. Regarding this health organisation, evaluation is done through field visits and review of annual reports that the present project. The information thus produced are made in the annual report of the NMCP is compiled achievements of all stakeholders in the fight against malaria in our country. This report is presented in strategy meetings of the Ministries of Health as a basis for decision making.
