# Supplementary material for: Supporting Better Evidence Generation and Use within Social Innovation in Health in Low- and Middle-Income Countries: A Qualitative Study
Source: PLoS One. 2017 Jan 26;12(1):e0170367. doi: 10.1371/journal.pone.0170367 (PMC5268497; doi:10.1371/journal.pone.0170367)
Supplement: S1 Dataset — (ZIP) [file pone.0170367.s002.zip › Data/Data - Interview transcripts/M5.doc]

Interviewer:	Okay, so M5, just for the record, can you state your full name, what your position is, and who you’re representing?

M5:	I’m a clinical director for non-communicable disease in our country.

Interviewer:	Okay. Just a nice question to start off with: can you tell, in your role, what achievement of your country has your department been most proud of? And can you tell me a bit of background about how that story happened? So a story of success.

M5:	A success… This is a new role. I haven’t completed four months yet in this role so…

Interviewer:	Okay.

M5:	I haven’t really worked in many big projects yet, truthfully. I see patients and we’re involved in a lot of strategic planning right now for NTB and also for the national health plan. And we also do community work and do outreach clinics… and I just finished working on the diabetes management guidelines for RMI. I’m getting feedback on that and we have regular team meetings.

Probably the best thing right now is the team— the team work. I can’t say I’m especially proud of anything at the moment because I haven’t really worked on any project. I haven’t worked on any project.

Interviewer:	No. It sounds like you’re doing a lot already. So with a lot of the projects that you’re working with, what do you think out of all the projects is the biggest issue or challenge your Ministry of Health is facing at the moment?

M5:	Okay. Well, the Ministry of Health… Actually, diabetes is the real problem. [REDACTED]. I come from a diabetes background when I worked in Fiji so one of the problems that we’re trying to tackle is foot—like preventing foot amputations—and we work together with [REDACTED] charities and... some other partners to come up with a foot proposal— a foot training proposal, and our Ministry and the doctors within our Ministry Medical Society, we’ve put together a proposal to actually spot a high-risk foot clinic, like a separate clinic with staffing and everything, and that’s one of the things that we’re working on. Like I said, nothing has actually been completed yet. It’s still a dream at the moment and we’re working towards that.

Interviewer:	No, it’s slow steps, really. It definitely sounds like it’s an issue that you’re starting to tackle.

M5:	[inaudible 00:03:53]

Interviewer:	Sorry? Hello? M5? Can you still hear me?

M5:	Hmmm?

Interviewer:	Can you still hear me? Sorry, it just cut off a bit.

M5:	Yes.

Interviewer:	Can you tell me how do you evaluate your progress towards particular goals, like whether that goal seems distant in the future or whether it’s quite close? What kind of tools do you use to monitor how you guys are going?

M5:	Like is this just in relation to the foot project or is it in relation to all of the things as well?

Interviewer:	In relation to all— everything that you do.

M5:	We have a data management, like a chronic disease management system that we use and so we can put in like... For example, I could pull up a report and say, “How many patients turned up today and how many patients had their such-and-such lab work done and what’s the percentage of this and that,” so that’s one of the ways. That’s the strongest thing—that data is quite good. That’s what we use for monitoring in the clinic and also monitoring some of our creation stuff, stuff like that.

Interviewer:	So that’s an existing…

M5:	I guess the other stuff is just like… Yeah, that’s an existing data management system, electronic management system.

Interviewer:	Okay. Sorry, I cut you off there. What was the other thing that you were starting to say?

M5:	Oh, no. I was just trying to think is it really called a data management system. Anyway, it’s a chronic disease electronic management system so it’s like a patient management system.

Interviewer:	Okay. And in that management system, what are some of the outcomes that you look at to see how your progress is towards your goals?

M5:	… We would look at the average HbA1c levels for patients so that’s one of the things, like how well patients are being controlled, what percentage of patients are receiving statins and what percentage are receiving ACE inhibitors, things like that, and the percentage of the total of patients that have a complications assessment and things like that.

Interviewer:	Okay. And so other than…

M5:	That’s more of a…

Interviewer:	Sorry. Hello?

M5:	It’s more like a clinical management of patients, like individual patients. That system is more… [audio drop 00:07:09] and quality assurance for the clinic.

Interviewer:	Quality assurance for the clinic? Okay. Other than the collecting data from that electronic chronic disease management system, are there any another monitoring and evaluation tools that you already have in place?

M5:	Yes. We have a monitoring and evaluation framework. That’s part of the plan. All of the plans that I talk to you about? All of them have those: plans for monitoring and evaluation with all the indicators and things like that.

Interviewer:	Can you give me an example of what you include in one of the monitoring and evaluation frameworks?

M5:	For the foot proposal, this would look something like… It’s for foot training so with all the sureties we want to train the trainers so staff will be trained to teach other staff how to manage the foot, so some of the indicators will be like number of trainings conducted by those who have been trained and then things like that.

I know that there are proper ways of saying all of these things: outcome, process indicators and everything… They’re all different so I don’t have the document here with me and...

Interviewer:	No, that’s alright.

M5:	…my thinking is not so…

Interviewer:	No, no, that’s alright. We just want a general understanding of what you include with your proposals and plans. Okay. Hello?

M5:	Just turning on my laptop.

Interviewer:	That’s alright. Are you okay if we move onto the next question? Hello? M5?

M5:	[inaudible 00:09:46] Sure.

Interviewer:	Okay. I’m interested to find out how you identify potential solutions to health problems? You talked a little bit about the diabetic foot clinic. As a Ministry of Health department, how do you learn about solutions to these health problems that you’re trying to tackle? So, for instance, do you go out and talk to other Ministries of Health or do you find about them in the media, when you go to conferences?

M5:	For me, I would think… ‘Cause I’ve been working in the diabetes area for a while and my experience from Fiji. I used to go to conferences for diabetes and I also had training in Sydney for diabetes and so I think—and largely from my experience working at the Diabetes Centre in Fiji—that’s what I bring here for the training and the solutions and the things like that. From the conferences, there’s no app for it.

Interviewer:	There’s no what? Sorry? Sorry, I didn’t quite catch what you said there.

M5:	There’s no app for it.

Interviewer:	Okay.

M5:	… No app, like a mobile app. One of the ideas…

Interviewer:	Yeah, no. It would be so much easier if there was just a simple app.

M5:	Yeah.

Interviewer:	So would you say that most of what you guys end up implementing is based on experiences of people working in the health sector on the ground?

M5:	Yeah. We’ve also do a bit of reading as well so what are the most cost-effective… We also find out some things like what worked in other countries like a similar foot proposal and a similar project I had background knowledge on, like some of the projects that were funded by the World Diabetes Foundation in the Caribbean. We’ve seen the foot projects and stuff like that. It’s also from reading on the Internet. And some journals and things like [that].

Interviewer:	Okay, that’s great. With the interventions that you end up taking up, I just wanted to get an idea about which ones you end up implementing or scaling and then which ones that you *don’t* end up implementing or scaling. Can you compare and contrast an idea that you’ve taken up versus that an idea that you decided not to take up?

M5:	When I was at the University, we worked on a project to take back to Fiji. It was a project like a mobile health application using community health workers, but in the area of diabetes. That kind of project… I had everything done for that, all the project planned—the whole thing— ‘cause it was a group project we worked on and it was for Fiji, but that kind of project will not work in our country because the Internet service is not so good. … Wi-Fi is only in certain areas. People don’t have Internet. It’s like that, so it depends on the area that you’re working in, and I think a mobile phone usually not as high as in Fiji.

So, that was one of the things I thought I could bring here, but then they don’t have a supportive environment for that. I guess some of the other things based on what is available in the country. For instance, here, we wanted to do something based on nutrition but we don’t have a nutritionist or a dietician in the country, so, yeah, it’s based on resources available in the environment as well for some of the interventions...

Interviewer:	Have there been any really, really great ideas—similar to what you just talked about with the diabetes mobile technology in Fiji— that you’ve also wanted to bring across but for some reason it hasn’t been possible?

Unknown:	The promotional materials.

M5:	Oh, yeah! We wanted to do like health promotional materials and do a lot, like focus on patient education as a whole, but what works in a certain place is probably not acceptable, perhaps because of the literacy level as well here. We had already made some brochures and things, but it’s not being accepted by people because they say, “All people don’t read over here! You can’t have brochures or anything like that. It’s not going to be effective. It has to all be in their language ‘cause nobody reads English.”

Unknown:	[inaudible 00:16:39]

M5:	Another challenge as well is the workforce here is very… A lot of them not really qualified for the post they hold so people working in health promotion, they could just have experience maybe working in an organization but they didn’t go to school to learn anything about health promotion at all, like that sort of thing… Are we talking about challenges? I forgot the question. I’m sorry.

Interviewer:	Yeah, so just talking about things that you’ve wanted to bring over to your country but for some reason or another it hasn’t quite worked. Yeah, okay.

So, in each of those cases, can you think specifically about pieces of evidence that you may or may not have used to influence your decision to take up an initiative or not take up an initiative? So we’re talking about evidence.

M5:	Evidence… Evidence like value of having locals and sustainable programs like the training of people, local people to train themselves? I think the evidence of those things… I’m assuming that it’s already known because they have been a lot of research on that... involved in the foot project and even for foot care, but honestly I didn’t read anything recently. It’s just…

Interviewer:	Okay. And can you tell me a bit more about what influences your decisions about whether to take a small project and scale it to a national level? What kind of things influence that decision?

M5:	Obviously, if it’s successful and if it’s affordable and there’s a lot of support in the community. There’s also support from funders and it’s acceptable by the community people and it can be sustained like on a larger scale.

One of the things that we’re working on is to have a community lifestyle program since diabetes is such a big problem, so what we’ve been doing is we’ve been going to communities and having, like, workshops— evening workshops— every few weeks in a row. We do screening and then we do exercise (within that session) and it’s usually in a community setting, say for example Muslim church community or LDS Church or community, so around that area people are invited to come.

So, we do all the screening and the exercise, aerobics. Then, we also have a little cooking class demo. Then, we do education like on promotion, health prevention and stuff like that. But the aim of this, what we’re doing is that so when we leave an area, if we do the program like 4 weeks in a row in that same area, the same community, after we leave, we are hoping for the people— for them to start up their own committee and be kind of energized to keep on that so as we move around the island to all the different communities, they can all have their own little committees where they do exercise and they have their walking clubs and they keep physically active and they continue with that, and then we would go and visit them.

But one of the problems that we’ve encountered so far is that when we leave, people don’t really continue with that, so the way that we do [audio drops 00:21:52] is we re-look at it, to make it sustainable so we’re thinking about maybe getting funding for it, to have the person who stays behind—the leader of the program who stays behind and does (within the community) carries out the physical activity and does that maybe have an incentive for them, whether it’s pay or something else, but there needs to be something to make it sustainable.

We’d look at how successful things are and obviously since sustainability’s a problem, we’re not going to upscale that kind of model. We have to [see] how things work and if they’re able to carry on, then we would. If it’s successful, we’ll look at that and then make it go— upscale it to nation.

Interviewer:	I see. In the example that you were talking about, you know that the program works at a small community level when you’ve got your staff there working together with the community and so these factors that you’re talking about: whether the program’s successful, whether it’s affordable, whether there’s community support, how do you measure those things? How do you tell with each of these factors whether you’ve reached your success point?

M5:	Well, when we leave, ideally we want that satellite clinic, that group to keep on going, to have regular weekly—at least weekly—meetings and to have physical activity to carry on. I think that was the biggest indicator for us. But then when we left, there was nothing going on, so [audio drops 00:23:50] indicators...

Interviewer:	Okay, so it seems like one of the big barriers for this project in particular is that it hasn’t been sustainable in the long-term. Was this something that you were thinking about at the beginning and was there anything that you tried to keep track of to try and ensure that it would be sustainable?

M5:	This is a joint project with the [REDACTED] and also the Ministry of Health, so yeah it’s not completely all about the planning part of it. We were just there to support and then after that we got into the planning of it as well.

But when we first started, we thought, “Okay. We’ll get them to come 4 to 5 weeks in a row,” so the first one we made them come every week for 5 weeks. One of the indicators that came up was to make sure they complete all the 5 weeks. We developed a little passport for them. We would stamp it each time they come and each time they’d come, they would receive a raffle ticket ‘cause raffles are quite popular here so we’d have a raffle.

Then right at the last… the final graduation, they would receive another [and] the raffle would be drawn. The people that would be there would be in the raffle so that was one of the incentives that we made to make sure that they’d come each time, but then I think we didn’t really think about after— what was there for them after— and I think that’s one of the areas we have to re-look at and we’re working on that.

Interviewer:	Okay, so I think you’ve briefly talked about some of this already, but can you list some of successes and challenges that you’ve had working with health not-for-profits or non-governmental organizations in your country?

M5:	Successes…

Interviewer:	Successes and challenges.

M5:	Okay. For not-for-profit… I don’t know whether Taiwan… That doesn’t come under an NGO, right?

Interviewer:	Taiwan? Hello? Sorry, M5?

M5:	They have a, like, … [audio drops 00:27:07] a small health branch that works with our Ministry, but that wouldn’t be an NGO.

Interviewer:	No, not quite.

M5:	That’s like a government.

Interviewer:	Yeah.

M5:	Or organizations with us. What do you think? … one of the challenges with other, like NGOs, is the fact that the NGO. They have their agenda and they work with you only up until, like, if it’s according to what they’re doing as well. One of the things is it can lead to a lot of duplication stuff.

It’s good to work with NGOs because they work with the grassroots people so they’re connected to them and we’re able to come in there and... I’m saying lots of words and I don’t think they make so much [sense] but I hope you understand what I mean.

Interviewer:	Yeah, no. I know what you mean.

M5:	I write better than I speak.

Interviewer:	Okay.

M5:	Hello?

Interviewer:	Yeah, hello? M5??

M5:	I’m here!

Interviewer:	Okay. Just…

M5:	Yeah.

Interviewer:	Hello? M5? Hello? Hello?

M5:	Hello? Yes?

Interviewer:	Yes, I can hear you. Can you hear me?

M5:	I’m here. I haven’t left.

Interviewer:	Okay.

M5:	Yes?

Interviewer:	Hello?

M5:	Yes.

Interviewer:	I think there’s a bit of lag. So just on the topic of the…

M5:	Yeah?

Interviewer:	… challenges working with…

M5:	Okay.

Interviewer:	…NGOs, so are there any other specific challenges you’ve come across, things like costs or funding or whether they actually have an impact?

M5:	[inaudible 00:30:02]

Interviewer:	Or regulatory challenges?

M5:	Yes, definitely. [audio drops 00:30:14] Yes, yes, definitely with funding. Some of the NGOs we work with don’t have money so they always… Yeah, funding problem.

Interviewer:	And is that something that you help NGOs address?

M5:	Hello?

Interviewer:	Hello? Is that something that you help NGOs address?

M5:	… Yes, yes, we try to as a Ministry. Yes, we do try and ... [audio drops 00:30:48] Yes.

Interviewer:	Okay.

M5:	Yes.

Interviewer:	So how do you go about doing that? Do you provide funding directly or do you provide other types of support or do you provide networks?

M5:	The Ministry works directly— gives funding to NGOs and working within the NDG program. And also, for example, if we have a project that we work together on, then we look on the sorts of things that we can fund like if it’s the trainings or the equipment or the T-shirts, or whatever. Whatever things that we can fund, then we look at it and, yeah, we do help with funding.

Interviewer:	Okay.

M5:	Even then, the Ministry itself also can’t fund everything and they’re also some… ‘cause the Ministry itself is reliant on other funding.

Interviewer:	What kind of funding do you have as a Ministry? Where do your funding sources come from?

M5:	Most of the funding, I think, comes from the compact, from the United States of America, and a lot of the health programs are funded by the CDC in America. We also have bits of funding from WHO, but most compact funding and CDC.

Interviewer:	okay. So M5, we’re coming towards the end of the interview. Two more questions. I’m quite interested in knowing what are the requirements for the our Ministry of Health taking up a new innovation. You talked quite a bit about the diabetic foot project. What kind of factors give you a reason to take up this new initiative?

M5:	Can you just repeat the question again?

Interviewer:	What are the requirements for a new intervention to be taken up by the Ministry?

M5:	Am I representing the Minster of Health or…?

Interviewer:	Just as a department, the Ministry of Health.

M5:	As a Ministry of Health… Well, I’ll try and answer, but I really can’t say for them as the Ministry of Health because I’m only new here. I don’t know exactly all of the workings, but I think if the new initiative is doable like if we funds available for it, is there going to be staff who can do the training? Are they going to be… If we have space for the clinic?

And of course, if the problem we are proposing to address so for example, foot amputation, if that’s a big enough problem for this place, then we would take up that initiative and it is a big problem here so this initiative is going to go ahead.

Interviewer:	Okay. And so, final question. Can you tell me in a perfect world, what do you think would help you improve access and delivery to health services in the your country?

M5:	What would improve access and delivery…? Access and delivery to what?

Interviewer:	Health services.

M5:	Is it just health in general?

Interviewer:	Yeah.

M5:	If… improve access and… What was it? Access and?

Interviewer:	Delivery of health services.

M5:	Okay. If we had the right number of health workers, if we were close to all members of the population alike. For example, we have outer islands and they have been isolated from the main health services and the level of care they receive is not as good as people would have in the urban areas, so that and also affordability of service… so health workers, if you have enough health centers or dispensaries, a reasonable distance to all of the people …

For example, it’s a user-pay system here so when patients are sick, they have to pay a fee and everybody pays the same amount. If there could be some equitable distribution of the services and ... patients don’t have to spend a lot for healthcare, I think that would be good. Yeah.

Interviewer:	Yeah, okay. If all of those things could happen, that would be amazing, wouldn’t it?

M5:	Mm-hmm.

Interviewer:	Okay, M5, that actually brings us to the end of the interview.

[end of recording: 00:37:35]
