# Supplementary material for: Supporting Better Evidence Generation and Use within Social Innovation in Health in Low- and Middle-Income Countries: A Qualitative Study
Source: PLoS One. 2017 Jan 26;12(1):e0170367. doi: 10.1371/journal.pone.0170367 (PMC5268497; doi:10.1371/journal.pone.0170367)
Supplement: S1 Dataset — (ZIP) [file pone.0170367.s002.zip › Data/Data - Interview transcripts/M6.doc]

Interviewer:	So, M6, just for the record, can you just state your full name and what your position is?

M6:	M6, minister of the Ministry of Health but I thought it’s was an anonymous system.

Interviewer:	Sorry. What was that?

M6:	I thought it was anonymous.

Interviewer:	Yes. We will anonymize the data, but when we’re analyzing we need to know how to classify it so we will anonymize the data once it’s presented. Don’t worry about that.

M6:	Okay.

Interviewer:	So, M6, I just wanted to get a really good idea about basically what you think is important as working in the Ministry of Health, so can you give me an example of one of the success stories that the Ministry of Health is most proud of?

M6:	One of the success stories would be, to me, attributing the MDGs so we were able to achieve most of the MDGs of most everything that were in the last couple of weeks ago. We were able to achieve child mortality, infant mortality. We were able to reduce maternal mortality as well as neonatal mortality. That’s in general, but we’re also very proud of the PMTCT achievement where we have less than 1% HIV transmission from mother to child in matters where HIV affected.

Interviewer:	Wow, that’s really great! So with the successes that you’ve had and maybe the not-so successfully stories as well, how do you go about as the Ministry of Health evaluating the progress towards your goals?

M6:	We are doing fairly well. The thing is that we’re on a very good track and the thing is to maintain that momentum and still continue working so that we can also meet the SDGs.

Interviewer:	Yeah. Do you have ways of measuring how you’re going?

M6:	Absolutely. We have 13 different systems in our country that help monitor the progress that we’re making so we have the HMIs, the health system in which all the health centers send their monthly reports about any consultation that they make, any mobility of any of these spaces, so that’s one. We have also the rapid estimates, the trucks, the pregnant women in the community and it sends alerts when we need to act and we have also the pharmacy, the electronic logistic information systems so we have a lot of different tools to measure progress that we’re making.

Interviewer:	And so, it sounds like you have a lot of sources of data and you collect a lot of data that’s quite comprehensive. Do you have particular monitoring or evaluation tools that you put the data into to be able to get something more meaningful and something that you can use to assess how you’re going?

M6:	Yeah. One of the systems can give summaries so we draw summaries and we can even visualize some of these things on some maps, like the HMI system can visualize where we are above the threshold that we set or things like that, so we do better visualization with maps but we also do summary with the indicator. But on a regular basis, we also send reports to the Prime Minister of these reports that we have in-house in the Ministry.

Interviewer:	Okay. I know it’s going to be quite difficult to define, but can you give me an example of some of the outcome indicators that you specifically try and look at to try and see whether the interventions that you’ve implemented are actually helping you achieve your goals towards the SDGs?

M6:	Oh, like maternal mortality? That’s one. Infant mortality—that’s another one. We look at the number of pregnant women who have attended the antenatal care and how often: once or four times. We look at the HIV testing on a monthly basis. We look at how many people were put on treatments. We look at the malaria incidence. We look at the TB incidence, for instance. So, we have plenty of those. We have a lot of different indicators to monitor the progress that we’re making.

Interviewer:	Yeah. No, that’s great. It sounds like you’re keeping a good track of what you’re doing. So with regard to trying to achieve the NDGs the and now the SDGs, there are lots of health problems that as a Ministry of Health you need to address. How do you learn about potential solutions to solving these health problems?

M6:	One thing is to read the literature, see how others have done it. That’s one of the first things, but also have technical working groups meet on a regular basis and analyze the data that we have and put it into context so that we draw solutions that are customized to our settings. Then, we implement strategies and see if they work so we move through them and we adapt them as we go.

Interviewer:	Okay, so it sounds like there’s a range of sources where you learn about solutions that you try to implement. Can you compare and contrast two examples, the first of which is an innovation or intervention as a Ministry of Health you’ve embraced and scaled up as part of a national health system and contrast that with something that maybe you decided not to embrace and scale?

M6:	One thing that we’ve scaled up is the HIV treatment where you have HIV treatment that’s provided in almost all our health facilities up to the health centers. That’s something started back in 2004 in very few hospitals—specialized hospitals— and then spread out to district hospitals then to health centers.

Initially, those treatments were provided by specialists only and then by general practitioner and then we did a task shifting and now treatment’s provided by nurses, so the task shifting piece is one thing that has been tried out in 3 places and then scaled up to everywhere in the country. That’s one example that we have.

Something that hasn’t worked out? I’m trying to find an example, because here we have pilots so we usually adopt only strategies that are scalable and that are [inaudible 00:07:31] so I’m trying to find an example. Let me see if I can find something that hasn’t worked off the top of my head…

Interviewer:	Or perhaps it would be useful to talk about what aspects of the HIV treatment project made it easily able to safe scale up at a national scale, so what kind of factors influenced your decisions to scale that up?

M6:	Firstly, one of the things is the equity and equality among the people, so we have one principle— one approach that has to apply to every intervention that we have. It has to be people-centered and it has to be provided across the whole country. That was one. So that not only people in one specific region reap the benefits of one intervention. That was a major problem.

Second thing is that it has to be evidence-based, obviously. It has to be something that works well and that’s proven to be working well. And another was to be something that’s relevant and sound for our context and that align in our strategies, so that’s like the task-shifting thing. We realized that there were a lot of people who were not accessing treatment because we didn’t have enough doctors across the country but we also realized that nurses could provide the treatment so we said let’s scale up these task-shifting initiative. Those are things that are sustainable and scalable, not things that will just be implemented in the region and never grow to be implemented in the whole country.

Interviewer:	Okay, I see. Okay. So just going back in particular to being evidence-based, you mentioned that it’s important that the projects you end up scaling up, they work well and they’re proven to be working well. Can you just in a little bit more detail, describe what kind of evidence do you look at when you’re trying to assess whether it’s working well and whether it’s proven?

M6:	As I’ve said, it’s the literature first, so to see what has been done elsewhere and looking at our own experience and see how we’ve been operating because for the task-shifting, some consultations at health centers were already done by nurses so they could already prescribe some treatments so we knew that being that innovative was not that much a risk because they already did that and that was already working well.

Interviewer:	Okay. So with the literature that you look at, what level of evidence do you accept as showing that something works well, so are you talking about like academic research papers or…? What kind of evidence do you accept as…

M6:	Mostly, but there is also various way[s] of balancing it out so if it has been proven the most, the most one is a randomized trial— the hierarchy of evidence—but also for some of these things we get very innovative and we balance the benefits and the risks so when we find out that the benefits outweigh the risks, we sometimes say to them, “Go” without necessarily having the highest of all evidence and then we monitor and we rule on that as we go.

Interviewer:	And then just regarding your own experiences as well, so you talked quite extensively before about the data collection and data management systems that you have in your country. With your own experiences, do you set up formal pilots and formal evaluations for the projects that you decide to talk on?

M6:	Very rarely because what we discuss is usually we take on projects that are very beneficial to our people. So the Phase Ones because we hate the word “pilots”, Phase One is usually very short and very conclusive because of the nature of the project that we take on.

Interviewer:	Okay. And do you possibly have any examples of maybe projects that you’ve decided not to take on and reasons why?

M6:	Let me see. I’m trying to figure out because I’ve been in this position for a year and through the year I’ve been here there is none, so I’m trying to dig.

Interviewer:	Oh, wow! So you’ve had a very successful year. Everything has been successful.

*[shared laughter]*

M6:	This year was successful.

Interviewer:	Oh, that’s very good news for you then.

M6:	That’s quite good. Let’s see. What hasn’t worked or what’s limited to very short settings?

Interviewer:	That’s alright. We can come back and revisit that question if you’d like.

M6:	Yes, sure, because I can’t think of anything right now.

Interviewer:	That’s okay. That’s perfectly fine. Okay, so you’ve talked about it a little bit— about what requirements make you decide that you want to scale up something to a national scale— but with new project ideas that you’re deciding say whether to pilot them in a small community or take them on for a first time, are there different requirements that you assess as to whether to actually just take up an idea or not?

M6:	Absolutely. The very first one is the safety. How is the initiative safe? How does it fit into our strategy? How does it respond to our needs and questions? How it will be perceived by the community? How are we going to involve that community to accept it? And the outcome. How is the outcome?

Then, you have all the balancing between priorities, competing priorities that we have in the health sector, so those are the things that we usually consider. The outcome. How good does it… because one of the principles here is to have a healthy and wealthy population, but also to be able to increase their level of development and their well-being and the wealth that they have, so that’s things that we put into the equation as well.

Interviewer:	Okay. And does evidence and literature play any part in whether to take up new innovations?

M6:	Absolutely. We’ll never take on things that haven’t worked yet… like look at that circumcision that we are taking on here. It’s because of the study that came out that shows that circumcision is protected for HIV up to 60%. … And such a project would never be took on if we hadn’t that kind of evidence.

Interviewer:	I see. Okay. It sounds like it’s definitely a factor in both taking up new innovations as well as scaling up innovations to a national scale.

M6:	Yes.

Interviewer:	Okay. Moving onto particularly working with health NGOs as a Ministry of Health, what kind of successes and challenges have you had working with non-governmental organizations?

M6:	They’re very few. In the first place, there were some NGOs who really want to align but we made sure that they aligned pretty quickly because we have a good size of what these NGOs do. We have a whole strategy to receive and welcome them and to orient them about the national objective and how the working relationships would be and their reporting as well. So, initially, when it was well-defined, it was a challenge because some would do what they want but very quickly we have align to the country priorities and that’s the thinking and we have a forum where it’s always very conciliatory and we exchange different points of view.

Interviewer:	Okay. You mentioned that you orientate them and you establish working relationships and also reporting requirements. Can you go into a little bit more detail about what you expect from NGOs when they decide to take something on and you decide to work with them?

M6:	One thing that we expect is that they know our plans so that they know where we are going, so that’s one thing. And they actually fit into our national priorities— that’s something— and they are integrated in the community where they work so they usually work with the local communities and local governments where they are established. Then, that they provide reports and show progress on the different actions that they are making.

Interviewer:	And what kind of things do you expect them report on? Do you, like, put parameters on what you want them to report on?

M6:	Absolutely. Every NGO that works has to present a national plan before we accept them to work in the country, so they submit their action plan or we do it together and then we orient them and then we set indicators that they will be reporting on. And usually the indicators do fit in our national indicators as well so it’s never something that’s standalone.

Interviewer:	Okay. That’s great. Okay. So, M6, we’re actually coming up to the final question. Just wondering, in a perfect world, what do you think would help you in the Ministry of Health improve access and delivery to health services?

M6:	Well, to employ doctors. We need to have the health facilities that we need everywhere so now we’re embarking on a strategy. We have the way our health system is organized. We have the [inaudible 00:19:14] care, the referrals and therapy rates up and then we have provincial for the region and then we have the district hospitals. We have 40-ish of them in the country so in each district we have a hospital, sometimes two.

Then, we have the health centers. We have [REDACTED] health centers in the country… in every sector so that’s administrative units. That means every sector has one health center. And then, we’re trying to get to the lower level to have health posts in every cell so it’s a lower level and then we have the villages. So if we have a health post in every cell, we know that the access will be better and people with healthcare closer to their homes.

With that, we also need to have the health professions that are trained for the level where they work in, so we have nurses in the health post but you also have a highly specialized physician [inaudible 00:20:15] type of care. That’s what we’re working on now is to try and get the [remaining? 00:20:20] resources that are qualified, especially for those very, very specialized settings.

Interviewer:	I see. And would you say that in your Ministry of Health that you have enough expertise in, like, evaluation and monitoring?

M6:	Yeah. We have been building that over the years. We do.

Interviewer:	Okay. Would you be able to give me a rough idea of what kind of resources it takes to run your evaluation and monitoring?

M6:	Resources in terms of money or in terms of people?

Interviewer:	Both.

M6:	Money, I’m not sure; I don’t know. One thing that I think is that we have an integrated supervision team that goes out in the field every quarter in addition to all the reporting that happens every month — to go and verify the data quality and so on so it’s usually a huge team of, like, 20 people who go and look at the administrative but also the clinical indicators and the financial status so it’s usually both teams.

Now that we have the electronic systems, we have reduced the physical check-in at the site. We do visit the site to be able to verify what has been reported is really accurate.

Interviewer:	Okay. No, that’s great. Okay. That was actually the last question and I just quickly wanted to revisit the question about if there’s anything that you haven’t taken up and whether you’ve thought of any examples, but otherwise we can leave it at that.

M6:	Yeah. I thought about asking you the question beforehand so I can prepare, but I’m not seeing anything that we haven’t scaled up or that we thought was not worth doing. I can’t think of anything.

Interviewer:	No, that’s perfectly fine. It sounds like you have a very successful time during your time working with the Ministry, so I think that’s a very good sign.

M6:	Thank you. We are hoping to continue like this.

Interviewer:	Yeah. So, M6, do you have any final comments that you’d like to make?

M6:	No. The thing is that the questions are very general. It would be helpful to have like more specific questions about a specific intervention, like I understand that you’re asking these questions to a lot of different people but it’s very, very general and we had a lot of different initiatives that scaled up. HIV was one, but we had the MCH programs as well, the vaccination type of thinking…

We’ve done the [REDACTED]vaccine. That’s another one that started early and it was very fast scale up to the whole country, so there’s a lot of initiatives that we could have talked about if we could look at it by sector, by type of... by field, I mean. That’s my only comment, but I don’t know the whole study and I don’t know the vision and maybe that’s perfectly fine with what you guys are trying to do.

Interviewer:	No, no, that’s great feedback. It’s always a very fine balance between trying to get enough detail but not taking up too much of your time… Thank you very much for sharing those stories with us and sharing those perspectives as representing the Ministry of Health on what kind of things are important and it’s very valuable having your perspective, so thank you very, very much for that.

M6:	Thank you. Thank you. I appreciate you guys asking us. Thank you.

Interviewer:	Yeah.

[end of recording: 00:24:20]
