# Supplementary material for: Supporting Better Evidence Generation and Use within Social Innovation in Health in Low- and Middle-Income Countries: A Qualitative Study
Source: PLoS One. 2017 Jan 26;12(1):e0170367. doi: 10.1371/journal.pone.0170367 (PMC5268497; doi:10.1371/journal.pone.0170367)
Supplement: S1 Dataset — (ZIP) [file pone.0170367.s002.zip › Data/Data - Interview transcripts/P10.doc]

P10:	Yes.

Interviewer:	Okay. Alright. So, P10, just for the record, can you tell us about what you do?

P10:	[REDACTED]

Interviewer:	Okay, so can you tell me a bit more background about the problem that you’re working on, particularly towards a lot about the HIV/AIDS issue and the transmission from mother-to-child?

P10:	Yes. What kind of aspects would you like to know more about?

Interviewer:	… I guess that’s actually why we’re asking this is that so we can try and determine whether outcomes that you define will align with the different things that you do as an organization, so if you can define the problem in terms of outcomes, that would be great.

P10:	If you can define the problem in terms of…?

Interviewer:	In terms of outcomes that need to be achieved to solve the problem.

P10:	Sure. So, I’ll start from the high level and then I’ll go further and at any point feel free to have me stop. The two ultimate goals or outcomes that we focus on achieving is (1) making sure an HIV-positive pregnant woman is healthy and thriving so making sure that she is (1) adhering to her HIV treatment so adherence is a key issue, but second is that she is retained in care and engaged in care along the WHO recommended antenatal visits but also some other key post issues that are critical for that mother. The second is that we are altering goals to make sure that the baby that she is carrying is born HIV-negative and if the baby is HIV-positive that that baby is *immediately* linked to care and treatment.

In addition, we looked at a whole host of issues such as the mother’s disclosure of her HIV status to hopefully a partner or to her family members which helps to ensure that she stays adherent to her treatment; making sure that she has support. We work on her self-esteem and self-efficacy of understanding how to present and how to talk about her HIV status, but also understanding how to take her medications, what she needs to do in that space.

We help her with breastfeeding, ensuring that she either exclusively breastfeeds or exclusively formula feeds but doesn’t do mixed feeding. We work on a host of other issues including family planning, immunization, tuberculosis and malaria and so on, so there’s a whole host of public health communicable as well as infectious disease issues that we focus on as well as a wellness and an overall health maintenance approach.

Interviewer:	Wow! That’s very extensive work that you do.

P10:	It’s wonderful. If you ever get a chance to see it in our countries, it tends to really impact people.

Interviewer:	Yeah, I can definitely see that. You’ve described a lot about the program-based outcomes and particularly the health-related outcomes. Are there other outcomes that you define in terms of, say, scale or other organizational/management type outcomes that you measure as well?

P10:	We definitely look at issues such as costs and scale so one of our… We are very cognizant of the issue of trying to provide an intervention and a set of interventions at a cost that is sustainable within local host countries.

We look at issues such as the salary of the [REDACTED], the employees, because one of our goals is government ownership and so we make sure that they are trained and supported as a cognitive that can be absorbed within systems at some point in time. We haven’t measured, per se, although evidently one of the countries that we are working in and speaking to look at some task shifting and some task augmentation outcomes.

Interviewer:	Alright. Go ahead. Hello? Sorry.

P10:	Yes. I think in terms of some… I think that’s kind of what’s coming to mind. I’m going to put my headphone just down for a second. I have to put some air conditioning on.

Interviewer:	Yeah, sure.

P10:	It’s quite hot somewhat.

Interviewer:	Okay, that’s fine.

P10:	It’s our winter, but global warming has really created some havoc in our temperatures… Okay. Those are kind of the ones that come to mind right now.

Interviewer:	Okay, okay. Alright. I just wanted to draw on one point that you brought up about the goal of having government ownership over what you do.

P10:	Yes.

Interviewer:	How do you influence government then to try and achieve this goal?

P10:	… A couple of ways. One is through a large kind of systemic policy and advocacy, making sure that there is both understanding and education around what a peer mentor model such as our organisation can do, the importance of psychosocial support and making sure that we are sharing results around our outcomes of retention, adherence, earlier presentation—those key issues that are important to both Department of Health, Ministry of Health, as well as international agencies.

The second is through a… very much a familiarity, a partnering approach. While most of our resources with the exception of... Most of our countries, our resources come from foreign donors, outside donors, outside of the local host country. In [REDACTED], we are very fortunate in that the country is working with us on a provincial level and providence by providence, providences are using their own revenue to buy into the program to support it and to help us to help them take it over.

Interviewer:	Wow! That’s great.

P10:	It is kind of a multi-problem approach.

Interviewer:	Okay. Other than government, are there any other main stakeholders that you aim to influence?

P10:	In addition to government agencies, we do work at also legislative bodies, so Parliament is definitely always a part of the group that we are trying to engage and support. I would say that international donors in country, especially multilaterals. (The UN agencies are key.) And then partners, various and other local implementing or international implementing partners, and the private sector as well.

Interviewer:	Okay, so now drawing upon what you said influencing the government and being able to show results at the large systemic level, how do you actually measure the progress of your work?

P10:	How do we measure?

Interviewer:	Yeah.

P10:	In terms of influencing the governments about our program? In terms of indicators, we don’t have a standard set of things that we use to measure that in the organization. These are activities that we tend to have I would say [a] more common sense approach to, but for us some of the key indicators that we would be having success are (1) that we are inviting [inaudible 00:09:45] to the table and stakeholders in areas that pertain to maternal child health, reproductive maternal-newborn child health spaces, and that tends to be both in terms of preparing policy, discussing policy, looking at strategy, so having that space at the table is a critical part of what we look at as success.

The second is (and which usually becomes before having a seat at the table) is government engagement in our actual service work.

Interviewer:	Okay.

P10:	We do conduct a lot of site visits and hands-on demonstration of our model. In almost every country, we have what we consider “model sites” where we do bring in outside and also inside targets into spaces to be able see hands-on and to experience the model and that tends to be a real important space for advocacy for us.

The third is making sure and seeing if whether the policies that we are trying to support and trying to get implemented. For example, in [REDACTED], we were able to help create a national [REDACTED] mentor of the program which is administrative guidelines that the Department of Health, the Ministry of Health adopted and use to create the national program that then we came in and provided technical assistance to help other implementers learn how to implement the program... and they influenced other donors to move their cohorts out of end-stage programs into implementing this model.

For us, it’s looking to make sure that the policies that either we support or our program models have uptake within the government and the [REDACTED] model is one of the highest nth degrees of national level support. [REDACTED] would be an example of national support without a national program, but then national mandates for providences to look at the issue, to determine if our model was something that they could or need to implement, and working with providences on a case-by-case situation.

Interviewer:	Okay. So going back to the actual programs, how do you measure the outcome of the program and how it’s running?

P10:	It’s really done on an individual level.

Interviewer:	Okay.

P10:	We do data collection across a host of indicators that roll up to some key objectives around the model, around key issues such as encouraging health-seeking behaviors, providing access to quality services, changing norms, creating supportive environments, and then within that we have very specific indicators, and this is collected on a client-by-client basis.

Right now, we’re in a transition phase of moving from a paper-based to an electronic data collection system, and we also do a site-level quality assurance process that each site looks at their client data and they review and they look at various trends on a monthly and quarterly basis. They share that information with cross-facility staff that are brought in to look at the data, to be able to understand it, and to be able to provide feedback. Then on a quarterly basis, sites share cross-data so a site will look at three months of data from their nearest site and be able to both critique and ask questions as to why their retention numbers are so high or so low, what kinds of things are they doing that might be different, are they trying some innovative things that made their data so strong, and we share that kind of cross-learning.

Interviewer:	Wow! Okay, that’s great. That’s great. So what are the main methods of collecting that data?

P10:	The main method is a client logbook that is kept by the mentor mother.

Interviewer:	Yeah, I see. Okay, so it sounds like… so each person’s kind of responsible for their own data collection and then you have a system where it all is collected together and shared with everyone in the organization.

P10:	Yes.

Interviewer:	Okay. Great. Okay.

P10:	And where possible, we do cooperate with information like CD4 count, tests, asking clients to bring their lab data and things like that, but we then also upload them to the system.

Interviewer:	Okay. Wow! Okay, so it’s a comprehensive data collection system that you’ve got.

P10:	Yes.

Interviewer:	Okay. For your evaluation and monitoring activities, like what you’ve discussed just then, what kind of resources does it take to make it possible?

P10:	… One, it takes *tremendous* labor hours—the system that we have in place—because it’s paper-based. It’s extremely laborious. So in addition to the [REDACTED] who implement data, there are site coordinators who review and do an initial quality review for completeness. Those data then get pulled into our database here at head office. We have a team of… It’s probably 3 individuals plus then waves of consultants that will do extraction, review, aggregation, review, quality assurance. It’s an extensive, extensive system.

Interviewer:	Yeah. Okay.

P10:	And right now we’re in the process (as I mentioned) of moving into electronic, into a mobile health process of both the platform so creating an electronic client diary. So, we’re both building the framework, building the applications, and with outside vendors making sure that it’s… It’s kind of off-the shelf technology but then customizing it. But again, it’s another large, large expense for the organization.

Interviewer:	Yeah, yeah, I see. But hopefully, it’ll make things a lot easier and streamlined.

P10:	We hope so. We do hope so.

Interviewer:	Okay, just one very specific question about randomized control trials. How do you feel about randomized trials and would you conduct one?

P10:	My experience in having overseen several and been part of them, I think they’re gold standards and I think for better or worse they are the gold seal of approval that helps to drive donors to support you or to try to create a platform of efficacy that gives you credibility.

That said, my experience has been that they’re expensive, that oftentimes organizations jump into them too early and ask some of the wrong questions, and often by the time you end up getting a viable set of things that you are actually testing and observing, you’re not really looking at the full intervention or the core intervention and if it changes during that time it’s a tough situation.

Interviewer:	Okay.

P10:	We have never conducted one, to date. We have just finished our first two fully external evaluations and we are in the process. We’re waiting for hierarchy approval from [REDACTED]. [REDACTED] is funding [REDACTED] to conduct a quasi- RCT and I say quasi because they are… I think that they’re cutting some corners on time and also on the comparison group issues, but they’re doing a randomized control trial of our program, our flagship core program that I’ve explained to you but also another program which is an early childhood development piece in [REDACTED]

Interviewer:	Wow! Wow. That sounds quite exciting. So P10, moving onto some of the challenges that you face in conducting evaluation so it doesn’t...

*[audio break/move to next audio]*

Interviewer:	Alright, so we’ll just start from where we left off. I just wanted to ask you about some of the challenges you face in conducting evaluation monitoring.

*[audio break/move to next audio]*

Interviewer:	P10, I just wanted to ask you about some of the challenges you face in conducting evaluation monitoring.

P10:	Okay. In general or is there any specific areas you’d like to focus on?

Interviewer:	No, in general.

P10:	I think I would break it down to a couple of key areas. The first would definitely be around human capacity, expertise, and skill; ensuring that we have individuals that have the capacity to actually conduct robust evaluation is critical. I think the second is around individuals with the right skillsets around qualitative and quantitative evaluation and also individuals that can conceptualize outcome and impact studies.

Interviewer:	Yeah.

P10:	Those are some initial key issues. I think the second broad bracket of issues that we face is the non-standardization of evaluation frameworks for key indicators in areas that donors support, so we were often having to hybridize what one donor wants in order to make that we’re proving what another donor wants and yet a third donor wants something completely different and so that tends to create a lot of both confusion and difficulty and although we try as best as we can to say, “We are collecting this data across this menu of indicators and these are the kind of outputs and outcomes and impacts that we report on,” folks really always want to have something that’s just a little bit different.

I think the other piece is a lack of any kind of uniformity in terms of Ministry of Health or Department of Health in terms of the systems that they use to either upload or to house, to aggregate data, and for us it’s a very, very difficult process as a smaller organization to be able to work and come up with something that’s customized for every single context in which you work.

Interviewer:	Okay. Just wanted to draw on your second point about demand status indicators and frameworks and how different funders want different information. As an organization, how do you try and come to terms with that?

P10:	I think early on the organization was trying to appease each donor. And so, you could see that for five donors we had very, very different, laborious sets of data and information that we would collect and share. We would try to be much more… judicious but also more standardized in our approach to be able to educate donors on what we were using, trying to standardize what we collect based on international standards where they exist, and trying to make sure that we were collecting data or at least what I would call “enhanced data” so data that might be a bit either richer or just a bit different than some national number or national dataset that we can use as a comparison.

Interviewer:	P10, you just cut off there.

P10:	Oops! Hello?

Interviewer:	Hello? Sorry. I caught “enriched data” and then it just cut off. Could you just repeat that?

P10:	Okay. Sure. Instead of enriched, it’s *enhanced* data. We try to collect data that might be just a bit different or a bit richer than what a country would collect so that we can use the country data as a comparison.

Interviewer:	Okay. You’ve identified quite a few big challenges in being able to conduct quality evaluation monitoring. In a perfect world, what would help you do this kind of work better?

P10:	In a perfect world, individuals that are trained in M&E would have a 60-ish percentage standard set of tools and approaches that they’d have in their toolkit or in their tool belt, that they would come to the table with that would be one piece.

The second would be that donors have a more harmonized set of indicators and would understand the difference between inputs and outputs and outcomes and things like that and would be able to come to the table with a more harmonized set of things that they wanted to collect information.

I think the third would be a bit more sharing of methodologies but also approaches of evaluation so that organizations could have more things that would be readily off the shelf to use as tools to actually implement evaluation.

Interviewer:	I see. Okay, okay. Can you describe some of the tools that you’re talking about?

P10:	Sure. For example, we moved into the space of gender-based violence and we are trying to collect information from clients as to the nature of the violence, being able to describe the context, to be able to come up with a scale and rate, and there are hundreds and hundreds and hundreds of these scales out there and very few have been either calibrated or have been referenced for the space in which we work and even more so the issue of language and translation and cultural fit and sensitivity in terms of language level has been really, really difficult.

So in that very, very just basic space, just even the tools of scales and collection instruments, having more open-source or spaces where these things are shared or point us to organizations that have such tools would be really, really helpful.

I think the second piece that we are really, really banging our heads against is around this mobile health platform, the data collection framework and the databases and how these things are gonna work together has been also extremely frustrating. We were very lucky early on that we had a public-private partnership with [REDACTED] and they helped us create our data warehouse and that has served as an organization for about [REDACTED] years.

It’s now to the point where the technology is a bit *too* updated and those that are now servicing because [REDACTED] doesn’t have service technology are just too costly for us to go on so we need to transition to something else and as we’re looking as this, we’re realizing (a) that we got really, really lucky that we had a partnership with [REDACTED] because they kicked in so much support that we never could have afforded, but also (b) we’re a little bit nervous that we’re gonna end up in a situation again in which we end up picking the wrong horse, that we’ll pick something that is gonna become obsolete in another 5 or 6 years, and then we’re stuck.

So I think that kind of space also in terms of if you’re an ‘X’ kind of organization doing these kinds of things, here are the kinds of tools that you could or should be using or these are the things you should be talking to vendors about before you make a decision. I also think that while there are, in the United States for example, there’s the… Gosh! It’s called the... I’m going to get the acronym or the name wrong, but it’s the Association of Evaluation folks. (I can’t remember what it’s called.) It’s the National Association or the association… and it’s basically a hands-on implementer. There’s a hands-on implementers working group in that entity where folks can talk about what they’re working on. They can get some mentoring, some coaching, some collegial support, peer engagement. We need those kinds of activities in the country level to be fostered by universities.

Interviewer:	Okay.

P10:	We’re lucky here in [REDACTED] the University of [REDACTED], they’re a bit more engaged and wanting to do that kind of thing. In many of the countries in which we work, there just isn’t any kind of an academic space which then I think is a bit of the ‘chicken and the egg’ as to the reason why we have such a hard time finding qualified candidates or candidates that are trained in a way that they have intellectual curiosity but also the skillset to be able to parse and dice a question and figure out different ways of how you might collect information or how you might use data to answer that.

Interviewer:	Right, I see. Okay. Wow! They’re all very interesting inserts and I hope that one day we’ll be able to try and solve those issues for you and create this perfect world where you can collect all this data...

P10:	I think that would be a perfect world and I hope one day we do have...

Interviewer:	One day. One day. So we’re coming up on the end of the interview now. Just wanted to ask one more question about programs and harm. Now for a lot of organizations, despite best intentions, there can be some harmful outcomes. Have you thought about ways in which your program could be harmful?

P10:	Most definitely… I’ll give you maybe a couple of examples and how we’ve [inaudible 00:10:30] a little so what we’ve done to be able to protect.

Interviewer:	Okay.

P10:	Just the very fact that our key frontline healthcare workers are HIV-positive women who recently have given birth, have gone through PMTCT, and because of their successful adherence to treatment, they’ve either been identified or they’ve come forward as candidates in the program to be trained. For many of them, this is their very first official employment and while we do everything we can to train them, to prepare them… We even help them understand this is going to be the first time they get paid so understanding how to have a bank account or how to have discussions about finances in the household. Oftentimes, the very nature of that—our employee is working and is getting paid— creates a potential problem for domestic or int…

Interviewer:	Sorry, P10. You just cut off there.

P10:	…this happening initially and we didn’t realize this was happening and…

Interviewer:	Sorry, P10. You just cut off a bit there.

P10:	Yes?

Interviewer:	I think you were talking about causes, the fact that employees are being paid creates problems, domestic and internationally, and then you just kind of cut off there. Hello?

P10:	Okay.

Interviewer:	Could you just repeat that line?

P10:	[inaudible 00:11:54] me again?

Interviewer:	Yep.

P10:	Can you hear me now?

Interviewer:	Yep.

P10:	Hello?

Interviewer:	Yes? Yes? Hello, P10?

P10:	Hello?

Interviewer:	Hello? Can you hear me?

P10:	Yeah, I think we’re breaking up. Can you… Yes, hello? Hello? I can hear you.

Interviewer:	Yep. That sounds good. Yes. Hello? Okay, it’s just cut off now. Hello, P10? Hello?

*[re-dials]*

Interviewer:	Hello?

P10:	Hello?

Interviewer:	Hello, P10? Okay.

P10:	Hello?

Interviewer:	I think we’re connected again. Hello? Can you hear me? Hello? Hello? Hello? Hello? Hello, P10?

*[re-dials]*

*[long pause]*

*[voicemail]*

P10:	“Hello. I’m not able to answer your call right now, but please leave your name, the time you called...”

*[hangs up]*

*[audio break/move to next audio]*

P10:	Yeah, just the nature of paying the [REDACTED] can create a situation in which they’re at risk for gender-based violence and we had not anticipated and what we were able to do and do very quickly is we have a supportive supervision system in place which former [REDACTED] serve as site coordinators and supervisor the [REDACTED] and they have weekly check-ins and there’s various questions we ask them about changes in their self-esteem and their self-efficacy and one of the key issues that was starting to come up was tension with their male partners around payment and so it lead us to (1) create an entirely new module in our training where we prepped them and prepared them for that, but also opened up a opportunity for new [REDACTED] to be able to invite their partners into an open discussion with their supervisor around what their role is, why it’s important, the fact that they will be paid, the fact that we will pay the mentor mother to a bank account, not giving cash to a husband, and it ended up kind of moving that process and changing it, but it was pretty scary initially.

Interviewer:	Wow! Yeah, it must’ve been quite scary, but it sounds like you found quite a good solution to it, although...

P10:	Yes. I think we were lucky that we had something in place that was able to pick it up and able to give us some trend information so we could react to it pretty quickly.

Interviewer:	Yeah. Wow, that’s great. Did you want to talk about any more examples about how you’ve thought about harm in your program?

P10:	About harm in our program… I’m just trying to think. I’m racking my brain thinking about harm and I’m not... We haven’t picked up things that we’ve thought, “Okay, this is something that we’ve either been afraid of” or was something that we have to expect it. While we’ve made lots of changes through the time around the intervention, nothing really jumps out as something that I would say would kind of fall in that space about harm.

Interviewer:	No, that’s fine. So, P10, that actually brings to the end of the interview. Do you have anything else you wanted to add?

P10:	No. Just was happy to be engaged with you all.

Interviewer:	It’s been an absolute pleasure talking to you. I’ve learned a lot about what you do and it sounds like absolutely amazing work and very successful, so congratulations on what you’ve achieved and thank you so much for speaking with us.

P10:	Thank you. And, Interviewer, I’m not sure if it would be helpful to you, but in terms of the research that you’re doing and how you’re moving forward but we are just ready to… I think within the next few weeks, we’ll be releasing our big annual evaluation and I’m happy to send you a copy so that you can actually get an example.

Interviewer:	Yeah. Yeah, that’ll be great. That’ll be perfect for what we’re looking at.

P10:	Okay.

Interviewer:	The other thing that you could potentially help with as well is we’re trying to look at the alignment between Ministry of Health funders as well as health initiatives, so if you have any contacts for Ministry of the Health or funders who you’ve been in contact with, it would be great if you could put us in contact with them so that we get a full picture of the different outcomes that each of the organizations or bodies are looking at and seeing where they align and where they don’t.

P10:	Okay. I will ask my assistant to reach out to our program folks and see if we can get you some contacts.

Interviewer:	Yep, that’ll be great. But otherwise, we’ll keep in touch and once we have all the findings collected, we’ll be in touch with the results.

P10:	Okay, good. Well, good luck. All the best to you.

Interviewer:	Yeah. Thank you very much, P10. I hope you enjoy the rest of your day. Bye.

P10:	Okay, bye-bye.

Interviewer:	Bye.

[end of recording]

[total time: 00:38:51]
