# Supplementary material for: Supporting Better Evidence Generation and Use within Social Innovation in Health in Low- and Middle-Income Countries: A Qualitative Study
Source: PLoS One. 2017 Jan 26;12(1):e0170367. doi: 10.1371/journal.pone.0170367 (PMC5268497; doi:10.1371/journal.pone.0170367)
Supplement: S1 Dataset — (ZIP) [file pone.0170367.s002.zip › Data/Data - Interview transcripts/P11.doc]

Interviewer:	A lot of the idea was born out of what happened on that day, so we’re very excited about it. Alright. First question. I just wanted to hear from both of you. Can you tell us a little bit about what you do with your organisation?

P11:	You mean just individually as staff members or just what our organisation does?

Interviewer:	Both.

P11:	[REDACTED]

Interviewer:	Okay, beautiful! Gosh. Thank you so much for such a beautiful summary, very succinct and very nice, and it’s amazing what you guys are achieving and the next steps from here out. Sound absolutely incredible.

P11:	Thank you.

Interviewer:	Yeah, it’s really important work that you’re doing [REDACTED]. I just wanted to explore a bit more to do with evaluation. How does your organisation specifically define success?

P11:	Success for us is saving lives and reducing mortality amongst the largest burdens of disease for people living more than 5 kilometers from the nearest clinic on average, which is by definition the most remote districts. We look at a few different indicators. In our early demonstration work, we looked at access to care for fever-related illnesses amongst children so malaria and pneumonia, diarrhea, and looked at coverage ‘cause one stat so the number there was [REDACTED] % when we started, was the proportion of children under 5 who had actually sought care or treatment from a formal healthcare provider two weeks prior to the sample being taken, so that’s a very low number. It correlates to a very high under 5 child mortality [rate] but it also correlates to an average age of death for the population sample of [REDACTED] years. Average walk was [REDACTED] hours from the nearest clinic up to [REDACTED] hours distance to the nearest clinic.

So, that’s just to give you a bit of the baseline. That number tracked up to [REDACTED] % about [REDACTED] years later so this was between [REDACTED] and [REDACTED]. Actually, another person took the first sample… and survey. She started that program up. Then, the other one we look at as a proxy for maternal mortality is facility-based deliveries.

Interviewer:	Okay.

P11:	And actually the whole maternal cascade: prenatal care, facility-based delivery and postnatal care. We’ve looked at the data more on facility-based deliveries recently. Probably important to note that that number was also around... The total maternal cascade was [REDACTED] %—mothers who had received the entire cascade. The facility-based delivery rose to [REDACTED] % prior to the Ebola crisis. During Ebola around the country, it fell [REDACTED] % from baselines of [REDACTED] down to [REDACTED] % so they were already bad then they got worse. In an are where we had been working, it only dropped [REDACTED] %. During the height of Ebola, [REDACTED] % and now it’s up at [REDACTED] %, the data from the latest quarterly report.

Interviewer:	Wow.

P11:	So those are two that we look at for coverage and the other things we’re tracking is performance of... I’d call those output indicators—the ones I just mentioned— which are mainly born and driven by the government of our country. These are indicators they look at in sort of interval periods between the DHs that are done.

And then in terms of inputs, we’re looking at the performance of community health workers themselves so we have a performance scorecard we’re experimenting with looking at do they actually do the work, have they diagnosed patients appropriately, are they actually on time and at work, and we tie that to their performance. So far, it just means whether they continue to be employed or not.

Lastly at outcome level, what we’re going to be tracking and the numbers are gonna come in this national rollout… We’re doing a prospective controlled step-wedge analysis in the first county where this national program is being scaled up at the moment and that will tell us some prospective cohort-like data on outcomes which we’ll look at under 5 child mortality [rate], look at the same maternal cascade. Of course, it’s hard to get maternal mortality data; you have to have very, very large samples.

And then on a national program, we’re actually right in the middle of designing what the impact evaluation will look like on a national scale, so we have a number of actors like [REDACTED] and this will be some kind of... our organisation has been encharged as the lead NGO developing the national program serving as the lead group for the Ministry of Health, so we’ll have both purview over this and help write the design (if not write it) with the Ministry and a number of other partners looking at the national scale up and that will look at impact indicators as well as process indicators, looking at how do you scale a program like this, what’s the relationship between county and regional, sub-national and national… How do you empower the national group? What’s the right staffing ratios? How do you take a supervision cadre—these nurse coaches, for instance—and scale up a new cadre into an existing system? What’s the cost of all of this? We have a projected cost of $[REDACTED] per capita at scale, but is that really gonna be the real cost, et cetera. These are gonna be the questions that we look at, so some operational research.

Interviewer:	Wow! Okay. So before you got involved in this amazing national program collaboration, do you guys start collecting much data on the scale and replicability of the program?

P11:	Which kind of metrics do you mean?

Interviewer:	Like more systems-based, organizational-based metrics to see whether a certain model that you’ve tested out could be replicated into another community?

P11:	Yeah. We had done that work actually really early… [REDACTED] and [REDACTED]. We had actually started as an HIV community health work organization and helped scale that from one site to [REDACTED] across the country and there was a lot of anecdotal learning from that that informed how we developed a more robust top ten conditions—women’s and child health at large— in a more remote area program, this primary care version, in the last [REDACTED] years.

The goal has always been national scale so we set up the program with the government and the Ministry of Health in [REDACTED] asking three questions which were scale-oriented: “How do you best supervise these community health workers?” It was all about how do you improve their performance in the scalability of a program within the public sector, but the three major questions the government asked were “how do you improve the supply chain,” “how do you improve supervision,” and “what’s the right set of incentives for these community health workers? Is it pay? Is it no pay? Is it performance-based pay? Is it just something different?” So those are the questions we went into the pilot with as key top questions from the governments. We asked them, “What are your top [REDACTED] scale-related policy questions?” and those were the three they came up with and then we wrapped a lot of our own questions around it that we knew were important for operational management.

Interviewer:	Wow, beautiful! Okay. You’ve talked a bit already about how you worked closely with the governments and trying to design your research questions around the research questions that they were interested in. Are there any other stakeholders who you try to influence and how?

P11:	That is the major one because we find them to be the major driver of scale and our goal is to scale via the public sector. Of course, we have at the table—because they’re either important technical implementers or they are financiers—we have [REDACTED]. The World Bank has been looking at this, all informing our evaluation as well, and we deliberately try to make that happen because we would like them to be influenced.

The short-term goal is to have this primarily aid funded by bi-laterals and multilaterals and domestic resources from the government of our country and philanthropy, which is why we’re involved in the [REDACTED] for instance. The sort of medium-term phase, the end-term phase, is for government to take on the majority line share of the costs and so in the interim between a three year period and a seven year period when the program will be fully scaled and government will have major financial ownership besides having clear authority over the program. In that four year period, we would live to see innovative forms of financing as well as continued sustained traditional bases of financing [REDACTED] et cetera for this program.

And so for them, it’s important to have them as key members of the evaluation in the first three years, so that’s kind of the intention is the first two to three years everyone’s looking at this national scale up as phase one and there’s going to be a robust evaluation done in concert with these actors at the end of phase one and that will help inform how the program is funded in phase two and what further questions are asked after that.

Interviewer:	Okay, so it sounds like you’ve got quite a lot of support from both kind of the policy-making side as well as the public sector side. What about private enterprise? Do you work much with them?

P11:	We have done work with private enterprise in terms of helping with the infrastructure and then in terms of supplies, so there are a few private entities that have given their product in kind with the intention that they might be able to just sort of create a shared value approach where they introduce their product to the market through this initiative then they might be able to negotiate low-margin, high-volume deals with the government. One of them is a [REDACTED] which does non-invasive hemoglobin monitoring and that’s one type of set-up.

Because we work in setting where there are no roads, no cell phones, no banks, on and on no water, but basically… “Remote is different than rural” is our main thing that we say ‘cause we’re again, as I said, very concentrated in trying to develop a model and for that truly physical last mile. We’ve had to bring in crowd and private enterprise when it’s relevant. For instance, [REDACTED] can tell you this story ‘cause she showed up when there were no telecommunications period in the district in an area so she was cut off as everybody else there was from everybody else. The only way to communicate was via paper… and satellite. We put up a satellite internet at our hub office.

Interviewer:	Wow.

P11:	So you can imagine very hard to do M-Health. With that set-up, you can’t do it, so we negotiated with leading telecom in the country, and persuaded them to consider opening a cell tower in this market, which for them was the least... It didn’t meet any of their criteria for the most likely market so they set up a deal structure with us where we prepaid them two years of service in advance. In essence, it was like a company pre-paying for service, say [REDACTED] coming to a small, rural town in North Carolina because Research Triangle Park has all the companies there.

So, we pre-paid them and that incentivized them to open up a tower there so then that brought mobile to a large portion of the population in the district in an area and allowed us to do a version of M-Health where we have sort of on-network and off-network. I think it’s one of the first on and off network, hybrid M-Health for community health working programs in the world is what our M-Health tells me. 70% are still off-grid, so we use Bluetooth with supervisors and phones on one part of the grid and then that interacts with regular M-Health in the other part.

But that’s how we’ve engaged private sector. It’s been primarily as-needed. It’s not part of our business model, per se, besides the supplies piece… It’s not like [REDACTED] where they have a fee-for-service model as part of it by selling products in a certain area. For us, it’s just been that we don’t have any ideology about any of it. We just think we should do whatever the evidence shows and what we’ve noticed from the evidence is that really remote areas, fee for any kind of service, even implementing a non-free service, is quite challenging ‘cause there are already a lot of barriers including transportation that already add costs to a care system that by government policy is free already. There’s already costs incurred out-of-pocket, about [REDACTED] % just by transportation, according to one survey.

And then the other main reason is ‘cause we just think that the best path to scale the program, at least in our country, is via government and we’re well-positioned (as you can see) to do that.

Interviewer:	Yeah, beautiful. Wow! It definitely sounds like there’s a lot of quite unique challenges working in remote areas.

P11:	Yeah. There are a billion people in these areas and if you look at the whole march towards, ‘cause I know a foundation very interested in inflection points and causes as well as organizations and just in issues in particular countries. The big inflection moment right now is these SDGs in 2030 and one of the most important SDGs is the health one that’s focused on universal health coverage.

Recent report from The World Bank of the WHO shows that they’re at least half a billion to a billion people who have no access to even basic health services. Most of those people live in these remote areas and the challenge is that they also don’t have the health workers. [REDACTED]

If you look at where… while a lot has been made [REDACTED] in terms of all the progress in global health, we reduced child mortality from 10-12 million 25 years ago down to 6 million a year. The goal is to get that down to half that or even less. Gates’ idea is can the world save 61 more million children’s lives by 2030. The delta between that, where we are now, and where we will be then, a lot of it will be influenced and shaped by what happens in these final frontier communities where the mortality rates are still as high as they were 50 years ago and the operational and systems-related challenges are not just… artifactual or they’re not just trivial. They truly do need to be different types of models of delivery that build on rural and urban areas but really are focused on remote areas.

Just one quick example. We work in a community. We just launched in a new district. We work in [REDACTED] districts now with the Ministry of Health and [REDACTED] of them [REDACTED] hours from the nearest clinic and there’s [REDACTED] people who live there.

Interviewer:	Gee! That’s definitely a big challenge to try to overcome. [REDACTED] hours! That’s quite phenomenal. Gee. Gosh.

P11:	If you look at [REDACTED] — all of those communities, those remote areas, have a very different delivery set-up than what is in, say, rural or urban areas where clinic proximity and other kind of proximity’s closer.

Interviewer:	I see. Okay.

P11:	Yeah.

Interviewer:	It sounds like despite the challenges that you guys are facing with trying to deliver quality healthcare services to remote areas, you’re still managing to balance that out quite well with your evaluation and monitoring activities. I’m just wondering a bit more about what kind of methods do you use to go about collecting your data?

P11:	Well, in the beginning, we do these baseline cross-sectional randomized cluster-based sampling for the district we come in to. And that’s a baseline. Then, we are now using a new method to look at… In the very beginning and we thought this was the right operational and strategic move who turned out… ‘Cause our goal was just to demonstrate early results and do it seamlessly, we didn’t try to do an RCT. That wasn’t what the burden of proof was ‘cause the government directs that for us. What they wanted to see was “is this feasible and can you get good outputs?”

And so, we did a time series cross-sectional survey again, which we’re informed that second set of data that I gave you from an area, so again that was just cross-sectional repeat time series in the same district. It’s a little easier for us too because there’s not any other actors in these places. It truly is just us ‘cause that’s why they’re remote in the first place so there’s not a lot of spillover effects or noise in our data.

So, that was stage one. In this next phase of scale up with the government, the national scale, we feel like we could do an RCT right now. We might—we’re considering it—but we may just stick with the next [REDACTED] years given that it’s a [REDACTED] -year timeframe for another decision point for public sector scale. That’s the time point, the next [REDACTED] years, where they’ll decide what level of follow-on funding will come in to the program. We’re looking at a prospective cohort type of analysis —the one I just described a step-wedge where we…

Interviewer:	Yeah.

P11:	In the new counties we’re scaling up to, we focus the intervention in [REDACTED] districts of [REDACTED] and then we are taking baseline data in all [REDACTED] of the districts and doing repeated sampling every 6 months in all of the control and the intervention districts, and then once we get early data, then we roll over the program to the second half of the districts.

That’s probably what we’ll do. At least that’s the leaning. We haven’t yet decided. Of course that’s in negotiation with the government and then we’ll hopefully build that into a full-fledged RTC as the government and others take interest in that kind of an idea, ‘cause we’d love to be able to... I think that becomes important in our head when we’re trying to shape and influence global policy in programming but also looking into future countries, how do you show full proof that this worked and will be relevant in a new country.

Interviewer:	Okay. Okay.

P11:	Of course there was never an RTC done of the Health Extension Workers program in Ethiopia as far as I know as like a full-out thing so I don’t even know if that’s truly necessary in the public sector, but it probably is more from just the efficacy standpoint for an organization to determine whether it’s having the true impact it wants to.

Interviewer:	Yeah. So what kind of factors are influencing your decision of whether or not you do run an RCT?

P11:	Number one is does it change a decision and decision being does it change a policy, does it change the way investments run into a program? In Our country again because our scale partners, the government, that’s the main sort of actor along with its proxies like [REDACTED]

The second one is just does it tell you anything more than an alternative study would and is the difference in what it tells you worth the cost, the extra cost of doing the RCT.

Interviewer:	Yeah. Okay, so following on from that, with your current evaluation activities, what kind of resources are you using? I’m talking things like your data collection methods and data storage, staff number, and things like that .

P11:	Say again?

Interviewer:	What kind of resources does it take for you to run your current evaluation activities?

P11:	Sure. … If you’re just looking at pure evaluation staff, the total staff size for our organisation is almost [REDACTED]

Interviewer:	Okay.

P11:	I think we’re at [REDACTED] and that’s not including the community health professionals. The total number of staff for evaluation… We’ll get you the exact number. I believe it’s around [REDACTED]

Interviewer:	Okay.

P11:	At least [REDACTED] of those are based in the field, in Our country out in the field. We do our research collaboratively with [REDACTED] and so we leverage their data analytics and their staffing as well as their experience so we do our IRBs. I think we have [REDACTED] of them active out of [REDACTED]. We leverage them so we either have them contribute in kind, ‘cause some of us have (like I do) faculty status there or as we’ve done recently we just got a research grant from [REDACTED]. A small one, but to fund a stand-alone research assistant that will recruit from [REDACTED] and in essence succumb them to our organisation to carry out this research or help it.

Interviewer:	Wow! That’s going to be really useful.

P11:	Yeah, yeah, yeah, yeah, yeah. It’ll just add in some of the extra bandwidth we need so it’s kind of… There’s both resources within the entity and then resources within our very close strategic partner at [REDACTED] that helps us. We kind of leverage that, of course.

Interviewer:	No, that’s quite nice. It sounds like you’ve got a real nice system set up to be able to conduct your evaluations quite smoothly. I’m just wondering what are the…

P11:	“Smoothly” will be interesting to see... I think one of the most complicated things in this public sector scale up will be how do you collect data within the public system? They have a community health management information system, for instance. And we know that it’s flawed, it’s insufficient, and so one of the things we’re doing is that we’re working with them to reform that system so that we can collect at least the monitoring data through that.

And then, making sure that data has the fidelity our initial data had, making sure that the process—the bottlenecks that come up, the delays to getting data, the delays to analyzing it, the consistency with the other data points we have from population-based surveys and then the usage of that data to actually change program and policy at national level, I think that’s going to be much more challenging. Not just ‘cause it’s higher volume, larger geography, more people, but just because it’s working within another institution—the government—which again has weak systems but fortunately has a lot of interest and experience reforming them.

Interviewer:	Yeah, I see. Okay, so it sounds like there are various challenges to be able to conduct the research side of things. What do you think is the biggest problem that you face and what kind of solutions have you come up with to try and overcome that?

P11:	From a research standpoint?

Interviewer:	Yeah. Well, research/evaluation standpoint.

P11:	Yeah. The biggest problem… That’s one challenge is how do you do this, ‘cause it’d be a lot easier if we were just not trying to scale through the government, just had our own data system that we just needed to grow it bigger.

Interviewer:	Okay, yeah.

P11:	I think the second one, just the logistical challenge of collecting data in remote areas… how many research and evaluation directors to get just a baseline sample have to ride, on any one week, ride on a motorbike through the jungle, put the motorbike into a wooden canoe, paddle across to the other side and either go on a motorbike or walk by foot for several hours just to spin a bottle in a village and collet your [REDACTED] people that you’re going to survey to get your data point from that one village? And that’s a director. So, that’s a challenge.

Interviewer:	Well, it’s a huge challenge. Okay, so if that wasn’t a challenge, which I mean it’s a pretty difficult scenario to imagine, particularly in the work that you do, what is the other main challenge that you would face being able to conduct evaluations?

P11:	… I think hiring’s another challenge, just hiring the right help, the field level for a particular stage of growth. The people that help you get bikes and canoes and go do surveys in the jungle are very different than the types of people you need to do an RCT. [REDACTED] Do you get a part-time person? Do you get a technical assist.? Do you get a third-party or do you build that expertise in-house by hiring those people? I think those are challenges from a hiring standpoint.

Interviewer:	Okay, okay. Yeah. I think that’s a common challenge that a lot of organizations face—how to choose the right people for the right job and it’s an ongoing issue for a lot of organizations.

P11:	Yeah? You’re hearing other people say it?

Interviewer:	Yeah… So we’re almost done, just two more questions.

P11:	Sure.

Interviewer:	Penultimate question. In a perfect world, what would help you do your work better?

P11:	You mean in research and evaluation, right?

Interviewer:	Well, particularly in research and evaluation, but in any other way as well.

P11:	To be honest, when an organization reaches a stage like this, having unrestricted mezzanine-style funding from philanthropic actors is really critical ‘cause even in terms of research evaluation but even more broadly operationally, we will get some public sector actors to fund this program but it’s gonna be quite restricted and there’s gonna be a lot of lost opportunity if there’s not coupled with unrestricted funding that can actually be used to do a second order evaluation or actually do an RCT if that’s what needed rather than what the Bank or Ministry of Health is willing to fund, which might be a lot less than that because we know it’s critical.

To me, that’s one critical thing and I think the second piece is trying to tie that investment to performance-based metrics and to outcomes. When we go to scale nationally, there’s gonna be a lot of other actors involved. We want levers to help the government ensure those partners are accountable to the actual national model, which is in essence our model, and funding’s a great way to create that lever. There are all sorts of other levers: government regulation, accountability, accreditation, certification, quality control, et cetera, but having their outcomes tied to funding would be another great thing.

Interviewer:	Okay, I see. Okay. Kind of related to the whole evaluation and monitoring and also being able to tie in external stakeholders, have you thought about ways in which your program could be harmful?

P11:	How it could be harmful?

Interviewer:	Yeah.

P11:	Yes, I think some of the ways that we thought about (1) is that without a true outcome evaluation… One example is when you go to cost the program at national scale. The costing looks different than it did when you first ran the program. If we are wrong in that costing or we pulled out the wrong pieces of the model to make it more cost-effective or efficient, we could for the next 2 ½ years have a program going in all these sites that’s just gonna be ineffective. It’s not even going to get to launch really, ‘cause it’s missing fundamental pieces, and that’ll just waste a lot of time for a bunch of people that could’ve gotten care. (The challenge of course in remote areas is they’re absolutely getting no care.) It’s just that time would be wasted in terms of planning different kinds of care.

That’s one. Other ways that it could be harmful? I think if people think that this is a substitute—community health professionals—for even more advanced levels of care or that this is an isolation from the broader primary health system which the premise of our model is it’s not. I called facility-based deliveries as a key data point in our impact evaluations because facilities are involved in our model. It’s just that these guys are [inaudible 00:38:09]. If people think these are standalone community health professionals not attached to anything, that are substitutes for more spending or healthcare system, I think that’s a real danger as well.

Interviewer:	Okay. Has that been a big issue for you so far?

P11:	No, but I mean it hasn’t yet come to pass. I think the next stage will tell us whether, but I think at an assumption level, you can tell implicitly those assumptions are being made, but we haven’t politically tried to highlight a lot of that yet because we don’t think... Right now, it’s just a matter of getting something out there to start and we believe that this will create all kinds of demands on the healthcare system that will force further reforms up the chain, ‘cause right now they’re not even counted. They’re not even visible. The Census is off by, like, [REDACTED] %.

[end of recording: 00:39:52]
