# Supplementary material for: Supporting Better Evidence Generation and Use within Social Innovation in Health in Low- and Middle-Income Countries: A Qualitative Study
Source: PLoS One. 2017 Jan 26;12(1):e0170367. doi: 10.1371/journal.pone.0170367 (PMC5268497; doi:10.1371/journal.pone.0170367)
Supplement: S1 Dataset — (ZIP) [file pone.0170367.s002.zip › Data/Data - Interview transcripts/P12.doc]

Interviewer:	Okay, so I just wanted to hear from you, P12. Can you tell us a bit about what your organisation does?

P12:	We deliver services to the rural poor through a social franchising network.

Interviewer:	Okay. Can you tell me a bit more about how you do that?

P12:	[REDACTED]

Interviewer:	Okay.

P12:	We train them to deliver some type of service, so what we really do is what we call “a fractional franchise.” They already have their own business and we may approach them, for example, in [REDACTED] to deliver IUD insertions *if* they’re qualified.

Interviewer:	Okay.

P12:	And if they are, we’ll give them training and know-how and put them in a [REDACTED] logistic plan so they can get IUDs and medicines and whatever else they need. But we only work with them for, say, the IUD insertions for family planning. They may have other parts of their practice that they manage that we don’t interfere with or monitor.

Interviewer:	Okay.

P12:	That’s where the term “fractional franchise” comes in. Depending on which funder we work with, the disease is different. [REDACTED].

Interviewer:	Okay, so it sounds like you’re very much embedded within the current framework that’s been set up and you kind of enhance the activities of the community that’s already there.

P12:	I don’t even know… Enhanced? Possibly, in some ways. I think when it comes to like diarrhea treatment and things like that, they’re probably already doing some treatment of clients who have come in for diarrhea, but in our program in [REDACTED] I think most of the providers...

Interviewer:	Hello? P12? Sorry. I think you just cut off.

P12:	… and they were allowed to do IUD insertions, that kind of thing. Other ones that were doing counseling or recommending family planning options really didn’t have a lot of experience in it and so we trained them and brought them up to speed.

We might run across a provider who’s completely skilled in what we do and we make them go through the training ‘cause it’s a requirement, but then they go and do their things with other providers. They may have skills in other parts of healthcare but they may not have paid so much attention to, say, tuberculosis for some reason. Anyways, we go in and we give them the groundwork, the framework, and things to deliver services that we’ve been tasked to deliver.

Interviewer:	Okay.

P12:	That make sense?

[REDACTED]

Interviewer:	Okay.

P12:	The problem is that the public health infrastructure that should be caring for all those people is non-existent.

Interviewer:	I see.

P12:	So, they turn to the private sector of these informally qualified providers ‘cause there is no other; there’s nothing nearby. For them to get an ounce of decent care, they have to travel very far to go to a public hospital which may have a doctor there, so what the informal sector does is provide services to people who want them. Actually, I think it’s a benefit in general that these exist. What the detriment is is the possible quality.

Interviewer:	Okay.

P12:	There’s been a lot of studies that look at doctors in [REDACTED] —both trained and untrained doctors—about their prescription habits, their diagnosing capabilities and there’s been a lot of studies that show that just because you have a qualification for a doctor, it doesn’t mean that you’re any better than another doctor.

Interviewer:	Okay. Wow!

P12:	There’s a lot of issues with the medical system here, the public health system, hence why there’s so much money and so much time and energy and technical advising that goes into the public health system here.

Interviewer:	I see. Okay. So how did you specifically get involved with your organisation?

P12:	[REDACTED]

Interviewer:	Nice! And so what specifically is your role then?

P12:	Say it again.

Interviewer:	What specifically is your role?

P12:	I’m [REDACTED]

Interviewer:	Cool. Okay. The next question is very linked in with what your role is is how does your organisation define success?

P12:	Obviously the easy way is to say that we define our success by meeting our targets as determined by whatever funder’s proposal or whatever funder’s requirements are. That’s the easy way. I think long-term success would be an adoption of this and promotion of primary healthcare as an important part of public health systems. I think most of the healthcare that is delivered right now is what they call “vertical.” I don’t know if you all these…

Interviewer:	Yep.

P12:	…terms or what your background is, but… [REDACTED]

Interviewer:	Yeah.

P12:	But everything’s… It’s HIV services, it’s TB services, it’s diarrhea services, but that’s not what really happens in real life. All these providers… Obviously if you’re going to like a voluntary clinic for HIV, everybody’s there for HIV, but in general when you come in for some infectious disease, you don’t look for the diarrhea doctor. Or you don’t go to the pneumonia clinic. You go to a clinic. And they deal with pneumonia, diarrhea. They deal with acid reflux. They obviously deal with broke bones, scrapes and bruises. The clinic that you approach, the public health clinics that are out in the rural areas are just… deal with anything, and so what we’re trying to do here is leverage all these informal providers who exist out there and remember, we don’t make them. We find them so they’re already out there working.

Interviewer:	Yeah, I see.

P12:	So it’s like what do you do? Do you let these guys kind of do their own thing? And again, some of them are pretty decent doctors and some aren’t, so how do you bring some kind of uniformity to that? How do you monitor them and check their quality and make sure they’re following some standards?

Our way in has been through these vertical diseases, but in general [REDACTED] is looking to build the notion that funders need to not just only think about the verticals, but think about primary healthcare. These guys do all the triage out there, basically. They do a lot of referrals or anything that’s really complicated or dangerous. But they still… If you get sick in a village, they run to the village doctor whoever he is, so he needs to know everything. He has resources to decide whether you’re in a life-threatening situation or not so we’re trying to work with these guys to kind of build that capacity with them.

Interviewer:	I see. Okay, so in both…

P12:	Let me throw one more thing in there. When you talk about success, we didn’t talk about sustainability and one of the things is that these guys charge money for services. It’s not free. It’s much easier… We’ve had a much easier time in [REDACTED] with that because there is kind of a financial... The population is used to having to pay for some things. What we’re finding in [REDACTED] is that the populations in the really rural low-income places are so used to getting free handouts from all the aid donors that they don’t want to pay for anything, so it’s hard to be sustainable.

Another one is so… I guess it’s too long to engross, but to get the international community to understand the importance of primary healthcare and working on this primary healthcare and population health and also sustainability ‘cause no one can afford to set up a quality public health system to cover all the [REDACTED] million people in [REDACTED]. It just isn’t feasible.

Interviewer:	Yeah. No, it’s a huge task to try and do that.

P12:	It sure is.

Interviewer:	So it sounds like the way you define success is three-fold, so first one— meeting funders’ requirements, second— adopting or promoting primary healthcare, and also third one is sustainability. So, in each of these areas, how do you think about impact and outcome measures?

P12:	Well, the impact numbers for the first one—the donors—are really well-defined. There might be a 30% increase in case notifications or whatever. You see population impact in diarrhea-related incidents reduction. Very, very, very straight-forward… For impact on the primary healthcare, I think… I don’t know. I haven’t really thought about it. I think at this point building the infrastructure and the system for some… structure and quality among all these informal providers is important. The government of [REDACTED] wants to embrace them. Everyone knows they exist. Everybody knows they’re useful in one way. Everyone knows that they *have* to exist because there’s nothing else out there, but the government of [REDACTED] doesn’t want to formally recognize them for a lot of reasons I won’t go into. It’s obvious: they’re not training people, are they?

Interviewer:	No, it definitely sounds like a complex issue.

P12:	It is a very complex issue. So, when we talk about the outcomes and impact for that, for them to realize that their current public health structure is... They realize. They know it. I guess what are they gonna do about it? That’s where I think the big hurdle lies and we’re just gonna wait and see. Are they going to go on and accept these guys somehow at some level into the public health system? Probably not because all the qualified doctors and all the associations don’t think it’s a great idea.

Interviewer:	I see.

P12:	So I don’t really have an answer for the impact on that one. Better primary healthcare obviously will help everybody: better access to primary healthcare or access to a primary healthcare doctor who can access a real doctor via Telemedicine or some type of technology. It’s obviously fantastic and it spreads the ability for good doctors to contact large groups of patients. Then impact and sustainability, that’s actually one of the measures we get. “Can you have a 60% cost recovery?” Those measures are out there. 60% is something they threw out at one of the first studies we did and we haven’t even come close to it yet.

But, the sustainability model is really difficult in a lot of ways because (a) there’s a lot of definitions that you’re talking about. Sustainability of a certain program? Are you talking about sustainability of [REDACTED] as an organization? Are you talking about sustainability of these providers out there in the field to continue to do their work? It’s really hard to define sustainability.

Interviewer:	What kind of measures do you use to ensure that your program and organization remain stable?

P12:	What kind of measures do I use? It’s more of actions and thoughts. I don’t really measure. We don’t really measure sustainability.

Interviewer:	Okay.

P12:	I’m not sure… I have some ideas on how I may measure it when it comes to financials and maybe some service delivery, but I think a lot of it is looking at the adoption of use and the promotion of primary healthcare in the systems.

Interviewer:	Okay.

P12:	I don’t really have a measure.

Interviewer:	Yeah. It’s a very difficult thing to measure. I’m sorry—that was a bit of a tough question for you.

P12:	No, no, no, it’s fine because we actually… I was at what they call a [REDACTED] so myself and a bunch of other monitoring/evaluation type people got together early in July and sat around for three days and talked for a full day about sustainability and what it means and we basically came to the same conclusion is that it’s really hard to measure because sustainability...

First, you have to frame the sustainability of what? What do you want to sustain? Do you want sustain the organization? Do you want to sustain… whatever, so you decide that, and then it’s like what? What are you trying to sustain? Are you trying to sustain the idea behind it? Is it the simple financial sustainability, which I think is what everybody want. Folks, they want things that are self-sufficient so I think sustainability is not the right word. It’s self-sufficient… when it comes to financial things.

And we do! Like I said, the measure is cost recovery—how much money did you spend, how much money are you getting back, how much costs do you cover? That’s a pretty simple sustainability measure when it comes to financial and again I think everybody can kind of talk a little whatever they want, but in general it’s financially. “Do we have to continue to give [REDACTED] 12 billion dollars?” I hope that’s 1.6 billion because somehow those services are being paid for, but we work with the poorest of the poor. They don’t have a lot of money and you have to ask yourself, “Do you really think you’re going to have sustainability in low-income, low resource settings?” Unlikely. If there was money to be made out there, it wouldn’t be us out there. They’d be like Costco. Someone would be out there making money. It’s just one of those…

Interviewer:	Yeah. Just adding on what you’ve just mentioned, I guess in some ways it’s easy to measure money because it’s a number when you’re thinking about sustainability in [third commerce? 00:17:24] and the actual other aspects of sustainability, which we’ve briefly touched on, are much more difficult to try and define and define outcome measures for.

P12:	Absolutely. Yeah.

Interviewer:	Yeah.

P12:	Yeah, well put.

Interviewer:	Okay, P12. I just wanted to go back to what you talked about with the acceptance of primary healthcare as a more widespread model. You talked a bit about funders and government and trying to influence them. Are there any other sectors or parties who you try and influence?

P12:	Into adopting primary healthcare?

Interviewer:	Or with your program and your mission in general.

P12:	This might be a better question for [REDACTED], but… mostly governments because it’s... That would be the big win. We can convince people like the [REDACTED] Foundation that this is a good idea, you give us some money or whoever and we have. They’ve show their commitment by giving us nice, large grants at some points, but it’s the government that holds back this initiative.

Again, I don’t know if there’s a right or wrong answer either. I know enough about our providers to know that they’re a necessity out there but I tell you, if I’m in bad shape, I am getting someone to drive me to one of the big cities and finding a real private hospital. It’s just the level of care is not that great. So, how do you boost that? How do you boost that level of care? And without government regulation and enforcement…

That’s one of the things we do. Since we provide all these campaigns and IEC and communications, they get benefit from us. They kind of want to stay in the network because once in a while there’s a commercial on TV that says, “[REDACTED],” and everybody’s watching TV at some restaurant and they know that the guy next to them is a [REDACTED] provider. It’s neat. They don’t want to leave the network and there’s some kind of leverage there for that.

But besides that little bit of leverage, there’s no regulation, so really it needs to be the government has to decide what they want to do with these people, but that’d be the place to go. Obviously, we’ve convinced some donors that social franchising is a good thing… So for me, for [REDACTED], most of social franchising is with informal providers. Again. There are formally trained providers who are actually part of the Telemedicine system so they can talk to me but also some of the providers, very few of them *are* formally trained. All of the clinics that we use, the franchise clinics, they’re all informally trained doctors.

So when it comes to social franchising, I think the informally thing is more of an issue with us. I think if you go to other social franchising programs, they don’t deal with people who are informally qualified so I think I speak only from that experience. Also when it comes to sustainability and such, I can only speak from our prospective. Obviously you don’t need government influence to socially franchise qualified providers here because they’re qualified, but I think you’ve just gotta keep beating the drum that maybe it’s the primary care focus that we’ve kind of missed over the last couple of decades when it comes to developing public health programs. I know they’re trying to knock out the most important diseases, but now they need to start talking about population health in general and preventative care and not curative care all the time. It’s kind of a…

Interviewer:	Sorry, go ahead.

P12:	Go ahead.

Interviewer:	No, no, no.

P12:	I think we’re all in the same boat when it comes to that. I think we can see how the vertical system has worked for a degree, but it seems ridiculous when we’re pushing for a certain amount of care to be done to our patients on diarrhea but we’re ignoring *other*!… Their 16-year-old son’s broken leg or something. It seems kind of preposterous and...

Interviewer:	Yeah. No, I can’t agree with you more. It’s definitely a huge issue which is a nightmare to untangle, but it’s very important work to do.

P12:	[REDACTED]

Interviewer:	[REDACTED]

P12:	[REDACTED]

Interviewer:	[REDACTED]

P12:	Oh, cool.

Interviewer:	It’s very inspiring.

P12:	That’s good.

Interviewer:	Yeah. P12, I just wanted to find a little bit more about the specifics of what you do in your everyday work. How do you actually measure the progress of [REDACTED] and what does it take to be able to measure progress like in terms of resources of evaluation monitoring?

P12:	The measuring, a lot of the reporting is how may of this you did—silver statistics—so there’s multiple ways that comes into our system. Some is the old-fashioned way where someone writes down a number of cases on a piece of paper and then one field officer comes and picks it up and drags it into the local office and they type it into an Excel sheet and then send it over here. Fortunately since I’ve been here, we’ve become much more sophisticated in most parts of the program where there’s either a tablet-based system to enter data or there’s an online system. A lot of is we count! We count a lot of stuff.

Then as part of the marching process, you’ve got to verify some of this so there’s teams that go out and verify on a sample basis certain parts of the program that need verifying and often an irregular… Often isn’t the right word, but a bunch of our programs use incentives to drive provider behavior so whenever there’s a good-sized incentive involved or things like that, we send people out to check to make sure that in fact Client A was serviced by Provider B and those kind of things so that’s part of the system— kind of watching, keeping your eye out… How do I put this diplomatically? I think sometimes there’s more effort spent on trying and *don’t* quote me, but this is just between... You can think about this over a beer if you drink or something like that, but more people spend more time and energy trying to beat the system than working the system so all of a sudden we’ll see a spike of so many cases and we’re like, “How did this guy somehow get through…?”

All the incentive things, usually there’s a photocopy that needs to be made, like if they’re giving a TB notification we have to get copies of tests before they get reimbursed, but there’s always some way for them to somehow work their way around the system and get the reimbursement without doing the work. And again, I swear it’s harder for them. It takes more energy to do that, but they still would rather do that than actually do the thing, so there’s a bunch in mind that goes on. People can make a decent amount of money in the real space if they do a lot of services for us so we gotta keep an eye on that. A lot of it is just watching and monitoring.

When it comes to evaluation, in some cases depending on the funder’s desire and the availability of money, we obviously deal with outside organizations to give us a look at impacts... and it might be a long-term population based huge study—a couple of rounds in 20,000 households, big things like that—or we also often hire local, external evaluators to maybe look at a certain problem or help fix this research issue to see... for whatever reason.

Sometimes it was part of a quality control. We had a local survey team go out and just sit in the office with a provider for 2 days, just document what they do, and obviously we had structured forms. They knew what to fill out. If people with diarrhea came in, how many questions did they ask the kid? Which questions did they ask? Did they about blood history? Things like that.

So, we’ll do some evaluations and some external evaluators come and check our quality, check the behavior of our providers, interview the clients post-care. Some of the other evaluations have to do with logistical systems where one of the funders has gone in and spent a bunch of time with our local business deals here, trying to really understand our logistics system for moving supplies around and seeing how efficient it is compared to a bunch of other programs that are funded, so it’s us and 2 other programs that do similar distribution of OS and zinc. (The mandate is to distribute OS and zinc out in the field, but we all do it in quite different ways.) Where’s the best bang for your buck? So, those type of evaluations happen.

Interviewer:	I see. Okay. So in conducting your evaluations, what do you think are the main challenges that you face?

P12:	For evaluation?

Interviewer:	Yeah.

P12:	Well, part of it is the money. Who wants to pay for it? That’s probably the biggest thing—[inaudible 00:28:37]…

Interviewer:	Okay.

P12:	Again, we could do some of this stuff internally, but there’s not as much weight as if does if done by an external person.

Interviewer:	Okay, so if you… Yeah, sorry, go ahead.

P12:	Go ahead. No, I won’t go into that anymore.

Interviewer:	I was going to move onto the next question, but no, keep going with what you were saying.

P12:	A lot of it is… I’ve been here for a couple of years and before I worked here, I worked for an organization called [REDACTED]. I don’t know if you’ve heard of them, but they do a lot of operations research studies, that kind of stuff—evaluating programs like I sit in now—and one of the hard parts about evaluation is the implementer needs the information as soon as possible. Like it’s no good if you do a baseline, you tell me in two years that, “Oh, there’s a problem with your program.”
“Like you know what? It’s only a three-year program. I can’t make any changes now.” That’s been a very hard part about evaluation and I knew it before and I know it even more now that that answer doesn’t help me now. I needed it a year and a half ago.

Interviewer:	Yeah, I see.

P12:	So, a lot of the things that I thought about here and I don’t really have a good answer for yet is trying to figure out how to use monitoring data and these short little studies then kind of course-correct as you go and I think that’s more the way to do things, like it makes my stomach curl now and I almost hurl (for a nice rhyme!) that when I see people drop like 5 million dollars on a end-line baseline study, see population [inaudible 00:30:10].

And granted it’s important, but they don’t spend any money to kind of work on things along the way or a steady… like they know that there was no change, but they’re not gonna know why. Or that there is change, but they’re really not gonna know why. They’re going to know something, but they’re really not going to document the processes enough and it just ends up being that when people get budgets, they want spend… 60% on implementation, 20% campaigns because they’ve got to raise awareness, right? The next 15% is administrative costs. They’re like, “Oh yeah, M&E. We’ll give them 5. Oh, evaluation? 2%.”

Because of course our limitation works fantastic because we thought of it and wrote a proposal about it, so it’s like shame on the people who write it and think everything works perfectly and shame on the funder for letting it be that way, ‘cause I really don’t have a lot of answers to why things work and didn’t work here. I know *some*. I have some evidence, but a lot of it I don’t know and I’ll never know because it’s passed. Everybody talks about these program, but often they’re not really well-designed or well-managed. That’s another thing too when it comes to… (Getting on my evaluation high horse.)

… You need to test two things when you go into these programs. One is the intervention plan, whatever it was. It may be like our social franchising, trying to reduce the risk, the uptake of OSC zinc, drop diarrhea and [inaudible 00:31:45], et cetera, but it’s also the ability of the management team to execute the plan. There have been places where I think the plan is good, the execution’s been terrible. It’s also been the other way around. It’s like, “You’ve got a great team here, but the intervention... It’s getting washed out.” It’s not happening because of all the things that are happening.

There’s a lot that goes on and no one ever really evaluates the management. I’ve never seen that anywhere. Maybe it has, but *I* haven’t, and that’s one thing I think I would look at first like, “What are the management decisions that got you here? Why’d you make that decision?” You have to understand that.

It’s never part of the impact packages, like we just got evaluated by a big team and they said, “This is the one thing that we think that affected your low impacts.” But… they just kind of guesstimated, like they didn’t do any real in-depth look at the management style and what we did, the decisions that we made. It’s just more than that.

Interviewer:	I see. So, is that something you have looked into or are planning to look into for your organisation?

P12:	No. I won’t look into it here.

Interviewer:	Okay.

P12:	No one wants to be judged that way. I’m being frank. I think that if you talk to some people, it’s like, “Oh yeah! Our doors are always open,” and stuff like that. You’ll hear this not just from us, but everybody— “Oh yeah, we’re happy to tell you again”— but when you really get down to it, and not just our organisation, a bunch of other organizations like [inaudible 00:33:16], no one really wants to their work to be looked at with that fine-tooth a comb.

But when you make decisions about when to start campaigns or not to collect certain data or things like that, it affects and I think they really need to take a better look at management style. There’s no shareholder base. You can’t vote with your feet in rural [REDACTED] when it comes to going to your local provider. (In the village, you have one or two.) There isn’t much choice so it’s not like that they can decide to go to a different hospital if they’re not getting the cure they want or they need. They can get in a car or a bus or whatever and take the trip, but it’s… Sometimes, the cost is new to them. You’ve gotta watch out for these people.

Interviewer:	Gosh! That’s quite a unique perspective. Thank you for sharing that with me.

P12:	No, I really think that. I’ve been in two places throughout my... So, for [REDACTED], I vetted a lot of programs. I’d either sit with an M&E team and a program team for couple of weeks where I’d get an idea of what’s going on and then you come back and baseline present. Sometimes they just didn’t… They’re like, “Why aren’t you doing this?!” It’s obvious to me that we talked about it when I left, but I haven’t seen any change in behavior because they have a different mission and I think another thing that comes to public health is that often the last year of the program, people are scrambling so much for new money that everything kind of falls off and I don’t know how to get public health to understand that if they really want sustainability, you can’t have your program people spending a third of their time trying to find new money the next year…

Either your program’s going to be sustainable or the organization. That’s where sustainability kind of has a conflict of interest. What did I do last couple of weeks? I’ve been writing proposals. What am I being paid right now? I’m being paid to analyze data for [REDACTED], but I’m writing proposals… ‘cause we have to because we have to get more money.

Interviewer:	I see. It’s a bit of a Catch-22.

P12:	Little extra philosophy there today for you, I guess.

Interviewer:	Yeah. I just wanted to take the conversation in a slightly different angle. A lot of health initiatives, particularly when they start off despite best intentions, are harmful to the communities that they serve. Have you possibly thought about ways which your program could be harmful?

P12:	Harmful? Of course.

Interviewer:	Can you detail a bit more?

P12:	… I don’t think our program’s been harmful, but I do think our network... How am I gonna say this? ‘Cause we’re a fractional franchise, we only train in Bihar for diarrhea, tuberculosis, and calazar treatment. We know for a fact that a lot of these doctors do surgeries and they have a surgical table and they give drips and injections and all kinds of things.

So, as an organization, we have this fractional franchise so it’s, “Oh, it’s not really us. It’s them! They’re doing their own thing.” But are we partly responsible? By bringing them business from our campaigns saying, “Go seek this [REDACTED] provider?”, could they get some child with diarrhea whose brother is having a pain in the stomach who’s 17 and all of a sudden the guy decides to take out his appendix and that kid dies? Yes! I’m sure that could happen, so are we somehow… giving them permission to be a doctor in those places because we slap our stuff on their side and we talk about them on the radio? I don’t know.

We’re validating them themselves as a trained provider. Do I think the local people who live in one village in Vaishali district of Bihar understand when they go to this doctor that we only trained them on diarrhea and pneumonia and tuberculosis? Probably not. Even though our campaigns may even say it specifically, but I think in general… And in a lot of ways, we’re driving business high. We’re making his business a little better, bigger, stronger ‘cause he’s getting more money. I don’t know. I don’t know. We try to get on top of quality. There has been some [inaudible 00:38:01]. There’s been 10,000 providers in [REDACTED] so there has been a couple of instances where bad things have happened. That could happen anywhere.

Interviewer:	Yeah.

P12:	But then you look at the quality of [REDACTED] medicine and, in general, it’s pretty shitty. I was sick in February. I had a really bad lung infection. I went to the hospital that you pay to go, the one that all the ex-pats go to because we all think it’s best, and I went into the emergency room. I had the lung thing. They didn’t ask if I smoked or *any* smoking history, any exposure that could have made my lungs act the way they were. They didn’t ask any of that! I’m like, “I’m a statistician, but I know more about medicine than he does!” like he didn’t ask me any patient history. They didn’t even ask me if I was allergic to anything. I’m in, like, a nice hospital. It’s like, “You’re clueless!”

So, it happens *everywhere*. In general, I think, and [inaudible 00:38:51]. So, there are good doctors here—don’t get me wrong—but there’s a lot of them that aren’t so great. I think that that kind of stuff wouldn’t have happened if I had been in the US. Someone would have asked if I had smoked at least. There would have been the proper algorithmic stuff. It doesn’t happen here... and I think there’s ways that they can push more algorithmic medicine. That’s why I joined [REDACTED], but it hasn’t really matured.

But algorithmic medicine is the way to go. I don’t want to teach an informal provider out in the village how to diagnose something. I want to teach him how to go through my simple algorithm on the tablet so he can diagnose anything, but the problem with that is that no doctor out there wants to be seen as a guy that just knows how to use a tablet. He wants to be seen as the guy who knows about medicine and the pride and all that ego crap that goes along with being a doctor. I fight that battle, too.

Interviewer:	I see. I see. Okay, so it sounds like there are just generally a lot of external battles which aren’t related to the program but could potentially be seen to impact the program outcomes.

P12:	Yeah. It’s complicated. Yeah, yeah. It’s not like a straight-forward, “You’re going out and passing out condoms.”

Interviewer:	No, fair enough.

P12:	[inaudible 00:40:07]

Interviewer:	Yeah. So, P12, we’re actually up to our final question now.

P12:	Alright.

Interviewer:	So, to end off on a good note, in a perfect world, what do you think would help you do your work better?

P12:	What do I think would help me do my work better? You mean my personal work or the work of our organisation?

Interviewer:	In both.

P12:	Good answer. My personal work? I would like to see… cleaner data, which I’ve fought for a lot, and pre-data is much cleaner now. Before, it was just not…

When people talk about evidence-based decision making, all these things, honestly, I don’t see enough of it. And not at our organisation. It’s a lot of other places, too. It’s just the information’s not there. A lot of the program teams don’t have that kind of analytic training. I see a lot of social franchising network places run by people without business degrees, but it’s their job as a business. I would like to see a retooling on a lot of these, to really look at data in a harder way, and then the desire to get more data. It’s hard to get data and good data.

Interviewer:	Yeah, I see. Can you just define a bit more what you mean about cleaner data?

P12:	Well, I mean a lot of it is… It could be as simple as I need to know ages of kids when we get… We have this thing called a [REDACTED] where it’s a special training and some providers who sign up for it get some extra equipment—a nebulizer for pneumonia. Actually, they get like a shiny, little picture to [inaudible 00:41:56] from us. (It’s kinda cute.) They get this extra stuff to do the thing, but I need to know... They can treat anybody with diarrhea. It’s for kids, but we’re focused on under 5, so if they don’t write down the age, what do I do with that?

It’s like this simple data stuff and you’re dependent on these providers who, really for the most part, have never written down anything. Also, some obnoxious American comes into their door in some place and says, “I need you to fill out these 20 columns.” They’re like, “What?!” So, I feel their pain but also I have to tell our funder what’s going on. We need to know that you diagnosed this guy properly so we know that you’re not killing people. It’s a battle there and the program teams *don’t* want to interfere with the teams, but I’m like, “I can’t tell our funder that our quality is good if I don’t get pieces of information…” It’s really hard that way. Everybody wants information but no one wants to spend the time, energy and money to collect enough of it.

Interviewer:	I see. Yeah. No, that’s a big issue because you’re just trying to overcome….

P12:	And finally before you disappear, one of the big things that I have to puff up my nose at is you only hear about good programs. You never heard about programs that fail... and there’s a few of them out there that... I know there’s a lot of impact evaluations. I’m in the hood. I sit at the bars with all these guys that have failed and the data doesn’t get published. I don’t know. Personally, some of my biggest lessons have been my failures and we don’t do any of that in public health… Not everything works. I know for a fact. We need to hear why things didn’t work and it may be because “management team was unsuccessful in implementing program to plan.” End of sentence. That might be just what it is, but we need to know and no one ever looks. I think that’s a big disservice to all of us.

Interviewer:	Yeah. No, definitely.

P12:	I’ve worked in behavior change communications in Africa for nearly a decade. If they all worked, I would’ve been out of a job. It *doesn’t* work as much as everybody say it does. But, you have to have good results or you won’t get any money. There’s always that. It stinks. Anyway…

Interviewer:	Yeah. No, I really can’t agree with you more. It’s extremely important to be able to publish data on things that don’t work because other people won’t be destined to repeat the same things, but there’s definitely not the incentives there to do that, which is a bit of a problem.

P12:	Absolutely. Anyway…

Interviewer:	Okay. Alright, P12. That actually brings us to the end of the interview and I did want to respect your time, so...

P12:	That’s fine… This is a quiet afternoon for me. My chunks of the proposals are done. I’m in-between waiting on something for tomorrow... I’m an awful…

[end of recording: 00:44:54]
