# Supplementary material for: Supporting Better Evidence Generation and Use within Social Innovation in Health in Low- and Middle-Income Countries: A Qualitative Study
Source: PLoS One. 2017 Jan 26;12(1):e0170367. doi: 10.1371/journal.pone.0170367 (PMC5268497; doi:10.1371/journal.pone.0170367)
Supplement: S1 Dataset — (ZIP) [file pone.0170367.s002.zip › Data/Data - Interview transcripts/P13.doc]

Interviewer:	First question is just tell us what you doWhat’s the problem you’re trying to solve? The mission, the organization, how you’re involved.

P13:	Yeah. So, how familiar are you with our organisation at all?

Interviewer:	Familiar…

P13:	From the website?

Interviewer:	Yeah. No, I’ve looked into it. I think it’s more just for the record to have somebody articulate it in their own words, if that’s possible.

P13:	Okay. Our organisation vision is really to help health systems integrate, the ways of addressing patients’ social needs into a standard part of healthcare delivery. [REDACTED]

Interviewer:	Alright. Excellent! Thank you. How would you say your organisation defines success, like what are the key outcomes or impacts that you are thinking about when assessing your work?

P13:	Well, we’re thinking about these all the time and actually this is a really good time to ask this question because we’re starting to articulate the levels at which you would expect to see both outputs and outcomes… And I can talk a little bit how we use those words since everyone uses them a little bit differently, which can be complicated, but I think a first level is really understanding the type of impact this work has on patients. You froze up for a minute. I just want to make sure you can still hear me.

Interviewer:	I can still hear. I’m all set.

P13:	So, when you think about this, this is the core part of either our own service delivery or as we help to train and facilitate hospitals doing this work independently, their ability to address patients’ social needs at a higher level of quality. There’s a set of outputs and outcomes that are associated with that level.

Our organisation is also operating sort of one level above that which is how do you engage healthcare leaders — leaders of healthcare institutions — so that they understand and are enabled to actually address patients’ social needs and this can be viewed at the tactical/clinical level but also at the highest level of the healthcare delivery system. Does that make sense?

Interviewer:	Yeah. That sentiment actually parlays into the question of influence, so obviously the heath system in [REDACTED] is… particular vis-à-vis developing a [REDACTED]. Who does your organisation aim to influence and how does it do that?

P13:	Yeah. This is a good question and again this is so apropos because we’re actually, right now, sort of honing and developing what are the set of metrics that we believe we should be holding ourselves to from a sector transformation standpoint. And you can imagine any healthcare system, the [REDACTED] healthcare system in particular, there are a number of entry points and nodes into changing the sector and our organisation believes that its influence — because of its engagement with hospital leaders — is through healthcare delivery system right now.

And so when I think about ways in which we define sector influence, it’s engaging a set of hospital leaders who are operating the vanguard, really understanding the importance of and thinking about how to deliver against addressing patients’ social needs. Then one step beyond that is a set of leaders… (Sorry. I talk with my hands and this is what happens when you’re on Google is that you’re going to get a lot of hands. Bear with me. Exactly! Thank you for doing it too. We can both do it.)

So, engaging that level beyond the chart. How do you convince and how do you start to convince that next layer of healthcare leaders, not the folks that are on the vanguard that you’re gonna go deep with, but how do you engage the next level and engage them with trainings and tools that enable them to start doing this themselves? I think along the margins of that what else would create a supportive environment for healthcare delivery systems to do this, so engaging infrastructure leaders that run [REDACTED] systems and how are they thinking about integrating social needs into the systems that they’re building that are used within the healthcare delivery systems that we work with?

What this doesn’t include necessarily right now is a deep engagement around policy and that is… Just to articulate, just to be clear, that is a specific choice on our organisation’s part around where we think we are best positioned to influence but also where social change will come from in this sector on this topic.

Interviewer:	Interesting. So how would you identify these vanguards and what is particularly helpful in sort of pushing them to acknowledge this biomedical only approach as somewhat insufficient? Or alternatively, they see that but they understand that your organisation provides a viable solution in that case?

P13:	I think there’s sort of two pathways. Because of the macro factors, both policy and payment reform that are impacting hospitals, we’re in the unusual position that hospitals are actually coming to *us* asking for advice on how to do this work so we have a large set of hospitals that come to us, not all of which are ready to engage deeply, and so it enables us actually to select a smaller group who are ready to engage deeply and will have alignment from the C-Suite down in order to do this work and potentially do it at scale, which is a very unusual place to be.

I’ve worked in a number of nonprofits and rarely do you have thousands of inbound requests for support and training. So, that is nice. That makes it easier to identify a set of people who are really passionate about this and then I think in addition our organisation is — at the sector level — incredibly serious about it not needing to be our organisation’s solution. There are versions of home-grown options if they are done with enough rigor that can be just as powerful. There are versions of what many other people would consider competitor products. They’re approaching it a little bit differently, but there is a enough room in this sector and our organisation’s goal is not your organisation everywhere, that the objective is not just who is your organisation’s working with and how we consider them the vanguard, but actually how are we getting together a group of hospital leaders who care about this regardless of whether they’re our organisation customers or not.

So what we’ve done recently and I think it’s been almost two months since we brought this group together, but [REDACTED] (who you reached out to initially) actually brought together a group of about 20 to 30 hospital leaders — some of whom were our organisation’s customers, some of whom were not — to actually get everyone in a room and ask that question that you’re starting to ask which is “Is there a shared agenda around addressing patients’ social needs?” and if so, what is it?

And this group got together and I think really affirmed that there is the value in bringing hospital leaders together to talk about this and there is a potential for a shared agenda… No one has landed on that yet, but they are starting to think about what are the pillars that are needed in order to engage more broadly across their hospitals, but also proliferate this across the sector. And that’s pretty exciting and that pathway is one that’s peer led and so that’s a pathway where you have a set of leaders who are excited about this work, whether they’re our organisation’s customers or not, and you actually feel that they can articulate a potentially shared agenda that they are using to influence their peers, which you and I know is just much more powerful than our organisation out there shouting about social determinants from the mountaintops. To have your peers articulate a need for it is an influence strategy that I think is likely to get us to scale much faster, get the sector to scale much faster... To be clear, not our organisation.

Interviewer:	Who is in that room? Who does your organisation consider to be its peers or allies in proving these types of services?

P13:	[REDACTED] could send you the full list. It was co-lead by us and some companies, I think… an organisaton… for some reason, I want to say this other one but I’m not sure. So, [REDACTED] can certainly give you the full list or I can make sure [REDACTED] can send it to you just so you have it.

Interviewer:	You are now frozen. Oh, you’re back.

P13:	I’m still here.

Interviewer:	… So we talked a little bit about level two. But back to the patients. We talked about them as a level of where we [inaudible 00:12:13] outcomes and impacts. What are the actual metrics you look at there and how do you measure those metrics?

P13:	So to be honest, patients is where I spend most of my time — partly because that’s where our organisation has spent most of the time over the past 4 or 5 years and so that’s where we’ve gone the farthest — and when we started this work, the question that we were testing was “can this be done inside a hospital?”, like it was actually that basic and so I said that as a little bit of a context to why we focused a lot on process metrics to start and I also think it gets to some of what I read in your consent form or the description of what you’re looking at which is from an implementation perspective, how can we use it to actually understand our own performance?

So, we spent a lot of time doing that around, answering the question of whether our organisation is delivering high-quality implementation … I sort of think of it as two inverted triangles. Over the past five years, our organisation has spent a lot of time coming from a lot of different articulation nodes and models in different sites and narrowing that in to one core set of activities that we felt like represented high-quality implementation and what we’re doing right now is actually breaking that apart and starting to proliferate a very clear set of product offerings that take those core pillars and pull them into different types of products with different types of workforces and different types of patients.

And so, this narrowing activity was highly informed by our dataset and so a lot of that was focused on what I would call output metrics. If you’re thinking about a logic model, it sort of falls in the middle so things like using our backend technology to understand how many patients in roles, how many times did we actually reach out to patients, what types of needs did the patients identify, what resources were they connected to, whether or not those patients had successful or unsuccessful closure. (There’s a variety of success categories that we have.)

And so, all of this requires: (1) a strong backend technology system, but also a clear training program because we have hundreds of advocates across the country who rotate in by a semester basis who are doing this work and so you gotta be able to train them in a way that’s scalable so that they’re all entering the data generally the same way. Then, we have program managers on the ground who, in addition to working with the advocates and making sure that they’re delivering service to patients, are also doing some random case audits in the system to make sure that the data is actually reflective of what actually happened.

We sort of extracted that dataset and all of those outputs are things that we use to better understand the [finality? 00:16:08] of the service we’re providing, questions like “how important is it to actually do follow-up with the patient? How much does that influence their ability to get to a successful connection? What are the different ways that people close their relationship with us and can we improve any of those closure reasons? Are there particular patient populations or landscapes that have a lot of unsuccessful connections?”

Or, the inverse. We’re just looking at some data on immigrant and there are very different needs requested by immigrant populations than non-immigrant populations. What’s interesting is that from a success standpoint, from a closure standpoint, they have a success rate that’s actually pretty similar, so they need different things. They probably, because of the [REDACTED] system, have access to very different resources than people who are naturalized citizens or green cards and so that means that our teams are actually having to pull different resources within the landscape in order to create successful connections at the same rate.

We sort of mine through our data to actually figure out were we serving the populations that we need to serve, what does it tell us about the resource landscape, and how can we do this better. So, that’s sort of one level. And then, over the last 2 years, we’re getting a lot of questions around “That’s great! I’m so glad you guys have a high-quality program. It’s why we hired you. What type of impact are you having? What type of clinical impact do you have? What type of utilization impact did these types of services on patient trajectory?” So that’s where, even in the last 6-8 months, I’ve been spending time thinking through what is the likely impact—both on the clinic itself, on the patient, and on the patient’s behavior—around utilization that might clarify the… holistic package of value for health...

Interviewer:	You have just cut out…

P13:	That was unexpected.

Interviewer:	No worries. You were talking about “holistic package of value” when I lost you.

P13:	Yeah, yeah. It’s a funny combination of words too when I say it… It’s reflective of the fact that I don’t think there’s a silver bullet here. I don’t think there is for all patient populations across pediatric to high-utilizing adults, I don’t think there’s one common... outcome metric that is really track-able across all these patient populations and so I think it’s thinking through what are some of the likely pathways and starting to build the clinical evidence that is going to be necessary to create the incentives for hospital leaders to be able to invest in this type of work.

Interviewer:	Okay. Do you—you being your organisation —ever think about or measure or attempt to measure potential harms of the program, which sounds like a strange question but obviously there’s been many programs with great intentions that actually when we look at long-term outcomes of the group, the intervention group does worse in some comparative and that’s strange and difficult and we often don’t see that until we do the type of implementation work maybe you’re already doing? Anyway, is this on the forefront of anyone’s mind or is it more a background thing?

P13:	That’s a fascinating question and I don’t think one I ever articulated in that way, so we have had.. It’s funny. I just finished some evaluation work and one of the questions that we looked at, there wasn’t a negative impact but there wasn’t an impact and so we often ask questions about why, like places where we would expect there to be an impact and there wasn’t, but not specifically searching for places where we’re doing harm.

That’s just a fascinating question. We have not dug in there. I think part of this is with folks who are on the ground who have a background in social work running our program. Many of them are looking for places where patients are not having the experience they should be having and so part of it is I do rely on the frontline to be managing the quality of the implementation and patient experience and we do do anonymous follow-up surveys with patients via text and ask them what they thought of the program as a way to make sure that they are getting the experience that they expect to have and those are — just candidly — they’re overwhelmingly positive. So, I think our Spidey-sense around this feels… is at a translatable concept. It feels like we’re gathering information like this in common sense ways, but actually haven’t thought about integrating it into evaluation.

Interviewer:	Fair play. So, that’s one potential avenue of evaluation to go down. Are there other big questions that have come to mind? “We wish we could answer whether our program does X, Y, Z…?” And what are those unanswered questions and what’s been difficult about, as someone who’s sort of charged with that, about gathering that data or figuring out those counterfactuals? What’s the barrier?

P13:	I want to be clear. I actually think there are more unanswered questions than answered questions in this space right now by a really large margin. I think we’re at the stage where… And I was just talking about this with another one of our researchers. He did the very simple work of overlapping our patient populations with the clinical data to say, “Do patients who actually screen positive for social needs in the clinic have different types of clinical diseases at different rates?” This is literally the most basic question that can be asked and I don’t think anyone has actually looked at it.

So, we are… I’m just setting the stage. I actually think that’s where we are in this space, asking some very basic questions and following some paths that are clinically informed and some paths that seem to meet a threshold around common sense that someone needs to go out and actually do some research around, so whether it’s our organisation or whether it’s by peers in other organizations, the group of folks is out there asking some great but pretty simple questions.

One of the things that I think is actually hardest right now or the patient population that I think is trickiest for us is actually pediatrics. Kids for the most part are really healthy, even when they are faced with some pretty challenging home and life situations and so they’re not a population where we’re dealing with kids who are sick, who have an immediate pathway for improvement... And often, the clinical impact of their early childhood situation takes years to really manifest itself. So, all of these are good things from a patient health perspective, but it means that doing a non-multi-year longitudinal study around this population is challenging to really define value and yet I think there’s a common understanding that there is a huge amount of value in helping children make it to their doctors’ appointments by enabling the parent to be able to get there, making sure kids have enough food in their house, which many of our families don’t, and making sure that kids aren’t living in situations where there’s cockroaches and mold and other airborne contaminants that are affecting their ability to breathe, so like really basic stuff but the ability for evaluation to create this ironclad case around pediatrics I think is challenging.

Interviewer:	Are there things that you think sort of in an ideal world would help you answer these questions, like resources that don’t exist yet and you would like to see?

P13:	Yeah, it’s funny. I’m heading out this week to [REDACTED] to talk with a researcher in this space who has done some work for [REDACTED] Foundation, to look at what is the clinical evidence that exists in this space, and I think the goal is to be talking with her around how do we bring together a set of researchers. And to be clear (and I should have actually said this from the onset but I’m sure you’ve seen it from my bio), I’m not a researcher, like I facilitate and lead this team. I do not have a PhD in evaluations research which is actually important and I think enables me to sit at the table without a strong agenda, but getting researchers together so that they are talking to each other around what is actually needed and to have that initial conversation to say, “If this work is as nascent as it seems to be, what are the ways in which it needs to be strengthened, not just for our organisation but for any hospital or any peer organization that believes in the need to address patients’ social needs?”

And so, I think that’s a question best asked by the folks who are actually doing this research and I think the set of hospital leaders should have input into that. This is a sector-level agenda issue, not our organisation’s issue.

Interviewer:	Okay, fair play. Well, we are just about half an hour so, like I said, I do want to respect you beginning your day but just also want to allow you the opportunity. Is there anything you want to say about evaluation or to the [REDACTED] Foundation, “Hey! It’d be great if you guys could X”? Something I didn’t ask?

P13:	No. [REDACTED] have been great and actually we’ve had a number of conversations with a particular person who I’m sure you’ve worked with extensively and they have been incredibly helpful. I’m sure that that will continue, but I would love it if it does. I think that’s great. I guess the only question I have is where does the information you’re collecting go next and how can it help inform the broader agenda across the sector?

Your charge likely goes beyond [REDACTED] healthcare and so I think there’s probably a set of folks you’re talking to for which this is particularly relevant and then a set of folks who you’re talking where this is really not relevant, but I’m sort of curious how can we pull this in? How can we actually have this active dialogue because I feel like there are a lot of people who are asking questions like the questions that you’re asking and I would love to get those people together in a room to say, “Okay, what have we learned and where do we go next?” Actually something that your foundation can do.

Do you culminate in bringing people together? And that would hopefully tag-team off of a sector-wide C-Suite level conversation that’s happening in the same location in November so that it’s tactically a place where we might be able to start to pull some of this together… So, yeah. If you have interest in that, I will sort of follow-up as that activity takes shape, but I feel like that’s a great place where if you’re starting to gleam outputs from your work, that might be a great place — if they’re relevant to the [REDACTED] market — that we pull them in.

Interviewer:	Great.

[end of recording: 00:32:42]
