# Supplementary material for: Supporting Better Evidence Generation and Use within Social Innovation in Health in Low- and Middle-Income Countries: A Qualitative Study
Source: PLoS One. 2017 Jan 26;12(1):e0170367. doi: 10.1371/journal.pone.0170367 (PMC5268497; doi:10.1371/journal.pone.0170367)
Supplement: S1 Dataset — (ZIP) [file pone.0170367.s002.zip › Data/Data - Interview transcripts/P14.doc]

Interviewer:	So, P14, thank you very much for taking part in our research. Just for the record, I just want you to tell us what do you do for your organisation?

P14:	What I do, I’m the program officer for [REDACTED]. As you might know, our organisation works across the country. We work in [REDACTED] countries across the globe and [REDACTED] is part of that, so what I do[REDACTED]

Interviewer:	Okay, that’s great. Okay. And can you give me a bit of a summary about your organisation and in particular, what kind of work your organisation are doing in [REDACTED]?

P14:	So as I mentioned, we work across many countries and across the globe— [REDACTED] countries and [REDACTED] is one of those programs. We started our program in [REDACTED]

So, our work is basically improving care at different layers of the entire country’s health systems, training the health providers, and actually we recently started doing/incorporating research aspects in our work which was basically intended to continue evaluating what we are doing and see what are the gaps that are remaining.

[REDACTED]

Interviewer:	That’s great. That’s great. Particularly drawing upon the research aspect you talked about, what’s the big question that you want to answer about your impact or your implementation of the various activities you undertake?

P14:	Basically the research is intended to: A) know the burden of disease— really try to understand what are the major burdens of the disease that are affecting the communities that we serve. So, in knowing that, that gives us a guidance on how we allocate our resources. Where do we put greater investments and where do we need to focus on? Where do we need to do advocacy to improve the health of the people?

So, the research is basically: 1) intended to show us where the greatest needs are to improve the health and wealth of the people. And second, research allows us to evaluate the programs that we are already, that we are currently running to see the impact, to see the progress that we’re making, to measure effectiveness of our programs, and the areas as well we need to improve and do a much better job, so basically it’s intended to do that and we’ll be moving forward with new initiative of the university: training the healthcare providers in understanding the need of research in the work that we are doing and how that can guide the rest of the work we are doing.

Interviewer:	Yeah, wow, that’s great! That’s really, really great. It’s very important particularly building up that research aspect and I’m very glad to hear you’re taking the initiative to do that and it sounds like it’s going to be an invaluable addition to what you already do. So talking about that, I just wanted to get an idea about how your organisation program in [REDACTED], how do you define success? How do you define the impact of what you do?

P14:	That’s a great question. All our inter-nations target the most poor and vulnerable communities in the country so that is where our major focus is, so all of our targets… all the programs we run are intended to serve the poor and the way we measure success is basically tracking different indicators on how we are improving the life of the people, so we support three district hospitals in the country. We support over 41 health centers in the country which are primary healthcare facilities that most of the people from the villages seek care. We also work with the community health workers who help us really do the work and they help the community level that includes sensitization and pro-national campaigns, so the way we measure success in all of those areas we work in is in tracking different indicators on how we are doing.

We track indicators related to number of deliveries of women who deliver at our supported facilities. We track indicators related to maternal and childcare numbers in the places where we work. We track different numbers of healthcare average, how many people are accessing the healthcare, so it depends on the program, but what I would say is that each program we are implementing has a defined number of indicators that we track on a quarterly and yearly basis and that’s where we can measure success of our work.

Interviewer:	Right. Okay, okay. Wow! That’s a really great system that you have, to have indicators specifically for each project that you track. So the indicators that you track, do you collect the data for those indicators based on a formal evaluation process or what other process do you use?

P14:	I would say both. There are some that are routinely collected at all the facilities that we support and there are others that we initiate evaluation.

The one example that I would give on the routinely collected data is a health system strengthening project that we have been running for the last five years in the eastern providence districts that we work in. We’ve developed a quarterly evaluation tool where we send our data officers to collect data from all the target health centers and we analyze those data that are collected on a quarterly basis, so that is one of the routinely collected data, and there are some—mostly new projects or new initiatives— that we normally launch and those we launch some specific evaluation projects at a certain time. So, I would say there are both. There are routinely collected data and there are some we initiate specific evaluation projects to measure the success.

Interviewer:	Okay. Can you give me an example of some of the specific evaluation projects you’ve undertaken?

P14:	There are a couple. One of those is one initiative called this project. This is an initiative we launched that was intended to improve neonatal care to health center and hospital level and this was really innovative because it was bringing in the component of putting in place a checklist to the nurses and doctors defining what needs to be done to urgently take care of a newborn and it has another innovation that are intended to train nurses, midwives, and doctors who specifically provide care to newborns.

So, we started that as a new initiative, and after six months we wanted to evaluate the effectiveness of the project. When we did that, it actually showed that we reduced the death of newborns by 30% in the two districts that we launched the initiative, and then we built on that. We actually have a grant in the pipeline that we are applying for to expand the program to most, 7 districts across the country and that is basically based on the successes that were shown in the evaluation that we did and now we are bringing the innovation program to scale, so that is one thing that I want to highlight.

Interviewer:	Wow, that’s incredible! That’s a phenomenal change that you’ve managed to initiate in just the first pilot. That’s absolutely incredible. Well done! Very well done.

I just wanted to clarify what you mean by “routinely collected data” when you talked about the healthcare center strengthening, so you mean routinely collected data as in you have specified time points when you come and collect the data? Or do you mean data that is already collected within the communities you’re working in and your data offices will just take that data?

P14:	Yeah, maybe I want to clarify that so routinely collected data… a kind of data you would find at the health facilities that we support. Basically, they record every single patient who comes to seek care in the facility, so they have that in their database to say how many daily…

Interviewer:	So it’s existing data?

P14:	Yeah, existing data.

Interviewer:	Okay.

P14:	So what our data officers do is on behalf of our organisation is going to the MOH supported health centers that we do support to collect those already collected data that it has processed and get some more guidance on how we are doing on that, so “routinely collected data” is the data that normally you would get at the facilities that are collected on a daily basis.

Interviewer:	And so then, when you’re talking about the formal evaluations, for example with that project, that’s data that you actively take that’s not already included in the healthcare facilities… or do you also use routinely collected data in that case?

P14:	I would say it’s a mixture of both. There are definitely data that you find at the health facilities which is routinely collected, but since this was a new program and we wanted to… The indicators that we were tracking, we couldn’t find those in the already collected data so we had to define them and find a way of tracking those so that’s why we normally have to launch a specific evaluation project to track the success of those new innovative programs that you would rather not find their documentation in the health system.

Interviewer:	I see. And so, you briefly touched on it with the data officers, but how do you actually go about doing the evaluations and how do you collect that data? Like what methods do you use, like do you use surveys or focus groups, for example?

P14:	Yeah. … Surveys are the most common method we employ. Focus groups, we don’t use them that much. We mostly use surveys so we did questionnaires based on the project that we launched and then we send our data officers to collect data using those surveys.

Interviewer:	Okay. Just wanted to find out a bit more about the indicators that you track. Can you give me some examples of the outcome indicators that you do track?

P14:	Outcome indicators?

Interviewer:	Yeah, sorry. Just indicators that you track.

P14:	Yeah, basically for some programs that are intended to improve maternal health in the concentrated areas that we work, we track indicators, let’s say number of deliveries that we record at our facilities. We record number of prenatal consultation visits that we then do at our facilities. We track maternal death that we record in our facilities, so those are kind of examples of indicators that we normally track and at the end of the specific time, we know, “Oh! We’ve been able to increase deliveries from this level to this level,” meaning women that would rather be delivering in the communities are reducing so the facility utilization is increasing in that level.

When we see the number of prenatal consultation visits increasing, that means we are reducing the risk of maternal death in the districts where we work because the more we deliver at facilities where they can find trained birth attendants, the more they are exposed to less risk of having complications… that would put their life at risk while delivering in the communities, so that’s basically what we do.

Interviewer:	I see. It sounds like you have a lot of data that you’re keen on collecting to track your progress as an organization.

P14:	Oh, yeah, lots.

Interviewer:	What kind of resources does it take? It must be very time-intensive. It must take a lot of manpower as well, so do you possibly have an idea about how many people are involved in actually collecting the data then analyzing it? What kind of money do you put into evaluation monitoring?

P14:	Wow! That’s a great question and as I said, as an organization that was started in [REDACTED] you can imagine with the growth of our work if you don’t have a growing monitoring and evaluation team, it would be really hard to know where you’re coming from and where you are going and where you should be going so one of the things we have done is building an M&E team. So, our monitoring and evaluation team has grown at the same proportion of the growth that we are recording across the organization.

Our M&E team, we have an M&E team based in [REDACTED] that works very closely with our in-country M&E teams. This is a department that helps us to define measureable indicators on different programs that we run, track the progress, collect data on a regular basis, and guide us in the evaluation and analysis on different projects that we work so we have invested in building a monitoring and evaluation team. We have data officers and we have data analysts who help us do that. I mean, it’s a significant investment. We need to help people—full-time people— who work with the program managers who are implementing and support the organization in doing some studies on the work that is going on.

On the numbers, I don’t have the numbers ready for me on how much we pay across the organization or in [REDACTED], but [REDACTED]

Interviewer:	Wow! Wow! That’s quite a lot of people. Do you have a rough idea about the money that goes into M&E, say on an annual basis?

P14:	I don’t, no, but I can find out how much we are paying on that.

Interviewer:	Yeah, no. That would be great if the number’s accessible, but otherwise don’t worry about it. Just phenomenal amount of resources go into M&E.

P14:	At the end of the day, when you count how much you would be losing when you don’t have a team that is helping you really track and monitor the work you are doing and you compare it with how much you would rather invest in having people onboard to help you do that, the former is the worst.

As you know, many organizations depend on donor funding and this has been escalating. Even donors who will support our work really need to be convinced about where their money is going and really at the end of the grants, you have to prove to the donors—your supporters—that this is what the impact your money has done and I would say in the coming years organizations that are not moving towards this direction of really being evidence-based, they are going to face challenges in raising resources to support their work.

So not only are we pressured by the donor requirements, we also strongly believe that for us to invest in whatever field, we need to be guided by data to know exactly what is the burden of disease and how much does it cost us to get where we want to take people from and take them to which direction, so it’s kind of an internal belief and understanding, but also want to make sure that we align with our supporters are going to need to demand.

Interviewer:	Yeah, for sure. Sure. And that can be a challenge sometimes. But, it sounds like you have a very positive culture towards data collection, being able to measure the progress and the impact that you’re making. What kind of challenges do you face though in conducting the evaluations that you want to conduct or collecting the data that you want to collect?

P14:	The challenges are some of them are just like trying to maintain this platform that you build to help you track the progress. Again, you need to have enough resources to have well-qualified staff to help you do that, and one of the challenges that we face is basically our mission. We work in the most rural areas across the countries where we work. It’s been quite a challenge to retain and attract qualified staff to come and work with us in those rural areas. Managers, we try to provide attractive remuneration and incentives. It’s still an issue to balance that to have really qualified staff to come work with us in those mostly rural and remote areas, so that is one of the challenges.

The second challenge is having systematic softwares or databases where you would get all this information in the same area and can be easily accessed, so we work with the Ministry of Health and it has its own health management information system, but where all the routinely collected data is recording and this is the database for the Ministry so we normally have to fill in the paperwork to access data. We do not have a shared database that we can access anytime we need it so sometimes we are kind of strained by really going through all of those complications to access data, so those are the major ones I would highlight.

Interviewer:	Definitely.

P14:	The last one, sometimes we find the data quality really bad and we need to invest in more time to clear and screen and really process the data to be [informing? 00:25:42], so data quality is another issue that we normally test.

Interviewer:	Yeah. Can you define a bit more about what you mean by “data quality”? So, what is good data that your organization works with?

P14:	Data quality is… Basically, it depends on how they collect the data. … Let’s say you want to see patients—I keep using this indicator— the number of deliveries at [a] specific health facility and when you go to the database, you probably may not find deliveries that are tracked per specific healthcare institution. You may find they say “district data”; they may say “district deliveries.” When you want to know, go deeply into understand exactly how these deliveries spread across all the health institutions we have in the district, sometimes we *don’t* find that, and we need to dig through and go at each and every facility to find that data.

So, at that time, when you get like one stick of numbers for the whole district that doesn’t give you a clear picture of the whole district, then you wouldn’t find that data probably helpful for you based on what you are looking at, so that is the kind of data already… that modifying tool.

Interviewer:	I see. Thank you for that clarification; that’s very useful. So, P14, we’re coming up to our last two questions, so interested to hear...

P14:	I want to make sure that we’re on time. Were you planning on finishing in 10 minutes?

Interviewer:	Yes, definitely. We will try and finish this up very shortly.

P14:	Okay. Good.

Interviewer:	This question is about harm. With some health programs that get off the ground, particularly at the beginning, despite best intentions, they can cause harm. Is this something that you’ve thought of as a program officer and, if so, how have you taken steps to measure harm and prevent it?

P14:	To prevent the interventions that can cause harm?

Interviewer:	Yeah. Well, first of all, have you thought about how to measure this as a program officer for [REDACTED]?

P14:	To measure what?

Interviewer:	Harm— the potential harmful effects of a health program.

P14:	Can you give me just like one example of the harm you are referring to? I’m just trying to understand.

Interviewer:	Yes. Say with a health program that opens a clinic and say with particular doctors who they train, they might not have a proper training protocol that comes through and some people might fall through the cracks and deliver maybe a program that’s quite different than what the program initially wanted to deliver, and sometimes maybe the wrong drugs are prescribed, for instance, and that harms the community who they’re working with, so that’s one example of harm.

Some other examples might be like environmental harm or harm to the border community such as harm to the waterways and things like that, so there’s some examples, but...

P14:	That’s a great question. I would just give one example, like every doc. We normally hire international doctors to come and take care of patients at all of these clinics that we support and one of the criteria that the country requires is that anyone who is coming to practice medicine in the country has to get a medical license. So for you to get even the medical license, then you have to put in your paperwork and show your qualifications and how you are qualified to be eligible and capable of providing healthcare to the patients, so that is one check that we normally have so when we deploy our staff, we know these are the staff that are certified and allowed to practice medicine in the country so that if there is any harm that can be caused on the patient, we know how to track that.

Another example is everything we import into the country, including medication, has been certified by the [REDACTED] Bureau of [REDACTED] so there is not any medication that can be used in the country without pressing through certification—the country’s certification of medication—so using that medication, we know it’s focused on the standards, it’s certified to be used in the country, and then with that, we assure we are reducing all of the risks that medication can give to a patient so those are kind of examples that I would highlight. I don’t know if I have an answer to each.

Interviewer:	I see. Sometimes it’s difficult to know if a program has potential harm, particularly before it started like with new initiatives. I guess the question that I’m really trying to get at is there some kind of way tat you try and account for that in your data collection or evaluation which will try and capture how the program could potentially be harmful, if it is harmful at all?

P14:	Yeah. The programs we run in the country are mostly health programs and we work through the existing health system structure. We are working through the institutions—health centers and hospitals that are Ministry of Health facilities—and they have established rules and regulations you have to go through and that’s what we follow. What we have been measuring are basically the challenges that we face and what our partners face and how we can meet to get those.

For the harmful aspect, I think you are referring to the [inaudible 00:32:44] risk mitigation aspects. You normally would do that when you are developing a project. There are no other ‘checks and balances’ that we basically put in place other than following the already predefined country rules and regulations. That’s what we normally follow. And again, in the health system structure, there are automatic. When you want to use a medication, it is a medication that is approved by international standard and national standard. There’s nothing else you would do. You are using medication that has an international certification.

Medical equipment’s the same. They go through inspection. The staff, they have their formal certification of their qualifications that allow them to do what they may be doing. So, that’s kind of what I think we have interest, but there is no other ways/communications that I would think of that we normally use.

Interviewer:	Okay, cool. That’s great. Final question: In a perfect world, what do you think would help you do your work better, particularly in relation to creating evidence to show the impact of your programs? What would help you do your work better?

P14:	That’s a great question. I think I would talk more on the non-government organizations like our organisation which, again, depend on donor funding and what we have done basically at our organisation [is] to start building this M&E platform. We’ve done this out of our unrestricted funding and one of the challenges we face in the proposals and projects we normally [devolve? 00:35:02] for donor funding, some people don’t understand the need of investing in M&E and when you try to build in some costs to support the M&E framework, some organizations, our donors don’t understand that. They think that would be counted out of the overheads of the organization as opposed to being part of the project, so if we can do more advocacy on that, for people to understand really why this should be cost-ed or taken as project costs that is really critical to the success of the project, that would be great.

The other thing that I would think of as really building sophisticated… I know there are a couple that exist, but basically having sophisticated databases that data can be tracked through. At our organisation, we are using [REDACTED] platform which is helping us to find one place where we share data and I think it’s great for us, but I would think of more sophisticated software that would help track data or find data at the same place, be accessed by people, have security of data that you have. Yeah, that’s what I would think of.

Interviewer:	That’s great. Well, hopefully we’ll be able to get onto that within the next couple of years because that seems like an extremely important aspect to have, particularly when you’re trying to collect that data and then have it in a central depository, to be able to analyze it efficiently, and then be able to feed it back through. So, yeah, extremely important. I can’t agree with you more.

P14:	Okay.

Interviewer:	So, P14, that brings us to the end of the interview. Thank you so much for your time. Is there anything else that you wanted to add or do you have any questions?

P14:	No.

[end of recording: 00:37:29]
