# Supplementary material for: Supporting Better Evidence Generation and Use within Social Innovation in Health in Low- and Middle-Income Countries: A Qualitative Study
Source: PLoS One. 2017 Jan 26;12(1):e0170367. doi: 10.1371/journal.pone.0170367 (PMC5268497; doi:10.1371/journal.pone.0170367)
Supplement: S1 Dataset — (ZIP) [file pone.0170367.s002.zip › Data/Data - Interview transcripts/P9.doc]

Interviewer:	So do you think… I obviously know what your organisation does but for the benefit of the recording just to have in your own words, tell us about what’s the problem the your organisation is trying to solve. What do you do?

P9:	[REDACTED]

Interviewer:	Awesome. When you’re thinking about I guess accessing and changing those paradigms and getting that done, how do you define success in that mission?

P9:	I think there’s a lot of different ways that we do that, some of which probably seem fairly obvious. The most immediate metric is of course the well-being of the patients that we serve so when we see better health outcomes in our [?-naries 00:01:50] and our districts that’s certainly an immediate measure of success. I think when we get new knowledge out into the world about how to do that, that’s another metric that we track.

When we serve… As I said before, when we have tangible results around medical education and training, showing that more people are coming into the profession oriented in a particular way to work, I’d say that’s another metric, and of course policy changes. We do work on that in a number of different ways, many of which are quieter, but we’re always part of coalitions that are trying to drive broader-level system change and so although that’s a little harder to say that’s all because of us, we certainly know the things that we’re participating in and internally at least we do take pride in it and keep track of those sorts of things that we’re trying to influence.

I’d say the money we raise and the number of people who kind of join up part of our movement would be another metric. There’s organizations that are inspired by and often incubated by our organisation. That would be another way in which we see ourselves building and growing the field, so there’s a lot of different ways that we measure success.

Interviewer:	Yeah, that was a broad… and multi-layered answer to that question. When you talk about some of those things, how people are oriented when they go in to do this work or spreading messages or… What are some of the ways? You said, “We track A, we track B, we track C, we track D.” What are some of the ways in which you track the five or six or seven things you just mentioned?

P9:	Well… with regard to educating the next generation, I guess I’m not sure we would say that we *track* things that happen kind of down the road. That is to say we train a doctor. Five years later, we’re looking to see that that doctor is doing things in the way that we want them to. Obviously, we’re not following through to that degree. I think we have just more faith and confidence that when doctors are trained in our methodology that they then have a different orientation to work when they go out to practice and so the metrics for something like that would be much more about successful participation in various degree and certification programs that we run or long-term training programs that we run.

So just to give a concrete example, we have in [REDACTED] … We are strengthening an existing system of first-year doctors out of med school who have to do public service for a year often get drop in rural clinics without any preparation or training, so the program we created there is designed to really reinforce that and these doctors go through a [REDACTED] curriculum to really change their attitudes and orient them towards global health delivery in a very particular way and I think by the end of those courses they have to demonstrate that they have a deeper understanding of that and we trust that they’ll carry it forward so that would be a proxy measure at some level, but there certainly are many tens or hundreds of doctors and nurses and clinicians who’ve gone through those kinds of training programs, so we feel like in the long run, it is a generational play that we’re making and that’s why we focus not just on the health delivery, but on the educational side of the equation as well.

Things like policy and stuff like that, again, it’s super, super hard to track the specific influence that our organisation has. For example, I’m establishing a fund, but [REDACTED] and others were at the table for all of those discussions and helping to shape those funding mechanisms and those are things we continue to try to do in a variety of different ways.

We always work in the countries where we work. We’re always working at a national level to influence and inform policy and I think there are ways we could point to that. I’m not sure the metrics are as simple as they are say for… Or not simple, but they’re not as [inaudible 00:06:33] for like tracking the number of people we have on ART drugs in rural [REDACTED]. That’s a little easier to pinpoint.

Interviewer:	Right, of course. Obviously influence… Those sort of things are tougher to track or to decipher it from other factors. Does your organisation have a dashboard— a series of five to ten indicators that you guys look to in board meetings or annual reviews or things like that to say, “In terms of capturing our most important priority, some can’t be put into numbers or a qualitative summary, but some can and here are the ones that can and here’s how we track them?”

P9:	Not really. I think… our different country sites track a kind of core set of metrics that are adapted to that local context, so for example in [REDACTED], they can tell you... They put some particular focus on generating new knowledge and research so we have a research group in [REDACTED] that’s been built up over time through our partnership with [REDACTED]. They’re tracking every year the number of papers that are being produced, the number of papers that are being first-authored by local [REDACTED] clinical staff and the numbers are quite impressive, but that’s not something that’s being tracked in a similar way in other countries so we don’t really have a global metric for that even though we all understand that something like that is a shared goal, ultimately.

Likewise, we have in our strategic plan because we’re much more of a federated network organization than we are a sort of a hierarchical, centralized organization, we tend to operate more against a shared set of long-term goals and operating principles and then different sites are moving in different ways to try to get down that path, but we do (in our plan) have what I would call four fundamental clinical goals that do tend to orient a lot of our conversation, particularly in the countries where we’re doing the most direct primary care delivery, and those are…

There are four of them. It’s reaching zero maternal deaths in childbirth, reaching zero death from tuberculosis, zero mother-to-child transmission of HIV, and zero death from malnutrition for children under 5, so those are four what we call kind of aspirational goals that were designed by our global delivery health partnership across [REDACTED], [inaudible 00:09:39], and then our organisation and the reason that the group came up with them is because we felt that, collectively, they would essentially drive the develop of a strong health system but if you had a system that could do those things, it would do almost everything else you needed it to do to provide good healthcare to poor people in remote settings.

We’ve tried at various times to kind of collect regular data against those four outcomes and we’ve sort of stuttered and faltered, partly because our data systems aren’t robust enough to do it, but everybody has those in mind and everybody is moving towards those and everybody is sort of tracking those in their own way. There are many other goals in the plan, but I would say those four are very oriented for everybody.

Interviewer:	Awesome, cool, so I want to talk about the influence goals and these clinical goals in turn, maybe starting with the latter. It was interesting how you framed the goals, not that we want to achieve these metrics, but that *if* we’re achieving these metrics they’ll probably find something about the health system strengthening at large, right?

P9:	Yeah.

Interviewer:	Which is not how maybe a lot of folks would frame them and that’s interesting. Given that, are the goals… Do you have the rates of those four indicators in all of your federated partners, all your partner country organizations?

P9:	Yeah.

Interviewer:	Or is it more that they all try to do things that are oriented towards those goals in different ways?

P9:	We do have data on all of those areas. I would say that the data is the strongest for HIV and TB. Malnutrition’s probably the hardest one and the most inconsistent in terms of data quality… And again, some sites have much more robust data than others, but I would say at least particularly in... Well, taking the two new [REDACTED] sites out of the mix for just a moment because they’re so new, but the other [REDACTED] major implemental sites—[REDACTED]—all are tracking information in these areas and then each of them might also have some additional metrics around some other kind of high-burden areas in those countries.

[REDACTED], which has the worst health outcomes of all the countries, is pretty focused on HIV, TB and maternal deaths even though they have malnutrition obviously as well, those are really the key ones. Malawi is much more about HIV and maternal death and malnutrition. Haiti tracks all of them, so we do have data around all of those areas.

Interviewer:	How is that collected? Is it different in every country? Is it using…

P9:	We have local M&E teams that do all of that data collection. We have a M&E team in [REDACTED] that is kind of reinforcing and capacity-building a team that works with those local teams to make sure that they’re looking at data and data quality. That team also pulls, aggregates information across sites and things like that.

We tried for a little while to kind of create a dashboard with all four of those metrics and look at them. We called them cross-site indicators. The problem is that they were… When you do that, it tends to be kind of like interesting, but not very actionable and I think the sites, at least in the our organisation universe, everybody is so oriented to action that they felt like, “God! This is taking a lot of time for us to produce this generic report for cross-site indicators and we’re not getting a lot in return for that. We kind of know what our data is and we’re thinking programmatically about how to sustain that or get better, and so we prefer to just sort of share what we have and stay focused on what we need to do.” We kind of have backed off a little bit on forcing it into a kind of single rubric, if you will.

Interviewer:	Yeah. What were some of the struggles of trying to do that and what were the costs… You referenced kind of cost benefits of trying to take that and it just not being worth it. What were some things at play?

P9:	Our M&E team would be in a much better position and I’m sure would be happy to kind of give you some more detail on what the struggles were around that. My impression from where I sat was it just took a lot of time from the M&E teams in the country to kind of pull data together and scrub it with the central team in [REDACTED] in a certain way and verify the reports. In the meantime, they’re just busy trying to make sure that the data is being collected on a daily basis for the grants we have to report on and other things like that, so I think it was just... and the teams tend to be pretty thin in the field and so I think they just felt like, “You guys kind of already know this even though it’s not in a format and it’s just taking a lot of our time.” That’s about as good an answer as I can give you without handing you off for more detail to our team.

Interviewer:	No, no worries. That is helpful. Let’s zoom out of the clinical stuff and go back to the policy influence stuff ‘cause that’s probably through all that your organisation has been involved in direct patient treatment for... Gosh! [REDACTED] years?

P9:	Yeah.

Interviewer:	It’s the paradigm changing work that maybe it’s most famous for, so I guess… I’m not even sure if that’s fair, but when it comes to that sort of thing, what other takeaways from the different types of engagement that your organisation has had over the years with Ministry of Health or international health bodies vis-à-vis, what helps to convince people that this is the way things should be done? What types of tactics, data, schmoozing do the your organisation clan employ in those discussions, be they large scale or small scale?

P9:	Yeah. Well, I think they are a couple of kind of top-line answers to that. One is I think a lot of our advocacy success has come from the fact that we’re a solidarity-based organization. We have a lot of credibility at the community level and a lot of our political clout is generated by that. It’s generated by our understanding of that ability to speak on behalf of the communities that we serve, so we’re a grassroots organization.

At the same time, we’re a global organization, and I think that’s a very unusual combination, particularly in countries. That gives us a very, very different kind of credibility than let’s say, just one of a more typical global NGO that might have its headquarters in the capital city, hired some local staff, but one always has the sense that they’re only a grant away from leaving. And so, we have this incredible ability to be patient as an organization.

[REDACTED] a really very good example. We sort of toiled away in the mountains for 7 or 8 years and we’ve got very good outcomes working in a handful of clinics. Governments came and governments went and we were sort of not in favor and the major partners like [REDACTED] and [REDACTED] and others... and we were kind of on the margins, but eventually our results kind of came to people’s attention and people realized that we really were out there in the mountains working day in and day out and when we were able to show some of the outcomes and the data, the government at one point finally said, “Hey, wait a minute! This is amazing! Like could you do this up scale?” and so we’re now in the midst of working hand in hand with them.

It’s a tricky setting. It’s not easy, but we’re trying to reform the entire system around the our organisation model and I think you can’t do that unless you have people’s trust and that requires not only being there but also spending time. Our team—our executive team in the city—just spends hours and hours and hours building relationships and getting to know people and building alliances and so forth and eventually that work starts to pay off. In [REDACTED], our executive director would be up till 2, 3 in the morning having a drink, talking strategy with the Minister of Health because we have a personal relationship with them. So, I think that’s been probably the biggest thing.

But the other thing and I think this is really, critically important and [REDACTED] has always seen this from the very beginning is that there is a deep and critical connection between research and change, so you can work with 60 or 70 patients in [REDACTED] but if you don’t write a great research paper about it, nobody ever knows about it and nothing ever changes. But if you have that [REDACTED] connection and you write the research paper, you start to show that we’re getting better outcomes in rural [REDACTED] than we are in urban [REDACTED], then it starts to get people’s attention when it’s published in a peer-review journal and that gives him credibility and our other clinical leaders the credibility to then speak at panels and advocate and start to build a reputation so I think the other really key piece is the generation of knowledge. I think that has positioned our organisation as strongly as any other factor.

Interviewer:	[REDACTED] lot of the early papers were qualitative papers about the experience of patients, but then there were also influential papers about MDR-TB and HIV or ARvs in low-income settings or low-visa settings that were more clinical-outcome oriented.

P9:	Yeah.

Interviewer:	… Is the way in which that type of research is conducted, used, valued different? Does some get employed at different times or do some people like the qualitative stuff better than the quan. stuff or vice versa? Are there conversations about that that are had?

P9:	I think what we as an organization… and I think [REDACTED] feels very strongly about this and therefore what we as an organization try to ensure is that operational research begin to gain credibility in the same way that more so called scientific research does and we do both. We have researchers who have NIH grants and are doing sort of blind studies and that sort of thing. I think that’s been a big part of our success, particularly around TB, but we also are generating a lot of papers, just about how to do things and I think [REDACTED] been working through Harvard to try to legitimize operational research as an academic pursuit, which is a little different than simply the qualitative kind of things but it’s more related to qualitative in a lot of ways.

I think that’s an ongoing debate in the Academy because the Academy only rewards hard science. It doesn’t seem to take the other as seriously, so I think that is a robust debate. It’s certainly one that’s ongoing and we obviously have a very strong view on the value of operational research.

Interviewer:	Yeah, that’s certainly a challenge. [REDACTED]

P9:	Exactly, exactly. I think one of the things they’re working on at [REDACTED] is trying to push towards recognizing good, operational research as part of someone’s academic portfolio so that that gets rewarded in the tenure system. Talk about a hard change! That really is a long slough, but I think [REDACTED] and [REDACTED] and others feel as though if we could eventually get [REDACTED] to recognize this and to adapt a little bit, it could change the whole academic marketplace. If [REDACTED] did it, a lot of other places would follow.

Interviewer:	Absolutely, so that’s one way in which one system’s change that would enable your organisation to do its work better, to get more people and NIH grants to do the type of stuff that they feel is important, this changing of the tenure system… What are other things that sort of in a perfect world, what would that world look like? What are things that would help you do that type of research, that type of work better?

P9:	Well I mean...

Interviewer:	Could be structural, could be small-scale. Sorry.

P9:	I think certainly creating more obvious academic pathways for researchers and clinicians who want to come in to this work… What I hear often is people have to find their own way and carve their own path. It’s very challenging. Institutions don’t have the built-in flexibility to allow this kind of work to happen very easily, so I do think the more we can facilitate that through things like changes to the tenure system or flexibility in hospital schedules or a variety of other things like that. Just as [REDACTED] been able to in a more idiosyncratic way with the breakdown at [REDACTED]. The more other institutions pick up similar structures like that, the more it would open those pathways so I think those cluster of things would be of great value.

I think the other thing is building that capacity in the countries where we work, working with local institutions from the very beginning as they build their professional cadre of human resources from the get-go, these sort of things are built in as values so we’re working hard to establish research hubs in all of our major sites and we’re building a university of global health in [REDACTED] that will be much more oriented around all of this, so I think we’re working on multiple levels just to try to create models that other people can emulate... sort of like [REDACTED] did with the residency program. I think that is also another example of a program that’s been very popular in that other institutions look to copy in different ways.

Interviewer:	Great. Last question. Just like I said, if you want to keep this to 30 minutes that we initially bargained from ya, and I’m sorry to end this in sort of a… a sadder note, but it’s important nonetheless, and so I guess one of the things we’re looking at is does your organisation ever think about ways in which its programs could be harmful? And if that sounds like a loaded question, but I guess just in the context of social interventions that have been done for the right reasons but have unintended consequences and this is something [REDACTED] teaches about that aren’t good for the people who it’s supposed to provide benefit. What types of systems or inquiries does your organisation have in place or set up to think about potential harms, that attract potential harm?

P9:	I’m a little stuck to answer that. I think there’s times when we don’t always do our work as well as we’d like. In that sense, there’s a kind of harm that comes if we don’t have the medicine we need or if we don’t have a car to get someone where they need to go, so there’s that kind of harm. It would be hard to argue that that’s worse than the alternative which is to not be there.

I think we get criticized all the time for doing work in a way that isn’t sustainable and so I suppose that’s an oblique attempt to suggest that we’re potentially causing harm ‘cause we’re doing things the system can’t sustain. I think on its face that’s a ridiculous argument, but that is an accusation that’s often leveled at us. I guess I could acknowledge that some people might think that and I know most of us at our organisation would disagree pretty strongly with that, so I don’t think it does do harm… I don’t know. I don’t know. This is… the hardest kind of work one does. There are other domains that have an equal share in education, criminal justice… other areas that entrench poverty and degradation, but I think in the world of health, what we’re doing is...I’d hate to sound self-righteous, but it’s fairly noble.

Interviewer:	Yeah, cost-effectiveness… sustainability...

P9:	Yeah. It’s a ridiculous argument because if you look at [REDACTED], they’re spending as a percentage of GDP a much higher percentage than almost any other kind of [REDACTED] country and they’re getting amazing outcomes. It’s not a lot of dollars, but it’s driving enormous societal change and benefit so it’s just a stupid argument. It makes no sense. We always make moral choices about how we allocate resources and the idea that we couldn’t allocate more is just absurd. And not to mention, when you actually implement our organisation’s model appropriately, you actually spend less than you do in the developed world so the people that throw that Molotov cocktail I think are just… not coming from the right place.

[end of recording: 00:31:50]
